# Supplementary material for: Naegleria fowleri: Protein structures to facilitate drug discovery for the deadly, pathogenic free-living amoeba
Source: PLoS One. 2021 Mar 24;16(3):e0241738. doi: 10.1371/journal.pone.0241738 (PMC7990177; doi:10.1371/journal.pone.0241738)
Supplement: S1 File — (ZIP) [file pone.0241738.s002.zip › 6aqy_full_validation.pdf]

Full wwPDB X-ray Structure Validation Report (i)

May 23, 2020 – 05:48 am BST

PDB ID	:	6AQY
Title	:	Crystal structure of a gdp-l-fucose synthetase from Naegleria fowleri
Authors	:	Seattle Structural Genomics Center for Infectious Disease; Seattle Structural Genomics Center for Infectious Disease (SSGCID)
Deposited on	:	2017-08-21
Resolution	:	2.55 Å(reported)

This is a Full wwPDB X-ray Structure Validation Report for a publicly released PDB entry.

We welcome your comments at *validation@mail.wwpdb.org*
A user guide is available at
https://www.wwpdb.org/validation/2017/XrayValidationReportHelp
with specific help available everywhere you see the (i) symbol.

The following versions of software and data (see references (i)) were used in the production of this report:

MolProbity	:	4.02b-467
Mogul	:	1.8.5 (274361), CSD as541be (2020)
Xtriage (Phenix)	:	1.13
EDS	:	2.11
Percentile statistics	:	20191225.v01 (using entries in the PDB archive December 25th 2019)
Refmac	:	5.8.0158
CCP4	:	7.0.044 (Gargrove)
Ideal geometry (proteins)	:	Engh & Huber (2001)
Ideal geometry (DNA, RNA)	:	Parkinson et al. (1996)
Validation Pipeline (wwPDB-VP)	:	2.11

1 Overall quality at a glance (i)

The following experimental techniques were used to determine the structure:
X-RAY DIFFRACTION

The reported resolution of this entry is 2.55 Å.

Percentile scores (ranging between 0-100) for global validation metrics of the entry are shown in the following graphic. The table shows the number of entries on which the scores are based.

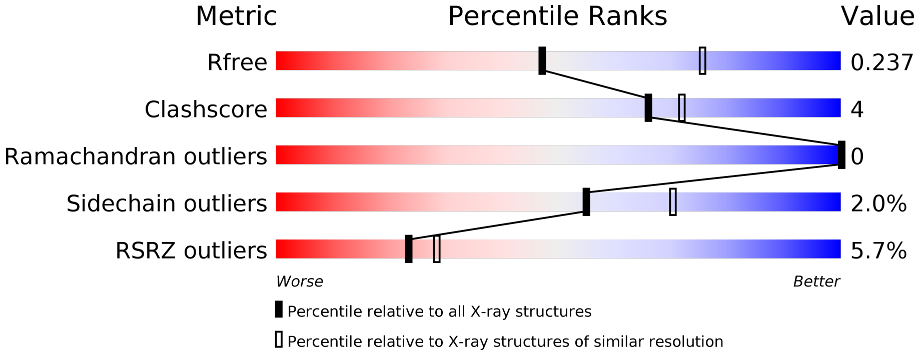

Metric	Whole archive (#Entries)	Similar resolution (#Entries, resolution range(Å))
R_{free}	130704	1284 (2.56-2.52)
Clashscore	141614	1332 (2.56-2.52)
Ramachandran outliers	138981	1315 (2.56-2.52)
Sidechain outliers	138945	1315 (2.56-2.52)
RSRZ outliers	127900	1272 (2.56-2.52)

The table below summarises the geometric issues observed across the polymeric chains and their fit to the electron density. The red, orange, yellow and green segments on the lower bar indicate the fraction of residues that contain outliers for >=3, 2, 1 and 0 types of geometric quality criteria respectively. A grey segment represents the fraction of residues that are not modelled. The numeric value for each fraction is indicated below the corresponding segment, with a dot representing fractions <=5% The upper red bar (where present) indicates the fraction of residues that have poor fit to the electron density. The numeric value is given above the bar.

Mol	Chain	Length	Quality of chain
1	A	341	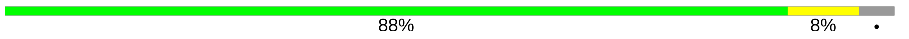
1	B	341	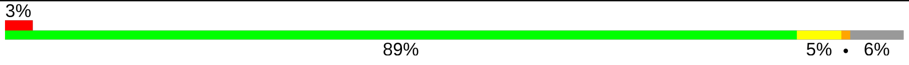
1	C	341	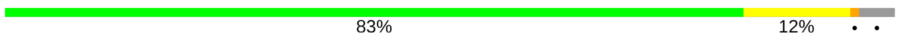
1	D	341	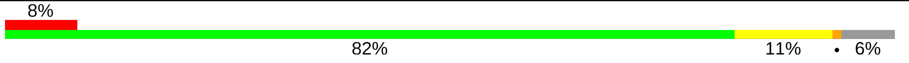
1	E	341	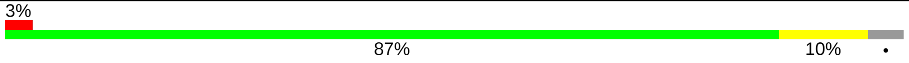
1	F	341	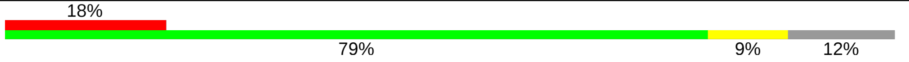

The following table lists non-polymeric compounds, carbohydrate monomers and non-standard residues in protein, DNA, RNA chains that are outliers for geometric or electron-density-fit criteria:

Mol	Type	Chain	Res	Chirality	Geometry	Clashes	Electron density
3	CL	A	402	-	-	-	X
3	CL	E	402	-	-	-	X

2 Entry composition (i)

There are 4 unique types of molecules in this entry. The entry contains 14671 atoms, of which 0 are hydrogens and 0 are deuteriums.

In the tables below, the ZeroOcc column contains the number of atoms modelled with zero occupancy, the AltConf column contains the number of residues with at least one atom in alternate conformation and the Trace column contains the number of residues modelled with at most 2 atoms.

- Molecule 1 is a protein called gdp-l-fucose synthetase.

Mol	Chain	Residues	Atoms					ZeroOcc	AltConf	Trace
			Total	C	N	O	S			
1	A	328	2542	1629	420	476	17	0	0	0
1	B	322	2499	1605	411	467	16	0	1	0
1	C	326	2545	1633	421	475	16	0	0	0
1	D	322	2366	1508	396	446	16	0	0	0
1	E	329	2535	1620	422	476	17	0	0	0
1	F	299	2012	1265	345	388	14	0	0	0

- Molecule 2 is SULFATE ION (three-letter code: SO4) (formula: O_4S).

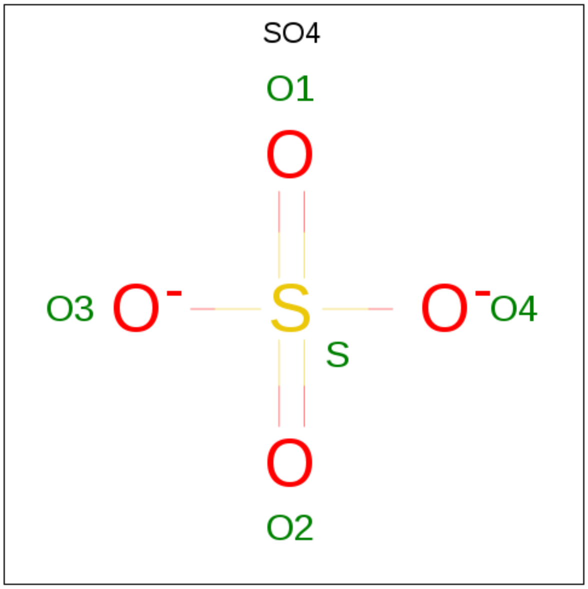

Mol	Chain	Residues	Atoms	ZeroOcc	AltConf
2	A	1	Total O S<br>5 4 1	0	0
2	B	1	Total O S<br>5 4 1	0	0
2	C	1	Total O S<br>5 4 1	0	0
2	E	1	Total O S<br>5 4 1	0	0

- Molecule 3 is CHLORIDE ION (three-letter code: CL) (formula: Cl).

Mol	Chain	Residues	Atoms	ZeroOcc	AltConf
3	A	1	Total Cl<br>1 1	0	0
3	E	1	Total Cl<br>1 1	0	0

- Molecule 4 is water.

Mol	Chain	Residues	Atoms	ZeroOcc	AltConf
4	A	50	Total O<br>50 50	0	0
4	B	44	Total O<br>44 44	0	0
4	C	18	Total O<br>18 18	0	0
4	D	4	Total O<br>4 4	0	0
4	E	30	Total O<br>30 30	0	0
4	F	4	Total O<br>4 4	0	0

3 Residue-property plots

These plots are drawn for all protein, RNA and DNA chains in the entry. The first graphic for a chain summarises the proportions of the various outlier classes displayed in the second graphic. The second graphic shows the sequence view annotated by issues in geometry and electron density. Residues are color-coded according to the number of geometric quality criteria for which they contain at least one outlier: green = 0, yellow = 1, orange = 2 and red = 3 or more. A red dot above a residue indicates a poor fit to the electron density (RSRZ > 2). Stretches of 2 or more consecutive residues without any outlier are shown as a green connector. Residues present in the sample, but not in the model, are shown in grey.

- Molecule 1: gdp-l-fucose synthetase

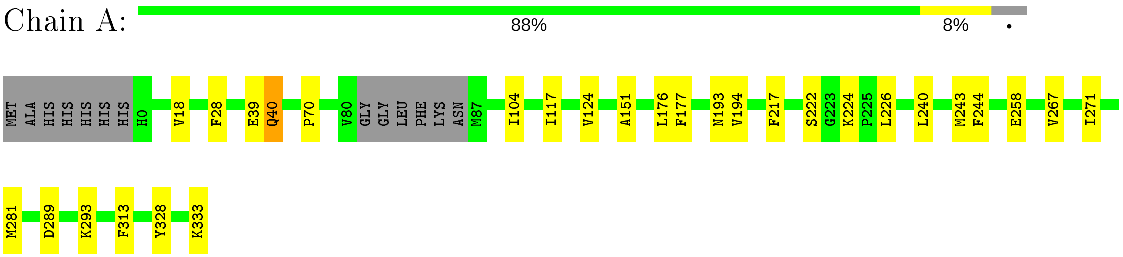

- Molecule 1: gdp-l-fucose synthetase

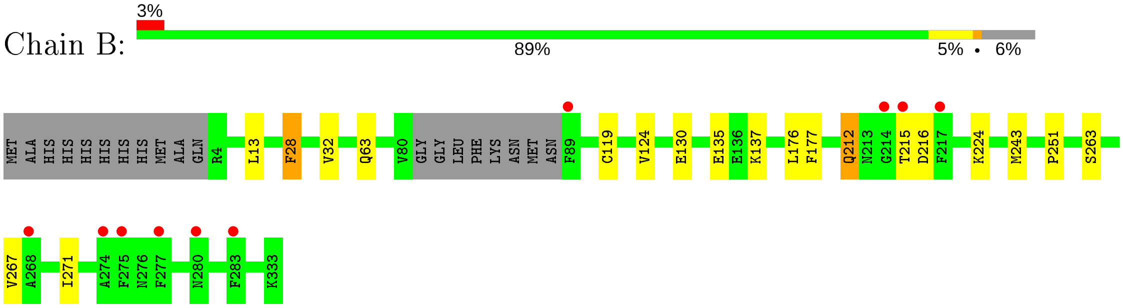

- Molecule 1: gdp-l-fucose synthetase

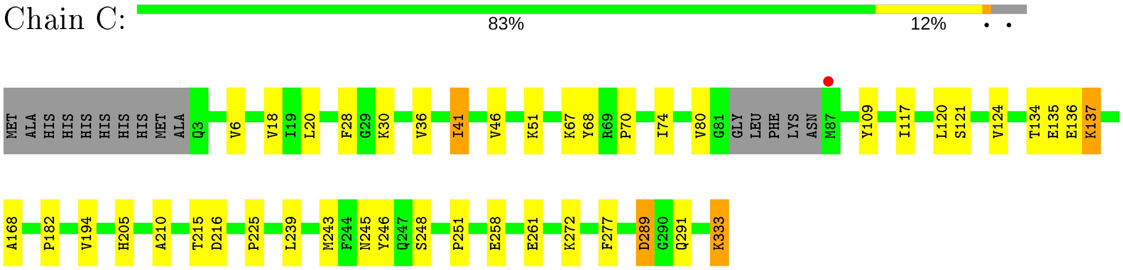

- Molecule 1: gdp-l-fucose synthetase

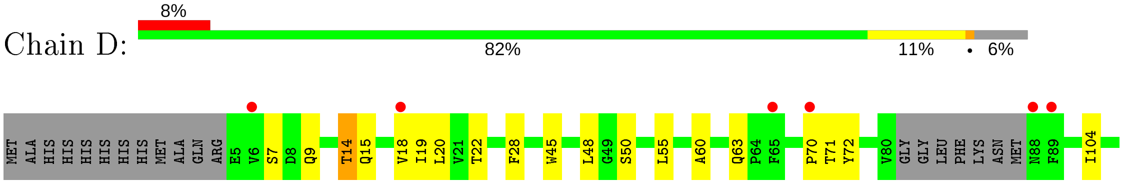

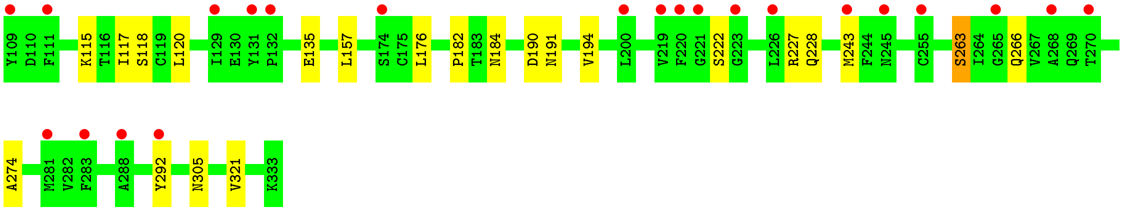

- Molecule 1: gdp-l-fucose synthetase

Chain E:

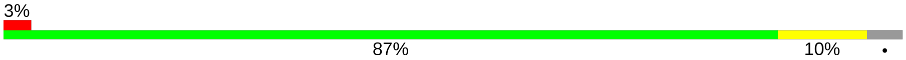

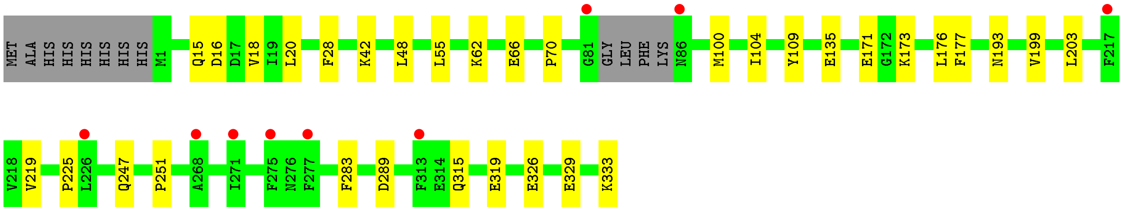

- Molecule 1: gdp-l-fucose synthetase

Chain F:

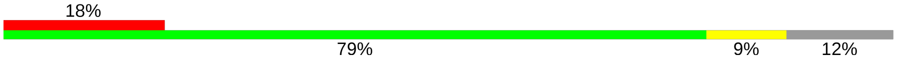

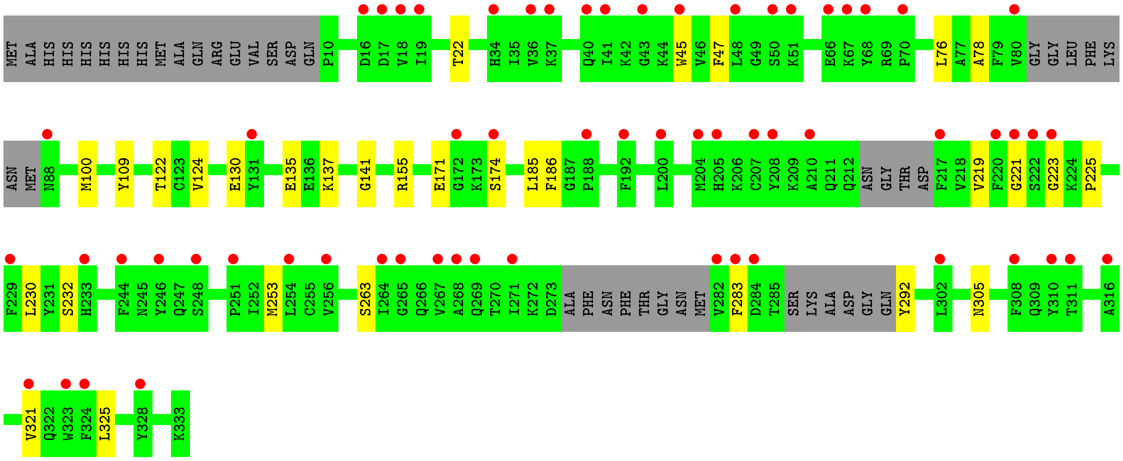

4 Data and refinement statistics

Property	Value	Source
Space group	P 1 21 1	Depositor
Cell constants<br>a, b, c, α, β, γ	99.73Å 102.10Å 121.82Å<br>90.00° 107.63° 90.00°	Depositor
Resolution (Å)	35.56 – 2.55<br>46.73 – 2.55	Depositor<br>EDS
% Data completeness<br>(in resolution range)	99.8 (35.56-2.55)<br>99.8 (46.73-2.55)	Depositor<br>EDS
R_{merge}	0.05	Depositor
R_{sym}	(Not available)	Depositor
$< I/\sigma(I) >$ [1]	2.67 (at 2.54Å)	Xtriage
Refinement program	PHENIX (1.12_2829: ???)	Depositor
R, R_{free}	0.186 , 0.240<br>0.186 , 0.237	Depositor<br>DCC
R_{free} test set	2059 reflections (2.72%)	wwPDB-VP
Wilson B-factor (Å^2)	57.5	Xtriage
Anisotropy	0.091	Xtriage
Bulk solvent k_{sol}(e/Å^3), B_{sol}(Å^2)	0.30 , 60.3	EDS
L-test for twinning[2]	$< \|L\| >$ = 0.50, $< L^2 >$ = 0.33	Xtriage
Estimated twinning fraction	No twinning to report.	Xtriage
F_o,F_c correlation	0.95	EDS
Total number of atoms	14671	wwPDB-VP
Average B, all atoms (Å^2)	71.0	wwPDB-VP

Xtriage's analysis on translational NCS is as follows: *The largest off-origin peak in the Patterson function is 3.09% of the height of the origin peak. No significant pseudotranslation is detected.*

[1] Intensities estimated from amplitudes.
[2] Theoretical values of $< |L| >$, $< L^2 >$ for acentric reflections are 0.5, 0.333 respectively for untwinned datasets, and 0.375, 0.2 for perfectly twinned datasets.

5 Model quality (i)

5.1 Standard geometry (i)

Bond lengths and bond angles in the following residue types are not validated in this section: SO4, CL

The Z score for a bond length (or angle) is the number of standard deviations the observed value is removed from the expected value. A bond length (or angle) with $|Z| > 5$ is considered an outlier worth inspection. RMSZ is the root-mean-square of all Z scores of the bond lengths (or angles).

Mol	Chain	Bond lengths		Bond angles	
		RMSZ	$\#\|Z\| > 5$	RMSZ	$\#\|Z\| > 5$
1	A	0.45	0/2608	0.59	0/3544
1	B	0.48	1/2568 (0.0%)	0.59	0/3491
1	C	0.45	0/2610	0.59	0/3540
1	D	0.35	0/2428	0.51	0/3318
1	E	0.44	0/2599	0.55	0/3532
1	F	0.35	0/2060	0.51	0/2821
All	All	0.43	1/14873 (0.0%)	0.56	0/20246

All (1) bond length outliers are listed below:

Mol	Chain	Res	Type	Atoms	Z	Observed(Å)	Ideal(Å)
1	B	119	CYS	CB-SG	-6.18	1.71	1.82

There are no bond angle outliers.

There are no chirality outliers.

There are no planarity outliers.

5.2 Too-close contacts (i)

In the following table, the Non-H and H(model) columns list the number of non-hydrogen atoms and hydrogen atoms in the chain respectively. The H(added) column lists the number of hydrogen atoms added and optimized by MolProbity. The Clashes column lists the number of clashes within the asymmetric unit, whereas Symm-Clashes lists symmetry related clashes.

Mol	Chain	Non-H	H(model)	H(added)	Clashes	Symm-Clashes
1	A	2542	0	2371	18	0
1	B	2499	0	2330	11	0
1	C	2545	0	2416	29	0
1	D	2366	0	2060	20	0

Continued on next page...

Continued from previous page...

Mol	Chain	Non-H	H(model)	H(added)	Clashes	Symm-Clashes
1	E	2535	0	2348	20	0
1	F	2012	0	1541	16	0
2	A	5	0	0	0	0
2	B	5	0	0	0	0
2	C	5	0	0	0	0
2	E	5	0	0	0	0
3	A	1	0	0	0	0
3	E	1	0	0	1	0
4	A	50	0	0	0	0
4	B	44	0	0	0	0
4	C	18	0	0	0	0
4	D	4	0	0	0	0
4	E	30	0	0	0	0
4	F	4	0	0	0	0
All	All	14671	0	13066	110	0

The all-atom clashscore is defined as the number of clashes found per 1000 atoms (including hydrogen atoms). The all-atom clashscore for this structure is 4.

All (110) close contacts within the same asymmetric unit are listed below, sorted by their clash magnitude.

Atom-1	Atom-2	Interatomic distance (Å)	Clash overlap (Å)
1:A:328:TYR:O	1:A:333:LYS:NZ	1.99	0.94
1:F:141:GLY:O	1:F:155:ARG:NH2	2.12	0.82
1:B:130:GLU:O	1:B:137:LYS:NZ	2.22	0.72
1:F:124:VAL:O	1:F:155:ARG:NH1	2.23	0.72
1:C:225:PRO:HG2	1:C:289:ASP:HB2	1.70	0.72
1:D:55:LEU:HD13	1:D:104:ILE:HD11	1.72	0.70
1:D:274:ALA:HB1	1:D:321:VAL:HG21	1.72	0.70
1:B:13:LEU:HD11	1:B:243:MET:HE3	1.79	0.64
1:A:258:GLU:OE2	1:A:293:LYS:NZ	2.30	0.62
1:A:240:LEU:HD23	1:A:243:MET:HE2	1.82	0.61
1:D:115:LYS:HE2	1:D:176:LEU:HD23	1.84	0.60
1:B:215:THR:HG22	1:B:216:ASP:H	1.65	0.60
1:F:130:GLU:O	1:F:137:LYS:NZ	2.37	0.58
1:F:109:TYR:OH	1:F:171:GLU:OE1	2.19	0.57
1:E:199:VAL:HG12	3:E:402:CL:CL	2.41	0.57
1:F:45:TRP:HB3	1:F:47:PHE:HE1	1.69	0.56
1:A:222:SER:OG	1:A:289:ASP:OD2	2.23	0.56
1:C:215:THR:OG1	1:C:216:ASP:N	2.39	0.56
1:A:267:VAL:O	1:A:271:ILE:HG12	2.06	0.55

Continued on next page...

Continued from previous page...

Atom-1	Atom-2	Interatomic distance (Å)	Clash overlap (Å)
1:D:120:LEU:O	1:D:182:PRO:HD2	2.05	0.55
1:D:22:THR:HG21	1:D:104:ILE:HD13	1.89	0.54
1:E:18:VAL:HB	1:E:70:PRO:HA	1.90	0.53
1:F:135:GLU:N	1:F:135:GLU:OE1	2.36	0.53
1:A:243:MET:HE3	1:A:244:PHE:CE1	2.44	0.53
1:D:60:ALA:O	1:D:63:GLN:HG2	2.07	0.53
1:F:185:LEU:HA	1:F:230:LEU:O	2.10	0.52
1:C:272:LYS:HE3	1:C:277:PHE:O	2.09	0.51
1:C:210:ALA:HA	1:C:215:THR:CG2	2.40	0.51
1:F:223:GLY:O	1:F:263:SER:OG	2.27	0.51
1:D:7:SER:OG	1:D:9:GLN:O	2.22	0.51
1:A:217:PHE:O	1:A:281:MET:HA	2.10	0.51
1:D:14:THR:OG1	1:D:15:GLN:N	2.44	0.50
1:F:22:THR:OG1	1:F:76:LEU:N	2.44	0.50
1:C:194:VAL:HG12	1:C:205:HIS:HB3	1.93	0.50
1:C:67:LYS:HE3	1:C:68:TYR:CZ	2.45	0.50
1:A:124:VAL:HG12	1:A:151:ALA:HA	1.93	0.50
1:C:46:VAL:HG11	1:C:68:TYR:CE2	2.47	0.50
1:A:39:GLU:C	1:A:40:GLN:HG2	2.33	0.50
1:C:333:LYS:NZ	1:E:333:LYS:O	2.45	0.50
1:E:20:LEU:HD11	1:E:48:LEU:HD11	1.94	0.50
1:B:13:LEU:HD11	1:B:243:MET:CE	2.42	0.49
1:E:193:ASN:ND2	1:E:333:LYS:OXT	2.37	0.49
1:D:118:SER:HB3	1:D:157:LEU:HD21	1.93	0.49
1:A:18:VAL:HB	1:A:70:PRO:HA	1.95	0.49
1:C:6:VAL:HG23	1:C:245:ASN:HB3	1.93	0.49
1:D:18:VAL:HB	1:D:70:PRO:HA	1.96	0.48
1:D:263:SER:HB3	1:D:266:GLN:CD	2.34	0.47
1:D:71:THR:HG1	1:D:72:TYR:HD1	1.63	0.47
1:A:176:LEU:HD12	1:A:177:PHE:H	1.80	0.47
1:F:78:ALA:HA	1:F:100:MET:HE1	1.96	0.47
1:F:225:PRO:HB3	1:F:292:TYR:O	2.14	0.47
1:C:210:ALA:HA	1:C:215:THR:HG23	1.97	0.47
1:C:121:SER:O	1:C:124:VAL:HG22	2.15	0.47
1:E:15:GLN:NE2	1:E:42:LYS:HG2	2.29	0.47
1:F:186:PHE:O	1:F:232:SER:N	2.47	0.46
1:C:20:LEU:HD12	1:C:20:LEU:HA	1.64	0.46
1:C:51:LYS:NZ	1:E:326:GLU:OE2	2.48	0.46
1:F:221:GLY:O	1:F:283:PHE:HB3	2.15	0.46
1:C:134:THR:OG1	1:C:137:LYS:HD2	2.15	0.46
1:F:321:VAL:O	1:F:325:LEU:N	2.34	0.46

Continued on next page...

Continued from previous page...

Atom-1	Atom-2	Interatomic distance (Å)	Clash overlap (Å)
1:C:18:VAL:HB	1:C:70:PRO:HA	1.98	0.46
1:D:20:LEU:HD11	1:D:48:LEU:HD11	1.98	0.46
1:E:225:PRO:HG2	1:E:289:ASP:HB2	1.98	0.46
1:D:135:GLU:OE1	1:D:135:GLU:N	2.39	0.45
1:D:190:ASP:OD1	1:D:191:ASN:N	2.48	0.45
1:C:135:GLU:HG2	1:C:251:PRO:O	2.17	0.45
1:C:36:VAL:HG13	1:C:41:ILE:HB	1.98	0.44
1:D:184:ASN:HB2	1:D:228:GLN:O	2.16	0.44
1:B:224:LYS:HZ2	1:C:216:ASP:CG	2.21	0.44
1:B:263:SER:O	1:B:267:VAL:HG23	2.16	0.44
1:C:120:LEU:O	1:C:182:PRO:HD2	2.17	0.44
1:E:219:VAL:HG22	1:E:283:PHE:HA	2.00	0.44
1:A:176:LEU:HD12	1:A:177:PHE:N	2.33	0.43
1:C:20:LEU:HD12	1:C:46:VAL:HB	1.98	0.43
1:E:100:MET:HE2	1:E:100:MET:HB3	1.91	0.43
1:E:109:TYR:OH	1:E:171:GLU:OE2	2.28	0.43
1:A:40:GLN:HB3	1:A:40:GLN:HE21	1.69	0.43
1:E:289:ASP:OD1	1:E:289:ASP:N	2.50	0.43
1:E:199:VAL:O	1:E:203:LEU:HD13	2.18	0.43
1:A:193:ASN:ND2	1:A:333:LYS:OXT	2.39	0.43
1:A:224:LYS:O	1:A:226:LEU:HD12	2.19	0.43
1:B:267:VAL:O	1:B:271:ILE:HG13	2.17	0.43
1:D:19:ILE:HD13	1:D:45:TRP:CE2	2.53	0.43
1:C:74:ILE:HG21	1:C:239:LEU:HD21	2.00	0.43
1:E:135:GLU:HG2	1:E:251:PRO:O	2.19	0.42
1:C:30:LYS:HD2	1:C:30:LYS:HA	1.87	0.42
1:E:176:LEU:HD12	1:E:177:PHE:N	2.34	0.42
1:B:212:GLN:O	1:B:212:GLN:HG3	2.19	0.42
1:C:36:VAL:HA	1:C:41:ILE:HG13	2.01	0.42
1:A:267:VAL:HG22	1:A:313:PHE:CZ	2.54	0.42
1:C:117:ILE:HD11	1:C:243:MET:HA	2.01	0.42
1:A:117:ILE:HD11	1:A:243:MET:HA	2.02	0.42
1:B:28:PHE:O	1:B:32:VAL:HG23	2.19	0.42
1:E:176:LEU:HD21	1:E:247:GLN:HA	2.02	0.42
1:D:227:ARG:NH1	1:D:292:TYR:O	2.50	0.41
1:E:109:TYR:CZ	1:E:173:LYS:HD3	2.56	0.41
1:E:315:GLN:O	1:E:319:GLU:HG3	2.20	0.41
1:C:246:TYR:CZ	1:C:248:SER:HB3	2.56	0.41
1:C:258:GLU:O	1:C:261:GLU:HG2	2.21	0.41
1:B:135:GLU:HG2	1:B:251:PRO:O	2.21	0.41
1:D:19:ILE:HD13	1:D:45:TRP:NE1	2.36	0.41

Continued on next page...

Continued from previous page...

Atom-1	Atom-2	Interatomic distance (Å)	Clash overlap (Å)
1:B:176:LEU:HD12	1:B:177:PHE:H	1.86	0.41
1:C:30:LYS:NZ	1:E:329:GLU:OE1	2.45	0.41
1:A:104:ILE:HA	1:A:104:ILE:HD13	1.96	0.40
1:C:136:GLU:CD	1:C:136:GLU:H	2.25	0.40
1:D:117:ILE:HD11	1:D:243:MET:HA	2.02	0.40
1:F:45:TRP:HB3	1:F:47:PHE:CE1	2.53	0.40
1:E:55:LEU:HD13	1:E:104:ILE:HD11	2.03	0.40
1:C:109:TYR:HE1	1:C:168:ALA:HB2	1.86	0.40
1:F:219:VAL:O	1:F:283:PHE:HA	2.22	0.40

There are no symmetry-related clashes.

5.3 Torsion angles (i)

5.3.1 Protein backbone (i)

In the following table, the Percentiles column shows the percent Ramachandran outliers of the chain as a percentile score with respect to all X-ray entries followed by that with respect to entries of similar resolution.

The Analysed column shows the number of residues for which the backbone conformation was analysed, and the total number of residues.

Mol	Chain	Analysed	Favoured	Allowed	Outliers	Percentiles	
1	A	324/341 (95%)	317 (98%)	7 (2%)	0	100	100
1	B	319/341 (94%)	312 (98%)	7 (2%)	0	100	100
1	C	322/341 (94%)	316 (98%)	6 (2%)	0	100	100
1	D	318/341 (93%)	313 (98%)	5 (2%)	0	100	100
1	E	325/341 (95%)	316 (97%)	9 (3%)	0	100	100
1	F	289/341 (85%)	286 (99%)	3 (1%)	0	100	100
All	All	1897/2046 (93%)	1860 (98%)	37 (2%)	0	100	100

There are no Ramachandran outliers to report.

5.3.2 Protein sidechains (i)

In the following table, the Percentiles column shows the percent sidechain outliers of the chain as a percentile score with respect to all X-ray entries followed by that with respect to entries of similar resolution.

The Analysed column shows the number of residues for which the sidechain conformation was analysed, and the total number of residues.

Mol	Chain	Analysed	Rotameric	Outliers	Percentiles	
1	A	263/298 (88%)	260 (99%)	3 (1%)	73	83
1	B	259/298 (87%)	255 (98%)	4 (2%)	65	77
1	C	268/298 (90%)	261 (97%)	7 (3%)	46	61
1	D	221/298 (74%)	214 (97%)	7 (3%)	39	53
1	E	258/298 (87%)	254 (98%)	4 (2%)	62	77
1	F	153/298 (51%)	149 (97%)	4 (3%)	46	61
All	All	1422/1788 (80%)	1393 (98%)	29 (2%)	55	70

All (29) residues with a non-rotameric sidechain are listed below:

Mol	Chain	Res	Type
1	A	28	PHE
1	A	40	GLN
1	A	194	VAL
1	B	28	PHE
1	B	63	GLN
1	B	124	VAL
1	B	212	GLN
1	C	28	PHE
1	C	41	ILE
1	C	80	VAL
1	C	137	LYS
1	C	289	ASP
1	C	291	GLN
1	C	333	LYS
1	D	14	THR
1	D	28	PHE
1	D	50	SER
1	D	194	VAL
1	D	222	SER
1	D	263	SER
1	D	305	ASN
1	E	16	ASP
1	E	28	PHE
1	E	62	LYS
1	E	66	GLU
1	F	122	THR
1	F	174	SER

Continued on next page...

Continued from previous page...

Mol	Chain	Res	Type
1	F	253	MET
1	F	305	ASN

Some sidechains can be flipped to improve hydrogen bonding and reduce clashes. All (4) such sidechains are listed below:

Mol	Chain	Res	Type
1	A	40	GLN
1	B	212	GLN
1	C	247	GLN
1	D	305	ASN

5.3.3 RNA (i)

There are no RNA molecules in this entry.

5.4 Non-standard residues in protein, DNA, RNA chains (i)

There are no non-standard protein/DNA/RNA residues in this entry.

5.5 Carbohydrates (i)

There are no carbohydrates in this entry.

5.6 Ligand geometry (i)

Of 6 ligands modelled in this entry, 2 are monoatomic - leaving 4 for Mogul analysis.

In the following table, the Counts columns list the number of bonds (or angles) for which Mogul statistics could be retrieved, the number of bonds (or angles) that are observed in the model and the number of bonds (or angles) that are defined in the Chemical Component Dictionary. The Link column lists molecule types, if any, to which the group is linked. The Z score for a bond length (or angle) is the number of standard deviations the observed value is removed from the expected value. A bond length (or angle) with $|Z| > 2$ is considered an outlier worth inspection. RMSZ is the root-mean-square of all Z scores of the bond lengths (or angles).

Mol	Type	Chain	Res	Link	Bond lengths			Bond angles		
					Counts	RMSZ	$\#\|Z\| > 2$	Counts	RMSZ	$\#\|Z\| > 2$
2	SO4	A	401	-	4,4,4	0.14	0	6,6,6	0.06	0
2	SO4	B	401	-	4,4,4	0.15	0	6,6,6	0.12	0
2	SO4	E	401	-	4,4,4	0.13	0	6,6,6	0.15	0

Mol	Type	Chain	Res	Link	Bond lengths			Bond angles		
					Counts	RMSZ	#|Z| > 2	Counts	RMSZ	#|Z| > 2
2	SO4	C	401	-	4,4,4	0.13	0	6,6,6	0.14	0

There are no bond length outliers.

There are no bond angle outliers.

There are no chirality outliers.

There are no torsion outliers.

There are no ring outliers.

No monomer is involved in short contacts.

5.7 Other polymers ⓘ

There are no such residues in this entry.

5.8 Polymer linkage issues ⓘ

There are no chain breaks in this entry.

6 Fit of model and data (i)

6.1 Protein, DNA and RNA chains (i)

In the following table, the column labelled '#RSRZ> 2' contains the number (and percentage) of RSRZ outliers, followed by percent RSRZ outliers for the chain as percentile scores relative to all X-ray entries and entries of similar resolution. The OWAB column contains the minimum, median, 95th percentile and maximum values of the occupancy-weighted average B-factor per residue. The column labelled 'Q< 0.9' lists the number of (and percentage) of residues with an average occupancy less than 0.9.

Mol	Chain	Analysed	<RSRZ>	#RSRZ>2			OWAB(Å^2)	Q<0.9
1	A	328/341 (96%)	0.06	0	100	100	32, 58, 96, 122	0
1	B	322/341 (94%)	0.28	10 (3%)	49	56	34, 53, 92, 130	0
1	C	326/341 (95%)	-0.01	1 (0%)	94	96	36, 59, 93, 137	0
1	D	322/341 (94%)	0.51	28 (8%)	10	12	53, 87, 124, 151	0
1	E	329/341 (96%)	0.18	9 (2%)	54	61	37, 61, 108, 125	0
1	F	299/341 (87%)	1.08	62 (20%)	1	0	50, 107, 138, 161	0
All	All	1926/2046 (94%)	0.34	110 (5%)	23	28	32, 68, 122, 161	0

All (110) RSRZ outliers are listed below:

Mol	Chain	Res	Type	RSRZ
1	F	36	VAL	10.0
1	F	18	VAL	6.7
1	F	316	ALA	6.5
1	F	246	TYR	5.2
1	F	265	GLY	5.1
1	F	283	PHE	4.8
1	B	217	PHE	4.6
1	D	226	LEU	4.4
1	E	313	PHE	4.4
1	F	208	TYR	4.4
1	F	268	ALA	4.4
1	D	18	VAL	4.3
1	F	256	VAL	4.2
1	F	221	GLY	4.1
1	F	17	ASP	4.0
1	F	264	ILE	3.9
1	B	280	ASN	3.9
1	D	131	TYR	3.8
1	F	68	TYR	3.8

Continued on next page...

Continued from previous page...

Mol	Chain	Res	Type	RSRZ
1	F	67	LYS	3.7
1	F	205	HIS	3.6
1	F	43	GLY	3.6
1	D	265	GLY	3.6
1	D	220	PHE	3.5
1	F	271	ILE	3.4
1	F	269	GLN	3.4
1	F	233	HIS	3.4
1	D	292	TYR	3.3
1	D	283	PHE	3.3
1	D	223	GLY	3.3
1	F	328	TYR	3.2
1	E	217	PHE	3.2
1	F	223	GLY	3.2
1	F	16	ASP	3.2
1	F	229	PHE	3.1
1	F	324	PHE	3.1
1	D	281	MET	3.1
1	B	283	PHE	3.1
1	E	81	GLY	3.1
1	F	80	VAL	3.0
1	F	41	ILE	3.0
1	F	220	PHE	3.0
1	D	268	ALA	3.0
1	F	210	ALA	3.0
1	E	226	LEU	3.0
1	D	288	ALA	3.0
1	F	284	ASP	2.9
1	F	310	TYR	2.9
1	D	221	GLY	2.9
1	F	321	VAL	2.9
1	C	87	MET	2.9
1	F	323	TRP	2.9
1	F	34	HIS	2.8
1	F	40	GLN	2.8
1	B	215	THR	2.8
1	D	255	CYS	2.8
1	F	244	PHE	2.8
1	F	267	VAL	2.8
1	F	172	GLY	2.7
1	F	204	MET	2.7
1	D	88	ASN	2.7

Continued on next page...

Continued from previous page...

Mol	Chain	Res	Type	RSRZ
1	F	254	LEU	2.7
1	D	129	ILE	2.6
1	F	308	PHE	2.6
1	D	6	VAL	2.6
1	F	37	LYS	2.6
1	F	222	SER	2.6
1	F	217	PHE	2.5
1	B	274	ALA	2.5
1	F	302	LEU	2.5
1	D	65	PHE	2.5
1	F	188	PRO	2.5
1	F	207	CYS	2.5
1	D	245	ASN	2.5
1	F	48	LEU	2.5
1	E	86	ASN	2.4
1	F	200	LEU	2.4
1	D	70	PRO	2.4
1	E	277	PHE	2.4
1	F	50	SER	2.4
1	E	271	ILE	2.4
1	F	66	GLU	2.4
1	B	89	PHE	2.3
1	E	268	ALA	2.3
1	D	200	LEU	2.3
1	D	219	VAL	2.3
1	B	268	ALA	2.3
1	D	270	THR	2.3
1	B	277	PHE	2.3
1	F	70	PRO	2.3
1	F	192	PHE	2.3
1	F	88	ASN	2.2
1	E	275	PHE	2.2
1	F	311	THR	2.2
1	F	282	VAL	2.2
1	B	214	GLY	2.2
1	F	251	PRO	2.2
1	D	174	SER	2.1
1	D	243	MET	2.1
1	D	111	PHE	2.1
1	F	174	SER	2.1
1	F	51	LYS	2.1
1	F	45	TRP	2.1

Continued on next page...

Continued from previous page...

Mol	Chain	Res	Type	RSRZ
1	F	131	TYR	2.1
1	D	132	PRO	2.0
1	F	248	SER	2.0
1	B	275	PHE	2.0
1	F	19	ILE	2.0
1	D	89	PHE	2.0
1	D	109	TYR	2.0

6.2 Non-standard residues in protein, DNA, RNA chains (i)

There are no non-standard protein/DNA/RNA residues in this entry.

6.3 Carbohydrates (i)

There are no carbohydrates in this entry.

6.4 Ligands (i)

In the following table, the Atoms column lists the number of modelled atoms in the group and the number defined in the chemical component dictionary. The B-factors column lists the minimum, median, 95th percentile and maximum values of B factors of atoms in the group. The column labelled 'Q< 0.9' lists the number of atoms with occupancy less than 0.9.

Mol	Type	Chain	Res	Atoms	RSCC	RSR	B-factors(Å^2)	Q<0.9
3	CL	E	402	1/1	0.26	1.07	193,193,193,193	0
3	CL	A	402	1/1	0.69	0.66	169,169,169,169	0
2	SO4	B	401	5/5	0.78	0.31	155,155,157,157	0
2	SO4	E	401	5/5	0.81	0.35	160,160,160,163	0
2	SO4	A	401	5/5	0.85	0.19	150,150,151,152	0
2	SO4	C	401	5/5	0.89	0.24	118,121,123,126	0

6.5 Other polymers (i)

There are no such residues in this entry.
