# Supplementary material for: Naegleria fowleri: Protein structures to facilitate drug discovery for the deadly, pathogenic free-living amoeba
Source: PLoS One. 2021 Mar 24;16(3):e0241738. doi: 10.1371/journal.pone.0241738 (PMC7990177; doi:10.1371/journal.pone.0241738)
Supplement: S1 File — (ZIP) [file pone.0241738.s002.zip › 6aqz_full_validation.pdf]

# Full wwPDB X-ray Structure Validation Report (i)

May 15, 2020 – 04:28 am BST

| | | |
|---|---|---|
| PDB ID | : | 6AQZ |
| Title | : | Crystal structure of a gdp-l-fucose synthetase from Naegleria fowleri bound to NADP |
| Authors | : | Seattle Structural Genomics Center for Infectious Disease; Seattle Structural Genomics Center for Infectious Disease (SSGCID) |
| Deposited on | : | 2017-08-21 |
| Resolution | : | 2.40 Å(reported) |

This is a Full wwPDB X-ray Structure Validation Report for a publicly released PDB entry.

We welcome your comments at *validation@mail.wwpdb.org*
A user guide is available at
https://www.wwpdb.org/validation/2017/XrayValidationReportHelp
with specific help available everywhere you see the (i) symbol.

The following versions of software and data (see references (i)) were used in the production of this report:

| | | |
|---|---|---|
| MolProbity | : | 4.02b-467 |
| Mogul | : | 1.8.5 (274361), CSD as541be (2020) |
| Xtriage (Phenix) | : | 1.13 |
| EDS | : | 2.11 |
| buster-report | : | 1.1.7 (2018) |
| Percentile statistics | : | 20191225.v01 (using entries in the PDB archive December 25th 2019) |
| Refmac | : | 5.8.0158 |
| CCP4 | : | 7.0.044 (Gargrove) |
| Ideal geometry (proteins) | : | Engh & Huber (2001) |
| Ideal geometry (DNA, RNA) | : | Parkinson et al. (1996) |
| Validation Pipeline (wwPDB-VP) | : | 2.11 |

# 1 Overall quality at a glance (i)

The following experimental techniques were used to determine the structure:
*X-RAY DIFFRACTION*

The reported resolution of this entry is 2.40 Å.

Percentile scores (ranging between 0-100) for global validation metrics of the entry are shown in the following graphic. The table shows the number of entries on which the scores are based.

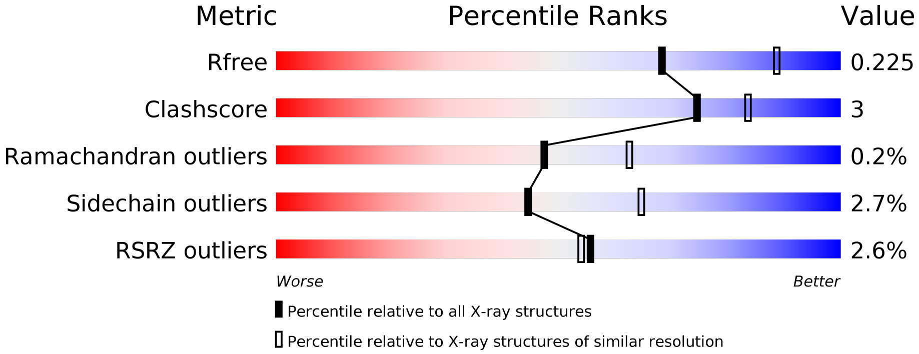


| Metric | Whole archive (#Entries) | Similar resolution (#Entries, resolution range(Å)) |
|---|---|---|
| $R_{free}$ | 130704 | 3907 (2.40-2.40) |
| Clashscore | 141614 | 4398 (2.40-2.40) |
| Ramachandran outliers | 138981 | 4318 (2.40-2.40) |
| Sidechain outliers | 138945 | 4319 (2.40-2.40) |
| RSRZ outliers | 127900 | 3811 (2.40-2.40) |

The table below summarises the geometric issues observed across the polymeric chains and their fit to the electron density. The red, orange, yellow and green segments on the lower bar indicate the fraction of residues that contain outliers for >=3, 2, 1 and 0 types of geometric quality criteria respectively. A grey segment represents the fraction of residues that are not modelled. The numeric value for each fraction is indicated below the corresponding segment, with a dot representing fractions <=5% The upper red bar (where present) indicates the fraction of residues that have poor fit to the electron density. The numeric value is given above the bar.

| Mol | Chain | Length | Quality of chain |
|---|---|---|---|
| 1 | A | 341 | 90% 8% • |
| 1 | B | 341 | % / 87% 7% 5% |
| 1 | C | 341 | 88% 10% • |
| 1 | D | 341 | 5% / 82% 13% 5% |
| 1 | E | 341 | 8% / 81% 9% • 8% |
| 1 | F | 341 | 88% 6% 6% |

# 2 Entry composition (i)

There are 4 unique types of molecules in this entry. The entry contains 15349 atoms, of which 0 are hydrogens and 0 are deuteriums.

In the tables below, the ZeroOcc column contains the number of atoms modelled with zero occupancy, the AltConf column contains the number of residues with at least one atom in alternate conformation and the Trace column contains the number of residues modelled with at most 2 atoms.

- Molecule 1 is a protein called gdp-l-fucose synthetase.

| Mol | Chain | Residues | Atoms | | | | | ZeroOcc | AltConf | Trace |
|---|---|---|---|---|---|---|---|---|---|---|
| 1 | A | 334 | Total | C | N | O | S | 0 | 1 | 0 |
| | | | 2610 | 1675 | 432 | 485 | 18 | | | |
| 1 | B | 323 | Total | C | N | O | S | 0 | 0 | 0 |
| | | | 2488 | 1594 | 414 | 464 | 16 | | | |
| 1 | C | 334 | Total | C | N | O | S | 0 | 1 | 0 |
| | | | 2605 | 1665 | 432 | 489 | 19 | | | |
| 1 | D | 324 | Total | C | N | O | S | 0 | 0 | 0 |
| | | | 2398 | 1526 | 406 | 450 | 16 | | | |
| 1 | E | 313 | Total | C | N | O | S | 0 | 0 | 0 |
| | | | 2246 | 1436 | 377 | 418 | 15 | | | |
| 1 | F | 321 | Total | C | N | O | S | 0 | 0 | 0 |
| | | | 2508 | 1612 | 414 | 466 | 16 | | | |

- Molecule 2 is NADP NICOTINAMIDE-ADENINE-DINUCLEOTIDE PHOSPHATE (three-letter code: NAP) (formula: $C_{21}H_{28}N_7O_{17}P_3$).

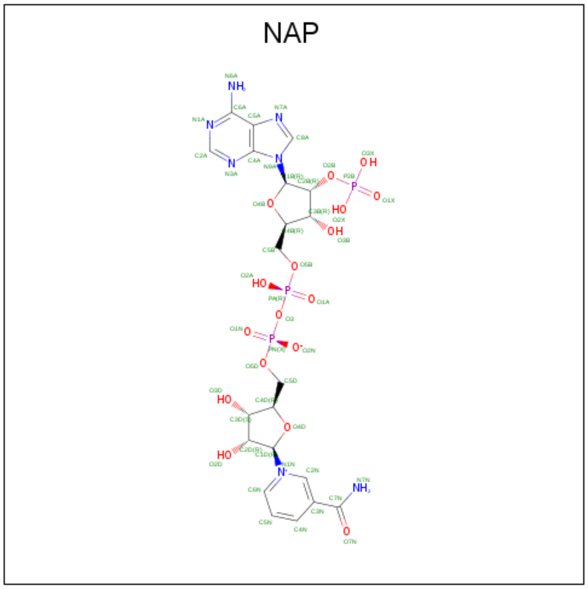

| Mol | Chain | Residues | Atoms | | | | | ZeroOcc | AltConf |
|---|---|---|---|---|---|---|---|---|---|
| 2 | A | 1 | Total 48 | C 21 | N 7 | O 17 | P 3 | 0 | 0 |
| 2 | B | 1 | Total 48 | C 21 | N 7 | O 17 | P 3 | 0 | 0 |
| 2 | C | 1 | Total 48 | C 21 | N 7 | O 17 | P 3 | 0 | 0 |
| 2 | E | 1 | Total 48 | C 21 | N 7 | O 17 | P 3 | 0 | 0 |
| 2 | F | 1 | Total 48 | C 21 | N 7 | O 17 | P 3 | 0 | 0 |

- Molecule 3 is 1,2-ETHANEDIOL (three-letter code: EDO) (formula: $C_2H_6O_2$).

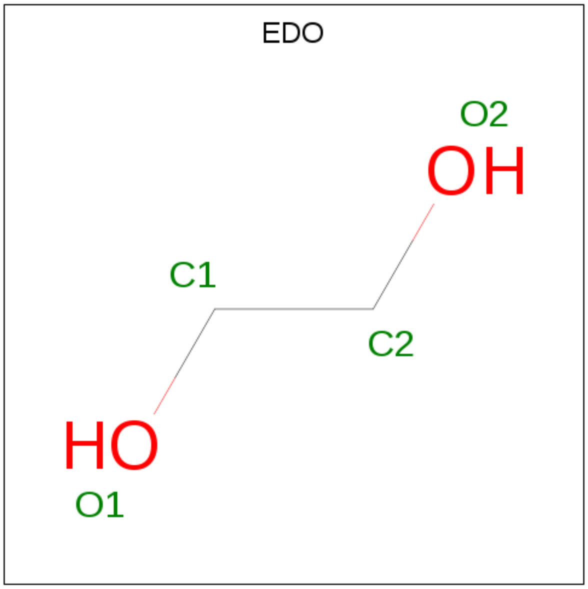


| Mol | Chain | Residues | Atoms | | | ZeroOcc | AltConf |
|---|---|---|---|---|---|---|---|
| 3 | B | 1 | Total 4 | C 2 | O 2 | 0 | 0 |

- Molecule 4 is water.

| Mol | Chain | Residues | Atoms | | ZeroOcc | AltConf |
|---|---|---|---|---|---|---|
| 4 | A | 70 | Total 70 | O 70 | 0 | 0 |
| 4 | B | 64 | Total 64 | O 64 | 0 | 0 |
| 4 | C | 51 | Total 51 | O 51 | 0 | 0 |
| 4 | D | 11 | Total 11 | O 11 | 0 | 0 |

*Continued on next page...*

*Continued from previous page...*

| Mol | Chain | Residues | Atoms | ZeroOcc | AltConf |
|---|---|---|---|---|---|
| 4 | E | 6 | Total O<br>6 6 | 0 | 0 |
| 4 | F | 48 | Total O<br>48 48 | 0 | 0 |

# 3 Residue-property plots

These plots are drawn for all protein, RNA and DNA chains in the entry. The first graphic for a chain summarises the proportions of the various outlier classes displayed in the second graphic. The second graphic shows the sequence view annotated by issues in geometry and electron density. Residues are color-coded according to the number of geometric quality criteria for which they contain at least one outlier: green = 0, yellow = 1, orange = 2 and red = 3 or more. A red dot above a residue indicates a poor fit to the electron density (RSRZ > 2). Stretches of 2 or more consecutive residues without any outlier are shown as a green connector. Residues present in the sample, but not in the model, are shown in grey.

- Molecule 1: gdp-l-fucose synthetase

Chain A:

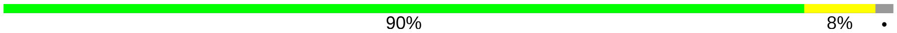


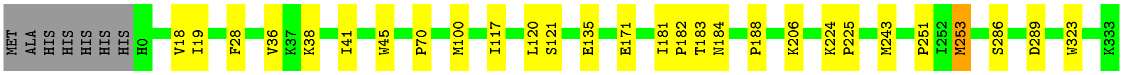


- Molecule 1: gdp-l-fucose synthetase

Chain B:

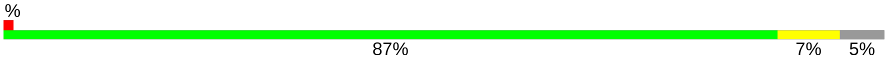


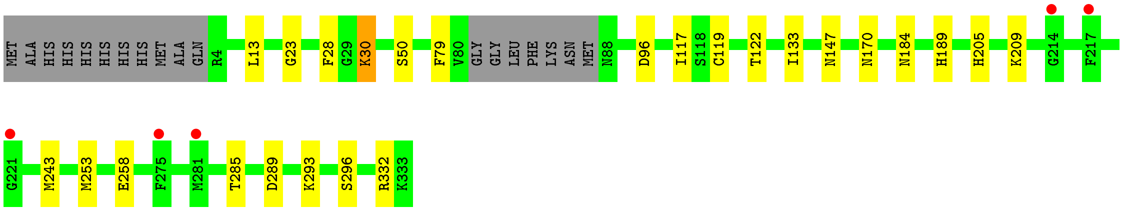


- Molecule 1: gdp-l-fucose synthetase

Chain C:

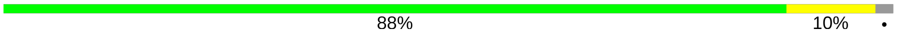


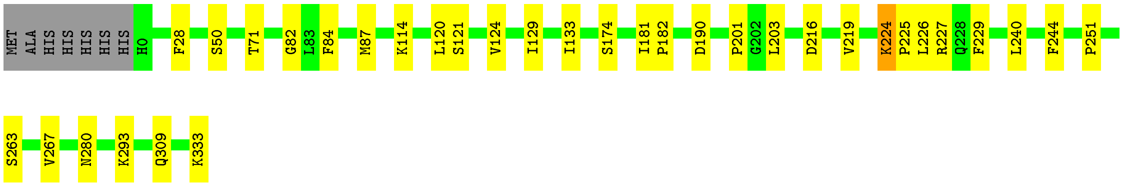


- Molecule 1: gdp-l-fucose synthetase

Chain D:

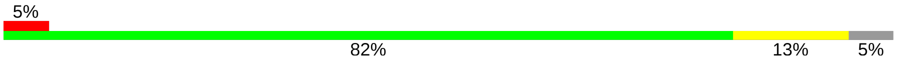


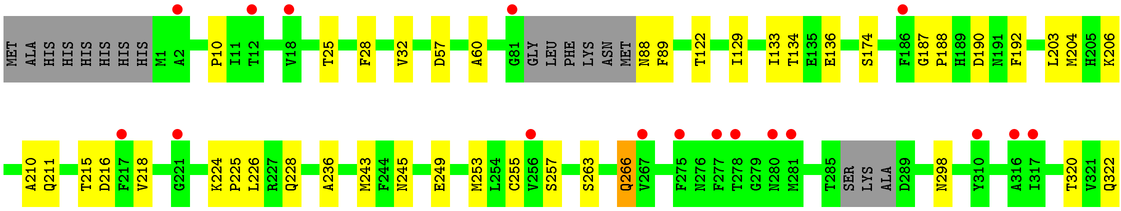

W323 Y328 K333

• Molecule 1: gdp-l-fucose synthetase

Chain E: 8% 81% 9% • 8%

MET ALA HIS HIS HIS HIS HIS HIS MET ALA GLN ARG E5 V6 S7 D8 Q9 D16 I19 G26 L27 F28 V36 I41 D54 L55 R56 Q63 P64 Y72 V73 I74 H75 L76 V80 I117 S118 C119 I129 E130 Y131 P132 E135 G172 I181 A196 V199 L200 L203 M204 H205 D216 F217 F220 G221 S222 G223 K224 P225 L226 R227 M243 F244 M253 L254 C255 V256 E259 S263 I264 G265 Q266 V267 A268 Q269 T270 T278 GLY ASN MET VAL PHE ASP THR SER LYS ALA ASP GLY GLN TYR LYS LYS T295 N298 A299 Q315 Y328 K333

• Molecule 1: gdp-l-fucose synthetase

Chain F: 88% 6% 6%

MET ALA HIS HIS HIS HIS HIS HIS MET ALA GLN ARG E5 V6 D17 V18 I19 L20 G26 L27 F28 V46 L55 P70 T71 V80 GLY GLY LEU PHE LYS ASN MET ASN F89 I104 C119 L120 S121 V124 Y131 R155 D158 K272 F277 S286 Q291 K333

# 4 Data and refinement statistics

| Property | Value | Source |
|---|---|---|
| Space group | P 1 21 1 | Depositor |
| Cell constants<br>a, b, c, $\alpha$, $\beta$, $\gamma$ | 97.37Å 104.03Å 120.17Å<br>90.00° 108.67° 90.00° | Depositor |
| Resolution (Å) | 46.12 – 2.40<br>48.49 – 2.40 | Depositor<br>EDS |
| % Data completeness<br>(in resolution range) | 98.8 (46.12-2.40)<br>98.9 (48.49-2.40) | Depositor<br>EDS |
| $R_{merge}$ | 0.05 | Depositor |
| $R_{sym}$ | (Not available) | Depositor |
| $< I/\sigma(I) >$ [1] | 2.19 (at 2.39Å) | Xtriage |
| Refinement program | PHENIX (1.12_2829: ???) | Depositor |
| R, $R_{free}$ | 0.178 , 0.224<br>0.178 , 0.225 | Depositor<br>DCC |
| $R_{free}$ test set | 2041 reflections (2.32%) | wwPDB-VP |
| Wilson B-factor (Å$^2$) | 53.4 | Xtriage |
| Anisotropy | 0.305 | Xtriage |
| Bulk solvent $k_{sol}$(e/Å$^3$), $B_{sol}$(Å$^2$) | 0.30 , 48.0 | EDS |
| L-test for twinning[2] | $< \|L\| > = 0.50$, $< L^2 > = 0.33$ | Xtriage |
| Estimated twinning fraction | No twinning to report. | Xtriage |
| $F_o$,$F_c$ correlation | 0.96 | EDS |
| Total number of atoms | 15349 | wwPDB-VP |
| Average B, all atoms (Å$^2$) | 67.0 | wwPDB-VP |

Xtriage's analysis on translational NCS is as follows: *The largest off-origin peak in the Patterson function is 3.26% of the height of the origin peak. No significant pseudotranslation is detected.*

[1]Intensities estimated from amplitudes.

[2]Theoretical values of $< |L| >$, $< L^2 >$ for acentric reflections are 0.5, 0.333 respectively for untwinned datasets, and 0.375, 0.2 for perfectly twinned datasets.

# 5 Model quality (i)

## 5.1 Standard geometry (i)

Bond lengths and bond angles in the following residue types are not validated in this section: NAP, EDO

The Z score for a bond length (or angle) is the number of standard deviations the observed value is removed from the expected value. A bond length (or angle) with $|Z| > 5$ is considered an outlier worth inspection. RMSZ is the root-mean-square of all Z scores of the bond lengths (or angles).

| Mol | Chain | Bond lengths | | Bond angles | |
|---|---|---|---|---|---|
| | | RMSZ | #\|Z\| >5 | RMSZ | #\|Z\| >5 |
| 1 | A | 0.46 | 0/2681 | 0.60 | 2/3639 (0.1%) |
| 1 | B | 0.45 | 1/2552 (0.0%) | 0.57 | 0/3468 |
| 1 | C | 0.44 | 0/2675 | 0.57 | 0/3631 |
| 1 | D | 0.36 | 0/2460 | 0.54 | 0/3350 |
| 1 | E | 0.37 | 1/2306 (0.0%) | 0.52 | 0/3155 |
| 1 | F | 0.44 | 1/2573 (0.0%) | 0.58 | 0/3492 |
| All | All | 0.42 | 3/15247 (0.0%) | 0.56 | 2/20735 (0.0%) |

All (3) bond length outliers are listed below:

| Mol | Chain | Res | Type | Atoms | Z | Observed(Å) | Ideal(Å) |
|---|---|---|---|---|---|---|---|
| 1 | E | 119 | CYS | CB-SG | -5.85 | 1.72 | 1.81 |
| 1 | F | 119 | CYS | CB-SG | -5.66 | 1.72 | 1.81 |
| 1 | B | 119 | CYS | CB-SG | -5.50 | 1.72 | 1.81 |

All (2) bond angle outliers are listed below:

| Mol | Chain | Res | Type | Atoms | Z | Observed(°) | Ideal(°) |
|---|---|---|---|---|---|---|---|
| 1 | A | 253 | MET | CA-CB-CG | -6.26 | 102.66 | 113.30 |
| 1 | A | 100 | MET | CG-SD-CE | 5.55 | 109.08 | 100.20 |

There are no chirality outliers.

There are no planarity outliers.

## 5.2 Too-close contacts (i)

In the following table, the Non-H and H(model) columns list the number of non-hydrogen atoms and hydrogen atoms in the chain respectively. The H(added) column lists the number of hydrogen atoms added and optimized by MolProbity. The Clashes column lists the number of clashes within

the asymmetric unit, whereas Symm-Clashes lists symmetry related clashes.

| Mol | Chain | Non-H | H(model) | H(added) | Clashes | Symm-Clashes |
|---|---|---|---|---|---|---|
| 1 | A | 2610 | 0 | 2468 | 13 | 0 |
| 1 | B | 2488 | 0 | 2311 | 10 | 0 |
| 1 | C | 2605 | 0 | 2444 | 18 | 0 |
| 1 | D | 2398 | 0 | 2110 | 22 | 0 |
| 1 | E | 2246 | 0 | 1911 | 17 | 0 |
| 1 | F | 2508 | 0 | 2385 | 12 | 0 |
| 2 | A | 48 | 0 | 25 | 2 | 0 |
| 2 | B | 48 | 0 | 23 | 2 | 0 |
| 2 | C | 48 | 0 | 25 | 3 | 0 |
| 2 | E | 48 | 0 | 24 | 2 | 0 |
| 2 | F | 48 | 0 | 23 | 2 | 0 |
| 3 | B | 4 | 0 | 6 | 1 | 0 |
| 4 | A | 70 | 0 | 0 | 0 | 0 |
| 4 | B | 64 | 0 | 0 | 0 | 0 |
| 4 | C | 51 | 0 | 0 | 1 | 0 |
| 4 | D | 11 | 0 | 0 | 0 | 0 |
| 4 | E | 6 | 0 | 0 | 0 | 0 |
| 4 | F | 48 | 0 | 0 | 1 | 0 |
| All | All | 15349 | 0 | 13755 | 91 | 0 |

The all-atom clashscore is defined as the number of clashes found per 1000 atoms (including hydrogen atoms). The all-atom clashscore for this structure is 3.

All (91) close contacts within the same asymmetric unit are listed below, sorted by their clash magnitude.

| Atom-1 | Atom-2 | Interatomic distance (Å) | Clash overlap (Å) |
|---|---|---|---|
| 1:D:263:SER:N | 1:D:266:GLN:OE1 | 2.05 | 0.89 |
| 1:D:206:LYS:NZ | 1:D:218:VAL:O | 2.16 | 0.79 |
| 1:C:226:LEU:O | 1:C:227:ARG:NH1 | 2.18 | 0.75 |
| 1:A:181:ILE:HB | 1:A:253:MET:HG2 | 1.69 | 0.74 |
| 1:F:17:ASP:HB2 | 1:F:71:THR:OG1 | 1.87 | 0.74 |
| 1:E:328:TYR:O | 1:E:333:LYS:NZ | 2.23 | 0.70 |
| 1:A:18:VAL:HB | 1:A:70:PRO:HA | 1.74 | 0.69 |
| 1:F:131:TYR:OH | 1:F:291:GLN:NE2 | 2.27 | 0.67 |
| 1:D:10:PRO:HG3 | 1:D:245:ASN:HD21 | 1.62 | 0.63 |
| 1:B:258:GLU:OE1 | 1:B:293:LYS:NZ | 2.31 | 0.62 |
| 1:E:54:ASP:OD1 | 1:E:56:ARG:HG2 | 2.00 | 0.61 |
| 1:E:225:PRO:O | 1:E:264:ILE:N | 2.35 | 0.59 |
| 1:C:309:GLN:NE2 | 4:C:502:HOH:O | 2.30 | 0.59 |
| 1:E:7:SER:OG | 1:E:9:GLN:O | 2.22 | 0.58 |

Continued on next page...

*Continued from previous page...*

| Atom-1 | Atom-2 | Interatomic distance (Å) | Clash overlap (Å) |
|---|---|---|---|
| 1:C:71:THR:HB | 1:C:114:LYS:HE3 | 1.85 | 0.58 |
| 1:D:328:TYR:O | 1:D:333:LYS:NZ | 2.34 | 0.58 |
| 1:D:88:ASN:OD1 | 1:D:89:PHE:N | 2.35 | 0.57 |
| 1:F:55:LEU:HD13 | 1:F:104:ILE:HD11 | 1.85 | 0.57 |
| 1:A:36:VAL:HA | 1:A:41[A]:ILE:HD13 | 1.85 | 0.57 |
| 2:E:401:NAP:N7N | 2:E:401:NAP:O2N | 2.39 | 0.55 |
| 1:D:210:ALA:HA | 1:D:215:THR:HG23 | 1.89 | 0.55 |
| 1:D:192:PHE:HE2 | 1:D:204:MET:HE2 | 1.73 | 0.54 |
| 1:F:18:VAL:HB | 1:F:70:PRO:HA | 1.89 | 0.54 |
| 1:F:120:LEU:O | 2:F:401:NAP:H6N | 2.08 | 0.53 |
| 1:B:30:LYS:HG2 | 1:B:189:HIS:CD2 | 2.45 | 0.52 |
| 1:C:121:SER:O | 1:C:124:VAL:HG22 | 2.10 | 0.51 |
| 1:D:226:LEU:HA | 1:D:263:SER:HA | 1.93 | 0.51 |
| 1:E:135:GLU:HG3 | 1:E:253:MET:HG3 | 1.92 | 0.51 |
| 1:E:266:GLN:O | 1:E:270:THR:OG1 | 2.28 | 0.51 |
| 1:C:203:LEU:HD22 | 1:C:219:VAL:HG12 | 1.93 | 0.50 |
| 1:B:205:HIS:O | 1:B:209:LYS:HG3 | 2.12 | 0.49 |
| 1:F:20:LEU:HD12 | 1:F:46:VAL:HB | 1.95 | 0.49 |
| 1:E:226:LEU:HA | 1:E:263:SER:HA | 1.95 | 0.48 |
| 1:B:117:ILE:HD11 | 1:B:243:MET:HA | 1.95 | 0.48 |
| 1:B:293:LYS:O | 1:F:286:SER:HB3 | 2.13 | 0.48 |
| 1:B:50:SER:OG | 2:B:401:NAP:O2X | 2.22 | 0.48 |
| 1:C:229:PHE:CE2 | 1:C:267:VAL:HG21 | 2.49 | 0.47 |
| 1:C:190:ASP:OD2 | 1:C:201:PRO:HG3 | 2.15 | 0.47 |
| 1:C:84:PHE:O | 1:C:87:MET:HG2 | 2.15 | 0.47 |
| 1:B:133:ILE:HG22 | 1:B:253:MET:HE3 | 1.97 | 0.47 |
| 1:D:57:ASP:HB3 | 1:D:60:ALA:HB3 | 1.97 | 0.47 |
| 1:C:263:SER:O | 1:C:267:VAL:HG23 | 2.15 | 0.46 |
| 1:A:117:ILE:HD11 | 1:A:243:MET:HA | 1.96 | 0.46 |
| 1:D:215:THR:OG1 | 1:D:216:ASP:N | 2.48 | 0.46 |
| 1:C:120:LEU:O | 2:C:401:NAP:H6N | 2.15 | 0.46 |
| 1:F:120:LEU:HD11 | 1:F:158:ASP:HB2 | 1.98 | 0.46 |
| 1:A:120:LEU:O | 2:A:401:NAP:H6N | 2.15 | 0.46 |
| 1:C:50:SER:HG | 2:C:401:NAP:P2B | 2.39 | 0.46 |
| 1:D:187:GLY:O | 1:D:190:ASP:HB2 | 2.15 | 0.46 |
| 1:E:199:VAL:O | 1:E:203:LEU:HD13 | 2.16 | 0.46 |
| 1:A:224:LYS:N | 1:A:225:PRO:CD | 2.79 | 0.46 |
| 1:E:181:ILE:HB | 1:E:253:MET:HG2 | 1.99 | 0.45 |
| 1:F:272:LYS:HE2 | 1:F:277:PHE:O | 2.17 | 0.45 |
| 1:C:82:GLY:HA3 | 1:C:87:MET:HG3 | 1.97 | 0.45 |
| 1:D:188:PRO:HB3 | 1:D:323:TRP:CG | 2.52 | 0.45 |

*Continued on next page...*

*Continued from previous page...*

| Atom-1 | Atom-2 | Interatomic distance (Å) | Clash overlap (Å) |
|---|---|---|---|
| 1:A:188:PRO:HB3 | 1:A:323:TRP:CG | 2.52 | 0.45 |
| 1:C:240:LEU:O | 1:C:244:PHE:HD1 | 1.99 | 0.44 |
| 1:D:210:ALA:HA | 1:D:215:THR:CG2 | 2.47 | 0.44 |
| 1:D:192:PHE:CE2 | 1:D:204:MET:HE2 | 2.52 | 0.44 |
| 1:C:129:ILE:HD13 | 1:C:133:ILE:HG12 | 2.00 | 0.43 |
| 1:D:255:CYS:SG | 1:D:298:ASN:HB3 | 2.58 | 0.43 |
| 1:B:79:PHE:CD2 | 1:B:96:ASP:HB3 | 2.53 | 0.43 |
| 1:D:32:VAL:HA | 1:D:236:ALA:HB1 | 1.99 | 0.43 |
| 1:F:155:ARG:HD3 | 4:F:501:HOH:O | 2.18 | 0.43 |
| 1:B:23:GLY:HA2 | 2:B:401:NAP:H1B | 2.01 | 0.43 |
| 1:D:129:ILE:HD13 | 1:D:133:ILE:HD12 | 2.01 | 0.43 |
| 1:D:203:LEU:HD23 | 1:D:203:LEU:HA | 1.89 | 0.43 |
| 1:C:216:ASP:OD1 | 1:C:280:ASN:HB2 | 2.18 | 0.42 |
| 1:E:205:HIS:CE1 | 1:E:333:LYS:HB2 | 2.54 | 0.42 |
| 1:E:74:ILE:HG22 | 1:E:76:LEU:HG | 2.02 | 0.42 |
| 1:F:26:GLY:HA3 | 2:F:401:NAP:O5B | 2.20 | 0.42 |
| 1:E:36:VAL:HA | 1:E:41:ILE:HD13 | 2.00 | 0.42 |
| 1:C:182:PRO:HG2 | 2:C:401:NAP:C6N | 2.49 | 0.42 |
| 1:E:117:ILE:HD11 | 1:E:243:MET:HA | 2.01 | 0.42 |
| 1:E:223:GLY:C | 1:E:225:PRO:HD3 | 2.39 | 0.42 |
| 1:E:63:GLN:HB2 | 1:E:64:PRO:HD3 | 2.01 | 0.42 |
| 1:A:135:GLU:HG3 | 1:A:253:MET:HG3 | 2.02 | 0.41 |
| 1:A:38:LYS:O | 1:D:136:GLU:HG3 | 2.21 | 0.41 |
| 1:E:26:GLY:HA3 | 2:E:401:NAP:O5B | 2.20 | 0.41 |
| 1:E:19:ILE:HG13 | 1:E:72:TYR:HB2 | 2.01 | 0.41 |
| 1:A:183:THR:HG23 | 1:A:184:ASN:N | 2.36 | 0.41 |
| 1:D:204:MET:HE1 | 1:D:320:THR:HG22 | 2.02 | 0.41 |
| 1:F:121:SER:O | 1:F:124:VAL:HG22 | 2.21 | 0.41 |
| 1:D:228:GLN:HE21 | 1:D:257:SER:C | 2.23 | 0.41 |
| 1:D:225:PRO:O | 1:D:263:SER:HA | 2.21 | 0.41 |
| 1:A:19:ILE:HG21 | 1:A:45:TRP:CZ3 | 2.56 | 0.40 |
| 1:B:289:ASP:OD1 | 3:B:402:EDO:H22 | 2.21 | 0.40 |
| 1:C:224:LYS:N | 1:C:225:PRO:CD | 2.85 | 0.40 |
| 1:A:182:PRO:HG2 | 2:A:401:NAP:C6N | 2.51 | 0.40 |
| 1:C:181:ILE:HD11 | 1:C:251:PRO:HB2 | 2.04 | 0.40 |
| 1:A:135:GLU:HG2 | 1:A:251:PRO:O | 2.21 | 0.40 |

There are no symmetry-related clashes.

## 5.3 Torsion angles (i)

### 5.3.1 Protein backbone (i)

In the following table, the Percentiles column shows the percent Ramachandran outliers of the chain as a percentile score with respect to all X-ray entries followed by that with respect to entries of similar resolution.

The Analysed column shows the number of residues for which the backbone conformation was analysed, and the total number of residues.

| Mol | Chain | Analysed | Favoured | Allowed | Outliers | Percentiles | |
|---|---|---|---|---|---|---|---|
| 1 | A | 333/341 (98%) | 327 (98%) | 6 (2%) | 0 | 100 | 100 |
| 1 | B | 319/341 (94%) | 313 (98%) | 5 (2%) | 1 (0%) | 41 | 55 |
| 1 | C | 333/341 (98%) | 329 (99%) | 3 (1%) | 1 (0%) | 41 | 55 |
| 1 | D | 318/341 (93%) | 312 (98%) | 5 (2%) | 1 (0%) | 41 | 55 |
| 1 | E | 309/341 (91%) | 296 (96%) | 13 (4%) | 0 | 100 | 100 |
| 1 | F | 317/341 (93%) | 312 (98%) | 5 (2%) | 0 | 100 | 100 |
| All | All | 1929/2046 (94%) | 1889 (98%) | 37 (2%) | 3 (0%) | 47 | 62 |

All (3) Ramachandran outliers are listed below:

| Mol | Chain | Res | Type |
|---|---|---|---|
| 1 | B | 184 | ASN |
| 1 | D | 224 | LYS |
| 1 | C | 224 | LYS |

### 5.3.2 Protein sidechains (i)

In the following table, the Percentiles column shows the percent sidechain outliers of the chain as a percentile score with respect to all X-ray entries followed by that with respect to entries of similar resolution.

The Analysed column shows the number of residues for which the sidechain conformation was analysed, and the total number of residues.

| Mol | Chain | Analysed | Rotameric | Outliers | Percentiles | |
|---|---|---|---|---|---|---|
| 1 | A | 273/298 (92%) | 267 (98%) | 6 (2%) | 52 | 71 |
| 1 | B | 255/298 (86%) | 246 (96%) | 9 (4%) | 36 | 55 |
| 1 | C | 272/298 (91%) | 268 (98%) | 4 (2%) | 65 | 80 |
| 1 | D | 227/298 (76%) | 216 (95%) | 11 (5%) | 25 | 41 |

*Continued on next page...*

*Continued from previous page...*

| Mol | Chain | Analysed | Rotameric | Outliers | Percentiles | |
|---|---|---|---|---|---|---|
| 1 | E | 200/298 (67%) | 191 (96%) | 9 (4%) | 27 | 44 |
| 1 | F | 265/298 (89%) | 264 (100%) | 1 (0%) | 91 | 96 |
| All | All | 1492/1788 (83%) | 1452 (97%) | 40 (3%) | 44 | 65 |

All (40) residues with a non-rotameric sidechain are listed below:

| Mol | Chain | Res | Type |
|---|---|---|---|
| 1 | A | 28 | PHE |
| 1 | A | 121 | SER |
| 1 | A | 171 | GLU |
| 1 | A | 206 | LYS |
| 1 | A | 286 | SER |
| 1 | A | 289 | ASP |
| 1 | B | 13 | LEU |
| 1 | B | 28 | PHE |
| 1 | B | 30 | LYS |
| 1 | B | 122 | THR |
| 1 | B | 147 | ASN |
| 1 | B | 170 | ASN |
| 1 | B | 285 | THR |
| 1 | B | 296 | SER |
| 1 | B | 332 | ARG |
| 1 | C | 28 | PHE |
| 1 | C | 174 | SER |
| 1 | C | 293 | LYS |
| 1 | C | 333 | LYS |
| 1 | D | 25 | THR |
| 1 | D | 28 | PHE |
| 1 | D | 122 | THR |
| 1 | D | 134 | THR |
| 1 | D | 174 | SER |
| 1 | D | 211 | GLN |
| 1 | D | 243 | MET |
| 1 | D | 249 | GLU |
| 1 | D | 253 | MET |
| 1 | D | 266 | GLN |
| 1 | D | 322 | GLN |
| 1 | E | 7 | SER |
| 1 | E | 28 | PHE |
| 1 | E | 80 | VAL |
| 1 | E | 199 | VAL |

*Continued on next page...*

*Continued from previous page...*

| Mol | Chain | Res | Type |
|---|---|---|---|
| 1 | E | 200 | LEU |
| 1 | E | 216 | ASP |
| 1 | E | 270 | THR |
| 1 | E | 315 | GLN |
| 1 | E | 333 | LYS |
| 1 | F | 28 | PHE |

Some sidechains can be flipped to improve hydrogen bonding and reduce clashes. All (7) such sidechains are listed below:

| Mol | Chain | Res | Type |
|---|---|---|---|
| 1 | B | 189 | HIS |
| 1 | B | 211 | GLN |
| 1 | C | 291 | GLN |
| 1 | D | 212 | GLN |
| 1 | D | 245 | ASN |
| 1 | F | 269 | GLN |
| 1 | F | 291 | GLN |

#### 5.3.3 RNA (i)

There are no RNA molecules in this entry.

### 5.4 Non-standard residues in protein, DNA, RNA chains (i)

There are no non-standard protein/DNA/RNA residues in this entry.

### 5.5 Carbohydrates (i)

There are no carbohydrates in this entry.

### 5.6 Ligand geometry (i)

6 ligands are modelled in this entry.

In the following table, the Counts columns list the number of bonds (or angles) for which Mogul statistics could be retrieved, the number of bonds (or angles) that are observed in the model and the number of bonds (or angles) that are defined in the Chemical Component Dictionary. The Link column lists molecule types, if any, to which the group is linked. The Z score for a bond length (or angle) is the number of standard deviations the observed value is removed from the

expected value. A bond length (or angle) with $|Z| > 2$ is considered an outlier worth inspection. RMSZ is the root-mean-square of all Z scores of the bond lengths (or angles).

| Mol | Type | Chain | Res | Link | Bond lengths | | | Bond angles | | |
|---|---|---|---|---|---|---|---|---|---|---|
| | | | | | Counts | RMSZ | $\#\|Z\| > 2$ | Counts | RMSZ | $\#\|Z\| > 2$ |
| 2 | NAP | E | 401 | - | 45,52,52 | 2.75 | 10 (22%) | 56,80,80 | 2.00 | 16 (28%) |
| 2 | NAP | C | 401 | - | 45,52,52 | 2.75 | 13 (28%) | 56,80,80 | 1.83 | 13 (23%) |
| 2 | NAP | B | 401 | 1 | 45,52,52 | 2.93 | 18 (40%) | 56,80,80 | 1.98 | 17 (30%) |
| 2 | NAP | F | 401 | - | 45,52,52 | 2.82 | 13 (28%) | 56,80,80 | 1.89 | 12 (21%) |
| 3 | EDO | B | 402 | - | 3,3,3 | 0.47 | 0 | 2,2,2 | 0.25 | 0 |
| 2 | NAP | A | 401 | - | 45,52,52 | 2.90 | 13 (28%) | 56,80,80 | 1.88 | 14 (25%) |

In the following table, the Chirals column lists the number of chiral outliers, the number of chiral centers analysed, the number of these observed in the model and the number defined in the Chemical Component Dictionary. Similar counts are reported in the Torsion and Rings columns. '-' means no outliers of that kind were identified.

| Mol | Type | Chain | Res | Link | Chirals | Torsions | Rings |
|---|---|---|---|---|---|---|---|
| 2 | NAP | E | 401 | - | - | 5/31/67/67 | 0/5/5/5 |
| 2 | NAP | C | 401 | - | - | 4/31/67/67 | 0/5/5/5 |
| 2 | NAP | B | 401 | 1 | - | 8/31/67/67 | 0/5/5/5 |
| 2 | NAP | F | 401 | - | - | 4/31/67/67 | 0/5/5/5 |
| 3 | EDO | B | 402 | - | - | 0/1/1/1 | - |
| 2 | NAP | A | 401 | - | - | 5/31/67/67 | 0/5/5/5 |

All (67) bond length outliers are listed below:

| Mol | Chain | Res | Type | Atoms | Z | Observed(Å) | Ideal(Å) |
|---|---|---|---|---|---|---|---|
| 2 | A | 401 | NAP | P2B-O2B | 14.11 | 1.86 | 1.59 |
| 2 | B | 401 | NAP | P2B-O2B | 13.88 | 1.85 | 1.59 |
| 2 | C | 401 | NAP | P2B-O2B | 13.79 | 1.85 | 1.59 |
| 2 | E | 401 | NAP | P2B-O2B | 12.81 | 1.83 | 1.59 |
| 2 | F | 401 | NAP | P2B-O2B | 11.46 | 1.81 | 1.59 |
| 2 | F | 401 | NAP | C2N-N1N | 6.86 | 1.43 | 1.35 |
| 2 | F | 401 | NAP | C4N-C3N | 6.34 | 1.50 | 1.39 |
| 2 | E | 401 | NAP | C4N-C3N | 6.32 | 1.50 | 1.39 |
| 2 | F | 401 | NAP | C5N-C4N | 5.80 | 1.51 | 1.38 |
| 2 | C | 401 | NAP | C4N-C3N | 5.65 | 1.49 | 1.39 |
| 2 | A | 401 | NAP | C4N-C3N | 5.40 | 1.48 | 1.39 |
| 2 | A | 401 | NAP | C5N-C4N | 5.22 | 1.49 | 1.38 |
| 2 | B | 401 | NAP | C4N-C3N | 5.22 | 1.48 | 1.39 |
| 2 | B | 401 | NAP | PN-O5D | 4.72 | 1.78 | 1.59 |

*Continued on next page...*

Continued from previous page...

| Mol | Chain | Res | Type | Atoms | Z | Observed(Å) | Ideal(Å) |
|---|---|---|---|---|---|---|---|
| 2 | C | 401 | NAP | C5N-C4N | 4.41 | 1.48 | 1.38 |
| 2 | E | 401 | NAP | C5N-C4N | 4.40 | 1.48 | 1.38 |
| 2 | E | 401 | NAP | PN-O5D | 4.34 | 1.76 | 1.59 |
| 2 | B | 401 | NAP | C3N-C7N | -4.34 | 1.44 | 1.50 |
| 2 | A | 401 | NAP | C2N-N1N | 4.26 | 1.40 | 1.35 |
| 2 | C | 401 | NAP | C2N-N1N | 4.13 | 1.40 | 1.35 |
| 2 | B | 401 | NAP | C2N-N1N | 4.04 | 1.39 | 1.35 |
| 2 | A | 401 | NAP | PN-O5D | 3.98 | 1.75 | 1.59 |
| 2 | E | 401 | NAP | C7N-N7N | 3.95 | 1.40 | 1.33 |
| 2 | A | 401 | NAP | C7N-N7N | 3.88 | 1.40 | 1.33 |
| 2 | B | 401 | NAP | C5N-C4N | 3.62 | 1.46 | 1.38 |
| 2 | F | 401 | NAP | C6N-N1N | 3.43 | 1.43 | 1.35 |
| 2 | F | 401 | NAP | C7N-N7N | 3.36 | 1.39 | 1.33 |
| 2 | F | 401 | NAP | O4D-C1D | 3.33 | 1.45 | 1.41 |
| 2 | F | 401 | NAP | C3D-C4D | 3.27 | 1.61 | 1.53 |
| 2 | E | 401 | NAP | O2D-C2D | -3.26 | 1.35 | 1.43 |
| 2 | B | 401 | NAP | C7N-N7N | 3.11 | 1.38 | 1.33 |
| 2 | C | 401 | NAP | O4B-C1B | 3.05 | 1.45 | 1.41 |
| 2 | A | 401 | NAP | C3D-C4D | 3.04 | 1.60 | 1.53 |
| 2 | B | 401 | NAP | C2A-N1A | 2.99 | 1.39 | 1.33 |
| 2 | F | 401 | NAP | PN-O5D | 2.97 | 1.71 | 1.59 |
| 2 | E | 401 | NAP | C2D-C1D | 2.83 | 1.58 | 1.53 |
| 2 | A | 401 | NAP | C6N-N1N | 2.81 | 1.42 | 1.35 |
| 2 | E | 401 | NAP | C2N-N1N | 2.81 | 1.38 | 1.35 |
| 2 | A | 401 | NAP | O2B-C2B | -2.80 | 1.33 | 1.44 |
| 2 | C | 401 | NAP | PN-O5D | 2.80 | 1.70 | 1.59 |
| 2 | F | 401 | NAP | O3D-C3D | -2.75 | 1.36 | 1.43 |
| 2 | E | 401 | NAP | O2B-C2B | -2.73 | 1.34 | 1.44 |
| 2 | B | 401 | NAP | O2B-C2B | -2.69 | 1.34 | 1.44 |
| 2 | F | 401 | NAP | O2D-C2D | -2.69 | 1.36 | 1.43 |
| 2 | B | 401 | NAP | C3D-C4D | 2.68 | 1.59 | 1.53 |
| 2 | C | 401 | NAP | O2D-C2D | -2.57 | 1.36 | 1.43 |
| 2 | F | 401 | NAP | O2B-C2B | -2.50 | 1.35 | 1.44 |
| 2 | C | 401 | NAP | O2B-C2B | -2.48 | 1.35 | 1.44 |
| 2 | B | 401 | NAP | O4B-C1B | 2.40 | 1.44 | 1.41 |
| 2 | B | 401 | NAP | C3B-C4B | 2.37 | 1.59 | 1.53 |
| 2 | B | 401 | NAP | C6A-N6A | 2.36 | 1.42 | 1.34 |
| 2 | C | 401 | NAP | C6N-C5N | -2.28 | 1.33 | 1.38 |
| 2 | B | 401 | NAP | C2D-C1D | 2.27 | 1.57 | 1.53 |
| 2 | B | 401 | NAP | PA-O5B | 2.24 | 1.68 | 1.59 |
| 2 | A | 401 | NAP | C3B-C4B | 2.23 | 1.58 | 1.53 |
| 2 | C | 401 | NAP | C6N-N1N | 2.18 | 1.40 | 1.35 |

Continued on next page...

*Continued from previous page...*

| Mol | Chain | Res | Type | Atoms | Z | Observed(Å) | Ideal(Å) |
|---|---|---|---|---|---|---|---|
| 2 | F | 401 | NAP | O5D-C5D | -2.17 | 1.36 | 1.44 |
| 2 | C | 401 | NAP | C3D-C4D | 2.17 | 1.58 | 1.53 |
| 2 | E | 401 | NAP | C2A-N1A | 2.16 | 1.37 | 1.33 |
| 2 | A | 401 | NAP | O2D-C2D | -2.16 | 1.37 | 1.43 |
| 2 | B | 401 | NAP | C4A-N3A | 2.15 | 1.38 | 1.35 |
| 2 | B | 401 | NAP | C6N-C5N | -2.12 | 1.33 | 1.38 |
| 2 | A | 401 | NAP | C6N-C5N | -2.12 | 1.33 | 1.38 |
| 2 | C | 401 | NAP | C7N-N7N | 2.12 | 1.37 | 1.33 |
| 2 | A | 401 | NAP | C2A-N3A | 2.10 | 1.35 | 1.32 |
| 2 | C | 401 | NAP | O5D-C5D | -2.04 | 1.36 | 1.44 |
| 2 | B | 401 | NAP | O2D-C2D | -2.01 | 1.38 | 1.43 |

All (72) bond angle outliers are listed below:

| Mol | Chain | Res | Type | Atoms | Z | Observed($^o$) | Ideal($^o$) |
|---|---|---|---|---|---|---|---|
| 2 | E | 401 | NAP | C5N-C4N-C3N | -6.70 | 112.42 | 120.34 |
| 2 | B | 401 | NAP | C5N-C4N-C3N | -6.64 | 112.48 | 120.34 |
| 2 | E | 401 | NAP | PN-O3-PA | -5.96 | 112.36 | 132.83 |
| 2 | A | 401 | NAP | C5N-C4N-C3N | -5.89 | 113.37 | 120.34 |
| 2 | B | 401 | NAP | PN-O3-PA | -5.80 | 112.92 | 132.83 |
| 2 | A | 401 | NAP | PN-O3-PA | -5.57 | 113.70 | 132.83 |
| 2 | F | 401 | NAP | PN-O3-PA | -5.32 | 114.56 | 132.83 |
| 2 | C | 401 | NAP | C5N-C4N-C3N | -5.24 | 114.14 | 120.34 |
| 2 | C | 401 | NAP | PN-O3-PA | -5.23 | 114.87 | 132.83 |
| 2 | F | 401 | NAP | C5N-C4N-C3N | -5.14 | 114.26 | 120.34 |
| 2 | B | 401 | NAP | C2N-C3N-C4N | 4.66 | 123.54 | 118.26 |
| 2 | C | 401 | NAP | O7N-C7N-N7N | -4.47 | 116.22 | 122.58 |
| 2 | A | 401 | NAP | C2N-C3N-C4N | 3.71 | 122.46 | 118.26 |
| 2 | F | 401 | NAP | O2B-P2B-O1X | -3.65 | 95.29 | 109.39 |
| 2 | F | 401 | NAP | O7N-C7N-N7N | -3.62 | 117.44 | 122.58 |
| 2 | E | 401 | NAP | C2N-C3N-C4N | 3.62 | 122.36 | 118.26 |
| 2 | F | 401 | NAP | O7N-C7N-C3N | 3.48 | 123.80 | 119.63 |
| 2 | C | 401 | NAP | O7N-C7N-C3N | 3.42 | 123.72 | 119.63 |
| 2 | E | 401 | NAP | O2B-P2B-O1X | -3.30 | 96.67 | 109.39 |
| 2 | F | 401 | NAP | C2N-C3N-C4N | 3.21 | 121.90 | 118.26 |
| 2 | A | 401 | NAP | O7N-C7N-N7N | -3.15 | 118.11 | 122.58 |
| 2 | E | 401 | NAP | O7N-C7N-C3N | 3.10 | 123.34 | 119.63 |
| 2 | B | 401 | NAP | C6N-N1N-C2N | -3.00 | 119.24 | 121.97 |
| 2 | C | 401 | NAP | O2B-P2B-O1X | -2.94 | 98.06 | 109.39 |
| 2 | B | 401 | NAP | O2B-P2B-O1X | -2.90 | 98.18 | 109.39 |
| 2 | E | 401 | NAP | C2A-N1A-C6A | -2.90 | 113.80 | 118.75 |
| 2 | E | 401 | NAP | PA-O5B-C5B | -2.75 | 105.53 | 121.68 |

*Continued on next page...*

*Continued from previous page...*

| Mol | Chain | Res | Type | Atoms | Z | Observed(°) | Ideal(°) |
|---|---|---|---|---|---|---|---|
| 2 | E | 401 | NAP | O7N-C7N-N7N | -2.72 | 118.71 | 122.58 |
| 2 | B | 401 | NAP | PA-O5B-C5B | -2.70 | 105.82 | 121.68 |
| 2 | A | 401 | NAP | C3B-C2B-C1B | -2.68 | 97.85 | 102.89 |
| 2 | C | 401 | NAP | C2N-C3N-C4N | 2.68 | 121.30 | 118.26 |
| 2 | E | 401 | NAP | O2N-PN-O1N | 2.62 | 125.19 | 112.24 |
| 2 | A | 401 | NAP | O7N-C7N-C3N | 2.60 | 122.75 | 119.63 |
| 2 | A | 401 | NAP | O2B-P2B-O1X | -2.60 | 99.37 | 109.39 |
| 2 | A | 401 | NAP | O3X-P2B-O2X | 2.58 | 117.49 | 107.64 |
| 2 | F | 401 | NAP | O3X-P2B-O2X | 2.57 | 117.48 | 107.64 |
| 2 | B | 401 | NAP | O7N-C7N-C3N | 2.55 | 122.69 | 119.63 |
| 2 | E | 401 | NAP | C6N-N1N-C2N | -2.54 | 119.66 | 121.97 |
| 2 | C | 401 | NAP | O3X-P2B-O2X | 2.54 | 117.35 | 107.64 |
| 2 | B | 401 | NAP | O3X-P2B-O2X | 2.53 | 117.32 | 107.64 |
| 2 | A | 401 | NAP | C6N-N1N-C2N | -2.52 | 119.68 | 121.97 |
| 2 | A | 401 | NAP | C2A-N1A-C6A | -2.52 | 114.45 | 118.75 |
| 2 | A | 401 | NAP | PA-O5B-C5B | -2.49 | 107.10 | 121.68 |
| 2 | B | 401 | NAP | C2A-N1A-C6A | -2.47 | 114.53 | 118.75 |
| 2 | B | 401 | NAP | O5D-PN-O1N | -2.47 | 99.44 | 109.07 |
| 2 | B | 401 | NAP | PN-O5D-C5D | -2.46 | 107.25 | 121.68 |
| 2 | C | 401 | NAP | PA-O5B-C5B | -2.42 | 107.48 | 121.68 |
| 2 | E | 401 | NAP | PN-O5D-C5D | -2.39 | 107.67 | 121.68 |
| 2 | E | 401 | NAP | O3X-P2B-O2X | 2.37 | 116.70 | 107.64 |
| 2 | C | 401 | NAP | O4B-C4B-C3B | 2.36 | 109.79 | 105.11 |
| 2 | E | 401 | NAP | C3B-C2B-C1B | -2.36 | 98.46 | 102.89 |
| 2 | B | 401 | NAP | O7N-C7N-N7N | -2.34 | 119.25 | 122.58 |
| 2 | C | 401 | NAP | O2N-PN-O1N | 2.32 | 123.71 | 112.24 |
| 2 | B | 401 | NAP | O2N-PN-O1N | 2.32 | 123.69 | 112.24 |
| 2 | F | 401 | NAP | O5D-PN-O1N | -2.29 | 100.12 | 109.07 |
| 2 | F | 401 | NAP | C2A-N1A-C6A | -2.25 | 114.91 | 118.75 |
| 2 | F | 401 | NAP | PA-O5B-C5B | -2.25 | 108.51 | 121.68 |
| 2 | F | 401 | NAP | O4B-C4B-C3B | 2.23 | 109.52 | 105.11 |
| 2 | B | 401 | NAP | O4B-C4B-C3B | 2.19 | 109.45 | 105.11 |
| 2 | E | 401 | NAP | O2N-PN-O5D | -2.19 | 97.59 | 107.75 |
| 2 | E | 401 | NAP | O4B-C4B-C3B | 2.18 | 109.42 | 105.11 |
| 2 | A | 401 | NAP | O2N-PN-O1N | 2.13 | 122.80 | 112.24 |
| 2 | C | 401 | NAP | C6N-N1N-C2N | -2.13 | 120.03 | 121.97 |
| 2 | B | 401 | NAP | O3X-P2B-O2B | -2.11 | 96.53 | 105.99 |
| 2 | A | 401 | NAP | O5D-PN-O1N | -2.09 | 100.89 | 109.07 |
| 2 | E | 401 | NAP | O5D-PN-O1N | -2.08 | 100.93 | 109.07 |
| 2 | F | 401 | NAP | C6N-N1N-C2N | -2.07 | 120.09 | 121.97 |
| 2 | C | 401 | NAP | C2A-N1A-C6A | -2.06 | 115.23 | 118.75 |
| 2 | A | 401 | NAP | O2N-PN-O5D | -2.03 | 98.33 | 107.75 |

*Continued on next page...*

*Continued from previous page...*

| Mol | Chain | Res | Type | Atoms | Z | Observed(°) | Ideal(°) |
|---|---|---|---|---|---|---|---|
| 2 | C | 401 | NAP | O5D-PN-O1N | -2.02 | 101.17 | 109.07 |
| 2 | B | 401 | NAP | C2N-N1N-C1D | 2.01 | 123.62 | 119.14 |
| 2 | B | 401 | NAP | C6N-C5N-C4N | 2.01 | 122.36 | 119.44 |

There are no chirality outliers.

All (26) torsion outliers are listed below:

| Mol | Chain | Res | Type | Atoms |
|---|---|---|---|---|
| 2 | B | 401 | NAP | C5D-O5D-PN-O3 |
| 2 | B | 401 | NAP | O4D-C4D-C5D-O5D |
| 2 | B | 401 | NAP | C3D-C4D-C5D-O5D |
| 2 | E | 401 | NAP | O4D-C4D-C5D-O5D |
| 2 | E | 401 | NAP | C3D-C4D-C5D-O5D |
| 2 | A | 401 | NAP | O4D-C4D-C5D-O5D |
| 2 | C | 401 | NAP | PN-O3-PA-O1A |
| 2 | F | 401 | NAP | PN-O3-PA-O1A |
| 2 | B | 401 | NAP | O4B-C4B-C5B-O5B |
| 2 | A | 401 | NAP | O4B-C4B-C5B-O5B |
| 2 | B | 401 | NAP | C5D-O5D-PN-O1N |
| 2 | B | 401 | NAP | C5D-O5D-PN-O2N |
| 2 | F | 401 | NAP | O4D-C4D-C5D-O5D |
| 2 | E | 401 | NAP | PN-O3-PA-O1A |
| 2 | B | 401 | NAP | PN-O3-PA-O2A |
| 2 | E | 401 | NAP | PN-O3-PA-O2A |
| 2 | B | 401 | NAP | PN-O3-PA-O1A |
| 2 | F | 401 | NAP | PN-O3-PA-O2A |
| 2 | A | 401 | NAP | PN-O3-PA-O1A |
| 2 | A | 401 | NAP | PN-O3-PA-O2A |
| 2 | C | 401 | NAP | O4D-C4D-C5D-O5D |
| 2 | A | 401 | NAP | C3D-C4D-C5D-O5D |
| 2 | C | 401 | NAP | O4B-C4B-C5B-O5B |
| 2 | C | 401 | NAP | PN-O3-PA-O2A |
| 2 | E | 401 | NAP | O4B-C4B-C5B-O5B |
| 2 | F | 401 | NAP | O4B-C4B-C5B-O5B |

There are no ring outliers.

6 monomers are involved in 12 short contacts:

| Mol | Chain | Res | Type | Clashes | Symm-Clashes |
|---|---|---|---|---|---|
| 2 | E | 401 | NAP | 2 | 0 |
| 2 | C | 401 | NAP | 3 | 0 |

*Continued on next page...*

*Continued from previous page...*

| Mol | Chain | Res | Type | Clashes | Symm-Clashes |
|---|---|---|---|---|---|
| 2 | B | 401 | NAP | 2 | 0 |
| 2 | F | 401 | NAP | 2 | 0 |
| 3 | B | 402 | EDO | 1 | 0 |
| 2 | A | 401 | NAP | 2 | 0 |

The following is a two-dimensional graphical depiction of Mogul quality analysis of bond lengths, bond angles, torsion angles, and ring geometry for all instances of the Ligand of Interest. In addition, ligands with molecular weight > 250 and outliers as shown on the validation Tables will also be included. For torsion angles, if less then 5% of the Mogul distribution of torsion angles is within 10 degrees of the torsion angle in question, then that torsion angle is considered an outlier. Any bond that is central to one or more torsion angles identified as an outlier by Mogul will be highlighted in the graph. For rings, the root-mean-square deviation (RMSD) between the ring in question and similar rings identified by Mogul is calculated over all ring torsion angles. If the average RMSD is greater than 60 degrees and the minimal RMSD between the ring in question and any Mogul-identified rings is also greater than 60 degrees, then that ring is considered an outlier. The outliers are highlighted in purple. The color gray indicates Mogul did not find sufficient equivalents in the CSD to analyse the geometry.

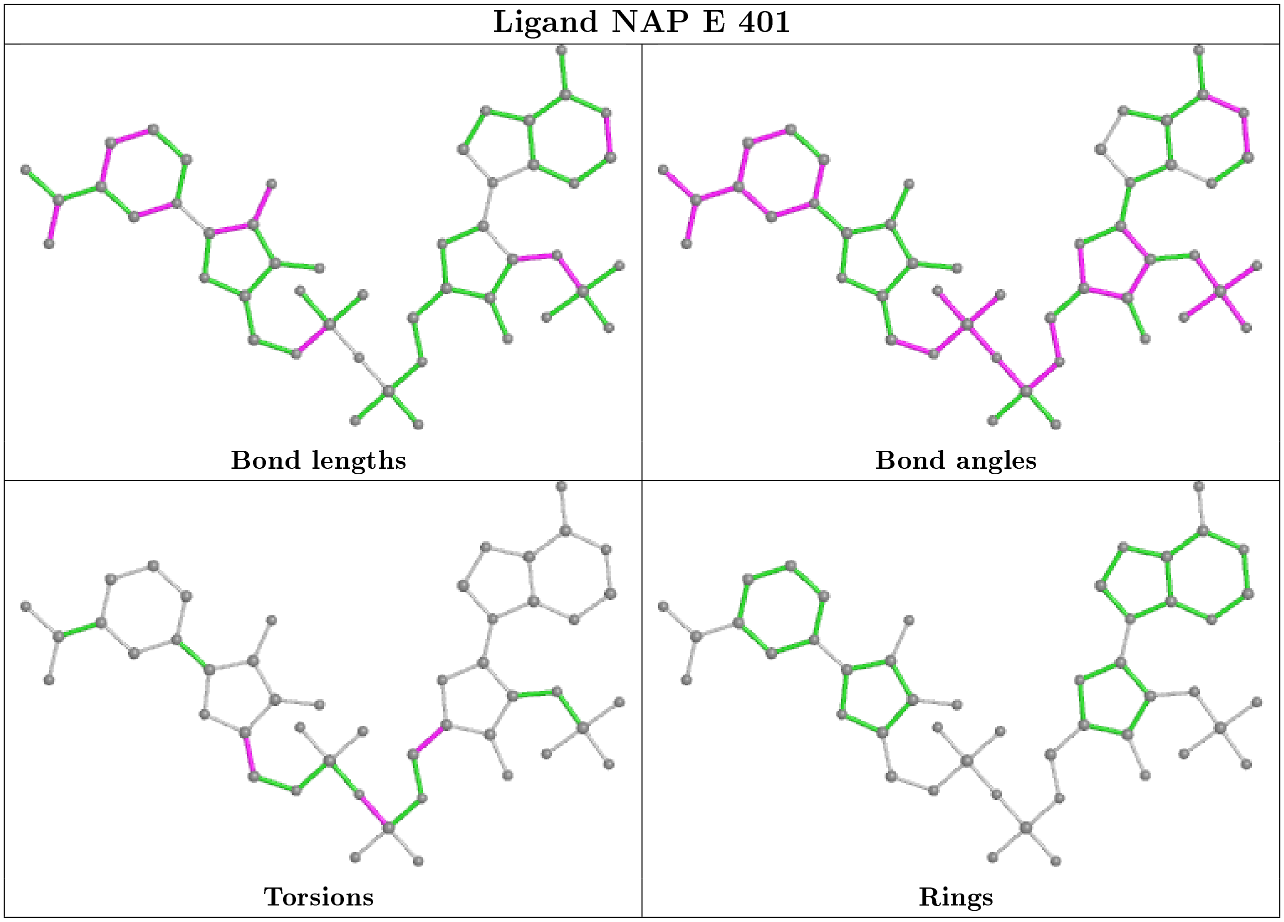

**Ligand NAP C 401**

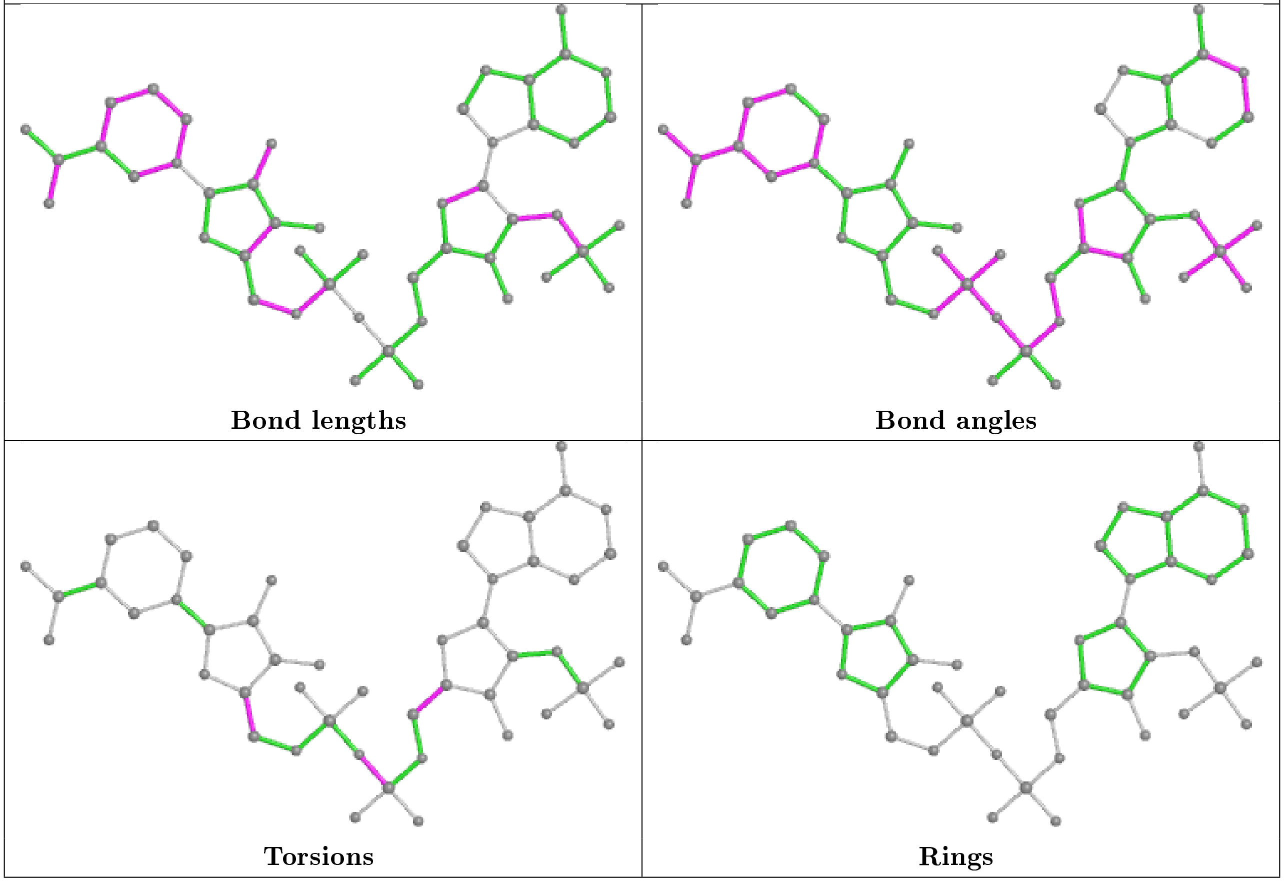

**Ligand NAP B 401**

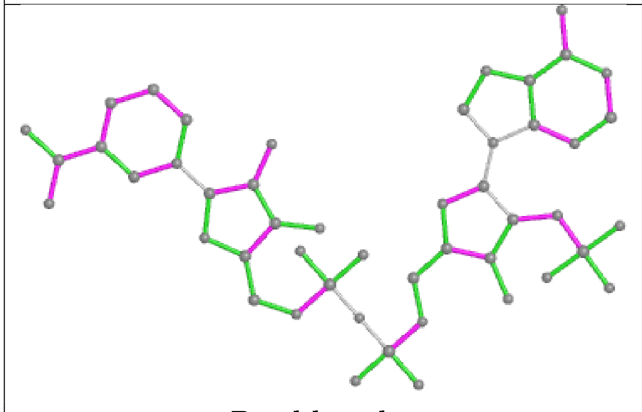

Bond lengths

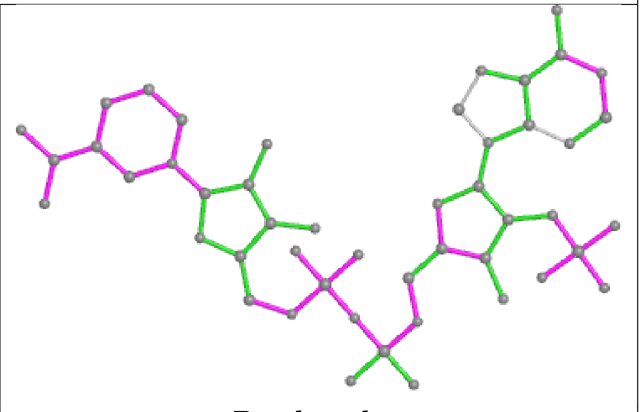

Bond angles

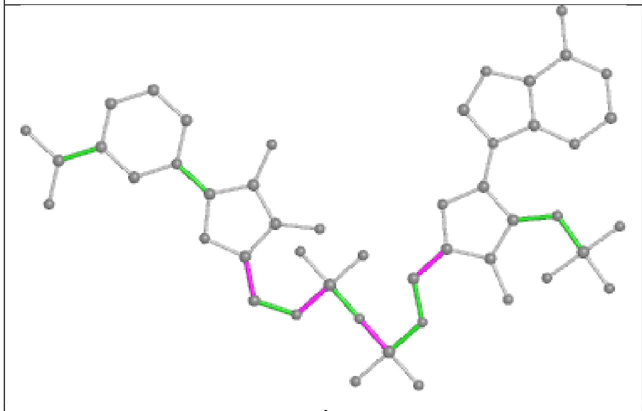

Torsions

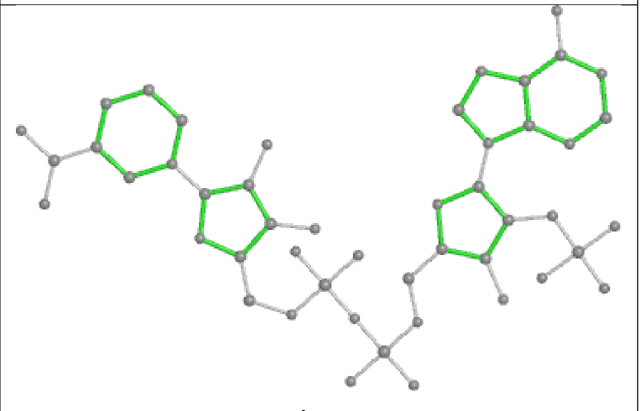

Rings

**Ligand NAP F 401**

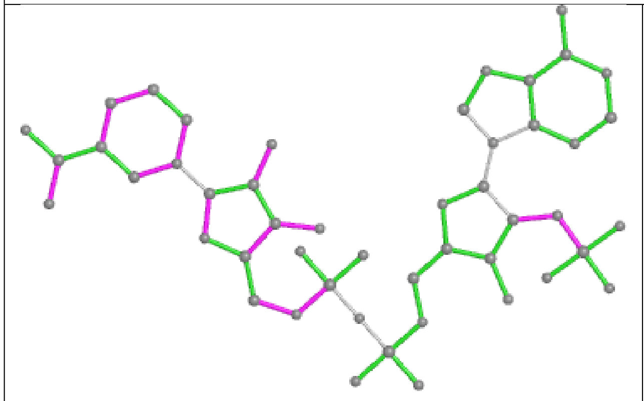

Bond lengths

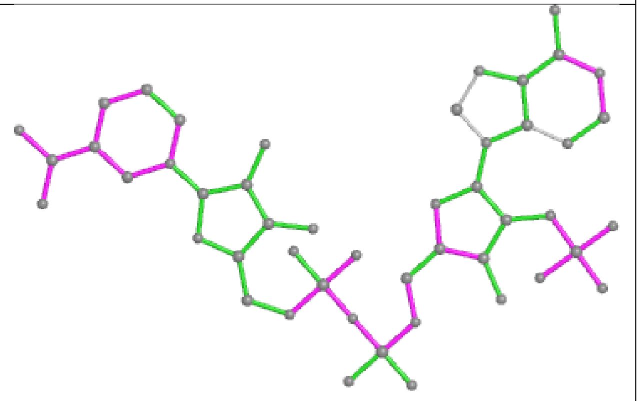

Bond angles

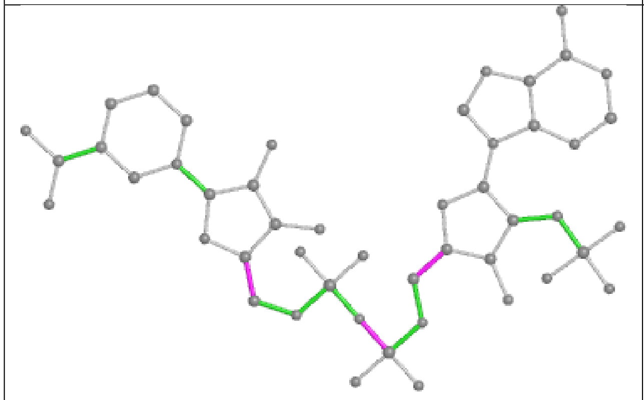

Torsions

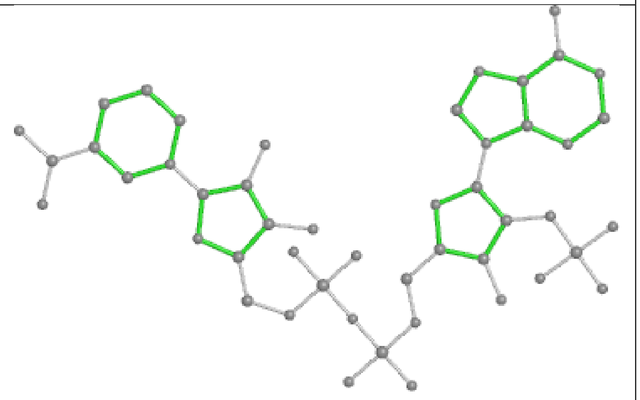

Rings

**Ligand NAP A 401**

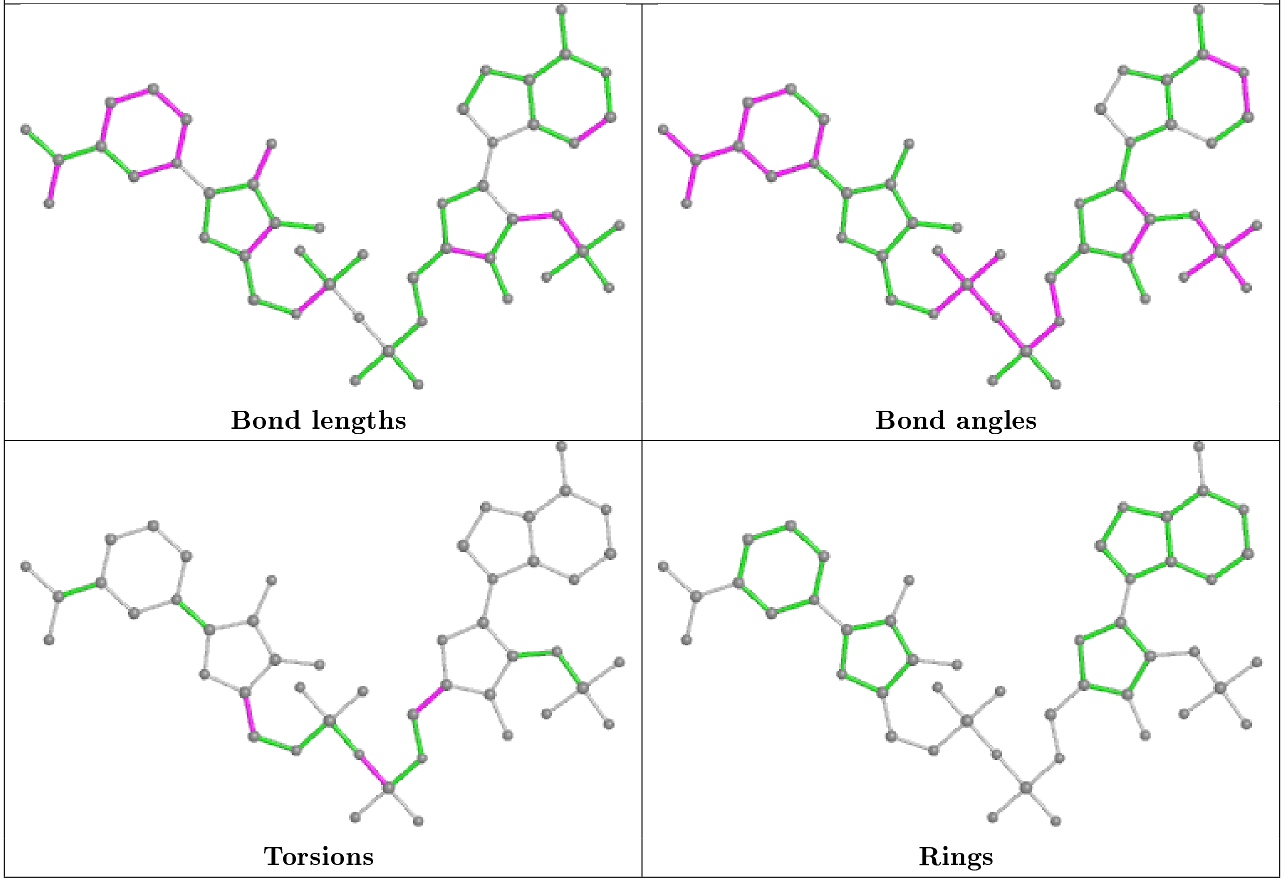


## 5.7 Other polymers (i)

There are no such residues in this entry.

## 5.8 Polymer linkage issues (i)

There are no chain breaks in this entry.

# 6 Fit of model and data

## 6.1 Protein, DNA and RNA chains

In the following table, the column labelled '#RSRZ> 2' contains the number (and percentage) of RSRZ outliers, followed by percent RSRZ outliers for the chain as percentile scores relative to all X-ray entries and entries of similar resolution. The OWAB column contains the minimum, median, $95^{th}$ percentile and maximum values of the occupancy-weighted average B-factor per residue. The column labelled 'Q< 0.9' lists the number of (and percentage) of residues with an average occupancy less than 0.9.

| Mol | Chain | Analysed | <RSRZ> | #RSRZ>2 | | | OWAB(Å$^2$) | Q<0.9 |
|---|---|---|---|---|---|---|---|---|
| 1 | A | 334/341 (97%) | -0.27 | 0 | 100 | 100 | 32, 56, 83, 105 | 0 |
| 1 | B | 323/341 (94%) | -0.09 | 5 (1%) | 73 | 72 | 34, 57, 104, 141 | 0 |
| 1 | C | 334/341 (97%) | -0.24 | 0 | 100 | 100 | 38, 55, 86, 112 | 0 |
| 1 | D | 324/341 (95%) | 0.22 | 17 (5%) | 27 | 26 | 46, 84, 112, 145 | 0 |
| 1 | E | 313/341 (91%) | 0.38 | 27 (8%) | 10 | 9 | 52, 90, 130, 170 | 0 |
| 1 | F | 321/341 (94%) | -0.31 | 1 (0%) | 94 | 93 | 36, 58, 85, 106 | 0 |
| All | All | 1949/2046 (95%) | -0.06 | 50 (2%) | 56 | 54 | 32, 65, 108, 170 | 0 |

All (50) RSRZ outliers are listed below:

| Mol | Chain | Res | Type | RSRZ |
|---|---|---|---|---|
| 1 | E | 225 | PRO | 6.1 |
| 1 | E | 226 | LEU | 5.9 |
| 1 | E | 220 | PHE | 4.9 |
| 1 | E | 269 | GLN | 4.8 |
| 1 | E | 265 | GLY | 3.7 |
| 1 | D | 275 | PHE | 3.7 |
| 1 | D | 278 | THR | 3.6 |
| 1 | D | 217 | PHE | 3.6 |
| 1 | E | 256 | VAL | 3.5 |
| 1 | D | 280 | ASN | 3.4 |
| 1 | F | 6 | VAL | 3.2 |
| 1 | E | 222 | SER | 3.1 |
| 1 | D | 221 | GLY | 3.1 |
| 1 | E | 270 | THR | 3.1 |
| 1 | E | 244 | PHE | 3.0 |
| 1 | E | 263 | SER | 3.0 |
| 1 | E | 221 | GLY | 3.0 |
| 1 | E | 267 | VAL | 2.9 |
| 1 | E | 255 | CYS | 2.8 |

*Continued on next page...*

*Continued from previous page...*

| Mol | Chain | Res | Type | RSRZ |
|---|---|---|---|---|
| 1 | B | 217 | PHE | 2.8 |
| 1 | D | 281 | MET | 2.8 |
| 1 | D | 316 | ALA | 2.7 |
| 1 | D | 18 | VAL | 2.7 |
| 1 | D | 2 | ALA | 2.6 |
| 1 | D | 81 | GLY | 2.6 |
| 1 | E | 299 | ALA | 2.5 |
| 1 | D | 277 | PHE | 2.5 |
| 1 | D | 12 | THR | 2.5 |
| 1 | E | 217 | PHE | 2.4 |
| 1 | B | 221 | GLY | 2.4 |
| 1 | D | 267 | VAL | 2.4 |
| 1 | E | 196 | ALA | 2.4 |
| 1 | D | 256 | VAL | 2.4 |
| 1 | D | 186 | PHE | 2.4 |
| 1 | D | 310 | TYR | 2.3 |
| 1 | B | 214 | GLY | 2.3 |
| 1 | E | 243 | MET | 2.2 |
| 1 | D | 317 | ILE | 2.2 |
| 1 | E | 129 | ILE | 2.2 |
| 1 | E | 259 | GLU | 2.2 |
| 1 | E | 227 | ARG | 2.2 |
| 1 | E | 16 | ASP | 2.2 |
| 1 | E | 268 | ALA | 2.2 |
| 1 | E | 72 | TYR | 2.2 |
| 1 | E | 298 | ASN | 2.2 |
| 1 | E | 132 | PRO | 2.2 |
| 1 | E | 172 | GLY | 2.2 |
| 1 | B | 275 | PHE | 2.1 |
| 1 | B | 281 | MET | 2.0 |
| 1 | E | 131 | TYR | 2.0 |

## 6.2 Non-standard residues in protein, DNA, RNA chains (i)

There are no non-standard protein/DNA/RNA residues in this entry.

## 6.3 Carbohydrates (i)

There are no carbohydrates in this entry.

## 6.4 Ligands (i)

In the following table, the Atoms column lists the number of modelled atoms in the group and the number defined in the chemical component dictionary. The B-factors column lists the minimum, median, $95^{th}$ percentile and maximum values of B factors of atoms in the group. The column labelled 'Q< 0.9' lists the number of atoms with occupancy less than 0.9.

| Mol | Type | Chain | Res | Atoms | RSCC | RSR | B-factors(Å$^2$) | Q<0.9 |
|---|---|---|---|---|---|---|---|---|
| 2 | NAP | B | 401 | 48/48 | 0.75 | 0.32 | 43,74,87,90 | 48 |
| 3 | EDO | B | 402 | 4/4 | 0.92 | 0.48 | 81,81,81,81 | 0 |
| 2 | NAP | E | 401 | 48/48 | 0.95 | 0.13 | 51,68,81,94 | 0 |
| 2 | NAP | F | 401 | 48/48 | 0.98 | 0.14 | 39,45,55,67 | 0 |
| 2 | NAP | C | 401 | 48/48 | 0.98 | 0.14 | 39,47,57,59 | 0 |
| 2 | NAP | A | 401 | 48/48 | 0.98 | 0.13 | 34,46,58,59 | 0 |

The following is a graphical depiction of the model fit to experimental electron density of all instances of the Ligand of Interest. In addition, ligands with molecular weight > 250 and outliers as shown on the geometry validation Tables will also be included. Each fit is shown from different orientation to approximate a three-dimensional view.

**Electron density around NAP B 401:**

2mF$_o$-DF$_c$ (at 0.7 rmsd) in gray
mF$_o$-DF$_c$ (at 3 rmsd) in purple (negative)
and green (positive)

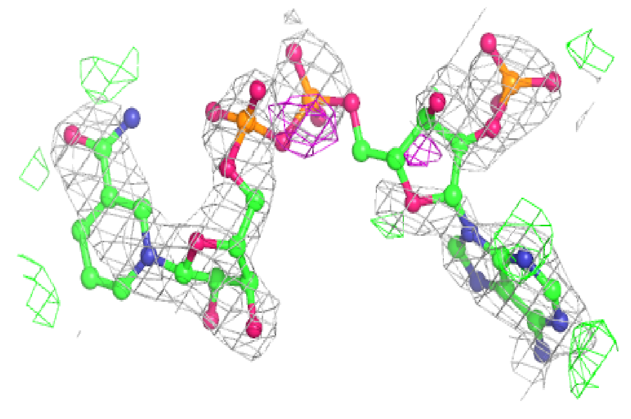

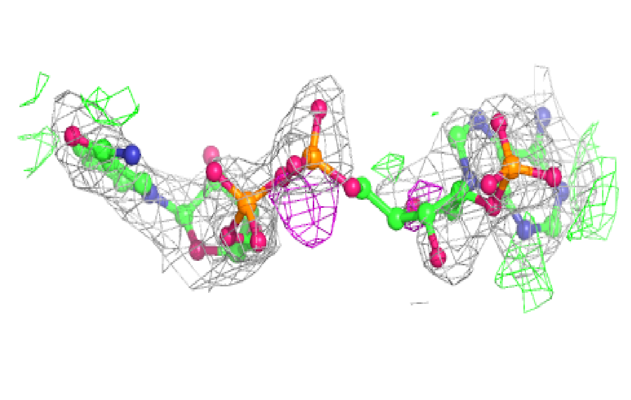

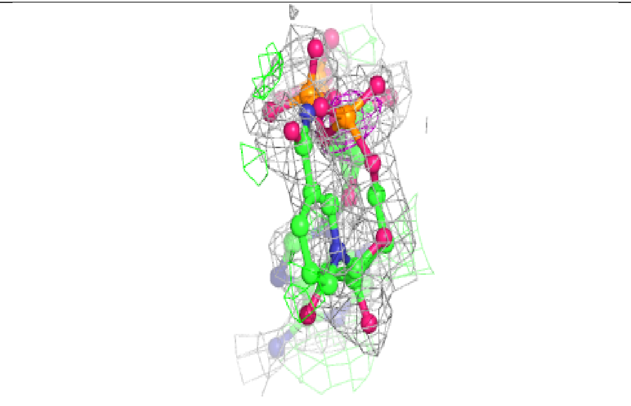

**Electron density around NAP E 401:**

2mF$_o$-DF$_c$ (at 0.7 rmsd) in gray
mF$_o$-DF$_c$ (at 3 rmsd) in purple (negative)
and green (positive)

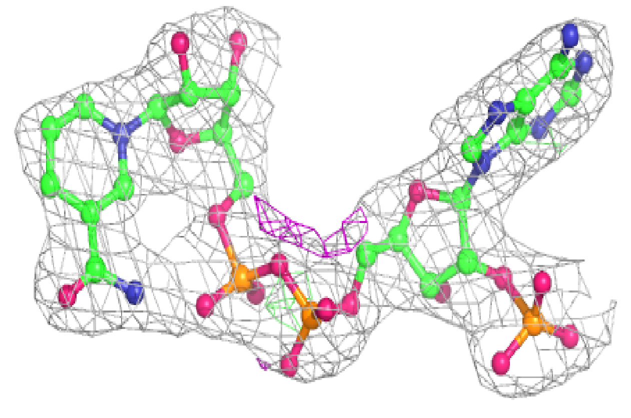

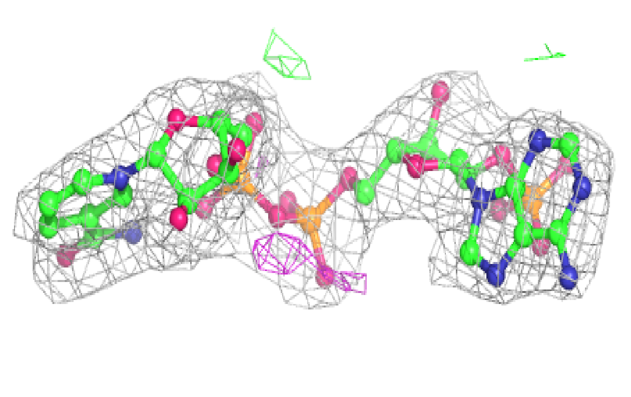

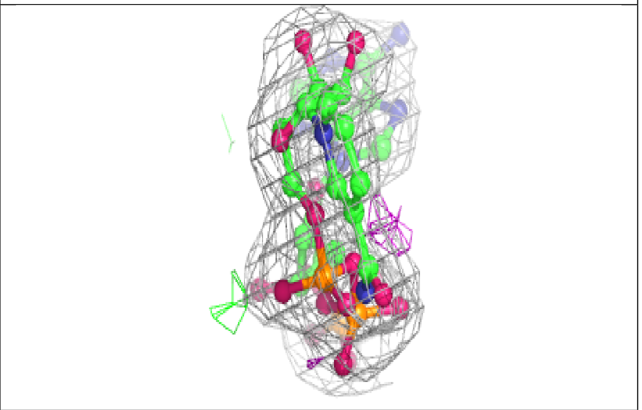

**Electron density around NAP F 401:**

2mF$_o$-DF$_c$ (at 0.7 rmsd) in gray
mF$_o$-DF$_c$ (at 3 rmsd) in purple (negative)
and green (positive)

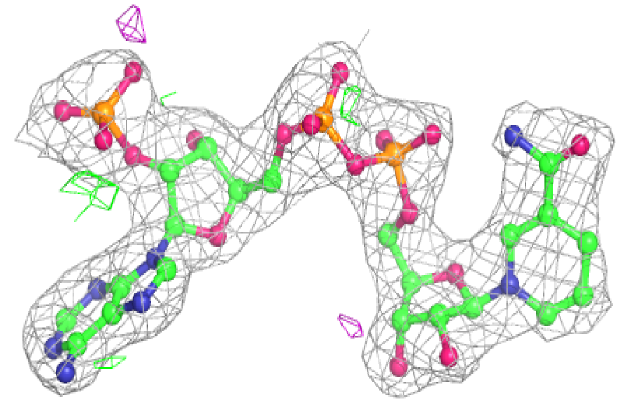

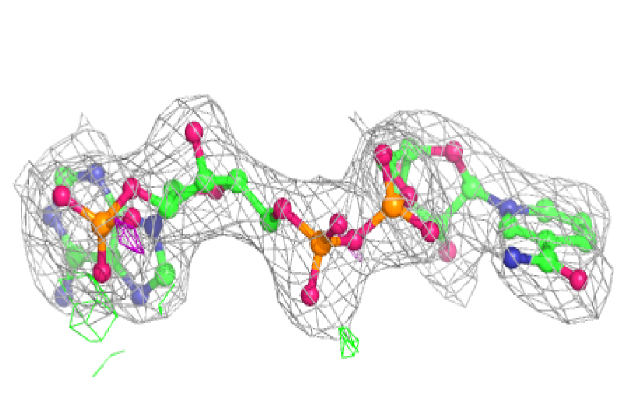

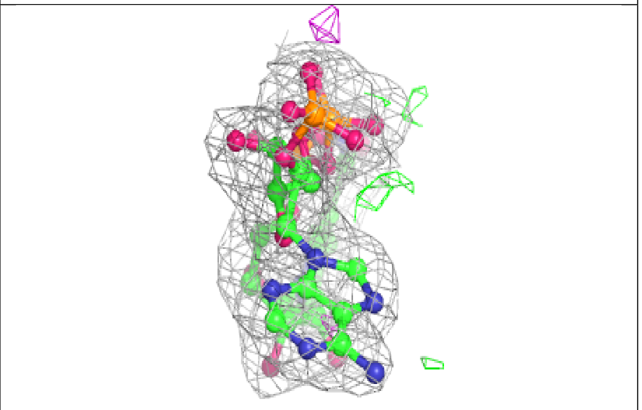

**Electron density around NAP C 401:**

$2mF_o$-$DF_c$ (at 0.7 rmsd) in gray
$mF_o$-$DF_c$ (at 3 rmsd) in purple (negative)
and green (positive)

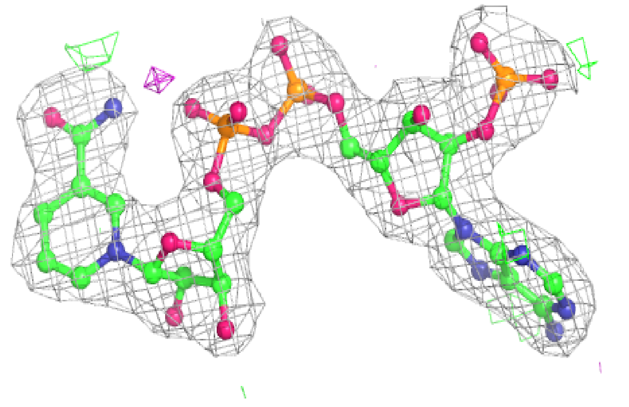

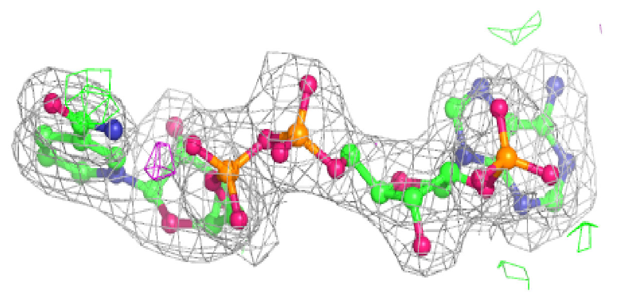

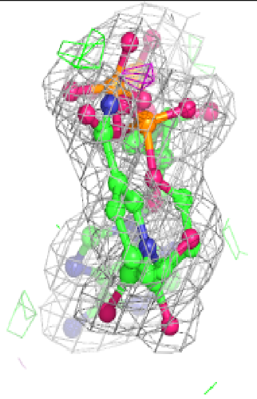

**Electron density around NAP A 401:**

$2mF_o$-$DF_c$ (at 0.7 rmsd) in gray
$mF_o$-$DF_c$ (at 3 rmsd) in purple (negative)
and green (positive)

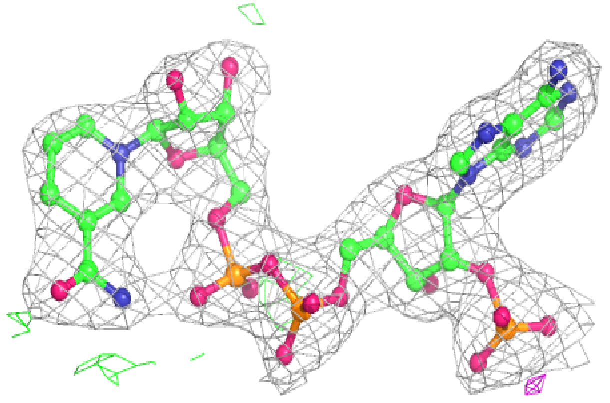

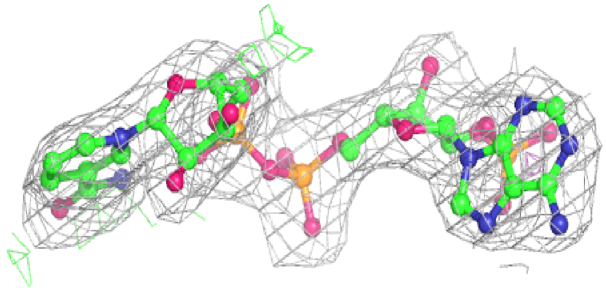

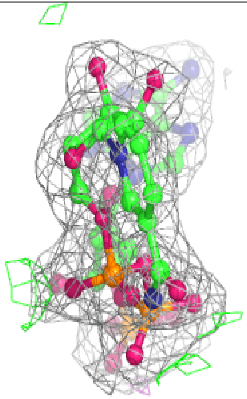

## 6.5 Other polymers ⓘ

There are no such residues in this entry.
