# Supplementary material for: Naegleria fowleri: Protein structures to facilitate drug discovery for the deadly, pathogenic free-living amoeba
Source: PLoS One. 2021 Mar 24;16(3):e0241738. doi: 10.1371/journal.pone.0241738 (PMC7990177; doi:10.1371/journal.pone.0241738)
Supplement: S1 File — (ZIP) [file pone.0241738.s002.zip › 6blj_full_validation.pdf]

Full wwPDB X-ray Structure Validation Report (i)

May 16, 2020 – 11:07 am BST

PDB ID	:	6BLJ
Title	:	Crystal structure of cytoplasmic Serine-tRNA ligase from Naegleria fowleri in complex with AMP
Authors	:	Seattle Structural Genomics Center for Infectious Disease (SSGCID)
Deposited on	:	2017-11-10
Resolution	:	2.10 Å(reported)

This is a Full wwPDB X-ray Structure Validation Report for a publicly released PDB entry.

We welcome your comments at *validation@mail.wwpdb.org*
A user guide is available at
https://www.wwpdb.org/validation/2017/XrayValidationReportHelp
with specific help available everywhere you see the (i) symbol.

The following versions of software and data (see references (i)) were used in the production of this report:

MolProbity	:	4.02b-467
Mogul	:	1.8.5 (274361), CSD as541be (2020)
Xtriage (Phenix)	:	1.13
EDS	:	2.11
buster-report	:	1.1.7 (2018)
Percentile statistics	:	20191225.v01 (using entries in the PDB archive December 25th 2019)
Refmac	:	5.8.0158
CCP4	:	7.0.044 (Gargrove)
Ideal geometry (proteins)	:	Engh & Huber (2001)
Ideal geometry (DNA, RNA)	:	Parkinson et al. (1996)
Validation Pipeline (wwPDB-VP)	:	2.11

1 Overall quality at a glance (i)

The following experimental techniques were used to determine the structure:
X-RAY DIFFRACTION

The reported resolution of this entry is 2.10 Å.

Percentile scores (ranging between 0-100) for global validation metrics of the entry are shown in the following graphic. The table shows the number of entries on which the scores are based.

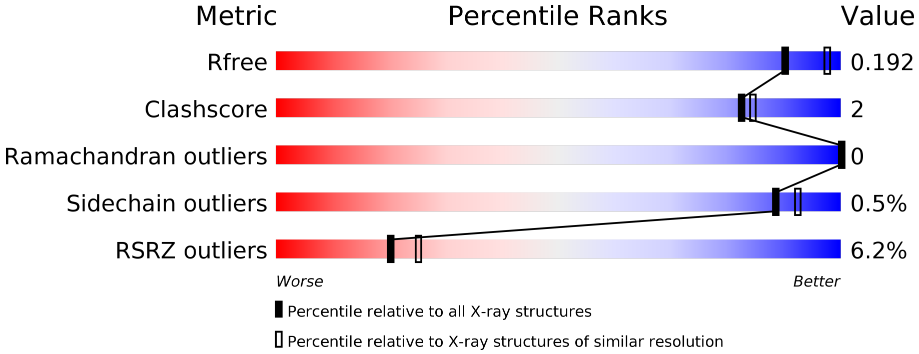

Metric	Whole archive (#Entries)	Similar resolution (#Entries, resolution range(Å))
R_{free}	130704	5197 (2.10-2.10)
Clashscore	141614	5710 (2.10-2.10)
Ramachandran outliers	138981	5647 (2.10-2.10)
Sidechain outliers	138945	5648 (2.10-2.10)
RSRZ outliers	127900	5083 (2.10-2.10)

The table below summarises the geometric issues observed across the polymeric chains and their fit to the electron density. The red, orange, yellow and green segments on the lower bar indicate the fraction of residues that contain outliers for >=3, 2, 1 and 0 types of geometric quality criteria respectively. A grey segment represents the fraction of residues that are not modelled. The numeric value for each fraction is indicated below the corresponding segment, with a dot representing fractions <=5% The upper red bar (where present) indicates the fraction of residues that have poor fit to the electron density. The numeric value is given above the bar.

Mol	Chain	Length	Quality of chain
1	A	485	4% / 91% 6% •
1	B	485	5% / 84% 5% 10%
1	C	485	8% / 85% • 11%

2 Entry composition (i)

There are 4 unique types of molecules in this entry. The entry contains 11672 atoms, of which 0 are hydrogens and 0 are deuteriums.

In the tables below, the ZeroOcc column contains the number of atoms modelled with zero occupancy, the AltConf column contains the number of residues with at least one atom in alternate conformation and the Trace column contains the number of residues modelled with at most 2 atoms.

- Molecule 1 is a protein called Serine-tRNA ligase.

Mol	Chain	Residues	Atoms					ZeroOcc	AltConf	Trace
1	A	473	Total 3755	C 2383	N 630	O 720	S 22	0	9	0
1	B	435	Total 3425	C 2164	N 583	O 658	S 20	0	5	0
1	C	431	Total 3382	C 2140	N 571	O 650	S 21	0	7	0

- Molecule 2 is ADENOSINE MONOPHOSPHATE (three-letter code: AMP) (formula: $C_{10}H_{14}N_5O_7P$).

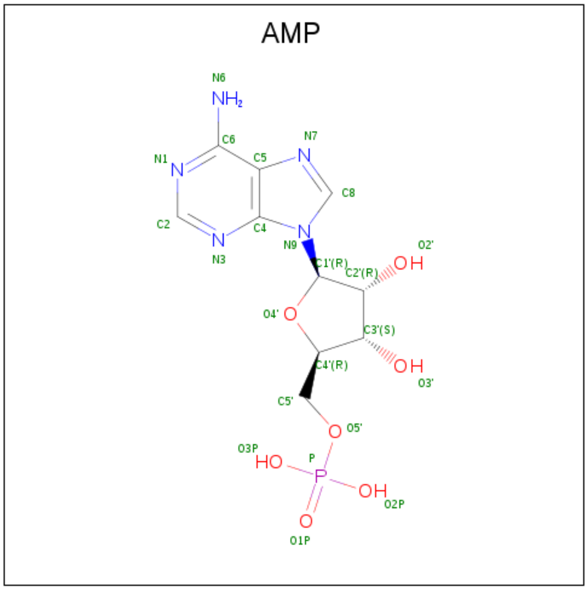

Mol	Chain	Residues	Atoms					ZeroOcc	AltConf
2	A	1	Total 23	C 10	N 5	O 7	P 1	0	0
2	B	1	Total 23	C 10	N 5	O 7	P 1	0	0
2	C	1	Total 23	C 10	N 5	O 7	P 1	0	0

- Molecule 3 is 1,2-ETHANEDIOL (three-letter code: EDO) (formula: $C_2H_6O_2$).

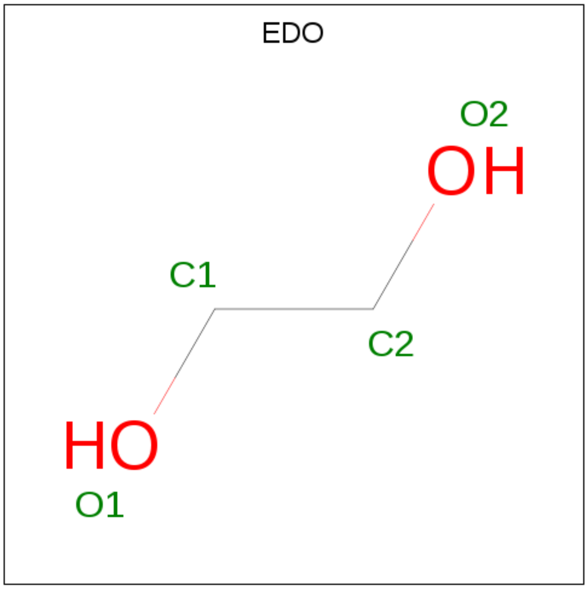

Mol	Chain	Residues	Atoms	ZeroOcc	AltConf
3	A	1	Total C O<br>4 2 2	0	0
3	B	1	Total C O<br>4 2 2	0	0
3	C	1	Total C O<br>4 2 2	0	0

- Molecule 4 is water.

Mol	Chain	Residues	Atoms	ZeroOcc	AltConf
4	A	359	Total O<br>361 361	0	3
4	B	413	Total O<br>419 419	0	6
4	C	247	Total O<br>249 249	0	2

3 Residue-property plots

These plots are drawn for all protein, RNA and DNA chains in the entry. The first graphic for a chain summarises the proportions of the various outlier classes displayed in the second graphic. The second graphic shows the sequence view annotated by issues in geometry and electron density. Residues are color-coded according to the number of geometric quality criteria for which they contain at least one outlier: green = 0, yellow = 1, orange = 2 and red = 3 or more. A red dot above a residue indicates a poor fit to the electron density (RSRZ > 2). Stretches of 2 or more consecutive residues without any outlier are shown as a green connector. Residues present in the sample, but not in the model, are shown in grey.

- Molecule 1: Serine-tRNA ligase

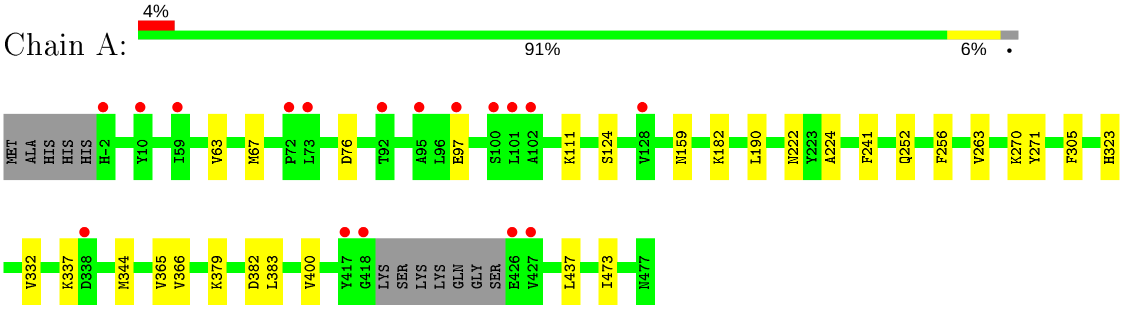

- Molecule 1: Serine-tRNA ligase

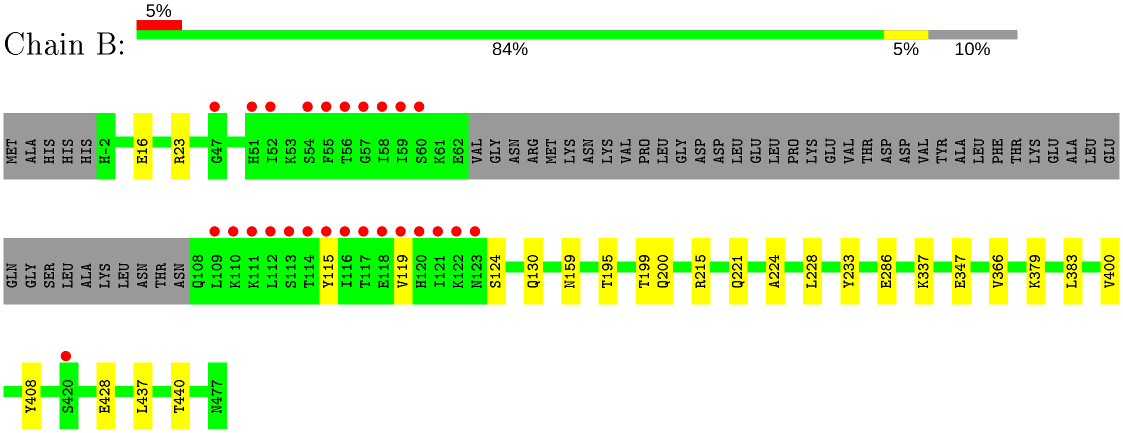

- Molecule 1: Serine-tRNA ligase

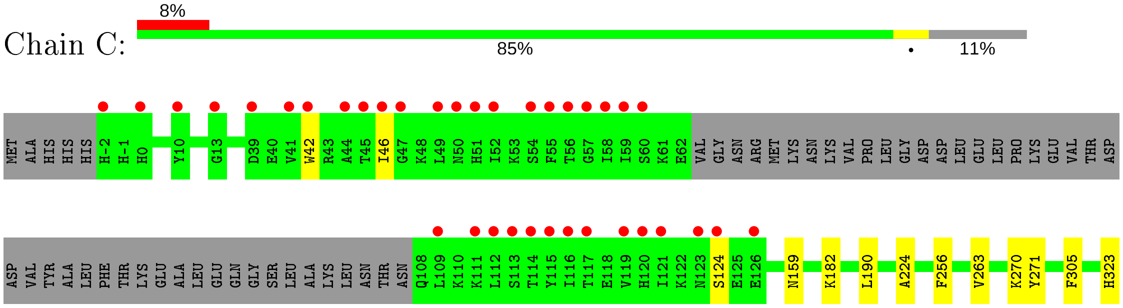

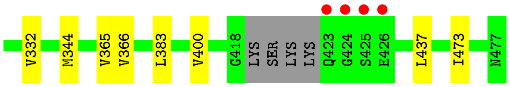
V332
M344
V365
V366
L383
V400
G418
LYS
SER
LYS
LYS
Q423
G424
S425
E426
L437
I473
N477

4 Data and refinement statistics (i)

Property	Value	Source
Space group	P 31 2 1	Depositor
Cell constants<br>a, b, c, α, β, γ	168.19Å 168.19Å 111.50Å<br>90.00° 90.00° 120.00°	Depositor
Resolution (Å)	42.05 – 2.10<br>49.36 – 2.10	Depositor<br>EDS
% Data completeness<br>(in resolution range)	99.8 (42.05-2.10)<br>99.8 (49.36-2.10)	Depositor<br>EDS
R_{merge}	0.07	Depositor
R_{sym}	(Not available)	Depositor
$< I/\sigma(I) >$ [1]	4.04 (at 2.10Å)	Xtriage
Refinement program	PHENIX	Depositor
R, R_{free}	0.158 , 0.191<br>0.159 , 0.192	Depositor<br>DCC
R_{free} test set	1963 reflections (1.86%)	wwPDB-VP
Wilson B-factor (Å^2)	31.3	Xtriage
Anisotropy	0.020	Xtriage
Bulk solvent k_{sol}(e/Å^3), B_{sol}(Å^2)	0.33 , 59.6	EDS
L-test for twinning[2]	$< \|L\| > = 0.50$, $< L^2 > = 0.33$	Xtriage
Estimated twinning fraction	0.018 for -h,-k,l	Xtriage
F_o,F_c correlation	0.96	EDS
Total number of atoms	11672	wwPDB-VP
Average B, all atoms (Å^2)	40.0	wwPDB-VP

Xtriage's analysis on translational NCS is as follows: *The largest off-origin peak in the Patterson function is 2.24% of the height of the origin peak. No significant pseudotranslation is detected.*

[1]Intensities estimated from amplitudes.

[2]Theoretical values of $< |L| >$, $< L^2 >$ for acentric reflections are 0.5, 0.333 respectively for untwinned datasets, and 0.375, 0.2 for perfectly twinned datasets.

5 Model quality (i)

5.1 Standard geometry (i)

Bond lengths and bond angles in the following residue types are not validated in this section: AMP, EDO

The Z score for a bond length (or angle) is the number of standard deviations the observed value is removed from the expected value. A bond length (or angle) with $|Z| > 5$ is considered an outlier worth inspection. RMSZ is the root-mean-square of all Z scores of the bond lengths (or angles).

Mol	Chain	Bond lengths		Bond angles	
		RMSZ	#|Z| >5	RMSZ	#|Z| >5
1	A	0.40	0/3852	0.55	1/5207 (0.0%)
1	B	0.45	0/3507	0.56	0/4741
1	C	0.38	0/3469	0.54	0/4692
All	All	0.41	0/10828	0.55	1/14640 (0.0%)

There are no bond length outliers.

All (1) bond angle outliers are listed below:

Mol	Chain	Res	Type	Atoms	Z	Observed(°)	Ideal(°)
1	A	241	PHE	CB-CA-C	-5.11	100.18	110.40

There are no chirality outliers.

There are no planarity outliers.

5.2 Too-close contacts (i)

In the following table, the Non-H and H(model) columns list the number of non-hydrogen atoms and hydrogen atoms in the chain respectively. The H(added) column lists the number of hydrogen atoms added and optimized by MolProbity. The Clashes column lists the number of clashes within the asymmetric unit, whereas Symm-Clashes lists symmetry related clashes.

Mol	Chain	Non-H	H(model)	H(added)	Clashes	Symm-Clashes
1	A	3755	0	3669	22	0
1	B	3425	0	3287	18	0
1	C	3382	0	3230	13	0
2	A	23	0	12	0	0
2	B	23	0	12	0	0
2	C	23	0	12	0	0
3	A	4	0	6	2	0

Continued on next page...

Continued from previous page...

Mol	Chain	Non-H	H(model)	H(added)	Clashes	Symm-Clashes
3	B	4	0	6	1	0
3	C	4	0	6	0	0
4	A	361	0	0	3	0
4	B	419	0	0	6	0
4	C	249	0	0	0	0
All	All	11672	0	10240	52	0

The all-atom clashscore is defined as the number of clashes found per 1000 atoms (including hydrogen atoms). The all-atom clashscore for this structure is 2.

All (52) close contacts within the same asymmetric unit are listed below, sorted by their clash magnitude.

Atom-1	Atom-2	Interatomic distance (Å)	Clash overlap (Å)
1:B:347:GLU:OE2	4:B:601:HOH:O	2.03	0.75
1:B:408:TYR:OH	4:B:602:HOH:O	2.05	0.73
1:B:286:GLU:OE2	4:B:603:HOH:O	2.11	0.68
1:A:252[A]:GLN:NE2	4:A:601:HOH:O	2.25	0.66
1:A:256:PHE:HD1	1:A:271:TYR:HH	1.42	0.66
1:B:159:ASN:HB3	1:B:366:VAL:HG13	1.79	0.63
1:C:383[B]:LEU:HB2	1:C:400:VAL:HB	1.82	0.62
1:B:379:LYS:HZ3	3:B:501:EDO:H11	1.65	0.61
1:B:428:GLU:OE1	4:B:604:HOH:O	2.17	0.59
1:C:256:PHE:HD2	1:C:271:TYR:CZ	2.22	0.57
1:A:383[A]:LEU:HB2	1:A:400:VAL:HB	1.85	0.57
1:C:224:ALA:HB1	1:C:437:LEU:HD23	1.87	0.56
1:C:159:ASN:HB3	1:C:366:VAL:HG13	1.88	0.55
1:B:224:ALA:HB1	1:B:437:LEU:HD23	1.89	0.55
1:C:383[A]:LEU:HB3	1:C:400:VAL:HB	1.89	0.55
1:A:224:ALA:HB1	1:A:437:LEU:HD23	1.89	0.54
1:A:263[A]:VAL:HG22	1:C:263[A]:VAL:HG22	1.89	0.54
1:B:383:LEU:HB3	1:B:400:VAL:HB	1.90	0.53
1:A:383[B]:LEU:HB3	1:A:400:VAL:HB	1.90	0.52
1:A:63:VAL:O	1:A:67:MET:HG2	2.09	0.52
1:C:365:VAL:HG22	1:C:383[B]:LEU:CD2	2.41	0.50
1:A:190:LEU:HD22	1:A:473:ILE:HG22	1.94	0.49
1:A:159:ASN:HB3	1:A:366[A]:VAL:CG1	2.43	0.49
1:A:256:PHE:HD1	1:A:271:TYR:OH	1.94	0.49
1:B:221:GLN:HG3	1:B:440:THR:HG21	1.94	0.48
1:B:159:ASN:HB3	1:B:366:VAL:CG1	2.44	0.47
1:A:365:VAL:HG22	1:A:383[A]:LEU:CD2	2.45	0.47
1:B:200:GLN:NE2	4:B:614:HOH:O	2.47	0.47

Continued on next page...

Continued from previous page...

Atom-1	Atom-2	Interatomic distance (Å)	Clash overlap (Å)
1:C:263[B]:VAL:HG13	1:C:270:LYS:HB2	1.97	0.47
1:C:305:PHE:HA	1:C:323:HIS:O	2.15	0.47
1:A:379:LYS:NZ	3:A:501:EDO:H12	2.30	0.46
1:A:379:LYS:HZ3	3:A:501:EDO:H12	1.81	0.46
1:A:76:ASP:OD1	1:A:111:LYS:NZ	2.49	0.45
1:C:42:TRP:CE2	1:C:46:ILE:HD11	2.51	0.45
1:A:159:ASN:HB3	1:A:366[A]:VAL:HG13	1.99	0.45
1:B:337:LYS:NZ	1:B:428:GLU:OE1	2.46	0.44
1:C:182:LYS:HB2	1:C:182:LYS:HE2	1.68	0.44
1:A:263[B]:VAL:HG13	1:A:270:LYS:HB2	2.00	0.44
1:B:115:TYR:CE2	1:B:119:VAL:HG21	2.53	0.44
1:B:228:LEU:HG	1:B:437:LEU:HD22	1.99	0.43
1:C:190:LEU:HD22	1:C:473:ILE:HG22	2.00	0.43
1:A:305:PHE:HA	1:A:323:HIS:O	2.19	0.42
1:A:366[B]:VAL:CG2	1:A:382:ASP:HB2	2.50	0.42
1:B:215:ARG:NH2	4:B:605:HOH:O	2.33	0.42
1:A:182:LYS:HB2	1:A:182:LYS:HE2	1.77	0.41
1:C:332:VAL:HG11	1:C:344:MET:HG2	2.02	0.41
1:B:195:THR:O	1:B:199:THR:HG23	2.21	0.41
1:A:332:VAL:HG11	1:A:344:MET:HG2	2.03	0.41
1:B:16:GLU:OE2	1:B:23:ARG:NH1	2.44	0.41
1:A:97:GLU:HG3	4:A:614:HOH:O	2.21	0.41
1:A:337:LYS:NZ	4:A:623:HOH:O	2.54	0.40
1:B:228:LEU:HB3	1:B:233:TYR:HB2	2.02	0.40

There are no symmetry-related clashes.

5.3 Torsion angles (i)

5.3.1 Protein backbone (i)

In the following table, the Percentiles column shows the percent Ramachandran outliers of the chain as a percentile score with respect to all X-ray entries followed by that with respect to entries of similar resolution.

The Analysed column shows the number of residues for which the backbone conformation was analysed, and the total number of residues.

Mol	Chain	Analysed	Favoured	Allowed	Outliers	Percentiles	
1	A	478/485 (99%)	474 (99%)	4 (1%)	0	100	100

Continued on next page...

Continued from previous page...

Mol	Chain	Analysed	Favoured	Allowed	Outliers	Percentiles	
1	B	436/485 (90%)	431 (99%)	5 (1%)	0	100	100
1	C	432/485 (89%)	426 (99%)	6 (1%)	0	100	100
All	All	1346/1455 (92%)	1331 (99%)	15 (1%)	0	100	100

There are no Ramachandran outliers to report.

5.3.2 Protein sidechains ⓘ

In the following table, the Percentiles column shows the percent sidechain outliers of the chain as a percentile score with respect to all X-ray entries followed by that with respect to entries of similar resolution.

The Analysed column shows the number of residues for which the sidechain conformation was analysed, and the total number of residues.

Mol	Chain	Analysed	Rotameric	Outliers	Percentiles	
1	A	405/426 (95%)	403 (100%)	2 (0%)	88	92
1	B	364/426 (85%)	362 (100%)	2 (0%)	88	92
1	C	358/426 (84%)	357 (100%)	1 (0%)	92	95
All	All	1127/1278 (88%)	1122 (100%)	5 (0%)	88	94

All (5) residues with a non-rotameric sidechain are listed below:

Mol	Chain	Res	Type
1	A	124	SER
1	A	222	ASN
1	B	124	SER
1	B	130	GLN
1	C	124	SER

Some sidechains can be flipped to improve hydrogen bonding and reduce clashes. All (3) such sidechains are listed below:

Mol	Chain	Res	Type
1	A	434	ASN
1	B	221	GLN
1	B	434	ASN

5.3.3 RNA (i)

There are no RNA molecules in this entry.

5.4 Non-standard residues in protein, DNA, RNA chains (i)

There are no non-standard protein/DNA/RNA residues in this entry.

5.5 Carbohydrates (i)

There are no carbohydrates in this entry.

5.6 Ligand geometry (i)

6 ligands are modelled in this entry.

In the following table, the Counts columns list the number of bonds (or angles) for which Mogul statistics could be retrieved, the number of bonds (or angles) that are observed in the model and the number of bonds (or angles) that are defined in the Chemical Component Dictionary. The Link column lists molecule types, if any, to which the group is linked. The Z score for a bond length (or angle) is the number of standard deviations the observed value is removed from the expected value. A bond length (or angle) with $|Z| > 2$ is considered an outlier worth inspection. RMSZ is the root-mean-square of all Z scores of the bond lengths (or angles).

Mol	Type	Chain	Res	Link	Bond lengths			Bond angles		
					Counts	RMSZ	$\#\|Z\| > 2$	Counts	RMSZ	$\#\|Z\| > 2$
3	EDO	B	501	-	3,3,3	0.49	0	2,2,2	0.25	0
2	AMP	C	500	-	22,25,25	0.69	0	25,38,38	0.98	2 (8%)
2	AMP	B	500	-	22,25,25	0.64	0	25,38,38	0.96	2 (8%)
2	AMP	A	500	-	22,25,25	0.71	0	25,38,38	0.89	2 (8%)
3	EDO	A	501	-	3,3,3	0.46	0	2,2,2	0.26	0
3	EDO	C	501	-	3,3,3	0.49	0	2,2,2	0.32	0

In the following table, the Chirals column lists the number of chiral outliers, the number of chiral centers analysed, the number of these observed in the model and the number defined in the Chemical Component Dictionary. Similar counts are reported in the Torsion and Rings columns. '-' means no outliers of that kind were identified.

Mol	Type	Chain	Res	Link	Chirals	Torsions	Rings
3	EDO	B	501	-	-	0/1/1/1	-
2	AMP	C	500	-	-	0/6/26/26	0/3/3/3
2	AMP	B	500	-	-	0/6/26/26	0/3/3/3

Continued on next page...

Continued from previous page...

Mol	Type	Chain	Res	Link	Chirals	Torsions	Rings
2	AMP	A	500	-	-	0/6/26/26	0/3/3/3
3	EDO	A	501	-	-	0/1/1/1	-
3	EDO	C	501	-	-	0/1/1/1	-

There are no bond length outliers.

All (6) bond angle outliers are listed below:

Mol	Chain	Res	Type	Atoms	Z	Observed(^{o})	Ideal(^{o})
2	C	500	AMP	O3P-P-O2P	2.87	118.62	107.64
2	B	500	AMP	O3P-P-O2P	2.75	118.16	107.64
2	A	500	AMP	C5-C6-N6	2.40	124.01	120.35
2	B	500	AMP	C5-C6-N6	2.39	123.98	120.35
2	C	500	AMP	C5-C6-N6	2.30	123.84	120.35
2	A	500	AMP	O2P-P-O1P	2.13	119.03	110.68

There are no chirality outliers.

There are no torsion outliers.

There are no ring outliers.

2 monomers are involved in 3 short contacts:

Mol	Chain	Res	Type	Clashes	Symm-Clashes
3	B	501	EDO	1	0
3	A	501	EDO	2	0

The following is a two-dimensional graphical depiction of Mogul quality analysis of bond lengths, bond angles, torsion angles, and ring geometry for all instances of the Ligand of Interest. In addition, ligands with molecular weight > 250 and outliers as shown on the validation Tables will also be included. For torsion angles, if less then 5% of the Mogul distribution of torsion angles is within 10 degrees of the torsion angle in question, then that torsion angle is considered an outlier. Any bond that is central to one or more torsion angles identified as an outlier by Mogul will be highlighted in the graph. For rings, the root-mean-square deviation (RMSD) between the ring in question and similar rings identified by Mogul is calculated over all ring torsion angles. If the average RMSD is greater than 60 degrees and the minimal RMSD between the ring in question and any Mogul-identified rings is also greater than 60 degrees, then that ring is considered an outlier. The outliers are highlighted in purple. The color gray indicates Mogul did not find sufficient equivalents in the CSD to analyse the geometry.

Ligand AMP C 500

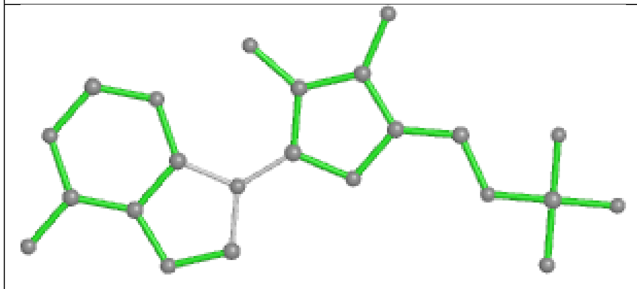

Bond lengths

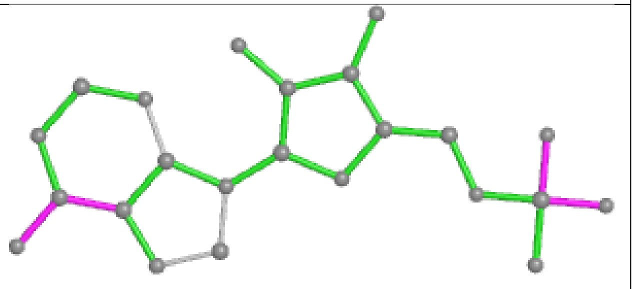

Bond angles

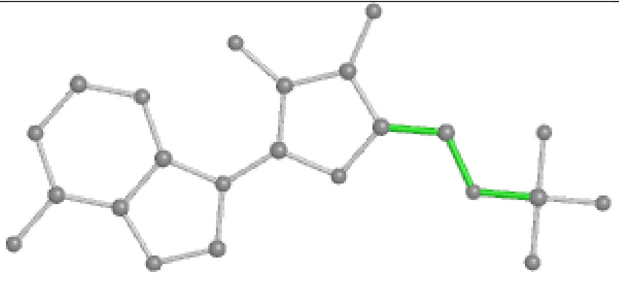

Torsions

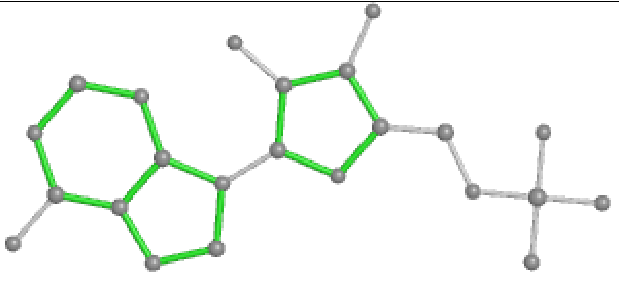

Rings

Ligand AMP B 500

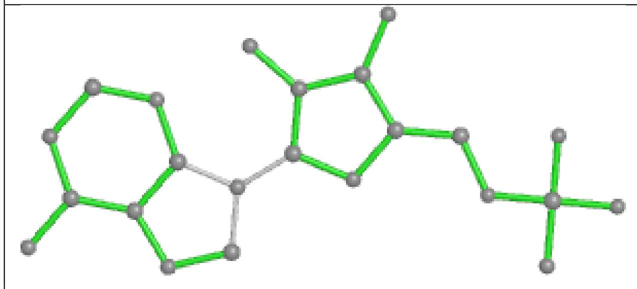

Bond lengths

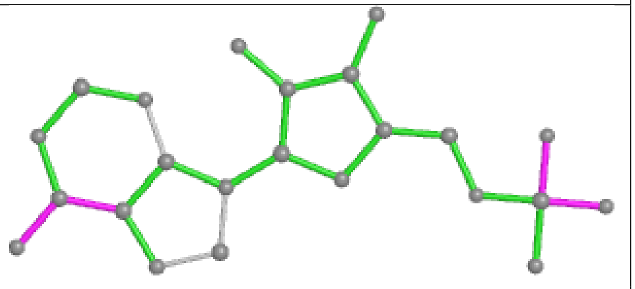

Bond angles

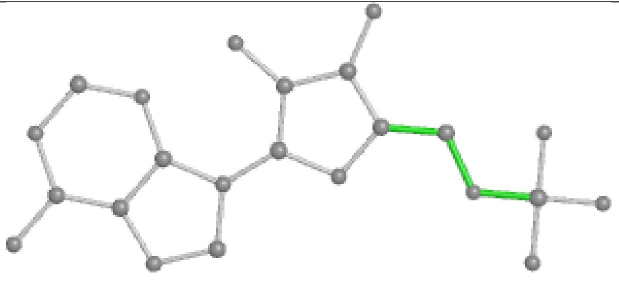

Torsions

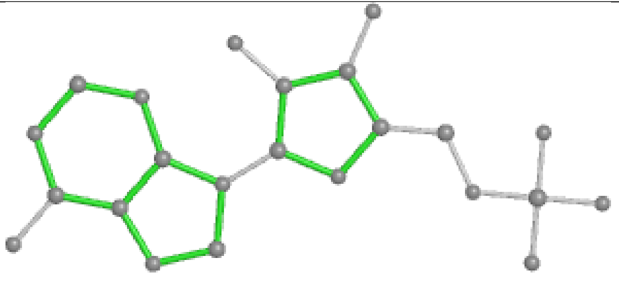

Rings

Ligand AMP A 500

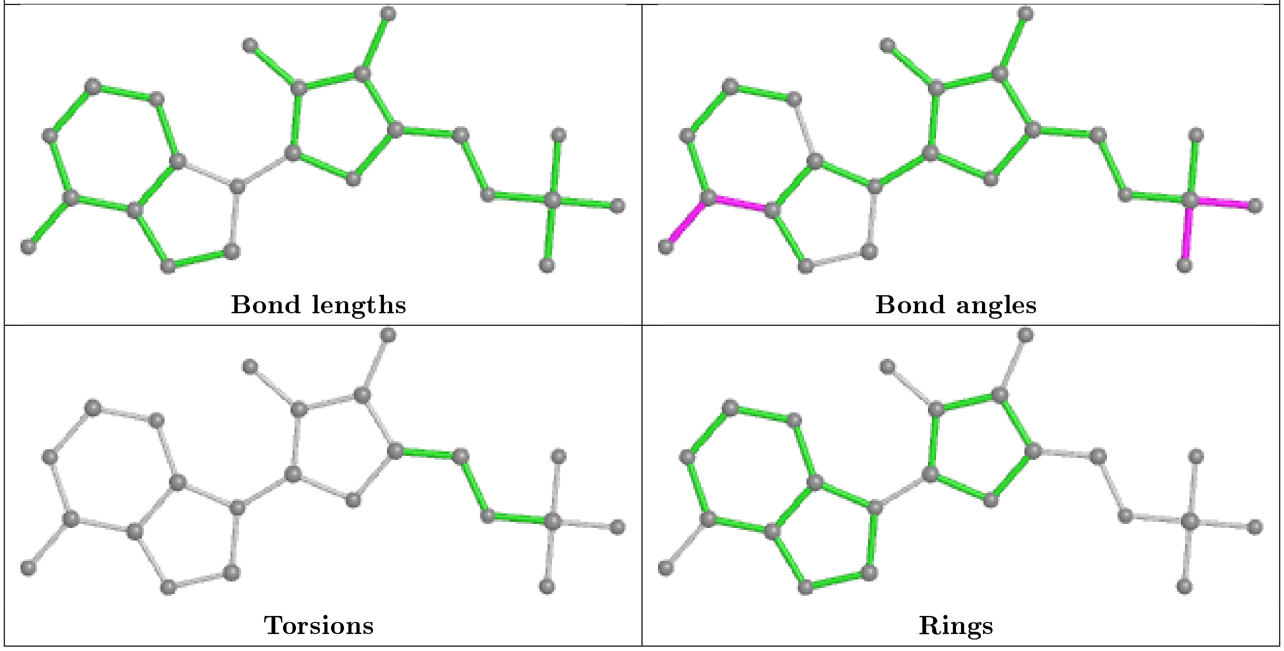

5.7 Other polymers (i)

There are no such residues in this entry.

5.8 Polymer linkage issues (i)

There are no chain breaks in this entry.

6 Fit of model and data

6.1 Protein, DNA and RNA chains

In the following table, the column labelled '#RSRZ> 2' contains the number (and percentage) of RSRZ outliers, followed by percent RSRZ outliers for the chain as percentile scores relative to all X-ray entries and entries of similar resolution. The OWAB column contains the minimum, median, 95th percentile and maximum values of the occupancy-weighted average B-factor per residue. The column labelled 'Q< 0.9' lists the number of (and percentage) of residues with an average occupancy less than 0.9.

Mol	Chain	Analysed	<RSRZ>	#RSRZ>2			OWAB(Å^2)	Q<0.9
1	A	473/485 (97%)	0.03	17 (3%)	42	49	20, 36, 60, 93	0
1	B	435/485 (89%)	-0.11	26 (5%)	21	27	17, 30, 84, 118	0
1	C	431/485 (88%)	0.21	40 (9%)	8	11	21, 39, 95, 118	0
All	All	1339/1455 (92%)	0.04	83 (6%)	20	25	17, 35, 85, 118	0

All (83) RSRZ outliers are listed below:

Mol	Chain	Res	Type	RSRZ
1	C	121	ILE	8.7
1	B	60	SER	8.6
1	B	112	LEU	8.5
1	C	10	TYR	7.8
1	C	55	PHE	7.7
1	C	54	SER	7.0
1	B	109	LEU	6.9
1	C	56	THR	6.5
1	C	112	LEU	6.1
1	B	113	SER	5.9
1	C	425	SER	5.8
1	C	57	GLY	5.7
1	B	58	ILE	5.7
1	C	52	ILE	5.6
1	C	59	ILE	5.5
1	C	116	ILE	5.5
1	B	57	GLY	5.4
1	C	46	ILE	5.3
1	B	55	PHE	5.2
1	B	59	ILE	5.2
1	B	119	VAL	5.1
1	C	113	SER	5.1
1	C	115	TYR	5.0

Continued on next page...

Continued from previous page...

Mol	Chain	Res	Type	RSRZ
1	B	115	TYR	4.9
1	B	110	LYS	4.8
1	C	51	HIS	4.8
1	C	119	VAL	4.8
1	A	10	TYR	4.6
1	C	123	ASN	4.3
1	C	49	LEU	4.3
1	C	124	SER	4.3
1	C	114	THR	4.2
1	B	120	HIS	4.2
1	C	47	GLY	4.1
1	B	114	THR	4.1
1	C	120	HIS	4.0
1	C	117	THR	4.0
1	C	426	GLU	3.9
1	B	123	ASN	3.9
1	B	111	LYS	3.9
1	B	116	ILE	3.8
1	C	424	GLY	3.8
1	B	56	THR	3.7
1	B	51	HIS	3.6
1	C	60	SER	3.5
1	A	95	ALA	3.4
1	C	111	LYS	3.4
1	B	117	THR	3.2
1	C	45	THR	3.2
1	B	52	ILE	3.2
1	C	41	VAL	3.1
1	A	102	ALA	3.0
1	B	121	ILE	2.9
1	A	418	GLY	2.9
1	C	44	ALA	2.9
1	C	109	LEU	2.8
1	C	-2	HIS	2.8
1	C	50	ASN	2.8
1	A	59	ILE	2.7
1	A	97	GLU	2.7
1	C	0	HIS	2.7
1	C	58	ILE	2.6
1	A	338	ASP	2.6
1	C	42	TRP	2.5
1	C	423	GLN	2.5

Continued on next page...

Continued from previous page...

Mol	Chain	Res	Type	RSRZ
1	A	-2	HIS	2.5
1	B	118	GLU	2.5
1	B	54	SER	2.4
1	A	72	PRO	2.4
1	A	427	VAL	2.3
1	B	47	GLY	2.3
1	A	92	THR	2.2
1	A	101	LEU	2.2
1	A	128	VAL	2.2
1	B	420	SER	2.1
1	A	73	LEU	2.1
1	C	126	GLU	2.1
1	B	122	LYS	2.1
1	A	417	TYR	2.1
1	A	100	SER	2.1
1	C	13	GLY	2.1
1	C	39	ASP	2.0
1	A	426	GLU	2.0

6.2 Non-standard residues in protein, DNA, RNA chains (i)

There are no non-standard protein/DNA/RNA residues in this entry.

6.3 Carbohydrates (i)

There are no carbohydrates in this entry.

6.4 Ligands (i)

In the following table, the Atoms column lists the number of modelled atoms in the group and the number defined in the chemical component dictionary. The B-factors column lists the minimum, median, 95th percentile and maximum values of B factors of atoms in the group. The column labelled 'Q< 0.9' lists the number of atoms with occupancy less than 0.9.

Mol	Type	Chain	Res	Atoms	RSCC	RSR	B-factors(Å^2)	Q<0.9
3	EDO	A	501	4/4	0.80	0.20	68,72,74,75	0
3	EDO	B	501	4/4	0.85	0.17	56,62,64,64	0
3	EDO	C	501	4/4	0.85	0.09	63,66,70,72	0
2	AMP	B	500	23/23	0.97	0.09	19,22,29,32	0
2	AMP	C	500	23/23	0.98	0.09	21,26,37,43	0

Continued on next page...

Continued from previous page...

Mol	Type	Chain	Res	Atoms	RSCC	RSR	B-factors($Å^2$)	Q<0.9
2	AMP	A	500	23/23	0.99	0.14	17,23,36,43	0

The following is a graphical depiction of the model fit to experimental electron density of all instances of the Ligand of Interest. In addition, ligands with molecular weight > 250 and outliers as shown on the geometry validation Tables will also be included. Each fit is shown from different orientation to approximate a three-dimensional view.

Electron density around AMP B 500:

$2mF_o$-DF_c (at 0.7 rmsd) in gray
mF_o-DF_c (at 3 rmsd) in purple (negative)
and green (positive)

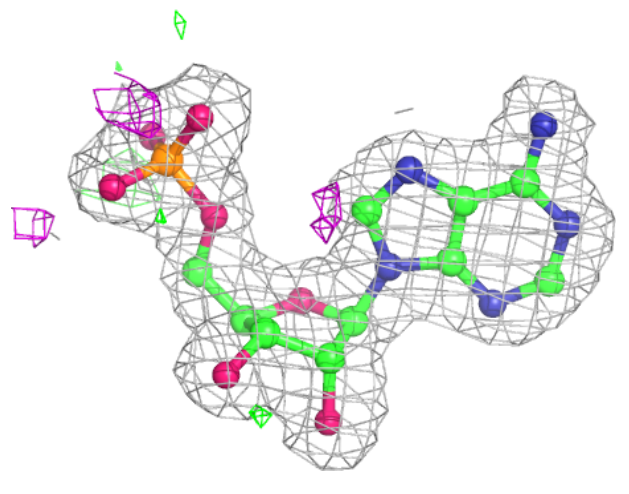

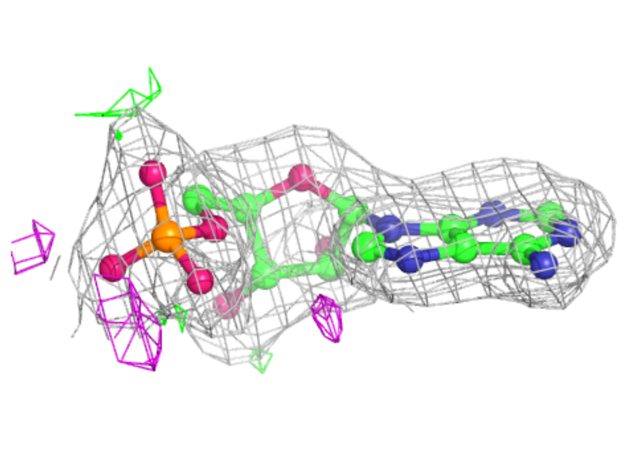

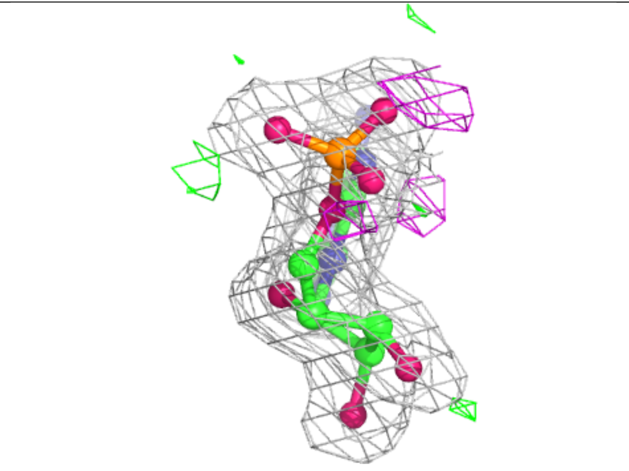

Electron density around AMP C 500:

2mF$_o$-DF$_c$ (at 0.7 rmsd) in gray
mF$_o$-DF$_c$ (at 3 rmsd) in purple (negative)
and green (positive)

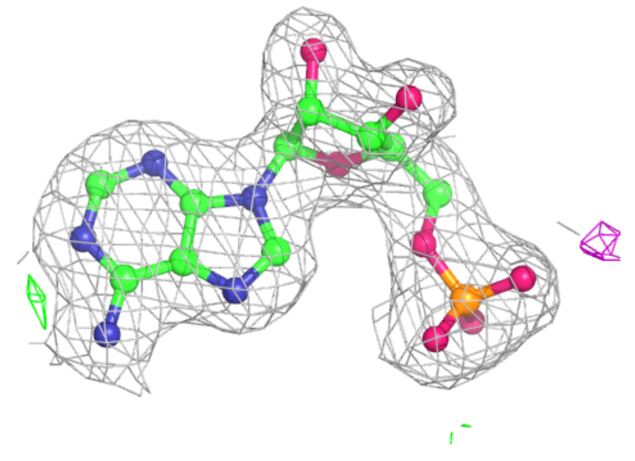

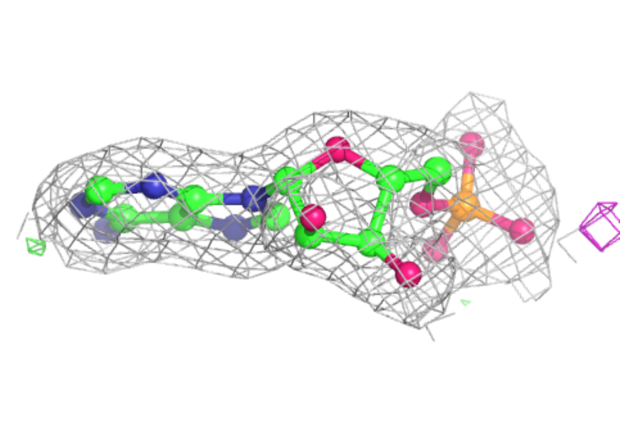

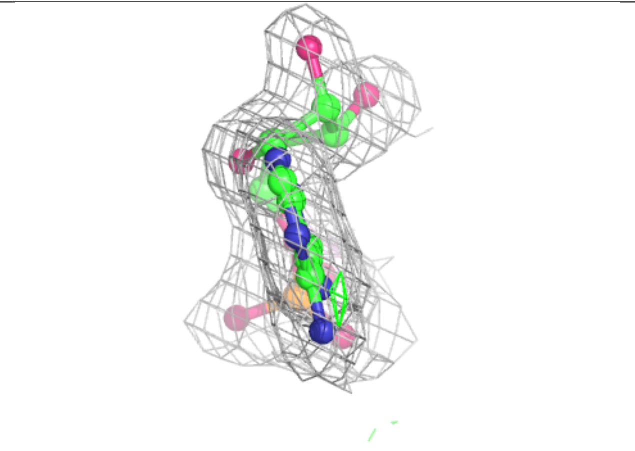

Electron density around AMP A 500:

$2mF_o$-DF_c (at 0.7 rmsd) in gray
mF_o-DF_c (at 3 rmsd) in purple (negative)
and green (positive)

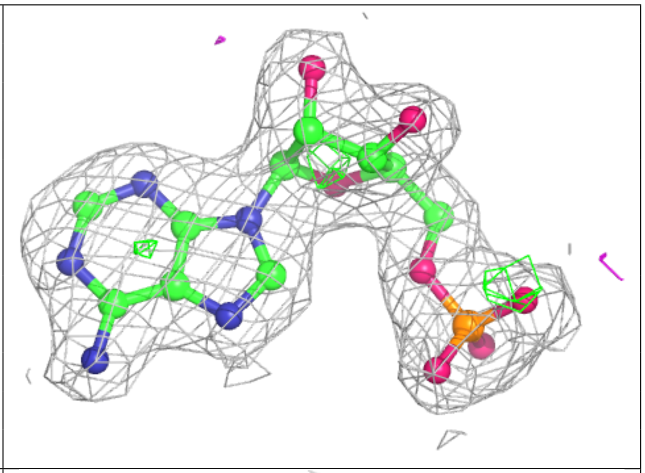

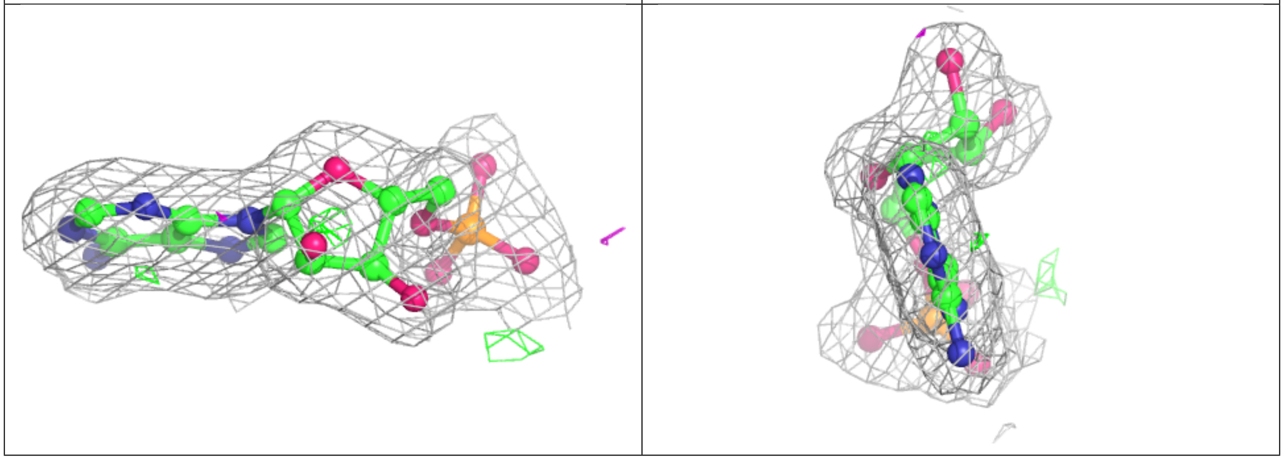

6.5 Other polymers ⓘ

There are no such residues in this entry.
