# Supplementary material for: Naegleria fowleri: Protein structures to facilitate drug discovery for the deadly, pathogenic free-living amoeba
Source: PLoS One. 2021 Mar 24;16(3):e0241738. doi: 10.1371/journal.pone.0241738 (PMC7990177; doi:10.1371/journal.pone.0241738)
Supplement: S1 File — (ZIP) [file pone.0241738.s002.zip › 6c87_full_validation.pdf]

Full wwPDB X-ray Structure Validation Report (i)

May 22, 2020 – 02:11 am BST

PDB ID	:	6C87
Title	:	Crystal structure of rab gdp dissociation inhibitor alpha from Naegleria fowleri
Authors	:	Seattle Structural Genomics Center for Infectious Disease (SSGCID)
Deposited on	:	2018-01-24
Resolution	:	2.60 Å(reported)

This is a Full wwPDB X-ray Structure Validation Report for a publicly released PDB entry.

We welcome your comments at *validation@mail.wwpdb.org*
A user guide is available at
https://www.wwpdb.org/validation/2017/XrayValidationReportHelp
with specific help available everywhere you see the (i) symbol.

The following versions of software and data (see references (i)) were used in the production of this report:

MolProbity	:	4.02b-467
Mogul	:	1.8.5 (274361), CSD as541be (2020)
Xtriage (Phenix)	:	1.13
EDS	:	2.11
Percentile statistics	:	20191225.v01 (using entries in the PDB archive December 25th 2019)
Refmac	:	5.8.0158
CCP4	:	7.0.044 (Gargrove)
Ideal geometry (proteins)	:	Engh & Huber (2001)
Ideal geometry (DNA, RNA)	:	Parkinson et al. (1996)
Validation Pipeline (wwPDB-VP)	:	2.11

1 Overall quality at a glance (i)

The following experimental techniques were used to determine the structure:
X-RAY DIFFRACTION

The reported resolution of this entry is 2.60 Å.

Percentile scores (ranging between 0-100) for global validation metrics of the entry are shown in the following graphic. The table shows the number of entries on which the scores are based.

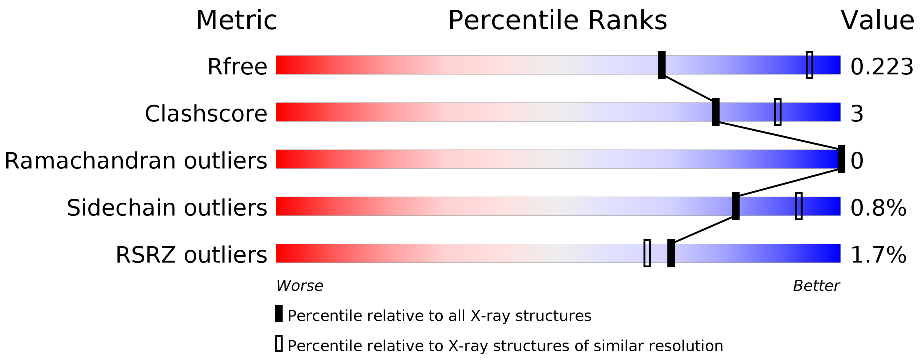

Metric	Whole archive (#Entries)	Similar resolution (#Entries, resolution range(Å))
R_{free}	130704	3163 (2.60-2.60)
Clashscore	141614	3518 (2.60-2.60)
Ramachandran outliers	138981	3455 (2.60-2.60)
Sidechain outliers	138945	3455 (2.60-2.60)
RSRZ outliers	127900	3104 (2.60-2.60)

The table below summarises the geometric issues observed across the polymeric chains and their fit to the electron density. The red, orange, yellow and green segments on the lower bar indicate the fraction of residues that contain outliers for >=3, 2, 1 and 0 types of geometric quality criteria respectively. A grey segment represents the fraction of residues that are not modelled. The numeric value for each fraction is indicated below the corresponding segment, with a dot representing fractions <=5% The upper red bar (where present) indicates the fraction of residues that have poor fit to the electron density. The numeric value is given above the bar.

Mol	Chain	Length	Quality of chain
1	A	452	% 91% 7% •
1	B	452	2% 90% 8% •
1	C	452	2% 89% 9% •
1	D	452	% 90% 8% •

2 Entry composition

There are 3 unique types of molecules in this entry. The entry contains 13820 atoms, of which 0 are hydrogens and 0 are deuteriums.

In the tables below, the ZeroOcc column contains the number of atoms modelled with zero occupancy, the AltConf column contains the number of residues with at least one atom in alternate conformation and the Trace column contains the number of residues modelled with at most 2 atoms.

- Molecule 1 is a protein called Rab GDP dissociation inhibitor alpha.

Mol	Chain	Residues	Atoms					ZeroOcc	AltConf	Trace
1	A	442	Total 3349	C 2146	N 539	O 644	S 20	0	0	0
1	B	441	Total 3355	C 2152	N 535	O 648	S 20	0	1	0
1	C	441	Total 3362	C 2155	N 537	O 650	S 20	0	0	0
1	D	441	Total 3348	C 2138	N 540	O 650	S 20	0	0	0

- Molecule 2 is SULFATE ION (three-letter code: SO4) (formula: O_4S).

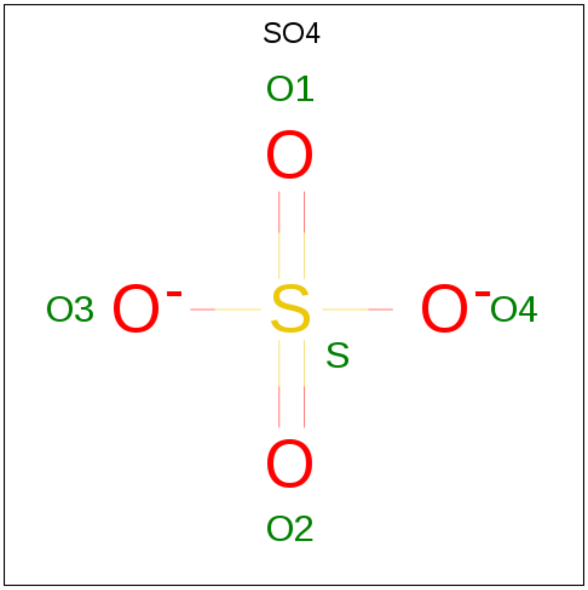

Mol	Chain	Residues	Atoms			ZeroOcc	AltConf
2	A	1	Total 5	O 4	S 1	0	0
2	B	1	Total 5	O 4	S 1	0	0

- Molecule 3 is water.

Mol	Chain	Residues	Atoms	ZeroOcc	AltConf
3	A	99	Total O<br>99 99	0	0
3	B	117	Total O<br>118 118	0	1
3	C	78	Total O<br>78 78	0	0
3	D	99	Total O<br>101 101	0	2

3 Residue-property plots ⓘ

These plots are drawn for all protein, RNA and DNA chains in the entry. The first graphic for a chain summarises the proportions of the various outlier classes displayed in the second graphic. The second graphic shows the sequence view annotated by issues in geometry and electron density. Residues are color-coded according to the number of geometric quality criteria for which they contain at least one outlier: green = 0, yellow = 1, orange = 2 and red = 3 or more. A red dot above a residue indicates a poor fit to the electron density (RSRZ > 2). Stretches of 2 or more consecutive residues without any outlier are shown as a green connector. Residues present in the sample, but not in the model, are shown in grey.

- Molecule 1: Rab GDP dissociation inhibitor alpha

Chain A:

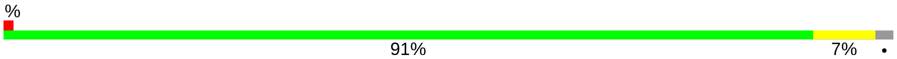

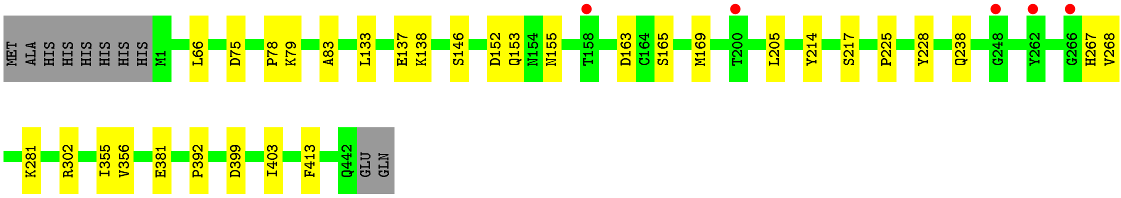

- Molecule 1: Rab GDP dissociation inhibitor alpha

Chain B:

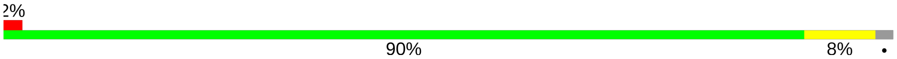

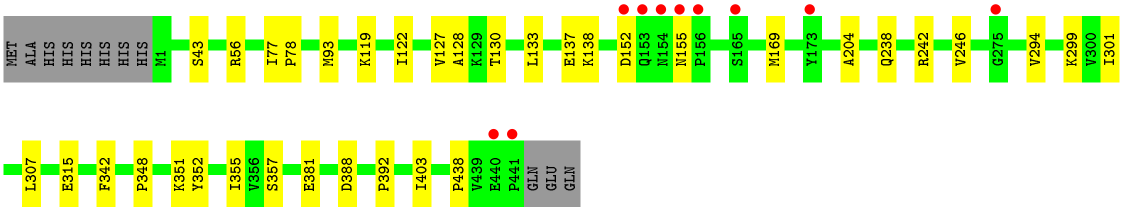

- Molecule 1: Rab GDP dissociation inhibitor alpha

Chain C:

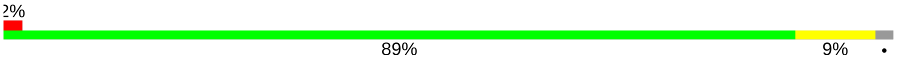

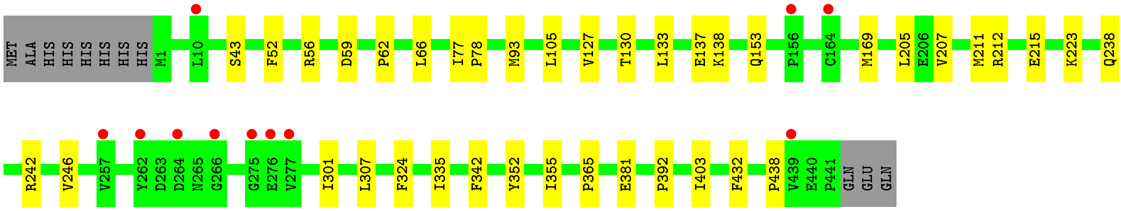

- Molecule 1: Rab GDP dissociation inhibitor alpha

Chain D:

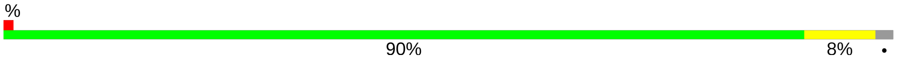

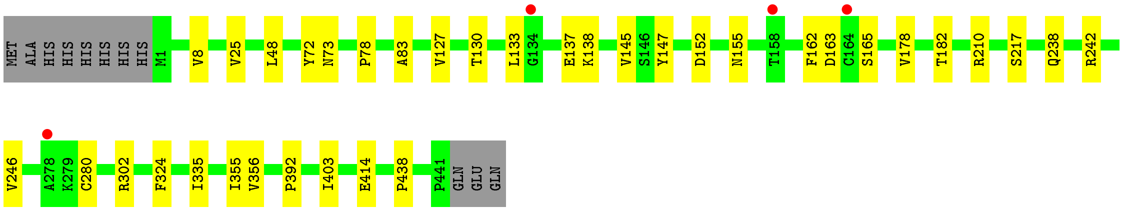
MET
ALA
HIS
HIS
HIS
HIS
HIS
HIS
M1
V8
V25
L48
Y72
N73
P78
A83
V127
T130
L133
G134
E137
K138
V145
S146
Y147
D152
N155
T158
F162
D163
C164
S165
V178
T182
R210
S217
Q238
R242
V246
A278
K279
C280
R302
F324
I335
I355
V356
P392
I403
E414
P438
P441
GLN
GLU
GLN

4 Data and refinement statistics (i)

Property	Value	Source
Space group	P 21 21 21	Depositor
Cell constants<br>a, b, c, α, β, γ	97.34Å 103.12Å 195.55Å<br>90.00° 90.00° 90.00°	Depositor
Resolution (Å)	47.95 – 2.60<br>47.95 – 2.60	Depositor<br>EDS
% Data completeness<br>(in resolution range)	99.8 (47.95-2.60)<br>99.8 (47.95-2.60)	Depositor<br>EDS
R_{merge}	0.07	Depositor
R_{sym}	(Not available)	Depositor
$< I/\sigma(I) >$ [1]	3.32 (at 2.61Å)	Xtriage
Refinement program	PHENIX 1.13_2998	Depositor
R, R_{free}	0.172 , 0.223<br>0.172 , 0.223	Depositor<br>DCC
R_{free} test set	1938 reflections (3.17%)	wwPDB-VP
Wilson B-factor (Å^2)	45.4	Xtriage
Anisotropy	0.267	Xtriage
Bulk solvent k_{sol}(e/Å^3), B_{sol}(Å^2)	0.32 , 64.2	EDS
L-test for twinning[2]	$< \lvert L \rvert > = 0.50$, $< L^2 > = 0.33$	Xtriage
Estimated twinning fraction	No twinning to report.	Xtriage
F_o,F_c correlation	0.95	EDS
Total number of atoms	13820	wwPDB-VP
Average B, all atoms (Å^2)	52.0	wwPDB-VP

Xtriage's analysis on translational NCS is as follows: *The analyses of the Patterson function reveals a significant off-origin peak that is 34.56 % of the origin peak, indicating pseudo-translational symmetry. The chance of finding a peak of this or larger height randomly in a structure without pseudo-translational symmetry is equal to 6.6909e-04. The detected translational NCS is most likely also responsible for the elevated intensity ratio.*

[1]Intensities estimated from amplitudes.

[2]Theoretical values of $< \lvert L \rvert >$, $< L^2 >$ for acentric reflections are 0.5, 0.333 respectively for untwinned datasets, and 0.375, 0.2 for perfectly twinned datasets.

5 Model quality (i)

5.1 Standard geometry (i)

Bond lengths and bond angles in the following residue types are not validated in this section: SO4

The Z score for a bond length (or angle) is the number of standard deviations the observed value is removed from the expected value. A bond length (or angle) with $|Z| > 5$ is considered an outlier worth inspection. RMSZ is the root-mean-square of all Z scores of the bond lengths (or angles).

Mol	Chain	Bond lengths		Bond angles	
		RMSZ	#|Z| >5	RMSZ	#|Z| >5
1	A	0.31	0/3428	0.51	0/4675
1	B	0.31	0/3438	0.51	0/4687
1	C	0.31	0/3442	0.51	0/4691
1	D	0.31	0/3425	0.52	0/4671
All	All	0.31	0/13733	0.51	0/18724

There are no bond length outliers.

There are no bond angle outliers.

There are no chirality outliers.

There are no planarity outliers.

5.2 Too-close contacts (i)

In the following table, the Non-H and H(model) columns list the number of non-hydrogen atoms and hydrogen atoms in the chain respectively. The H(added) column lists the number of hydrogen atoms added and optimized by MolProbity. The Clashes column lists the number of clashes within the asymmetric unit, whereas Symm-Clashes lists symmetry related clashes.

Mol	Chain	Non-H	H(model)	H(added)	Clashes	Symm-Clashes
1	A	3349	0	3167	15	0
1	B	3355	0	3171	20	0
1	C	3362	0	3182	20	0
1	D	3348	0	3168	20	0
2	A	5	0	0	0	0
2	B	5	0	0	0	0
3	A	99	0	0	0	0
3	B	118	0	0	2	0
3	C	78	0	0	1	0

Continued on next page...

Continued from previous page...

Mol	Chain	Non-H	H(model)	H(added)	Clashes	Symm-Clashes
3	D	101	0	0	1	0
All	All	13820	0	12688	75	0

The all-atom clashscore is defined as the number of clashes found per 1000 atoms (including hydrogen atoms). The all-atom clashscore for this structure is 3.

All (75) close contacts within the same asymmetric unit are listed below, sorted by their clash magnitude.

Atom-1	Atom-2	Interatomic distance (Å)	Clash overlap (Å)
1:B:78:PRO:HG3	1:B:355:ILE:HD12	1.61	0.80
1:C:78:PRO:HG3	1:C:355:ILE:HD12	1.63	0.77
1:A:78:PRO:HG3	1:A:355:ILE:HD12	1.68	0.75
1:C:56:ARG:NH2	1:C:381:GLU:OE1	2.20	0.75
1:D:78:PRO:HG3	1:D:355:ILE:HD12	1.69	0.73
1:C:130:THR:O	1:C:138:LYS:NZ	2.21	0.72
1:A:66:LEU:HD11	1:A:381:GLU:HG3	1.71	0.71
1:B:56:ARG:NH1	3:B:601:HOH:O	2.26	0.68
1:A:133:LEU:HD22	1:A:137:GLU:HG2	1.75	0.68
1:D:392:PRO:HB3	1:D:403:ILE:HB	1.77	0.67
1:B:169:MET:HE2	1:B:204:ALA:HB1	1.76	0.64
1:C:133:LEU:HD22	1:C:137:GLU:HG2	1.82	0.62
1:A:133:LEU:O	1:A:138:LYS:NZ	2.34	0.60
1:A:153:GLN:HE21	1:A:205:LEU:HD22	1.66	0.59
1:A:152:ASP:HB3	1:A:155:ASN:HB3	1.86	0.58
1:B:348:PRO:HG2	1:B:351:LYS:HG3	1.85	0.57
1:C:93:MET:HE1	1:C:432:PHE:CZ	2.41	0.56
1:C:392:PRO:HB3	1:C:403:ILE:HB	1.88	0.55
1:A:163:ASP:OD2	1:A:165:SER:OG	2.22	0.54
1:B:133:LEU:HD22	1:B:137:GLU:HG2	1.89	0.54
1:B:56:ARG:NH2	1:B:381:GLU:OE1	2.40	0.54
1:D:163:ASP:OD2	1:D:165:SER:OG	2.23	0.54
1:C:93:MET:O	1:C:438:PRO:HG3	2.08	0.53
1:D:73:ASN:ND2	3:D:503:HOH:O	2.42	0.53
1:D:133:LEU:HD22	1:D:137:GLU:HG2	1.90	0.53
1:A:392:PRO:HB3	1:A:403:ILE:HB	1.92	0.52
1:B:392:PRO:HB3	1:B:403:ILE:HB	1.92	0.52
3:B:682[B]:HOH:O	1:D:83:ALA:O	2.18	0.51
1:B:152:ASP:HB3	1:B:155:ASN:HB3	1.93	0.50
1:C:66:LEU:HD11	1:C:381:GLU:HG3	1.92	0.50
1:A:214:TYR:CE2	1:A:225:PRO:HG3	2.47	0.50
1:C:301:ILE:HD12	1:C:365:PRO:HB2	1.94	0.50

Continued on next page...

Continued from previous page...

Atom-1	Atom-2	Interatomic distance (Å)	Clash overlap (Å)
1:C:93:MET:HE1	1:C:432:PHE:HZ	1.77	0.50
1:D:147:TYR:OH	1:D:162:PHE:HB2	2.12	0.50
1:C:212:ARG:NH2	3:C:504:HOH:O	2.45	0.50
1:D:127:VAL:O	1:D:130:THR:HG22	2.11	0.49
1:A:83:ALA:HB3	1:A:217:SER:HB3	1.95	0.49
1:A:75:ASP:OD2	1:A:228:TYR:OH	2.26	0.49
1:C:43:SER:HB3	1:C:77:ILE:HD12	1.95	0.48
1:D:83:ALA:HB3	1:D:217:SER:HB3	1.96	0.48
1:A:268:VAL:HG23	1:A:399:ASP:O	2.14	0.47
1:B:294:VAL:HG12	1:B:392:PRO:HA	1.97	0.47
1:B:93:MET:O	1:B:438:PRO:HG3	2.14	0.47
1:D:242:ARG:O	1:D:246:VAL:HG23	2.16	0.45
1:D:127:VAL:HG21	1:D:145:VAL:HG21	1.98	0.45
1:D:130:THR:O	1:D:138:LYS:HE2	2.17	0.45
1:C:307:LEU:HB2	1:C:352:TYR:HB2	1.99	0.45
1:C:207:VAL:O	1:C:211:MET:HG3	2.16	0.45
1:B:299:LYS:HE2	1:B:388:ASP:OD1	2.17	0.45
1:B:43:SER:HB3	1:B:77:ILE:HD12	1.99	0.45
1:D:178:VAL:HB	1:D:182:THR:HB	1.98	0.45
1:B:127:VAL:O	1:B:130:THR:HG22	2.17	0.44
1:B:307:LEU:HB2	1:B:352:TYR:HB2	2.01	0.43
1:C:242:ARG:O	1:C:246:VAL:HG23	2.18	0.43
1:D:152:ASP:HB3	1:D:155:ASN:HB3	2.01	0.43
1:B:128:ALA:O	1:B:138:LYS:HD3	2.19	0.42
1:A:79:LYS:HB2	1:A:413:PHE:CE2	2.54	0.42
1:C:153:GLN:HE21	1:C:205:LEU:HD22	1.83	0.42
1:C:52:PHE:CD1	1:C:62:PRO:HG3	2.55	0.42
1:A:302:ARG:HA	1:A:356:VAL:O	2.20	0.41
1:C:324:PHE:HB2	1:C:335:ILE:HB	2.02	0.41
1:B:119:LYS:HD2	1:B:315:GLU:OE1	2.20	0.41
1:D:210:ARG:NH1	1:D:414:GLU:OE2	2.53	0.41
1:C:127:VAL:O	1:C:130:THR:HG22	2.20	0.41
1:D:8:VAL:HG23	1:D:280:CYS:HB3	2.01	0.41
1:D:25:VAL:HB	1:D:438:PRO:HB3	2.01	0.41
1:B:133:LEU:O	1:B:138:LYS:NZ	2.51	0.41
1:B:242:ARG:O	1:B:246:VAL:HG23	2.20	0.41
1:D:324:PHE:HB2	1:D:335:ILE:HB	2.03	0.41
1:D:302:ARG:HA	1:D:356:VAL:O	2.21	0.40
1:B:301:ILE:O	1:B:357:SER:HA	2.21	0.40
1:C:105:LEU:HD11	1:C:223:LYS:HD3	2.03	0.40
1:A:267:HIS:HE1	1:A:281:LYS:O	2.05	0.40

Continued on next page...

Continued from previous page...

Atom-1	Atom-2	Interatomic distance (Å)	Clash overlap (Å)
1:B:122:ILE:HA	1:B:122:ILE:HD13	1.83	0.40
1:D:48:LEU:HD22	1:D:72:TYR:CE2	2.56	0.40

There are no symmetry-related clashes.

5.3 Torsion angles (i)

5.3.1 Protein backbone (i)

In the following table, the Percentiles column shows the percent Ramachandran outliers of the chain as a percentile score with respect to all X-ray entries followed by that with respect to entries of similar resolution.

The Analysed column shows the number of residues for which the backbone conformation was analysed, and the total number of residues.

Mol	Chain	Analysed	Favoured	Allowed	Outliers	Percentiles	
1	A	440/452 (97%)	433 (98%)	7 (2%)	0	100	100
1	B	440/452 (97%)	432 (98%)	8 (2%)	0	100	100
1	C	439/452 (97%)	432 (98%)	7 (2%)	0	100	100
1	D	439/452 (97%)	432 (98%)	7 (2%)	0	100	100
All	All	1758/1808 (97%)	1729 (98%)	29 (2%)	0	100	100

There are no Ramachandran outliers to report.

5.3.2 Protein sidechains (i)

In the following table, the Percentiles column shows the percent sidechain outliers of the chain as a percentile score with respect to all X-ray entries followed by that with respect to entries of similar resolution.

The Analysed column shows the number of residues for which the sidechain conformation was analysed, and the total number of residues.

Mol	Chain	Analysed	Rotameric	Outliers	Percentiles	
1	A	353/400 (88%)	350 (99%)	3 (1%)	81	92
1	B	355/400 (89%)	352 (99%)	3 (1%)	81	92
1	C	356/400 (89%)	351 (99%)	5 (1%)	67	85

Continued on next page...

Continued from previous page...

Mol	Chain	Analysed	Rotameric	Outliers	Percentiles	
1	D	355/400 (89%)	354 (100%)	1 (0%)	92	98
All	All	1419/1600 (89%)	1407 (99%)	12 (1%)	81	92

All (12) residues with a non-rotameric sidechain are listed below:

Mol	Chain	Res	Type
1	A	146	SER
1	A	169	MET
1	A	238	GLN
1	B	238	GLN
1	B	342[A]	PHE
1	B	342[B]	PHE
1	C	59	ASP
1	C	169	MET
1	C	215	GLU
1	C	238	GLN
1	C	342	PHE
1	D	238	GLN

Some sidechains can be flipped to improve hydrogen bonding and reduce clashes. All (10) such sidechains are listed below:

Mol	Chain	Res	Type
1	A	153	GLN
1	A	238	GLN
1	B	153	GLN
1	B	159	HIS
1	B	238	GLN
1	B	265	ASN
1	C	153	GLN
1	C	159	HIS
1	C	238	GLN
1	D	238	GLN

5.3.3 RNA (i)

There are no RNA molecules in this entry.

5.4 Non-standard residues in protein, DNA, RNA chains (i)

There are no non-standard protein/DNA/RNA residues in this entry.

5.5 Carbohydrates (i)

There are no carbohydrates in this entry.

5.6 Ligand geometry (i)

2 ligands are modelled in this entry.

In the following table, the Counts columns list the number of bonds (or angles) for which Mogul statistics could be retrieved, the number of bonds (or angles) that are observed in the model and the number of bonds (or angles) that are defined in the Chemical Component Dictionary. The Link column lists molecule types, if any, to which the group is linked. The Z score for a bond length (or angle) is the number of standard deviations the observed value is removed from the expected value. A bond length (or angle) with $|Z| > 2$ is considered an outlier worth inspection. RMSZ is the root-mean-square of all Z scores of the bond lengths (or angles).

Mol	Type	Chain	Res	Link	Bond lengths			Bond angles		
					Counts	RMSZ	$\#\|Z\| > 2$	Counts	RMSZ	$\#\|Z\| > 2$
2	SO4	B	500	-	4,4,4	0.13	0	6,6,6	0.06	0
2	SO4	A	500	-	4,4,4	0.13	0	6,6,6	0.10	0

There are no bond length outliers.

There are no bond angle outliers.

There are no chirality outliers.

There are no torsion outliers.

There are no ring outliers.

No monomer is involved in short contacts.

5.7 Other polymers (i)

There are no such residues in this entry.

5.8 Polymer linkage issues (i)

There are no chain breaks in this entry.

6 Fit of model and data (i)

6.1 Protein, DNA and RNA chains (i)

In the following table, the column labelled '#RSRZ> 2' contains the number (and percentage) of RSRZ outliers, followed by percent RSRZ outliers for the chain as percentile scores relative to all X-ray entries and entries of similar resolution. The OWAB column contains the minimum, median, 95th percentile and maximum values of the occupancy-weighted average B-factor per residue. The column labelled 'Q< 0.9' lists the number of (and percentage) of residues with an average occupancy less than 0.9.

Mol	Chain	Analysed	<RSRZ>	#RSRZ>2			OWAB(Å^2)	Q<0.9
1	A	442/452 (97%)	-0.24	5 (1%)	80	78	27, 48, 87, 112	0
1	B	441/452 (97%)	-0.26	10 (2%)	60	54	27, 46, 91, 132	0
1	C	441/452 (97%)	-0.19	11 (2%)	57	51	27, 52, 98, 125	0
1	D	441/452 (97%)	-0.30	4 (0%)	84	82	26, 47, 87, 109	0
All	All	1765/1808 (97%)	-0.25	30 (1%)	70	66	26, 49, 91, 132	0

All (30) RSRZ outliers are listed below:

Mol	Chain	Res	Type	RSRZ
1	C	277	VAL	5.0
1	C	156	PRO	4.0
1	B	156	PRO	3.3
1	B	441	PRO	3.2
1	C	257	VAL	3.0
1	A	248	GLY	2.9
1	C	264	ASP	2.8
1	C	266	GLY	2.8
1	B	440	GLU	2.7
1	D	164	CYS	2.7
1	B	165	SER	2.6
1	A	266	GLY	2.5
1	C	439	VAL	2.4
1	B	154	ASN	2.4
1	B	155	ASN	2.4
1	B	173	TYR	2.3
1	D	134	GLY	2.3
1	C	10	LEU	2.3
1	D	158	THR	2.2
1	B	152	ASP	2.2
1	A	262	TYR	2.2

Continued on next page...

Continued from previous page...

Mol	Chain	Res	Type	RSRZ
1	B	275	GLY	2.2
1	C	275	GLY	2.1
1	C	276	GLU	2.1
1	B	153	GLN	2.1
1	A	200	THR	2.1
1	A	158	THR	2.1
1	C	164	CYS	2.0
1	C	262	TYR	2.0
1	D	278	ALA	2.0

6.2 Non-standard residues in protein, DNA, RNA chains (i)

There are no non-standard protein/DNA/RNA residues in this entry.

6.3 Carbohydrates (i)

There are no carbohydrates in this entry.

6.4 Ligands (i)

In the following table, the Atoms column lists the number of modelled atoms in the group and the number defined in the chemical component dictionary. The B-factors column lists the minimum, median, 95th percentile and maximum values of B factors of atoms in the group. The column labelled 'Q< 0.9' lists the number of atoms with occupancy less than 0.9.

Mol	Type	Chain	Res	Atoms	RSCC	RSR	B-factors($Å^2$)	Q<0.9
2	SO4	A	500	5/5	0.90	0.32	106,108,110,111	0
2	SO4	B	500	5/5	0.93	0.18	93,97,99,103	0

6.5 Other polymers (i)

There are no such residues in this entry.
