# Supplementary material for: Naegleria fowleri: Protein structures to facilitate drug discovery for the deadly, pathogenic free-living amoeba
Source: PLoS One. 2021 Mar 24;16(3):e0241738. doi: 10.1371/journal.pone.0241738 (PMC7990177; doi:10.1371/journal.pone.0241738)
Supplement: S1 File — (ZIP) [file pone.0241738.s002.zip › 6cu3_full_validation.pdf]

Full wwPDB X-ray Structure Validation Report (i)

Aug 21, 2020 – 01:12 PM BST

PDB ID	:	6CU3
Title	:	Crystal structure of a protein arginine N-methyltransferase from Naegleria fowleri
Authors	:	Seattle Structural Genomics Center for Infectious Disease (SSGCID)
Deposited on	:	2018-03-23
Resolution	:	2.50 Å(reported)

This is a Full wwPDB X-ray Structure Validation Report for a publicly released PDB entry.

We welcome your comments at *validation@mail.wwpdb.org*
A user guide is available at
https://www.wwpdb.org/validation/2017/XrayValidationReportHelp
with specific help available everywhere you see the (i) symbol.

The following versions of software and data (see references (i)) were used in the production of this report:

MolProbity	:	4.02b-467
Mogul	:	1.8.5 (274361), CSD as541be (2020)
Xtriage (Phenix)	:	1.13
EDS	:	2.13.1
Percentile statistics	:	20191225.v01 (using entries in the PDB archive December 25th 2019)
Refmac	:	5.8.0158
CCP4	:	7.0.044 (Gargrove)
Ideal geometry (proteins)	:	Engh & Huber (2001)
Ideal geometry (DNA, RNA)	:	Parkinson et al. (1996)
Validation Pipeline (wwPDB-VP)	:	2.13.1

1 Overall quality at a glance (i)

The following experimental techniques were used to determine the structure:
X-RAY DIFFRACTION

The reported resolution of this entry is 2.50 Å.

Percentile scores (ranging between 0-100) for global validation metrics of the entry are shown in the following graphic. The table shows the number of entries on which the scores are based.

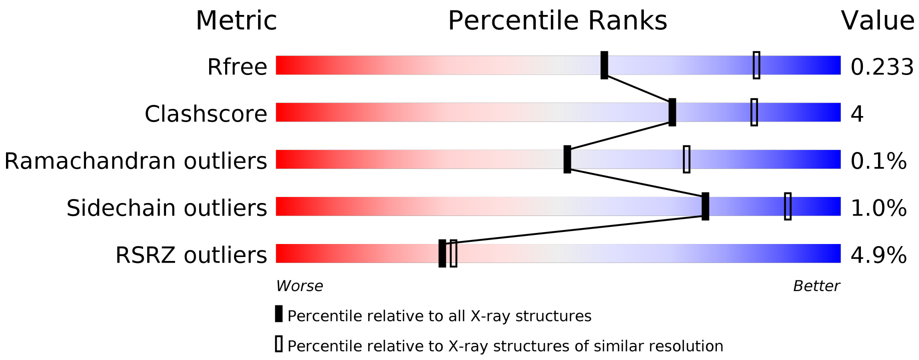

Metric	Whole archive (#Entries)	Similar resolution (#Entries, resolution range(Å))
R_{free}	130704	4661 (2.50-2.50)
Clashscore	141614	5346 (2.50-2.50)
Ramachandran outliers	138981	5231 (2.50-2.50)
Sidechain outliers	138945	5233 (2.50-2.50)
RSRZ outliers	127900	4559 (2.50-2.50)

The table below summarises the geometric issues observed across the polymeric chains and their fit to the electron density. The red, orange, yellow and green segments on the lower bar indicate the fraction of residues that contain outliers for >=3, 2, 1 and 0 types of geometric quality criteria respectively. A grey segment represents the fraction of residues that are not modelled. The numeric value for each fraction is indicated below the corresponding segment, with a dot representing fractions <=5% The upper red bar (where present) indicates the fraction of residues that have poor fit to the electron density. The numeric value is given above the bar.

Mol	Chain	Length	Quality of chain
1	A	336	% / 85% 8% 7%
1	B	336	83% 10% 7%
1	C	336	2% / 79% 11% • 9%
1	D	336	2% / 85% 8% 7%
1	E	336	84% 8% • 8%
1	F	336	22% / 78% • 20%

2 Entry composition (i)

There are 3 unique types of molecules in this entry. The entry contains 14652 atoms, of which 0 are hydrogens and 0 are deuteriums.

In the tables below, the ZeroOcc column contains the number of atoms modelled with zero occupancy, the AltConf column contains the number of residues with at least one atom in alternate conformation and the Trace column contains the number of residues modelled with at most 2 atoms.

- Molecule 1 is a protein called protein arginine N-methyltransferase.

Mol	Chain	Residues	Atoms					ZeroOcc	AltConf	Trace
1	A	311	Total<br>2492	C<br>1603	N<br>405	O<br>471	S<br>13	0	3	0
1	B	312	Total<br>2504	C<br>1618	N<br>406	O<br>468	S<br>12	0	7	0
1	C	306	Total<br>2356	C<br>1524	N<br>370	O<br>449	S<br>13	0	2	0
1	D	311	Total<br>2422	C<br>1555	N<br>389	O<br>466	S<br>12	0	1	0
1	E	309	Total<br>2487	C<br>1601	N<br>405	O<br>467	S<br>14	0	3	0
1	F	270	Total<br>1861	C<br>1168	N<br>310	O<br>373	S<br>10	0	0	0

- Molecule 2 is 1,2-ETHANEDIOL (three-letter code: EDO) (formula: $C_2H_6O_2$).

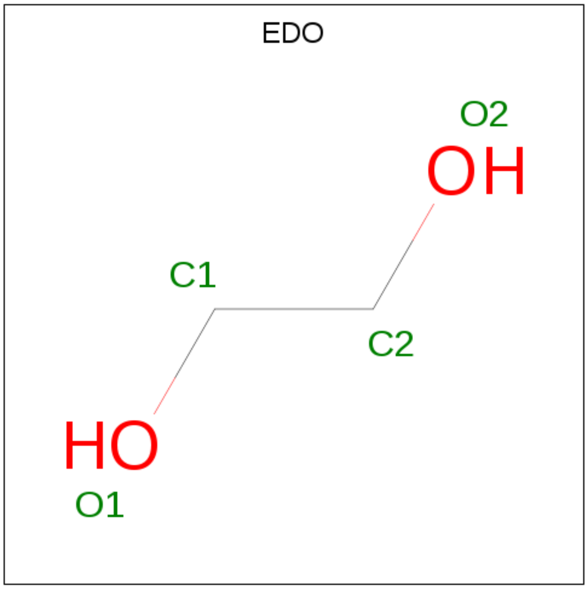

Mol	Chain	Residues	Atoms	ZeroOcc	AltConf
2	A	1	Total C O<br>4 2 2	0	0
2	A	1	Total C O<br>4 2 2	0	0
2	A	1	Total C O<br>4 2 2	0	0
2	A	1	Total C O<br>4 2 2	0	0
2	A	1	Total C O<br>4 2 2	0	0
2	B	1	Total C O<br>4 2 2	0	0
2	B	1	Total C O<br>4 2 2	0	0
2	B	1	Total C O<br>4 2 2	0	0
2	B	1	Total C O<br>4 2 2	0	0
2	B	1	Total C O<br>4 2 2	0	0
2	C	1	Total C O<br>4 2 2	0	0
2	E	1	Total C O<br>4 2 2	0	0
2	E	1	Total C O<br>4 2 2	0	0
2	E	1	Total C O<br>4 2 2	0	0
2	E	1	Total C O<br>4 2 2	0	0
2	E	1	Total C O<br>4 2 2	0	0
2	E	1	Total C O<br>4 2 2	0	0
2	E	1	Total C O<br>4 2 2	0	0

- Molecule 3 is water.

Mol	Chain	Residues	Atoms	ZeroOcc	AltConf
3	A	124	Total O<br>124 124	0	0
3	B	123	Total O<br>124 124	0	1

Continued on next page...

Continued from previous page...

Mol	Chain	Residues	Atoms	ZeroOcc	AltConf
3	C	32	Total O<br>32 32	0	0
3	D	56	Total O<br>56 56	0	0
3	E	109	Total O<br>109 109	0	0
3	F	13	Total O<br>13 13	0	0

3 Residue-property plots

These plots are drawn for all protein, RNA, DNA and oligosaccharide chains in the entry. The first graphic for a chain summarises the proportions of the various outlier classes displayed in the second graphic. The second graphic shows the sequence view annotated by issues in geometry and electron density. Residues are color-coded according to the number of geometric quality criteria for which they contain at least one outlier: green = 0, yellow = 1, orange = 2 and red = 3 or more. A red dot above a residue indicates a poor fit to the electron density (RSRZ > 2). Stretches of 2 or more consecutive residues without any outlier are shown as a green connector. Residues present in the sample, but not in the model, are shown in grey.

- Molecule 1: protein arginine N-methyltransferase

Chain A:

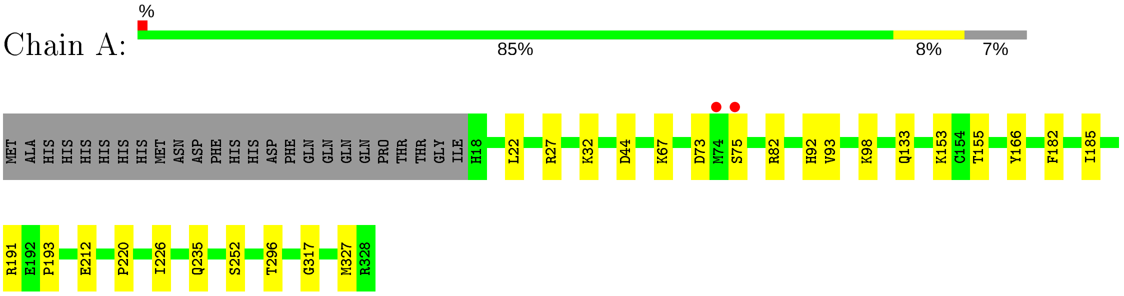

- Molecule 1: protein arginine N-methyltransferase

Chain B:

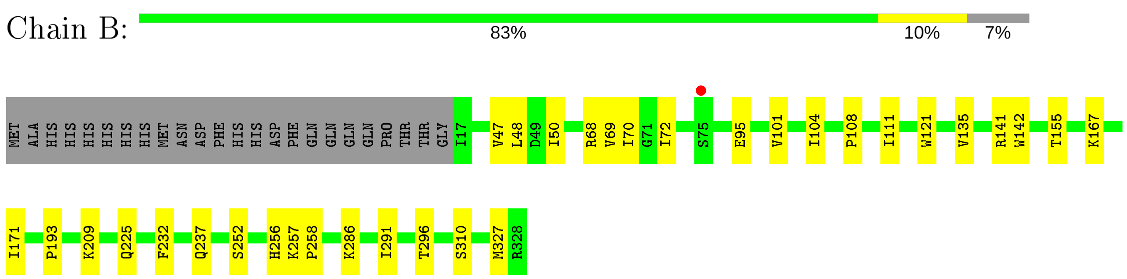

- Molecule 1: protein arginine N-methyltransferase

Chain C:

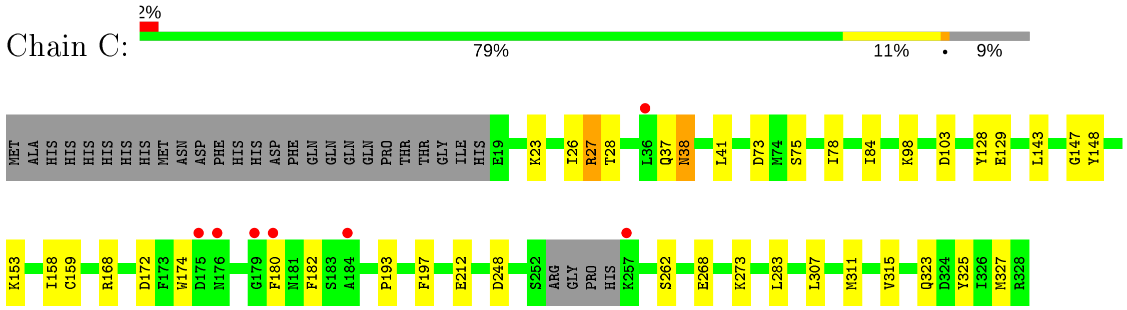

- Molecule 1: protein arginine N-methyltransferase

Chain D:

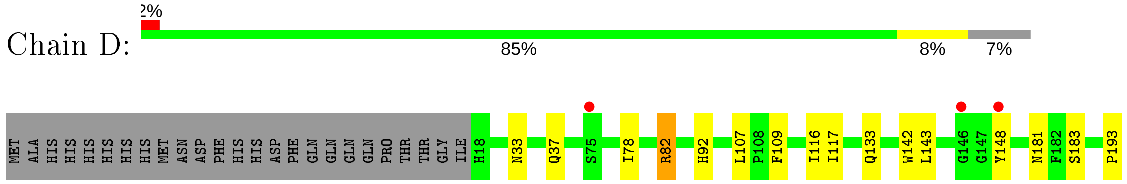

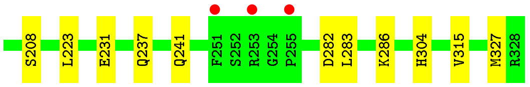

• Molecule 1: protein arginine N-methyltransferase

Chain E:

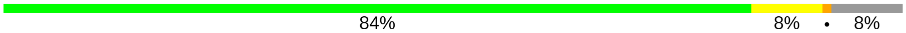

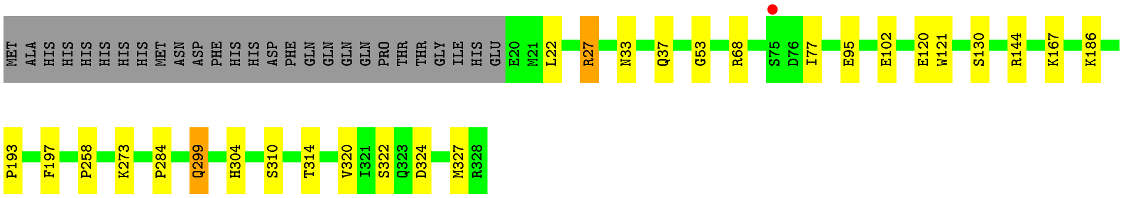

• Molecule 1: protein arginine N-methyltransferase

Chain F:

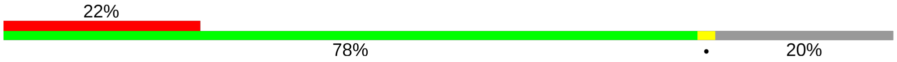

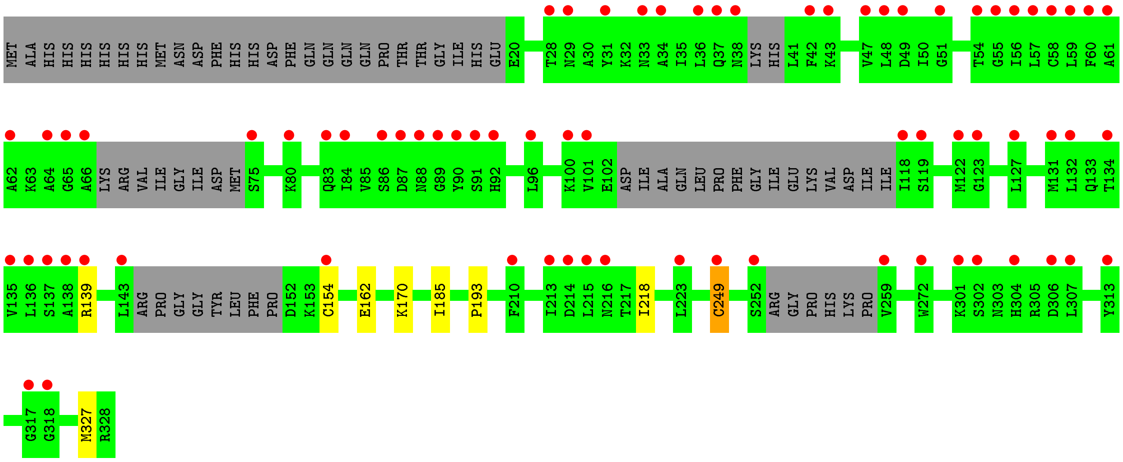

4 Data and refinement statistics (i)

Property	Value	Source
Space group	P 21 21 2	Depositor
Cell constants<br>a, b, c, α, β, γ	249.63Å 101.46Å 105.89Å<br>90.00° 90.00° 90.00°	Depositor
Resolution (Å)	47.00 – 2.50<br>47.51 – 2.50	Depositor<br>EDS
% Data completeness<br>(in resolution range)	99.8 (47.00-2.50)<br>99.9 (47.51-2.50)	Depositor<br>EDS
R_{merge}	0.08	Depositor
R_{sym}	(Not available)	Depositor
$< I/\sigma(I) >$ [1]	3.12 (at 2.51Å)	Xtriage
Refinement program	PHENIX	Depositor
R, R_{free}	0.190 , 0.233<br>0.190 , 0.233	Depositor<br>DCC
R_{free} test set	2168 reflections (2.31%)	wwPDB-VP
Wilson B-factor (Å^2)	40.8	Xtriage
Anisotropy	0.567	Xtriage
Bulk solvent k_{sol}(e/Å^3), B_{sol}(Å^2)	0.32 , 54.7	EDS
L-test for twinning[2]	$< \|L\| >$ = 0.49, $< L^2 >$ = 0.33	Xtriage
Estimated twinning fraction	0.000 for -h,l,k	Xtriage
F_o,F_c correlation	0.94	EDS
Total number of atoms	14652	wwPDB-VP
Average B, all atoms (Å^2)	54.0	wwPDB-VP

Xtriage's analysis on translational NCS is as follows: *The largest off-origin peak in the Patterson function is 17.66% of the height of the origin peak. No significant pseudotranslation is detected.*

[1] Intensities estimated from amplitudes.

[2] Theoretical values of $< |L| >$, $< L^2 >$ for acentric reflections are 0.5, 0.333 respectively for untwinned datasets, and 0.375, 0.2 for perfectly twinned datasets.

5 Model quality (i)

5.1 Standard geometry (i)

Bond lengths and bond angles in the following residue types are not validated in this section: EDO

The Z score for a bond length (or angle) is the number of standard deviations the observed value is removed from the expected value. A bond length (or angle) with $|Z| > 5$ is considered an outlier worth inspection. RMSZ is the root-mean-square of all Z scores of the bond lengths (or angles).

Mol	Chain	Bond lengths		Bond angles	
		RMSZ	#|Z| >5	RMSZ	#|Z| >5
1	A	0.47	0/2556	0.61	0/3461
1	B	0.48	0/2582	0.60	0/3501
1	C	0.36	0/2412	0.59	1/3278 (0.0%)
1	D	0.36	0/2478	0.54	0/3367
1	E	0.49	0/2551	0.62	1/3449 (0.0%)
1	F	0.39	1/1894 (0.1%)	0.57	1/2583 (0.0%)
All	All	0.43	1/14473 (0.0%)	0.59	3/19639 (0.0%)

All (1) bond length outliers are listed below:

Mol	Chain	Res	Type	Atoms	Z	Observed(Å)	Ideal(Å)
1	F	249	CYS	CB-SG	-7.05	1.70	1.82

All (3) bond angle outliers are listed below:

Mol	Chain	Res	Type	Atoms	Z	Observed(^{o})	Ideal(^{o})
1	C	307	LEU	CB-CG-CD2	-11.13	92.08	111.00
1	E	299	GLN	CB-CA-C	-8.60	93.20	110.40
1	F	249	CYS	CA-CB-SG	7.82	128.07	114.00

There are no chirality outliers.

There are no planarity outliers.

5.2 Too-close contacts (i)

In the following table, the Non-H and H(model) columns list the number of non-hydrogen atoms and hydrogen atoms in the chain respectively. The H(added) column lists the number of hydrogen atoms added and optimized by MolProbity. The Clashes column lists the number of clashes within the asymmetric unit, whereas Symm-Clashes lists symmetry related clashes.

Mol	Chain	Non-H	H(model)	H(added)	Clashes	Symm-Clashes
1	A	2492	0	2437	16	0
1	B	2504	0	2455	23	0
1	C	2356	0	2238	24	0
1	D	2422	0	2298	16	0
1	E	2487	0	2457	15	0
1	F	1861	0	1447	7	0
2	A	20	0	29	5	0
2	B	20	0	30	0	0
2	C	4	0	6	0	0
2	E	28	0	42	1	0
3	A	124	0	0	0	0
3	B	124	0	0	1	0
3	C	32	0	0	0	0
3	D	56	0	0	0	0
3	E	109	0	0	1	0
3	F	13	0	0	2	0
All	All	14652	0	13439	99	0

The all-atom clashscore is defined as the number of clashes found per 1000 atoms (including hydrogen atoms). The all-atom clashscore for this structure is 4.

All (99) close contacts within the same asymmetric unit are listed below, sorted by their clash magnitude.

Atom-1	Atom-2	Interatomic distance (Å)	Clash overlap (Å)
1:F:154:CYS:HA	1:F:249:CYS:HB3	1.61	0.82
1:D:107:LEU:HD11	1:D:142:TRP:CE2	2.22	0.75
1:A:235:GLN:HG2	2:A:405:EDO:H12	1.69	0.73
1:B:68[B]:ARG:NH1	1:B:95:GLU:HB2	2.05	0.71
1:B:104:ILE:O	1:B:141:ARG:NH2	2.19	0.70
1:C:75:SER:HA	1:C:98:LYS:HD3	1.74	0.70
1:F:170:LYS:NZ	3:F:401:HOH:O	2.26	0.69
1:A:193:PRO:HG2	1:A:327:MET:HG3	1.80	0.64
1:C:27:ARG:NH1	1:C:28:THR:OG1	2.31	0.63
1:D:107:LEU:CD1	1:D:142:TRP:CE2	2.82	0.62
1:D:116:ILE:HG12	1:D:148:TYR:HB2	1.82	0.61
1:C:193:PRO:HG2	1:C:327:MET:HG3	1.82	0.60
1:E:53:GLY:HA2	1:E:77:ILE:HD13	1.83	0.60
1:A:75:SER:HA	1:A:98:LYS:HG2	1.83	0.58
1:E:193:PRO:HG2	1:E:327:MET:HG3	1.85	0.58
1:D:92:HIS:H	1:D:92:HIS:CD2	2.20	0.57
1:D:193:PRO:HG2	1:D:327:MET:HG3	1.85	0.57
1:C:37:GLN:C	1:C:38:ASN:HD22	2.07	0.57

Continued on next page...

Continued from previous page...

Atom-1	Atom-2	Interatomic distance (Å)	Clash overlap (Å)
1:D:107:LEU:HD11	1:D:142:TRP:CZ2	2.40	0.56
1:A:182:PHE:HD2	1:A:185:ILE:HD12	1.71	0.56
1:B:68[A]:ARG:HD3	1:B:111:ILE:HD11	1.89	0.55
1:A:166:TYR:CG	2:A:404:EDO:H21	2.41	0.55
1:B:48:LEU:HD21	1:B:50[B]:ILE:HD11	1.89	0.55
1:E:284:PRO:HD2	2:E:407:EDO:H12	1.89	0.54
1:B:101:VAL:HA	1:B:104:ILE:HD12	1.88	0.54
1:E:299:GLN:OE1	1:E:304:HIS:ND1	2.40	0.54
1:B:47[A]:VAL:HG22	1:B:69:VAL:HG22	1.92	0.52
1:B:141:ARG:HG2	1:B:142:TRP:CE2	2.45	0.52
1:C:73:ASP:H	1:C:78:ILE:HD11	1.73	0.52
1:E:68:ARG:NH1	1:E:95:GLU:HB2	2.26	0.51
1:A:22:LEU:HD23	1:A:27[B]:ARG:HH22	1.76	0.51
1:D:107:LEU:CD1	1:D:142:TRP:CZ2	2.94	0.51
1:F:139:ARG:NH1	1:F:218:ILE:O	2.31	0.51
1:B:68[B]:ARG:HH12	1:B:95:GLU:HB2	1.76	0.50
1:D:33:ASN:O	1:D:37:GLN:HB2	2.12	0.50
1:C:323:GLN:HB3	1:C:325:TYR:CE1	2.47	0.49
1:C:153:LYS:HE2	1:C:212:GLU:OE1	2.12	0.49
1:D:133:GLN:NE2	1:D:223:LEU:HD12	2.28	0.49
1:F:193:PRO:HG2	1:F:327:MET:HG3	1.94	0.49
1:B:232:PHE:HE1	1:B:291[A]:ILE:HD12	1.78	0.49
1:B:237:GLN:HG3	1:B:286:LYS:HD3	1.95	0.48
1:C:174:TRP:O	1:C:182:PHE:HB2	2.12	0.48
1:D:181:ASN:HD21	1:D:183:SER:HB3	1.76	0.48
1:A:44:ASP:HA	1:A:67:LYS:HB3	1.95	0.48
1:E:68:ARG:HH11	1:E:95:GLU:HB2	1.79	0.48
1:B:48:LEU:HD11	1:B:72:ILE:HD11	1.95	0.48
1:C:73:ASP:N	1:C:78:ILE:HD11	2.30	0.46
1:B:256[B]:HIS:CG	1:B:257:LYS:H	2.33	0.46
1:A:133:GLN:OE1	1:A:220:PRO:HB3	2.15	0.46
1:D:117:ILE:HG13	1:D:143:LEU:HD13	1.97	0.46
1:C:143:LEU:HD11	1:C:147:GLY:HA3	1.96	0.46
1:D:283:LEU:HD11	1:D:315:VAL:HG21	1.99	0.45
1:D:241:GLN:HG3	1:D:282:ASP:OD1	2.15	0.45
1:B:108:PRO:O	1:B:111:ILE:HD12	2.17	0.45
1:C:26:ILE:HD11	1:C:268:GLU:HB3	1.98	0.45
1:E:22:LEU:HD23	1:E:27[A]:ARG:HH12	1.82	0.45
1:C:84:ILE:HD11	1:F:185:ILE:HD11	1.98	0.45
1:A:32:LYS:HB3	1:A:32:LYS:HE3	1.74	0.45
1:E:197:PHE:CZ	1:E:273:LYS:HG2	2.52	0.45

Continued on next page...

Continued from previous page...

Atom-1	Atom-2	Interatomic distance (Å)	Clash overlap (Å)
1:C:168:ARG:HA	1:C:172:ASP:HB2	1.98	0.44
1:C:128:TYR:CD1	1:C:327:MET:HB3	2.53	0.44
1:C:158[A]:ILE:HG13	1:C:159:CYS:N	2.33	0.44
1:C:283:LEU:HD11	1:C:315:VAL:HG21	1.99	0.44
1:A:212:GLU:HB2	2:A:403:EDO:O2	2.18	0.44
1:B:121:TRP:HB3	1:B:135:VAL:HG21	1.99	0.44
1:E:27[A]:ARG:HE	1:E:120:GLU:CD	2.22	0.43
1:B:258:PRO:HA	3:B:510:HOH:O	2.17	0.43
1:C:180:PHE:CD1	1:C:180:PHE:N	2.84	0.43
1:B:167:LYS:HA	1:B:171:ILE:HD12	2.00	0.43
1:D:208:SER:HB2	1:D:231:GLU:O	2.19	0.43
1:C:197:PHE:CZ	1:C:273:LYS:HG2	2.54	0.43
1:E:102:GLU:H	1:E:102:GLU:CD	2.21	0.43
1:C:311:MET:HB2	1:C:323:GLN:HB2	2.00	0.43
1:C:41:LEU:HD11	1:C:148:TYR:CE1	2.53	0.43
1:D:78:ILE:O	1:D:82:ARG:HB2	2.18	0.43
1:B:155:THR:HG21	1:B:209:LYS:HE2	2.02	0.42
1:E:33:ASN:O	1:E:37:GLN:HB2	2.19	0.42
1:A:155:THR:OG1	2:A:403:EDO:H12	2.20	0.42
1:B:193:PRO:HG2	1:B:327:MET:HG3	2.01	0.42
1:C:23:LYS:HB3	1:C:23:LYS:HE2	1.82	0.42
1:E:310[B]:SER:HB2	1:E:324:ASP:OD1	2.20	0.42
1:B:256[B]:HIS:CG	1:B:257:LYS:N	2.88	0.41
1:F:162:GLU:HB3	3:F:405:HOH:O	2.20	0.41
1:A:82:ARG:HH12	2:A:401:EDO:H21	1.84	0.41
1:B:296:THR:OG1	1:B:310:SER:HB2	2.19	0.41
1:C:27:ARG:HG3	1:C:28:THR:N	2.34	0.41
1:E:314:THR:HG22	1:E:320:VAL:HG12	2.01	0.41
1:F:154:CYS:CA	1:F:249:CYS:HB3	2.34	0.41
1:D:237:GLN:HG3	1:D:286:LYS:HD3	2.02	0.41
1:A:153:LYS:NZ	1:A:252:SER:OG	2.49	0.41
1:A:226:ILE:O	1:A:296:THR:HA	2.21	0.41
1:C:248:ASP:OD1	1:C:262:SER:OG	2.36	0.41
1:A:317:GLY:HA2	1:B:68[B]:ARG:NH2	2.36	0.41
1:B:70:ILE:HD11	1:B:111:ILE:HD13	2.02	0.41
1:B:68[A]:ARG:CD	1:B:111:ILE:HD11	2.50	0.40
1:C:128:TYR:CE2	1:C:129:GLU:HG3	2.56	0.40
1:A:92:HIS:CD2	1:A:93:VAL:HG23	2.56	0.40
1:E:121:TRP:O	1:E:130:SER:HB2	2.20	0.40
1:E:322:SER:HB2	3:E:544:HOH:O	2.21	0.40

There are no symmetry-related clashes.

5.3 Torsion angles (i)

5.3.1 Protein backbone (i)

In the following table, the Percentiles column shows the percent Ramachandran outliers of the chain as a percentile score with respect to all X-ray entries followed by that with respect to entries of similar resolution.

The Analysed column shows the number of residues for which the backbone conformation was analysed, and the total number of residues.

Mol	Chain	Analysed	Favoured	Allowed	Outliers	Percentiles	
1	A	312/336 (93%)	306 (98%)	6 (2%)	0	100	100
1	B	317/336 (94%)	308 (97%)	9 (3%)	0	100	100
1	C	304/336 (90%)	297 (98%)	7 (2%)	0	100	100
1	D	310/336 (92%)	303 (98%)	7 (2%)	0	100	100
1	E	310/336 (92%)	304 (98%)	5 (2%)	1 (0%)	41	61
1	F	258/336 (77%)	253 (98%)	5 (2%)	0	100	100
All	All	1811/2016 (90%)	1771 (98%)	39 (2%)	1 (0%)	51	73

All (1) Ramachandran outliers are listed below:

Mol	Chain	Res	Type
1	E	258	PRO

5.3.2 Protein sidechains (i)

In the following table, the Percentiles column shows the percent sidechain outliers of the chain as a percentile score with respect to all X-ray entries followed by that with respect to entries of similar resolution.

The Analysed column shows the number of residues for which the sidechain conformation was analysed, and the total number of residues.

Mol	Chain	Analysed	Rotameric	Outliers	Percentiles	
1	A	271/299 (91%)	269 (99%)	2 (1%)	84	94
1	B	272/299 (91%)	269 (99%)	3 (1%)	73	89
1	C	245/299 (82%)	242 (99%)	3 (1%)	71	88
1	D	255/299 (85%)	252 (99%)	3 (1%)	71	88
1	E	273/299 (91%)	268 (98%)	5 (2%)	59	81
1	F	148/299 (50%)	148 (100%)	0	100	100

Continued on next page...

Continued from previous page...

Mol	Chain	Analysed	Rotameric	Outliers	Percentiles	
All	All	1464/1794 (82%)	1448 (99%)	16 (1%)	76	89

All (16) residues with a non-rotameric sidechain are listed below:

Mol	Chain	Res	Type
1	A	73	ASP
1	A	191	ARG
1	B	225	GLN
1	B	252[A]	SER
1	B	252[B]	SER
1	C	27	ARG
1	C	38	ASN
1	C	103	ASP
1	D	82	ARG
1	D	109	PHE
1	D	304	HIS
1	E	27[A]	ARG
1	E	27[B]	ARG
1	E	144	ARG
1	E	167	LYS
1	E	186	LYS

Some sidechains can be flipped to improve hydrogen bonding and reduce clashes. All (5) such sidechains are listed below:

Mol	Chain	Res	Type
1	C	38	ASN
1	D	92	HIS
1	D	181	ASN
1	E	237	GLN
1	F	271	HIS

5.3.3 RNA ⓘ

There are no RNA molecules in this entry.

5.4 Non-standard residues in protein, DNA, RNA chains ⓘ

There are no non-standard protein/DNA/RNA residues in this entry.

5.5 Carbohydrates (i)

There are no monosaccharides in this entry.

5.6 Ligand geometry (i)

18 ligands are modelled in this entry.

In the following table, the Counts columns list the number of bonds (or angles) for which Mogul statistics could be retrieved, the number of bonds (or angles) that are observed in the model and the number of bonds (or angles) that are defined in the Chemical Component Dictionary. The Link column lists molecule types, if any, to which the group is linked. The Z score for a bond length (or angle) is the number of standard deviations the observed value is removed from the expected value. A bond length (or angle) with $|Z| > 2$ is considered an outlier worth inspection. RMSZ is the root-mean-square of all Z scores of the bond lengths (or angles).

Mol	Type	Chain	Res	Link	Bond lengths			Bond angles		
					Counts	RMSZ	$\#\|Z\| > 2$	Counts	RMSZ	$\#\|Z\| > 2$
2	EDO	B	401	-	3,3,3	0.57	0	2,2,2	0.13	0
2	EDO	E	403	-	3,3,3	0.44	0	2,2,2	0.55	0
2	EDO	B	405	-	3,3,3	0.45	0	2,2,2	0.82	0
2	EDO	B	402	-	3,3,3	0.47	0	2,2,2	0.43	0
2	EDO	A	402	-	3,3,3	0.46	0	2,2,2	0.52	0
2	EDO	B	403	-	3,3,3	0.59	0	2,2,2	0.15	0
2	EDO	E	402	-	3,3,3	0.43	0	2,2,2	0.54	0
2	EDO	C	401	-	3,3,3	0.53	0	2,2,2	0.25	0
2	EDO	E	405	-	3,3,3	0.53	0	2,2,2	0.26	0
2	EDO	E	404	-	3,3,3	0.48	0	2,2,2	0.23	0
2	EDO	A	405	-	3,3,3	0.55	0	2,2,2	0.25	0
2	EDO	E	407	-	3,3,3	0.54	0	2,2,2	0.08	0
2	EDO	E	406	-	3,3,3	0.43	0	2,2,2	0.65	0
2	EDO	E	401	-	3,3,3	0.53	0	2,2,2	0.29	0
2	EDO	B	404	-	3,3,3	0.36	0	2,2,2	0.68	0
2	EDO	A	401	-	3,3,3	0.59	0	2,2,2	0.21	0
2	EDO	A	403	-	3,3,3	0.54	0	2,2,2	0.12	0
2	EDO	A	404	-	3,3,3	1.01	0	2,2,2	0.23	0

In the following table, the Chirals column lists the number of chiral outliers, the number of chiral centers analysed, the number of these observed in the model and the number defined in the Chemical Component Dictionary. Similar counts are reported in the Torsion and Rings columns. '-' means no outliers of that kind were identified.

Mol	Type	Chain	Res	Link	Chirals	Torsions	Rings
2	EDO	B	401	-	-	1/1/1/1	-
2	EDO	E	403	-	-	0/1/1/1	-

Continued on next page...

Continued from previous page...

Mol	Type	Chain	Res	Link	Chirals	Torsions	Rings
2	EDO	B	405	-	-	1/1/1/1	-
2	EDO	B	402	-	-	0/1/1/1	-
2	EDO	A	402	-	-	1/1/1/1	-
2	EDO	B	403	-	-	1/1/1/1	-
2	EDO	E	402	-	-	1/1/1/1	-
2	EDO	C	401	-	-	1/1/1/1	-
2	EDO	E	405	-	-	0/1/1/1	-
2	EDO	E	404	-	-	0/1/1/1	-
2	EDO	A	405	-	-	1/1/1/1	-
2	EDO	E	407	-	-	0/1/1/1	-
2	EDO	E	406	-	-	1/1/1/1	-
2	EDO	E	401	-	-	0/1/1/1	-
2	EDO	B	404	-	-	0/1/1/1	-
2	EDO	A	401	-	-	1/1/1/1	-
2	EDO	A	403	-	-	1/1/1/1	-
2	EDO	A	404	-	-	0/1/1/1	-

There are no bond length outliers.

There are no bond angle outliers.

There are no chirality outliers.

All (10) torsion outliers are listed below:

Mol	Chain	Res	Type	Atoms
2	B	405	EDO	O1-C1-C2-O2
2	B	403	EDO	O1-C1-C2-O2
2	A	405	EDO	O1-C1-C2-O2
2	C	401	EDO	O1-C1-C2-O2
2	E	406	EDO	O1-C1-C2-O2
2	B	401	EDO	O1-C1-C2-O2
2	A	403	EDO	O1-C1-C2-O2
2	A	402	EDO	O1-C1-C2-O2
2	E	402	EDO	O1-C1-C2-O2
2	A	401	EDO	O1-C1-C2-O2

There are no ring outliers.

5 monomers are involved in 6 short contacts:

Mol	Chain	Res	Type	Clashes	Symm-Clashes
2	A	405	EDO	1	0
2	E	407	EDO	1	0

Continued on next page...

Continued from previous page...

Mol	Chain	Res	Type	Clashes	Symm-Clashes
2	A	401	EDO	1	0
2	A	403	EDO	2	0
2	A	404	EDO	1	0

5.7 Other polymers (i)

There are no such residues in this entry.

5.8 Polymer linkage issues (i)

There are no chain breaks in this entry.

6 Fit of model and data

6.1 Protein, DNA and RNA chains

In the following table, the column labelled '#RSRZ> 2' contains the number (and percentage) of RSRZ outliers, followed by percent RSRZ outliers for the chain as percentile scores relative to all X-ray entries and entries of similar resolution. The OWAB column contains the minimum, median, 95th percentile and maximum values of the occupancy-weighted average B-factor per residue. The column labelled 'Q< 0.9' lists the number of (and percentage) of residues with an average occupancy less than 0.9.

Mol	Chain	Analysed	<RSRZ>	#RSRZ>2			OWAB(Å^2)	Q<0.9
1	A	311/336 (92%)	-0.27	2 (0%)	89	90	24, 38, 62, 87	0
1	B	312/336 (92%)	-0.23	1 (0%)	94	94	24, 39, 64, 107	0
1	C	306/336 (91%)	-0.06	7 (2%)	60	63	41, 63, 96, 120	0
1	D	311/336 (92%)	-0.09	6 (1%)	66	69	32, 61, 89, 120	0
1	E	309/336 (91%)	-0.25	1 (0%)	94	94	24, 39, 65, 79	0
1	F	270/336 (80%)	1.18	73 (27%)	0	0	44, 94, 129, 148	0
All	All	1819/2016 (90%)	0.02	90 (4%)	29	31	24, 51, 107, 148	0

All (90) RSRZ outliers are listed below:

Mol	Chain	Res	Type	RSRZ
1	F	135	VAL	7.1
1	F	60	PHE	6.8
1	F	88	ASN	6.8
1	F	84	ILE	6.6
1	F	137	SER	6.1
1	F	138	ALA	5.9
1	F	55	GLY	5.7
1	F	49	ASP	5.3
1	F	31	TYR	5.2
1	F	87	ASP	5.1
1	F	56	ILE	5.0
1	F	65	GLY	4.8
1	C	180	PHE	4.6
1	F	216	ASN	4.5
1	F	54	THR	4.4
1	F	101	VAL	4.2
1	F	37	GLN	4.2
1	F	259	VAL	4.2
1	F	86	SER	4.0

Continued on next page...

Continued from previous page...

Mol	Chain	Res	Type	RSRZ
1	F	89	GLY	4.0
1	F	91	SER	4.0
1	F	36	LEU	4.0
1	F	62	ALA	3.9
1	F	249	CYS	3.9
1	F	118	ILE	3.8
1	F	317	GLY	3.8
1	F	119	SER	3.8
1	C	176	ASN	3.6
1	B	75	SER	3.6
1	F	90	TYR	3.6
1	F	122	MET	3.5
1	F	48	LEU	3.5
1	F	92	HIS	3.5
1	F	47	VAL	3.4
1	F	307	LEU	3.3
1	F	96	LEU	3.2
1	F	132	LEU	3.2
1	D	255	PRO	3.2
1	F	80	LYS	3.2
1	F	143	LEU	3.0
1	F	131	MET	3.0
1	F	61	ALA	3.0
1	F	215	LEU	2.9
1	F	252	SER	2.9
1	F	304	HIS	2.9
1	D	75	SER	2.9
1	F	64	ALA	2.8
1	F	139	ARG	2.8
1	F	29	ASN	2.7
1	F	154	CYS	2.7
1	F	134	THR	2.7
1	F	57	LEU	2.7
1	F	136	LEU	2.7
1	E	75	SER	2.7
1	F	58	CYS	2.6
1	F	33	ASN	2.6
1	F	302	SER	2.6
1	F	223	LEU	2.5
1	F	83	GLN	2.5
1	C	36	LEU	2.5
1	F	42	PHE	2.5

Continued on next page...

Continued from previous page...

Mol	Chain	Res	Type	RSRZ
1	F	59	LEU	2.5
1	F	301	LYS	2.5
1	F	38	ASN	2.5
1	F	123	GLY	2.5
1	D	251	PHE	2.4
1	F	214	ASP	2.4
1	F	43	LYS	2.4
1	F	213	ILE	2.4
1	C	175	ASP	2.4
1	D	253	ARG	2.4
1	C	257	LYS	2.3
1	F	28	THR	2.3
1	F	210	PHE	2.3
1	A	74	MET	2.3
1	C	184	ALA	2.2
1	C	179	GLY	2.2
1	A	75	SER	2.2
1	F	272	TRP	2.2
1	F	51	GLY	2.2
1	F	34	ALA	2.2
1	D	146	GLY	2.2
1	F	318	GLY	2.2
1	F	75	SER	2.2
1	D	148	TYR	2.1
1	F	127	LEU	2.1
1	F	306	ASP	2.1
1	F	100	LYS	2.1
1	F	313	TYR	2.0
1	F	66	ALA	2.0

6.2 Non-standard residues in protein, DNA, RNA chains ⓘ

There are no non-standard protein/DNA/RNA residues in this entry.

6.3 Carbohydrates ⓘ

There are no monosaccharides in this entry.

6.4 Ligands (i)

In the following table, the Atoms column lists the number of modelled atoms in the group and the number defined in the chemical component dictionary. The B-factors column lists the minimum, median, 95th percentile and maximum values of B factors of atoms in the group. The column labelled 'Q< 0.9' lists the number of atoms with occupancy less than 0.9.

Mol	Type	Chain	Res	Atoms	RSCC	RSR	B-factors(Å^2)	Q<0.9
2	EDO	C	401	4/4	0.60	0.23	76,78,79,80	0
2	EDO	E	401	4/4	0.67	0.16	76,81,82,83	0
2	EDO	A	403	4/4	0.68	0.26	71,74,75,77	0
2	EDO	A	405	4/4	0.69	0.27	67,71,73,74	0
2	EDO	A	401	4/4	0.78	0.15	76,77,78,81	0
2	EDO	B	403	4/4	0.83	0.24	59,61,62,63	0
2	EDO	A	404	4/4	0.83	0.21	30,36,36,37	0
2	EDO	E	404	4/4	0.85	0.22	71,72,73,73	0
2	EDO	E	407	4/4	0.87	0.20	66,67,67,70	0
2	EDO	B	401	4/4	0.89	0.18	54,61,61,66	0
2	EDO	E	402	4/4	0.89	0.29	72,73,75,75	0
2	EDO	B	405	4/4	0.89	0.17	65,68,71,76	0
2	EDO	A	402	4/4	0.89	0.22	50,54,57,63	0
2	EDO	B	404	4/4	0.89	0.26	65,68,68,72	0
2	EDO	E	405	4/4	0.89	0.18	66,75,81,81	0
2	EDO	E	406	4/4	0.90	0.22	64,64,69,72	0
2	EDO	B	402	4/4	0.91	0.14	74,76,78,79	0
2	EDO	E	403	4/4	0.92	0.16	74,76,76,78	0

6.5 Other polymers (i)

There are no such residues in this entry.
