# Supplementary material for: Naegleria fowleri: Protein structures to facilitate drug discovery for the deadly, pathogenic free-living amoeba
Source: PLoS One. 2021 Mar 24;16(3):e0241738. doi: 10.1371/journal.pone.0241738 (PMC7990177; doi:10.1371/journal.pone.0241738)
Supplement: S1 File — (ZIP) [file pone.0241738.s002.zip › 6nlx_full_validation.pdf]

Full wwPDB X-ray Structure Validation Report (i)

May 13, 2020 – 04:37 am BST

PDB ID	:	6NLX
Title	:	Crystal Structure of Glyceraldehyde-3-phosphate Dehydrogenase from Naegleria fowleri with bound NAD
Authors	:	Seattle Structural Genomics Center for Infectious Disease; Seattle Structural Genomics Center for Infectious Disease (SSGCID)
Deposited on	:	2019-01-09
Resolution	:	1.80 Å(reported)

This is a Full wwPDB X-ray Structure Validation Report for a publicly released PDB entry.

We welcome your comments at *validation@mail.wwpdb.org*
A user guide is available at
https://www.wwpdb.org/validation/2017/XrayValidationReportHelp
with specific help available everywhere you see the (i) symbol.

The following versions of software and data (see references (i)) were used in the production of this report:

MolProbity	:	4.02b-467
Mogul	:	1.8.5 (274361), CSD as541be (2020)
Xtriage (Phenix)	:	1.13
EDS	:	2.11
buster-report	:	1.1.7 (2018)
Percentile statistics	:	20191225.v01 (using entries in the PDB archive December 25th 2019)
Refmac	:	5.8.0158
CCP4	:	7.0.044 (Gargrove)
Ideal geometry (proteins)	:	Engh & Huber (2001)
Ideal geometry (DNA, RNA)	:	Parkinson et al. (1996)
Validation Pipeline (wwPDB-VP)	:	2.11

1 Overall quality at a glance (i)

The following experimental techniques were used to determine the structure:
X-RAY DIFFRACTION

The reported resolution of this entry is 1.80 Å.

Percentile scores (ranging between 0-100) for global validation metrics of the entry are shown in the following graphic. The table shows the number of entries on which the scores are based.

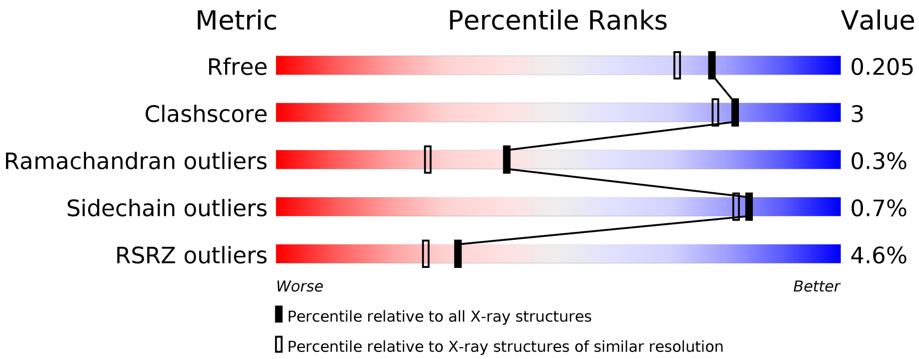

Metric	Whole archive (#Entries)	Similar resolution (#Entries, resolution range(Å))
R_{free}	130704	5950 (1.80-1.80)
Clashscore	141614	6793 (1.80-1.80)
Ramachandran outliers	138981	6697 (1.80-1.80)
Sidechain outliers	138945	6696 (1.80-1.80)
RSRZ outliers	127900	5850 (1.80-1.80)

The table below summarises the geometric issues observed across the polymeric chains and their fit to the electron density. The red, orange, yellow and green segments on the lower bar indicate the fraction of residues that contain outliers for >=3, 2, 1 and 0 types of geometric quality criteria respectively. A grey segment represents the fraction of residues that are not modelled. The numeric value for each fraction is indicated below the corresponding segment, with a dot representing fractions <=5% The upper red bar (where present) indicates the fraction of residues that have poor fit to the electron density. The numeric value is given above the bar.

Mol	Chain	Length	Quality of chain
1	A	341	9% / 91% 8% •
1	B	341	91% 6% • •
1	C	341	92% 6% •
1	D	341	9% / 92% 5% •

The following table lists non-polymeric compounds, carbohydrate monomers and non-standard residues in protein, DNA, RNA chains that are outliers for geometric or electron-density-fit crite-

ria:

Mol	Type	Chain	Res	Chirality	Geometry	Clashes	Electron density
2	EDO	A	402[A]	-	-	-	X
2	EDO	A	402[B]	-	-	-	X

2 Entry composition (i)

There are 4 unique types of molecules in this entry. The entry contains 11566 atoms, of which 0 are hydrogens and 0 are deuteriums.

In the tables below, the ZeroOcc column contains the number of atoms modelled with zero occupancy, the AltConf column contains the number of residues with at least one atom in alternate conformation and the Trace column contains the number of residues modelled with at most 2 atoms.

- Molecule 1 is a protein called Glyceraldehyde-3-phosphate Dehydrogenase.

Mol	Chain	Residues	Atoms					ZeroOcc	AltConf	Trace
1	A	337	Total 2576	C 1632	N 440	O 490	S 14	0	9	0
1	B	333	Total 2605	C 1655	N 439	O 494	S 17	0	9	0
1	C	335	Total 2616	C 1660	N 437	O 502	S 17	0	11	0
1	D	332	Total 2540	C 1613	N 428	O 483	S 16	0	7	0

- Molecule 2 is 1,2-ETHANEDIOL (three-letter code: EDO) (formula: $C_2H_6O_2$).

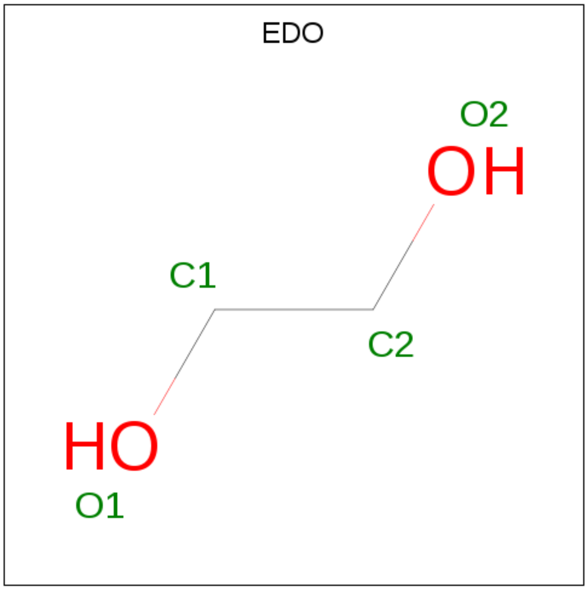

Mol	Chain	Residues	Atoms			ZeroOcc	AltConf
2	A	1	Total 4	C 2	O 2	0	0
2	A	1	Total 8	C 4	O 4	0	1

Continued on next page...

Continued from previous page...

Mol	Chain	Residues	Atoms	ZeroOcc	AltConf
2	A	1	Total C O<br>4 2 2	0	0
2	B	1	Total C O<br>4 2 2	0	0
2	B	1	Total C O<br>4 2 2	0	0
2	C	1	Total C O<br>4 2 2	0	0
2	C	1	Total C O<br>4 2 2	0	0
2	C	1	Total C O<br>8 4 4	0	1
2	C	1	Total C O<br>4 2 2	0	0
2	C	1	Total C O<br>4 2 2	0	0
2	D	1	Total C O<br>4 2 2	0	0
2	D	1	Total C O<br>4 2 2	0	0
2	D	1	Total C O<br>4 2 2	0	0

- Molecule 3 is NICOTINAMIDE-ADENINE-DINUCLEOTIDE (three-letter code: NAD) (formula: $C_{21}H_{27}N_{7}O_{14}P_{2}$).

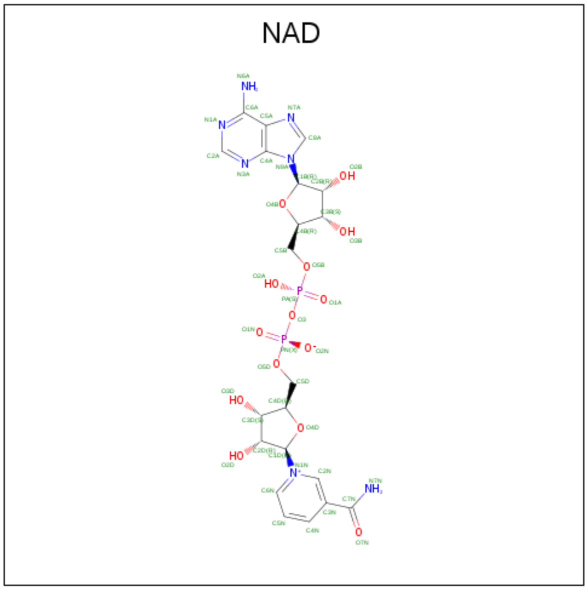

Mol	Chain	Residues	Atoms					ZeroOcc	AltConf
3	B	1	Total<br>44	C<br>21	N<br>7	O<br>14	P<br>2	0	0
3	C	1	Total<br>44	C<br>21	N<br>7	O<br>14	P<br>2	0	0
3	D	1	Total<br>44	C<br>21	N<br>7	O<br>14	P<br>2	0	0

- Molecule 4 is water.

Mol	Chain	Residues	Atoms		ZeroOcc	AltConf
4	A	210	Total<br>211	O<br>211	0	2
4	B	299	Total<br>303	O<br>303	0	4
4	C	337	Total<br>338	O<br>338	0	1
4	D	185	Total<br>185	O<br>185	0	0

3 Residue-property plots (i)

These plots are drawn for all protein, RNA and DNA chains in the entry. The first graphic for a chain summarises the proportions of the various outlier classes displayed in the second graphic. The second graphic shows the sequence view annotated by issues in geometry and electron density. Residues are color-coded according to the number of geometric quality criteria for which they contain at least one outlier: green = 0, yellow = 1, orange = 2 and red = 3 or more. A red dot above a residue indicates a poor fit to the electron density (RSRZ > 2). Stretches of 2 or more consecutive residues without any outlier are shown as a green connector. Residues present in the sample, but not in the model, are shown in grey.

- Molecule 1: Glyceraldehyde-3-phosphate Dehydrogenase

Chain A:

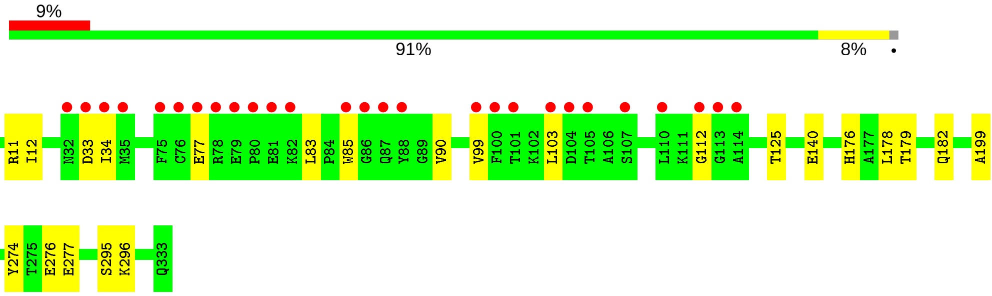

- Molecule 1: Glyceraldehyde-3-phosphate Dehydrogenase

Chain B:

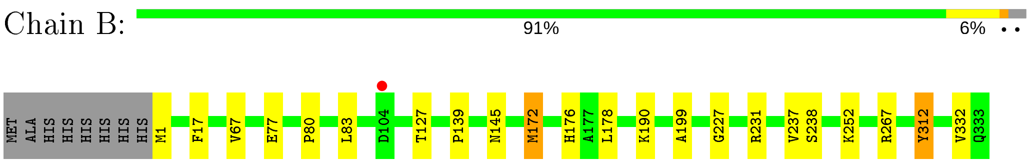

- Molecule 1: Glyceraldehyde-3-phosphate Dehydrogenase

Chain C:

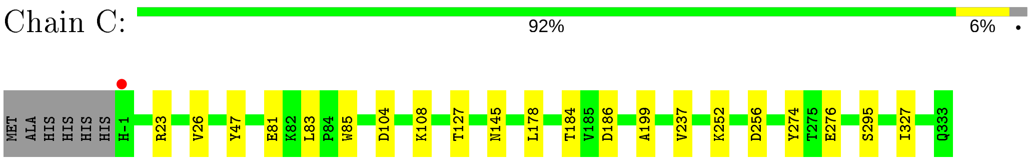

- Molecule 1: Glyceraldehyde-3-phosphate Dehydrogenase

Chain D:

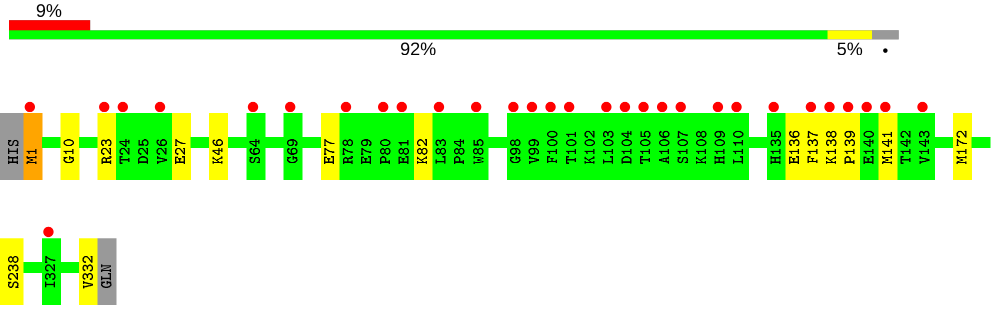

4 Data and refinement statistics

Property	Value	Source
Space group	P 21 21 21	Depositor
Cell constants<br>a, b, c, α, β, γ	73.01Å 119.94Å 162.90Å<br>90.00° 90.00° 90.00°	Depositor
Resolution (Å)	49.47 – 1.80<br>49.47 – 1.80	Depositor<br>EDS
% Data completeness<br>(in resolution range)	97.4 (49.47-1.80)<br>97.4 (49.47-1.80)	Depositor<br>EDS
R_{merge}	0.08	Depositor
R_{sym}	(Not available)	Depositor
$< I/\sigma(I) >$ [1]	2.89 (at 1.79Å)	Xtriage
Refinement program	PHENIX	Depositor
R, R_{free}	0.169 , 0.204<br>0.170 , 0.205	Depositor<br>DCC
R_{free} test set	2019 reflections (1.56%)	wwPDB-VP
Wilson B-factor (Å^2)	20.7	Xtriage
Anisotropy	0.284	Xtriage
Bulk solvent k_{sol}(e/Å^3), B_{sol}(Å^2)	0.32 , 50.8	EDS
L-test for twinning[2]	$< \|L\| > = 0.48$, $< L^2 > = 0.31$	Xtriage
Estimated twinning fraction	No twinning to report.	Xtriage
F_o,F_c correlation	0.96	EDS
Total number of atoms	11566	wwPDB-VP
Average B, all atoms (Å^2)	29.0	wwPDB-VP

Xtriage's analysis on translational NCS is as follows: *The largest off-origin peak in the Patterson function is 3.89% of the height of the origin peak. No significant pseudotranslation is detected.*

[1] Intensities estimated from amplitudes.

[2] Theoretical values of $< |L| >$, $< L^2 >$ for acentric reflections are 0.5, 0.333 respectively for untwinned datasets, and 0.375, 0.2 for perfectly twinned datasets.

5 Model quality (i)

5.1 Standard geometry (i)

Bond lengths and bond angles in the following residue types are not validated in this section: EDO, NAD

The Z score for a bond length (or angle) is the number of standard deviations the observed value is removed from the expected value. A bond length (or angle) with $|Z| > 5$ is considered an outlier worth inspection. RMSZ is the root-mean-square of all Z scores of the bond lengths (or angles).

Mol	Chain	Bond lengths		Bond angles	
		RMSZ	$\#|Z| > 5$	RMSZ	$\#|Z| > 5$
1	A	0.34	0/2656	0.56	0/3610
1	B	0.36	0/2683	0.57	0/3630
1	C	0.39	0/2701	0.60	1/3664 (0.0%)
1	D	0.33	0/2612	0.54	0/3549
All	All	0.36	0/10652	0.57	1/14453 (0.0%)

There are no bond length outliers.

All (1) bond angle outliers are listed below:

Mol	Chain	Res	Type	Atoms	Z	Observed(^{o})	Ideal(^{o})
1	C	186	ASP	CB-CG-OD1	6.12	123.81	118.30

There are no chirality outliers.

There are no planarity outliers.

5.2 Too-close contacts (i)

In the following table, the Non-H and H(model) columns list the number of non-hydrogen atoms and hydrogen atoms in the chain respectively. The H(added) column lists the number of hydrogen atoms added and optimized by MolProbity. The Clashes column lists the number of clashes within the asymmetric unit, whereas Symm-Clashes lists symmetry related clashes.

Mol	Chain	Non-H	H(model)	H(added)	Clashes	Symm-Clashes
1	A	2576	0	2528	19	0
1	B	2605	0	2645	14	0
1	C	2616	0	2618	11	0
1	D	2540	0	2507	11	0
2	A	16	0	24	2	0
2	B	8	0	12	0	0

Continued on next page...

Continued from previous page...

Mol	Chain	Non-H	H(model)	H(added)	Clashes	Symm-Clashes
2	C	24	0	36	0	0
2	D	12	0	18	0	0
3	B	44	0	26	0	0
3	C	44	0	26	0	0
3	D	44	0	26	1	0
4	A	211	0	0	3	0
4	B	303	0	0	4	0
4	C	338	0	0	2	0
4	D	185	0	0	2	0
All	All	11566	0	10466	54	0

The all-atom clashscore is defined as the number of clashes found per 1000 atoms (including hydrogen atoms). The all-atom clashscore for this structure is 3.

All (54) close contacts within the same asymmetric unit are listed below, sorted by their clash magnitude.

Atom-1	Atom-2	Interatomic distance (Å)	Clash overlap (Å)
1:D:1:MET:HG2	1:D:27:GLU:HB2	1.67	0.74
1:D:77:GLU:HG2	1:D:82:LYS:HB2	1.76	0.68
1:C:26[B]:VAL:HG22	1:C:327:ILE:HD11	1.76	0.67
1:A:178:LEU:HD22	1:A:199:ALA:HB1	1.77	0.65
1:D:138:LYS:H	1:D:141:MET:CE	2.14	0.60
1:A:103:LEU:HD23	1:A:125:THR:HG22	1.83	0.59
1:B:127:THR:HG23	1:B:145:ASN:OD1	2.03	0.59
1:B:176:HIS:HB3	1:B:231:ARG:HD3	1.85	0.59
1:C:127:THR:HG23	1:C:145:ASN:OD1	2.04	0.58
1:A:140:GLU:OE1	4:A:501:HOH:O	2.18	0.57
1:D:138:LYS:H	1:D:141:MET:HE2	1.71	0.55
1:D:137:PHE:HA	1:D:141:MET:HE1	1.88	0.54
1:A:33:ASP:OD1	1:A:34:ILE:N	2.42	0.52
1:C:184[A]:THR:HG22	4:C:1946:HOH:O	2.08	0.52
1:A:85:TRP:HB3	1:A:90:VAL:HB	1.92	0.51
1:D:10:GLY:HA3	3:D:1502:NAD:O5B	2.10	0.51
1:B:190:LYS:HG3	4:B:575:HOH:O	2.09	0.51
1:B:139:PRO:HG3	1:B:332:VAL:HG12	1.92	0.51
1:C:81:GLU:HB3	1:C:108:LYS:HB3	1.93	0.51
1:C:178:LEU:HD22	1:C:199:ALA:HB1	1.93	0.50
1:A:176:HIS:HB3	1:A:231[B]:ARG:HD3	1.93	0.49
1:B:252:LYS:NZ	4:B:504:HOH:O	2.29	0.49
1:B:172[B]:MET:HG3	1:B:227:GLY:HA3	1.96	0.48
1:D:23:ARG:NH2	4:D:1603:HOH:O	2.34	0.48

Continued on next page...

Continued from previous page...

Atom-1	Atom-2	Interatomic distance (Å)	Clash overlap (Å)
1:C:274:TYR:CE2	1:C:276:GLU:HG3	2.48	0.47
1:D:139:PRO:HG3	1:D:332:VAL:HG12	1.97	0.47
1:D:46:LYS:NZ	4:D:1604:HOH:O	2.35	0.47
1:A:277:GLU:OE1	1:C:47:TYR:OH	2.32	0.46
1:A:83:LEU:O	1:A:112:GLY:HA3	2.16	0.46
1:A:274:TYR:CE1	1:A:276:GLU:HG3	2.51	0.45
1:D:176:HIS:HB3	1:D:231:ARG:HD3	1.99	0.45
1:C:252:LYS:NZ	1:C:256:ASP:OD1	2.48	0.45
1:C:83:LEU:HD13	1:C:85:TRP:CZ2	2.51	0.44
1:A:179:THR:OG1	1:A:231[B]:ARG:NH2	2.43	0.44
1:B:267:ARG:NH2	4:B:519:HOH:O	2.51	0.44
1:A:12:ILE:H	2:A:403:EDO:H12	1.83	0.43
1:B:178:LEU:HD22	1:B:199:ALA:HB1	2.00	0.43
1:C:23:ARG:HD2	4:C:1706:HOH:O	2.18	0.43
1:A:276:GLU:HG2	1:A:295[B]:SER:OG	2.19	0.43
1:A:12:ILE:HD13	1:A:12:ILE:HA	1.94	0.42
1:B:17:PHE:HZ	1:B:67:VAL:HG21	1.85	0.42
1:A:276:GLU:O	1:A:296:LYS:HE3	2.20	0.42
1:A:99:VAL:O	4:A:503:HOH:O	2.21	0.42
1:B:172[A]:MET:HE2	1:B:172[A]:MET:HB3	1.91	0.42
1:D:176:HIS:HA	1:D:238:SER:HB3	2.00	0.42
1:A:77:GLU:HB2	1:A:83:LEU:HD21	2.02	0.41
1:A:208:THR:HG23	4:A:660:HOH:O	2.19	0.41
1:B:238:SER:HB2	1:B:312:TYR:CZ	2.56	0.41
1:B:80:PRO:HA	1:B:83:LEU:HD12	2.02	0.41
1:A:11:ARG:HB3	2:A:403:EDO:H12	2.04	0.40
1:B:77:GLU:HB3	1:B:83:LEU:HD21	2.03	0.40
1:C:276:GLU:HG2	1:C:295[A]:SER:OG	2.21	0.40
1:A:182:GLN:HB3	1:A:199:ALA:HB2	2.02	0.40
1:B:127:THR:HG21	4:B:764:HOH:O	2.21	0.40

There are no symmetry-related clashes.

5.3 Torsion angles (i)

5.3.1 Protein backbone (i)

In the following table, the Percentiles column shows the percent Ramachandran outliers of the chain as a percentile score with respect to all X-ray entries followed by that with respect to entries of similar resolution.

The Analysed column shows the number of residues for which the backbone conformation was analysed, and the total number of residues.

Mol	Chain	Analysed	Favoured	Allowed	Outliers	Percentiles	
1	A	344/341 (101%)	332 (96%)	11 (3%)	1 (0%)	41	27
1	B	340/341 (100%)	328 (96%)	11 (3%)	1 (0%)	41	27
1	C	344/341 (101%)	330 (96%)	13 (4%)	1 (0%)	41	27
1	D	337/341 (99%)	324 (96%)	12 (4%)	1 (0%)	41	27
All	All	1365/1364 (100%)	1314 (96%)	47 (3%)	4 (0%)	41	27

All (4) Ramachandran outliers are listed below:

Mol	Chain	Res	Type
1	A	237	VAL
1	B	237	VAL
1	C	237	VAL
1	D	237	VAL

5.3.2 Protein sidechains (i)

In the following table, the Percentiles column shows the percent sidechain outliers of the chain as a percentile score with respect to all X-ray entries followed by that with respect to entries of similar resolution.

The Analysed column shows the number of residues for which the sidechain conformation was analysed, and the total number of residues.

Mol	Chain	Analysed	Rotameric	Outliers	Percentiles	
1	A	276/290 (95%)	275 (100%)	1 (0%)	91	89
1	B	291/290 (100%)	287 (99%)	4 (1%)	67	59
1	C	291/290 (100%)	289 (99%)	2 (1%)	84	81
1	D	274/290 (94%)	271 (99%)	3 (1%)	73	68
All	All	1132/1160 (98%)	1122 (99%)	10 (1%)	84	75

All (10) residues with a non-rotameric sidechain are listed below:

Mol	Chain	Res	Type
1	A	-2	HIS
1	B	1	MET
1	B	172[A]	MET
1	B	172[B]	MET

Continued on next page...

Continued from previous page...

Mol	Chain	Res	Type
1	B	312	TYR
1	C	104[A]	ASP
1	C	104[B]	ASP
1	D	1	MET
1	D	136	GLU
1	D	172	MET

Some sidechains can be flipped to improve hydrogen bonding and reduce clashes. There are no such sidechains identified.

5.3.3 RNA (i)

There are no RNA molecules in this entry.

5.4 Non-standard residues in protein, DNA, RNA chains (i)

There are no non-standard protein/DNA/RNA residues in this entry.

5.5 Carbohydrates (i)

There are no carbohydrates in this entry.

5.6 Ligand geometry (i)

18 ligands are modelled in this entry.

In the following table, the Counts columns list the number of bonds (or angles) for which Mogul statistics could be retrieved, the number of bonds (or angles) that are observed in the model and the number of bonds (or angles) that are defined in the Chemical Component Dictionary. The Link column lists molecule types, if any, to which the group is linked. The Z score for a bond length (or angle) is the number of standard deviations the observed value is removed from the expected value. A bond length (or angle) with $|Z| > 2$ is considered an outlier worth inspection. RMSZ is the root-mean-square of all Z scores of the bond lengths (or angles).

Mol	Type	Chain	Res	Link	Bond lengths			Bond angles		
					Counts	RMSZ	$\#\|Z\| > 2$	Counts	RMSZ	$\#\|Z\| > 2$
2	EDO	C	1604[B]	-	3,3,3	0.47	0	2,2,2	0.27	0
2	EDO	C	1603	-	3,3,3	0.52	0	2,2,2	0.21	0
2	EDO	A	401	-	3,3,3	0.47	0	2,2,2	0.26	0
3	NAD	C	1602	-	42,48,48	0.94	3 (7%)	50,73,73	1.11	3 (6%)

Mol	Type	Chain	Res	Link	Bond lengths			Bond angles		
					Counts	RMSZ	#|Z| > 2	Counts	RMSZ	#|Z| > 2
2	EDO	C	1601	-	3,3,3	0.42	0	2,2,2	0.42	0
2	EDO	B	403	-	3,3,3	0.49	0	2,2,2	0.18	0
3	NAD	B	401	-	42,48,48	1.01	3 (7%)	50,73,73	0.96	3 (6%)
2	EDO	A	402[B]	-	3,3,3	0.44	0	2,2,2	0.36	0
2	EDO	B	402	-	3,3,3	0.45	0	2,2,2	0.33	0
2	EDO	D	1501	-	3,3,3	0.46	0	2,2,2	0.23	0
2	EDO	D	1503	-	3,3,3	0.43	0	2,2,2	0.43	0
2	EDO	A	402[A]	-	3,3,3	0.49	0	2,2,2	0.35	0
2	EDO	D	1504	-	3,3,3	0.46	0	2,2,2	0.39	0
2	EDO	C	1606	-	3,3,3	0.43	0	2,2,2	0.28	0
2	EDO	C	1605	-	3,3,3	0.46	0	2,2,2	0.39	0
2	EDO	C	1604[A]	-	3,3,3	0.50	0	2,2,2	0.19	0
2	EDO	A	403	-	3,3,3	0.56	0	2,2,2	0.11	0
3	NAD	D	1502	-	42,48,48	1.00	3 (7%)	50,73,73	0.97	0

In the following table, the Chirals column lists the number of chiral outliers, the number of chiral centers analysed, the number of these observed in the model and the number defined in the Chemical Component Dictionary. Similar counts are reported in the Torsion and Rings columns. '-' means no outliers of that kind were identified.

Mol	Type	Chain	Res	Link	Chirals	Torsions	Rings
2	EDO	C	1604[B]	-	-	0/1/1/1	-
2	EDO	C	1603	-	-	0/1/1/1	-
2	EDO	A	401	-	-	0/1/1/1	-
3	NAD	C	1602	-	-	6/26/62/62	0/5/5/5
2	EDO	C	1601	-	-	1/1/1/1	-
2	EDO	B	403	-	-	0/1/1/1	-
3	NAD	B	401	-	-	5/26/62/62	0/5/5/5
2	EDO	A	402[B]	-	-	0/1/1/1	-
2	EDO	B	402	-	-	0/1/1/1	-
2	EDO	D	1501	-	-	0/1/1/1	-
2	EDO	D	1503	-	-	1/1/1/1	-
2	EDO	A	402[A]	-	-	0/1/1/1	-
2	EDO	D	1504	-	-	1/1/1/1	-
2	EDO	C	1606	-	-	0/1/1/1	-
2	EDO	C	1605	-	-	0/1/1/1	-
2	EDO	C	1604[A]	-	-	0/1/1/1	-
2	EDO	A	403	-	-	1/1/1/1	-
3	NAD	D	1502	-	-	5/26/62/62	0/5/5/5

All (9) bond length outliers are listed below:

Mol	Chain	Res	Type	Atoms	Z	Observed(Å)	Ideal(Å)
3	D	1502	NAD	PN-O5D	2.58	1.69	1.59
3	B	401	NAD	PN-O5D	2.36	1.68	1.59
3	C	1602	NAD	PN-O5D	2.27	1.68	1.59
3	B	401	NAD	C2N-N1N	2.13	1.37	1.35
3	D	1502	NAD	C8A-N7A	-2.13	1.30	1.34
3	B	401	NAD	C8A-N7A	-2.06	1.31	1.34
3	D	1502	NAD	O4D-C4D	2.05	1.49	1.45
3	C	1602	NAD	C8A-N7A	-2.03	1.31	1.34
3	C	1602	NAD	O4D-C4D	2.01	1.49	1.45

All (6) bond angle outliers are listed below:

Mol	Chain	Res	Type	Atoms	Z	Observed(^{o})	Ideal(^{o})
3	B	401	NAD	O4B-C1B-C2B	-2.51	103.25	106.93
3	C	1602	NAD	O2N-PN-O1N	2.24	123.34	112.24
3	C	1602	NAD	O3B-C3B-C4B	-2.19	104.71	111.05
3	C	1602	NAD	O4B-C1B-C2B	-2.12	103.83	106.93
3	B	401	NAD	C5A-C6A-N6A	2.09	123.53	120.35
3	B	401	NAD	O2N-PN-O1N	2.06	122.41	112.24

There are no chirality outliers.

All (20) torsion outliers are listed below:

Mol	Chain	Res	Type	Atoms
3	D	1502	NAD	O4D-C1D-N1N-C2N
3	D	1502	NAD	O4D-C1D-N1N-C6N
3	D	1502	NAD	C2D-C1D-N1N-C2N
3	D	1502	NAD	C2D-C1D-N1N-C6N
3	C	1602	NAD	O4D-C1D-N1N-C2N
3	C	1602	NAD	O4D-C1D-N1N-C6N
3	C	1602	NAD	C2D-C1D-N1N-C2N
3	C	1602	NAD	C2D-C1D-N1N-C6N
3	B	401	NAD	O4D-C1D-N1N-C2N
3	B	401	NAD	O4D-C1D-N1N-C6N
3	B	401	NAD	C2D-C1D-N1N-C2N
3	B	401	NAD	C2D-C1D-N1N-C6N
2	A	403	EDO	O1-C1-C2-O2
2	D	1503	EDO	O1-C1-C2-O2
3	D	1502	NAD	O4B-C4B-C5B-O5B
3	C	1602	NAD	O4B-C4B-C5B-O5B
2	C	1601	EDO	O1-C1-C2-O2
2	D	1504	EDO	O1-C1-C2-O2

Continued on next page...

Continued from previous page...

Mol	Chain	Res	Type	Atoms
3	B	401	NAD	O4B-C4B-C5B-O5B
3	C	1602	NAD	C3B-C4B-C5B-O5B

There are no ring outliers.

2 monomers are involved in 3 short contacts:

Mol	Chain	Res	Type	Clashes	Symm-Clashes
2	A	403	EDO	2	0
3	D	1502	NAD	1	0

The following is a two-dimensional graphical depiction of Mogul quality analysis of bond lengths, bond angles, torsion angles, and ring geometry for all instances of the Ligand of Interest. In addition, ligands with molecular weight > 250 and outliers as shown on the validation Tables will also be included. For torsion angles, if less then 5% of the Mogul distribution of torsion angles is within 10 degrees of the torsion angle in question, then that torsion angle is considered an outlier. Any bond that is central to one or more torsion angles identified as an outlier by Mogul will be highlighted in the graph. For rings, the root-mean-square deviation (RMSD) between the ring in question and similar rings identified by Mogul is calculated over all ring torsion angles. If the average RMSD is greater than 60 degrees and the minimal RMSD between the ring in question and any Mogul-identified rings is also greater than 60 degrees, then that ring is considered an outlier. The outliers are highlighted in purple. The color gray indicates Mogul did not find sufficient equivalents in the CSD to analyse the geometry.

Ligand NAD C 1602

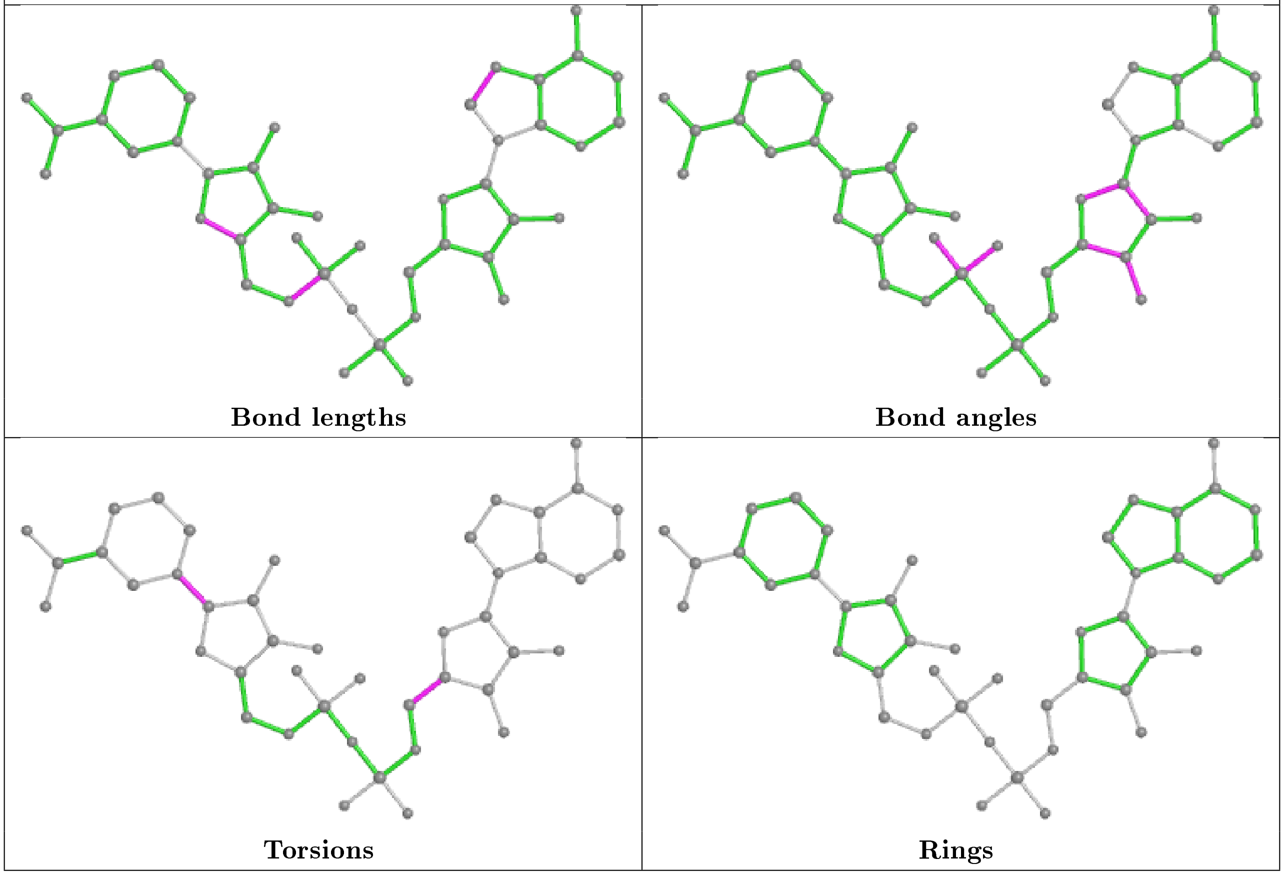

Ligand NAD B 401

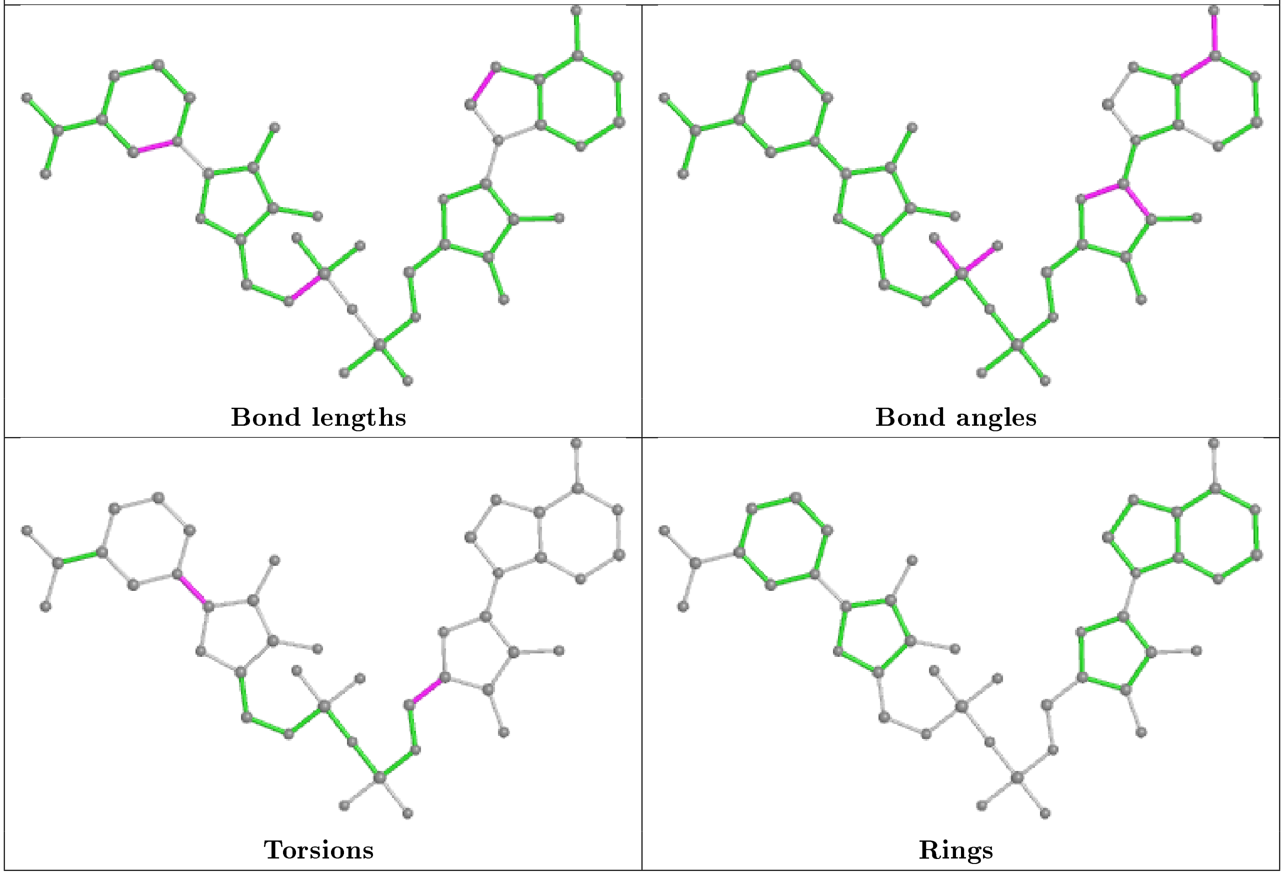

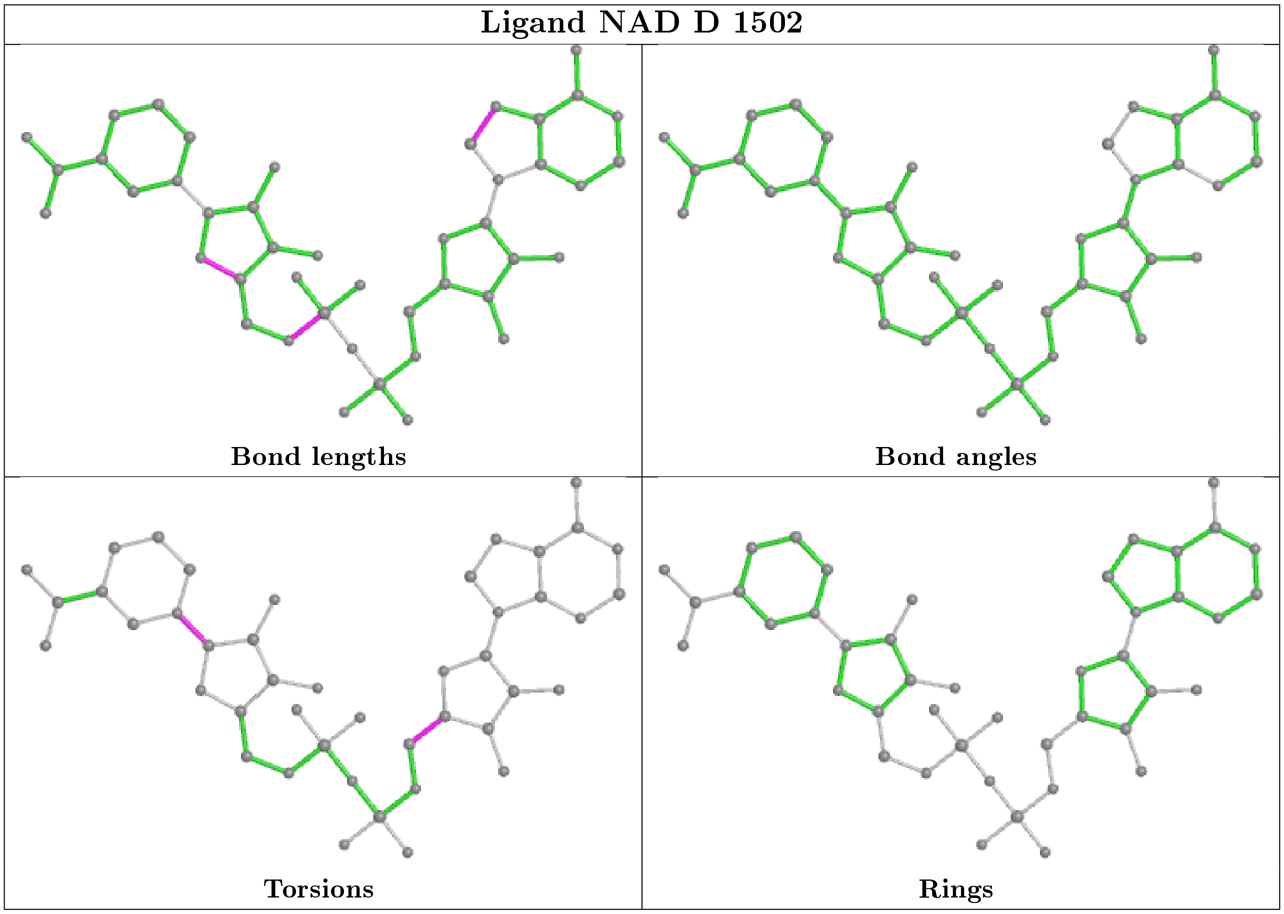

5.7 Other polymers (i)

There are no such residues in this entry.

5.8 Polymer linkage issues (i)

There are no chain breaks in this entry.

6 Fit of model and data (i)

6.1 Protein, DNA and RNA chains (i)

In the following table, the column labelled '#RSRZ> 2' contains the number (and percentage) of RSRZ outliers, followed by percent RSRZ outliers for the chain as percentile scores relative to all X-ray entries and entries of similar resolution. The OWAB column contains the minimum, median, 95th percentile and maximum values of the occupancy-weighted average B-factor per residue. The column labelled 'Q< 0.9' lists the number of (and percentage) of residues with an average occupancy less than 0.9.

Mol	Chain	Analysed	<RSRZ>	#RSRZ>2			OWAB(Å^2)	Q<0.9
1	A	337/341 (98%)	0.43	29 (8%)	10	8	12, 28, 66, 83	0
1	B	333/341 (97%)	-0.03	1 (0%)	94	92	12, 23, 40, 54	0
1	C	335/341 (98%)	0.03	1 (0%)	94	92	11, 19, 35, 76	0
1	D	332/341 (97%)	0.62	31 (9%)	8	6	13, 34, 64, 88	0
All	All	1337/1364 (98%)	0.26	62 (4%)	32	26	11, 25, 58, 88	0

All (62) RSRZ outliers are listed below:

Mol	Chain	Res	Type	RSRZ
1	A	76	CYS	7.5
1	A	103	LEU	7.2
1	A	34	ILE	5.8
1	A	99	VAL	5.2
1	A	35	MET	5.1
1	A	101	THR	4.6
1	D	26	VAL	4.5
1	A	100	PHE	4.4
1	D	137	PHE	4.2
1	A	75	PHE	4.2
1	A	110	LEU	4.2
1	A	104	ASP	4.1
1	D	80	PRO	4.1
1	D	106	ALA	3.9
1	A	87	GLN	3.8
1	A	81	GLU	3.8
1	D	101	THR	3.7
1	D	110	LEU	3.7
1	D	24	THR	3.4
1	D	1	MET	3.3
1	D	104	ASP	3.3

Continued on next page...

Continued from previous page...

Mol	Chain	Res	Type	RSRZ
1	A	112	GLY	3.3
1	D	103	LEU	3.3
1	D	141	MET	3.2
1	D	138	LYS	3.2
1	A	86	GLY	3.1
1	D	81	GLU	3.0
1	D	85	TRP	2.9
1	D	135	HIS	2.9
1	A	82	LYS	2.8
1	D	143	VAL	2.8
1	D	100	PHE	2.8
1	D	109	HIS	2.7
1	A	114	ALA	2.7
1	A	88	TYR	2.7
1	A	-2	HIS	2.6
1	A	80	PRO	2.6
1	A	107	SER	2.6
1	C	-1	HIS	2.5
1	D	69	GLY	2.5
1	D	78	ARG	2.5
1	D	327	ILE	2.5
1	A	105	THR	2.5
1	D	83	LEU	2.4
1	D	98	GLY	2.4
1	A	32	ASN	2.4
1	D	139	PRO	2.4
1	A	33	ASP	2.3
1	A	77	GLU	2.3
1	A	-3	HIS	2.3
1	D	140	GLU	2.2
1	B	104	ASP	2.2
1	D	233	PRO	2.2
1	A	85	TRP	2.2
1	D	23	ARG	2.2
1	A	79	GLU	2.2
1	D	107	SER	2.1
1	D	64	SER	2.1
1	D	105	THR	2.1
1	D	99	VAL	2.1
1	A	113	GLY	2.1
1	A	78	ARG	2.0

6.2 Non-standard residues in protein, DNA, RNA chains (i)

There are no non-standard protein/DNA/RNA residues in this entry.

6.3 Carbohydrates (i)

There are no carbohydrates in this entry.

6.4 Ligands (i)

In the following table, the Atoms column lists the number of modelled atoms in the group and the number defined in the chemical component dictionary. The B-factors column lists the minimum, median, 95th percentile and maximum values of B factors of atoms in the group. The column labelled 'Q< 0.9' lists the number of atoms with occupancy less than 0.9.

Mol	Type	Chain	Res	Atoms	RSCC	RSR	B-factors($Å^2$)	Q<0.9
2	EDO	A	402[B]	4/4	0.73	0.41	27,28,30,30	4
2	EDO	A	402[A]	4/4	0.73	0.41	27,29,30,34	4
2	EDO	C	1605	4/4	0.74	0.17	51,51,52,56	0
2	EDO	A	401	4/4	0.80	0.27	44,45,50,55	0
2	EDO	D	1504	4/4	0.80	0.31	46,50,51,57	0
2	EDO	D	1503	4/4	0.80	0.13	43,44,46,52	0
2	EDO	A	403	4/4	0.80	0.17	29,31,34,37	0
3	NAD	D	1502	44/44	0.86	0.19	25,46,54,57	0
2	EDO	B	403	4/4	0.86	0.18	37,38,38,44	0
2	EDO	C	1604[A]	4/4	0.89	0.33	39,39,40,40	4
2	EDO	C	1604[B]	4/4	0.89	0.33	36,39,40,41	4
2	EDO	B	402	4/4	0.93	0.10	37,37,39,41	0
2	EDO	D	1501	4/4	0.93	0.12	55,56,57,60	0
2	EDO	C	1603	4/4	0.94	0.15	28,31,31,37	0
2	EDO	C	1601	4/4	0.95	0.11	37,44,48,53	0
3	NAD	C	1602	44/44	0.96	0.10	8,15,19,24	0
2	EDO	C	1606	4/4	0.96	0.13	28,32,42,48	0
3	NAD	B	401	44/44	0.96	0.09	11,17,21,23	0

The following is a graphical depiction of the model fit to experimental electron density of all instances of the Ligand of Interest. In addition, ligands with molecular weight > 250 and outliers as shown on the geometry validation Tables will also be included. Each fit is shown from different orientation to approximate a three-dimensional view.

Electron density around NAD D 1502:

2mF$_o$-DF$_c$ (at 0.7 rmsd) in gray
mF$_o$-DF$_c$ (at 3 rmsd) in purple (negative)
and green (positive)

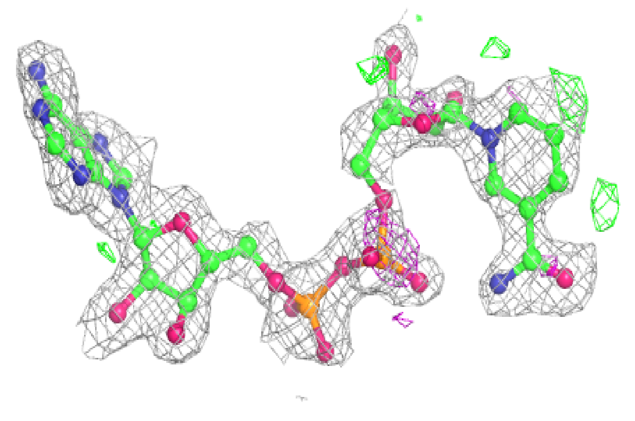

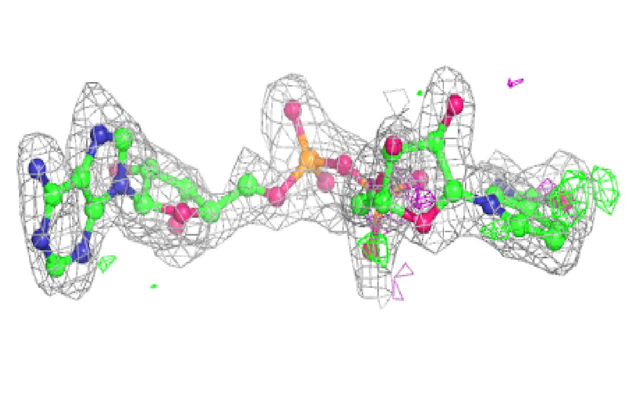

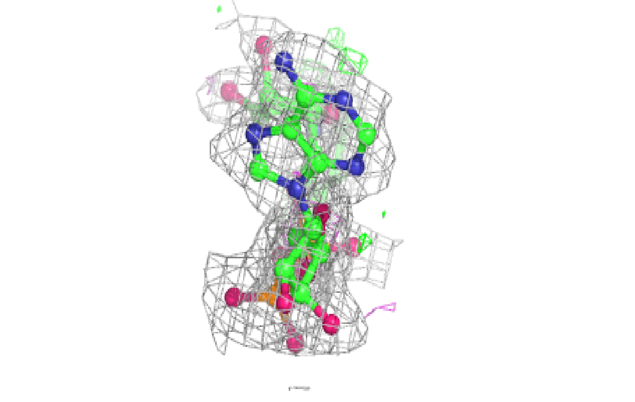

Electron density around NAD C 1602:

2mF$_o$-DF$_c$ (at 0.7 rmsd) in gray
mF$_o$-DF$_c$ (at 3 rmsd) in purple (negative)
and green (positive)

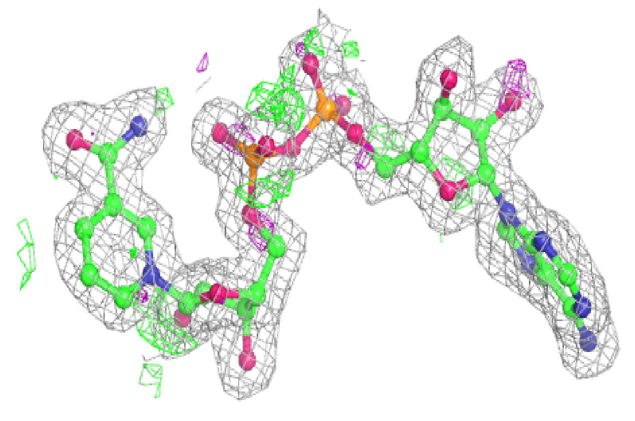

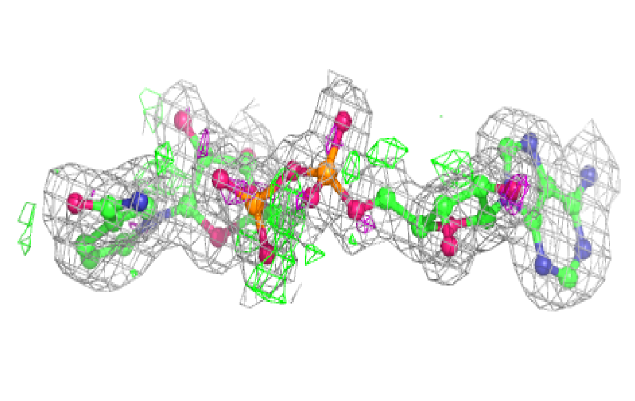

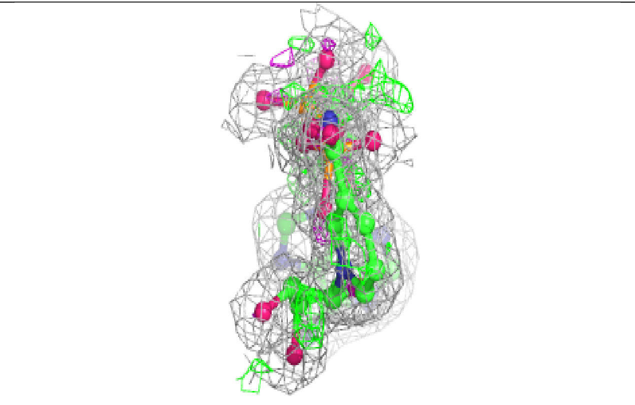

Electron density around NAD B 401:

$2mF_o$-DF_c (at 0.7 rmsd) in gray
mF_o-DF_c (at 3 rmsd) in purple (negative)
and green (positive)

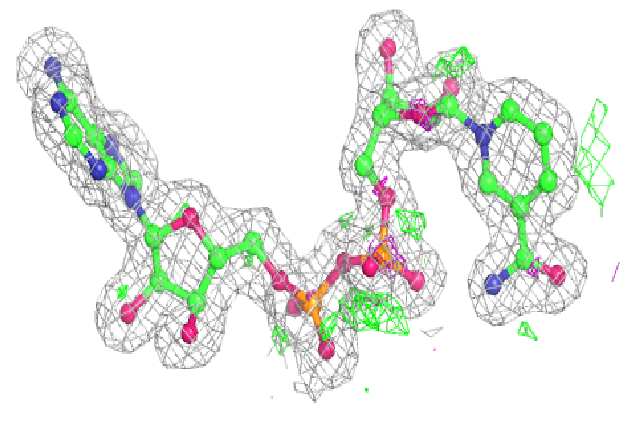

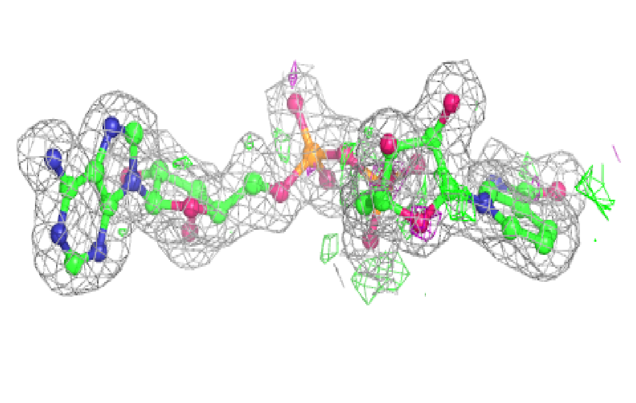

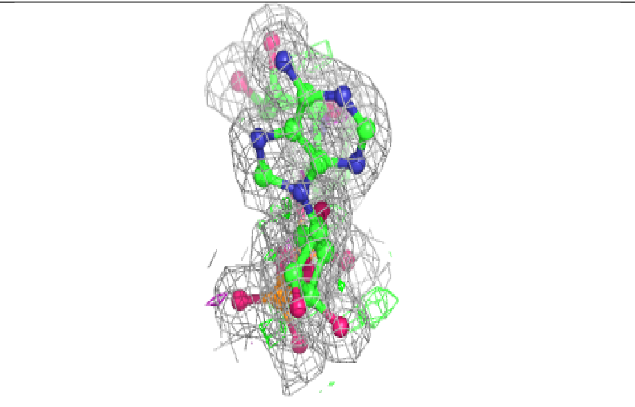

6.5 Other polymers ⓘ

There are no such residues in this entry.
