# Supplementary material for: Naegleria fowleri: Protein structures to facilitate drug discovery for the deadly, pathogenic free-living amoeba
Source: PLoS One. 2021 Mar 24;16(3):e0241738. doi: 10.1371/journal.pone.0241738 (PMC7990177; doi:10.1371/journal.pone.0241738)
Supplement: S1 File — (ZIP) [file pone.0241738.s002.zip › 6um4_full_validation.pdf]

Full wwPDB X-ray Structure Validation Report (i)

May 17, 2020 – 10:15 am BST

PDB ID	:	6UM4
Title	:	Crystal structure of malate dehydrogenase from Naegleria fowleri ATCC 30863
Authors	:	Seattle Structural Genomics Center for Infectious Disease (SSGCID)
Deposited on	:	2019-10-09
Resolution	:	2.05 Å(reported)

This is a Full wwPDB X-ray Structure Validation Report for a publicly released PDB entry.

We welcome your comments at *validation@mail.wwpdb.org*
A user guide is available at
https://www.wwpdb.org/validation/2017/XrayValidationReportHelp
with specific help available everywhere you see the (i) symbol.

The following versions of software and data (see references (i)) were used in the production of this report:

MolProbity	:	4.02b-467
Mogul	:	1.8.5 (274361), CSD as541be (2020)
Xtriage (Phenix)	:	1.13
EDS	:	2.11
Percentile statistics	:	20191225.v01 (using entries in the PDB archive December 25th 2019)
Refmac	:	5.8.0158
CCP4	:	7.0.044 (Gargrove)
Ideal geometry (proteins)	:	Engh & Huber (2001)
Ideal geometry (DNA, RNA)	:	Parkinson et al. (1996)
Validation Pipeline (wwPDB-VP)	:	2.11

1 Overall quality at a glance (i)

The following experimental techniques were used to determine the structure:
X-RAY DIFFRACTION

The reported resolution of this entry is 2.05 Å.

Percentile scores (ranging between 0-100) for global validation metrics of the entry are shown in the following graphic. The table shows the number of entries on which the scores are based.

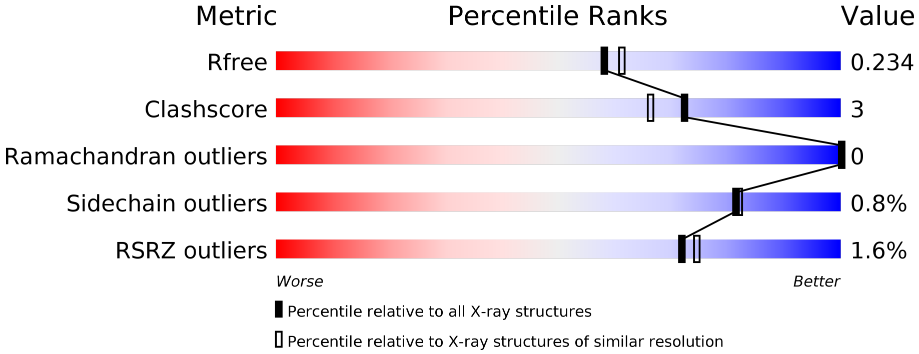

Metric	Whole archive (#Entries)	Similar resolution (#Entries, resolution range(Å))
R_{free}	130704	1692 (2.04-2.04)
Clashscore	141614	1773 (2.04-2.04)
Ramachandran outliers	138981	1752 (2.04-2.04)
Sidechain outliers	138945	1752 (2.04-2.04)
RSRZ outliers	127900	1672 (2.04-2.04)

The table below summarises the geometric issues observed across the polymeric chains and their fit to the electron density. The red, orange, yellow and green segments on the lower bar indicate the fraction of residues that contain outliers for >=3, 2, 1 and 0 types of geometric quality criteria respectively. A grey segment represents the fraction of residues that are not modelled. The numeric value for each fraction is indicated below the corresponding segment, with a dot representing fractions <=5% The upper red bar (where present) indicates the fraction of residues that have poor fit to the electron density. The numeric value is given above the bar.

Mol	Chain	Length	Quality of chain
1	A	444	% / 68% 5% 27%
1	B	444	% / 66% 7% 27%
1	C	444	2% / 66% 7% 27%
1	D	444	2% / 67% 6% 27%
1	E	444	% / 68% 5% 27%
1	F	444	% / 67% 7% 27%

Continued on next page...

Continued from previous page...

Mol	Chain	Length	Quality of chain
1	G	444	% 66% 7% 27%
1	H	444	% 68% 5% 27%
1	I	444	2% 66% 7% 27%
1	J	444	% 66% 7% 27%
1	K	444	2% 67% 7% 27%
1	L	444	69% 5% 27%

2 Entry composition (i)

There are 3 unique types of molecules in this entry. The entry contains 31359 atoms, of which 0 are hydrogens and 0 are deuteriums.

In the tables below, the ZeroOcc column contains the number of atoms modelled with zero occupancy, the AltConf column contains the number of residues with at least one atom in alternate conformation and the Trace column contains the number of residues modelled with at most 2 atoms.

- Molecule 1 is a protein called Malate dehydrogenase.

Mol	Chain	Residues	Atoms					ZeroOcc	AltConf	Trace
			Total	C	N	O	S			
1	A	326	2442	1540	423	464	15	0	3	0
1	B	326	2419	1523	418	464	14	0	2	0
1	C	326	2431	1526	427	464	14	0	2	0
1	D	325	2420	1523	420	463	14	0	2	0
1	E	326	2428	1528	421	464	15	0	1	0
1	F	326	2436	1531	424	466	15	0	3	0
1	G	326	2422	1523	419	466	14	0	2	0
1	H	326	2419	1523	416	466	14	0	2	0
1	I	326	2423	1525	420	464	14	0	2	0
1	J	323	2410	1514	420	463	13	0	1	0
1	K	326	2398	1507	416	462	13	0	0	0
1	L	326	2443	1537	426	465	15	0	2	0

- Molecule 2 is 1,2-ETHANEDIOL (three-letter code: EDO) (formula: $C_2H_6O_2$).

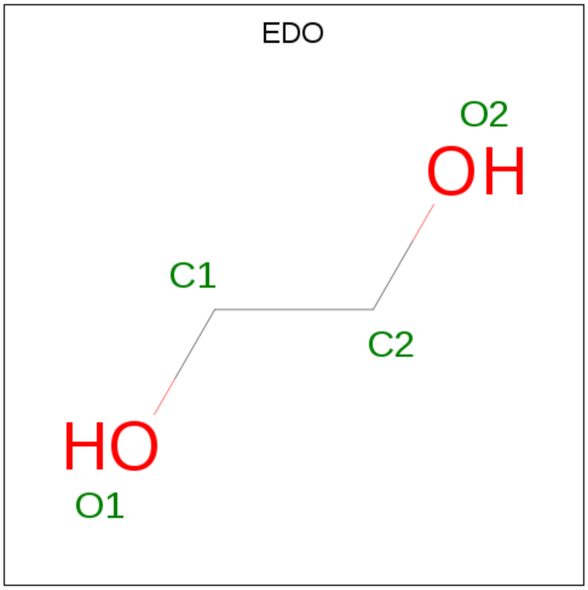

Mol	Chain	Residues	Atoms	ZeroOcc	AltConf
2	D	1	Total C O<br>4 2 2	0	0
2	J	1	Total C O<br>4 2 2	0	0
2	J	1	Total C O<br>4 2 2	0	0
2	L	1	Total C O<br>4 2 2	0	0

- Molecule 3 is water.

Mol	Chain	Residues	Atoms	ZeroOcc	AltConf
3	A	223	Total O<br>225 225	0	2
3	B	177	Total O<br>178 178	0	1
3	C	163	Total O<br>165 165	0	2
3	D	169	Total O<br>171 171	0	2
3	E	199	Total O<br>201 201	0	2
3	F	179	Total O<br>180 180	0	1
3	G	183	Total O<br>184 184	0	1
3	H	186	Total O<br>188 188	0	2

Continued on next page...

Continued from previous page...

Mol	Chain	Residues	Atoms		ZeroOcc	AltConf
3	I	164	Total 164	O 164	0	0
3	J	220	Total 221	O 221	0	1
3	K	175	Total 176	O 176	0	1
3	L	199	Total 199	O 199	0	0

3 Residue-property plots ⓘ

These plots are drawn for all protein, RNA and DNA chains in the entry. The first graphic for a chain summarises the proportions of the various outlier classes displayed in the second graphic. The second graphic shows the sequence view annotated by issues in geometry and electron density. Residues are color-coded according to the number of geometric quality criteria for which they contain at least one outlier: green = 0, yellow = 1, orange = 2 and red = 3 or more. A red dot above a residue indicates a poor fit to the electron density (RSRZ > 2). Stretches of 2 or more consecutive residues without any outlier are shown as a green connector. Residues present in the sample, but not in the model, are shown in grey.

- Molecule 1: Malate dehydrogenase

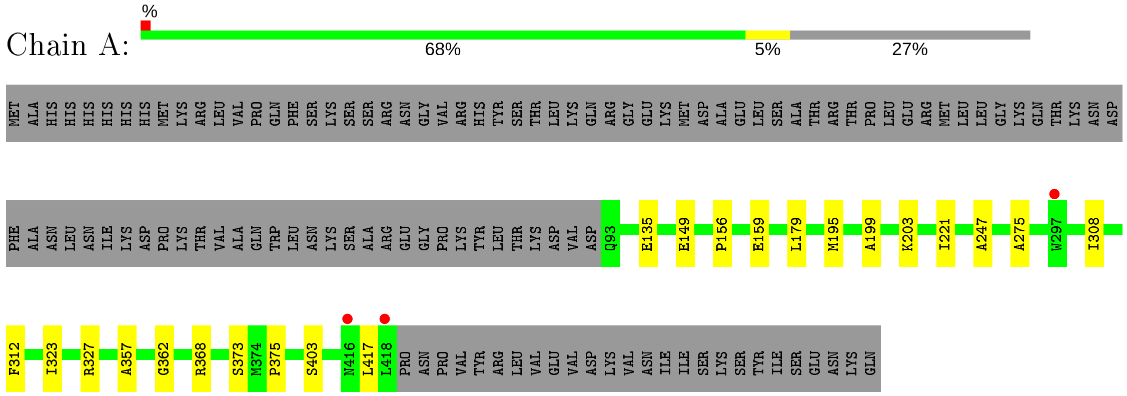

- Molecule 1: Malate dehydrogenase

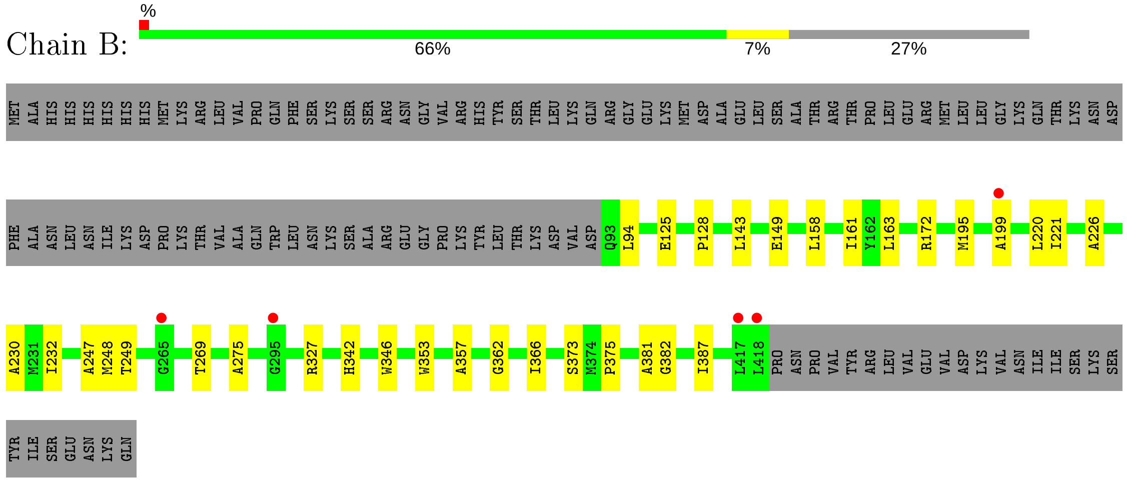

- Molecule 1: Malate dehydrogenase

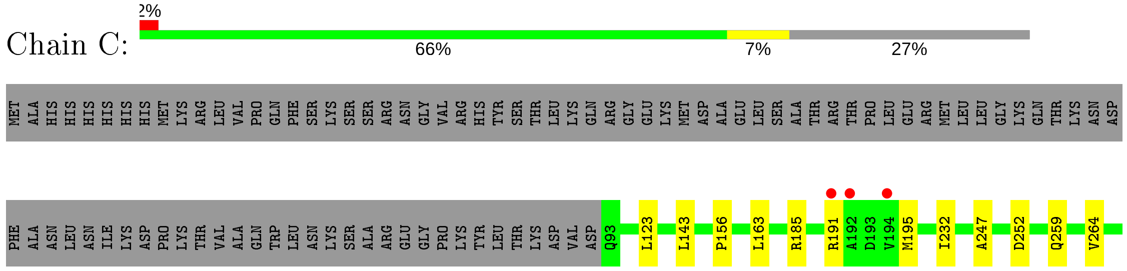

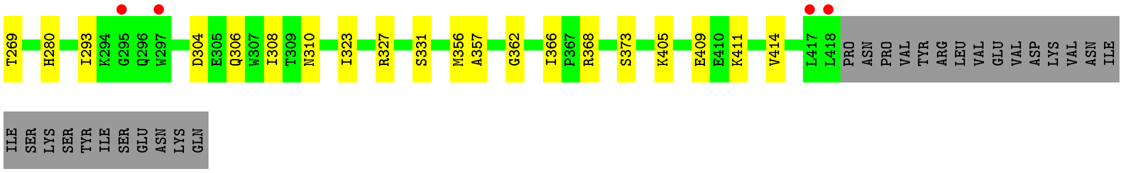

• Molecule 1: Malate dehydrogenase

Chain D:

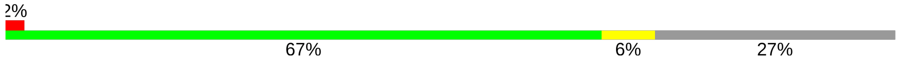

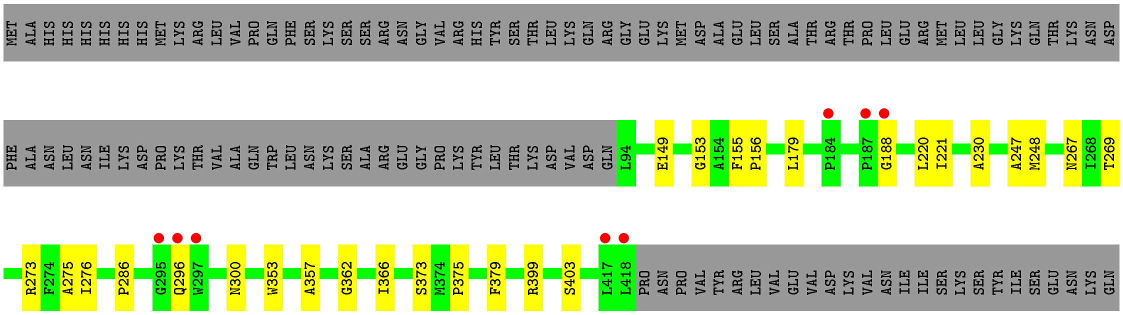

• Molecule 1: Malate dehydrogenase

Chain E:

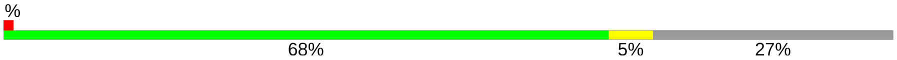

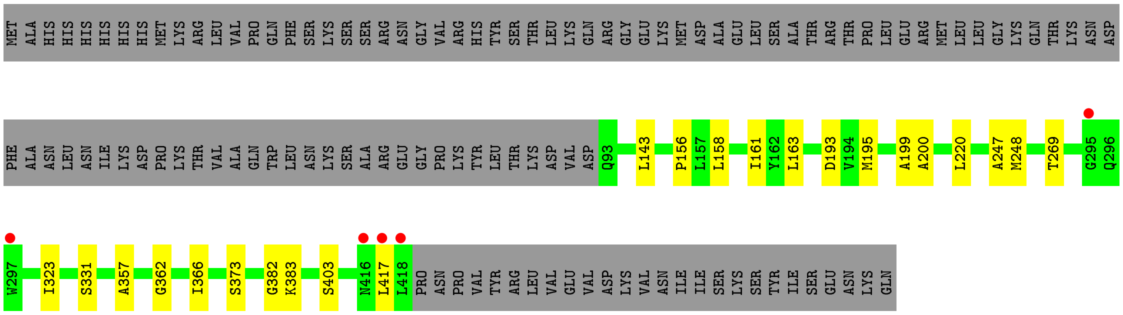

• Molecule 1: Malate dehydrogenase

Chain F:

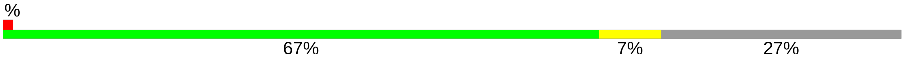

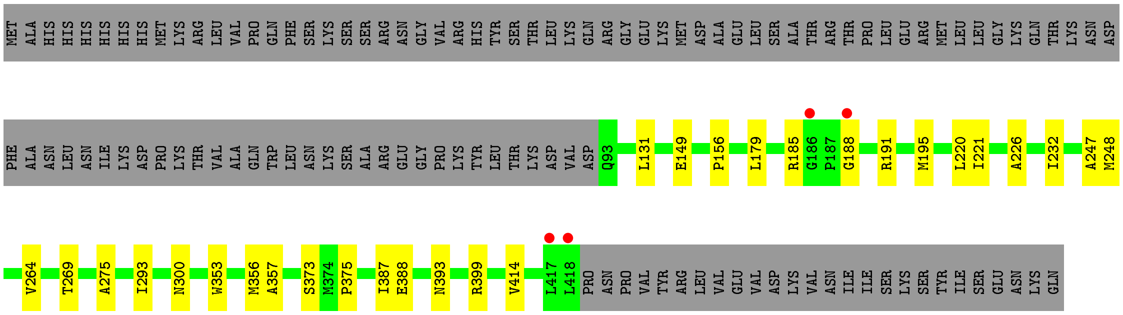

• Molecule 1: Malate dehydrogenase

Chain G:

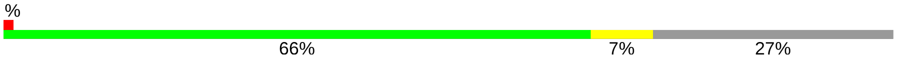

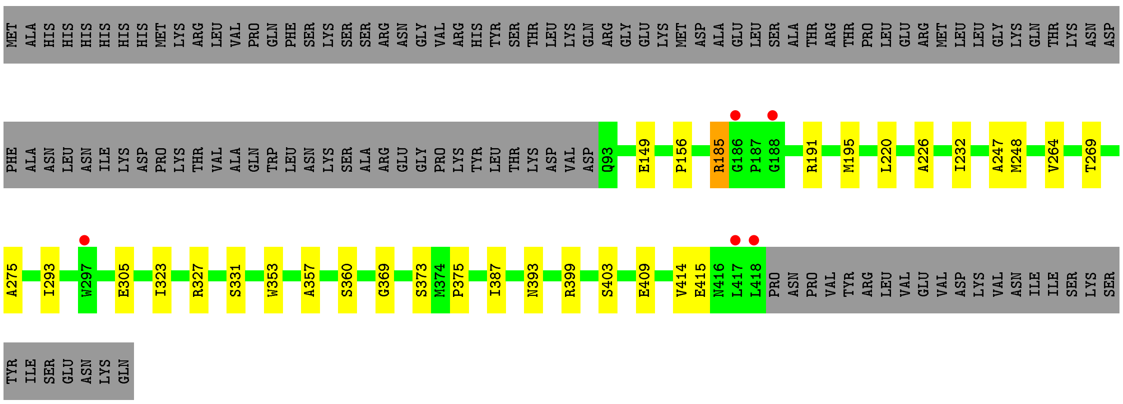

• Molecule 1: Malate dehydrogenase

Chain H:

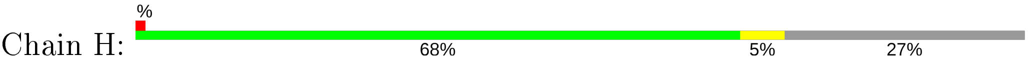

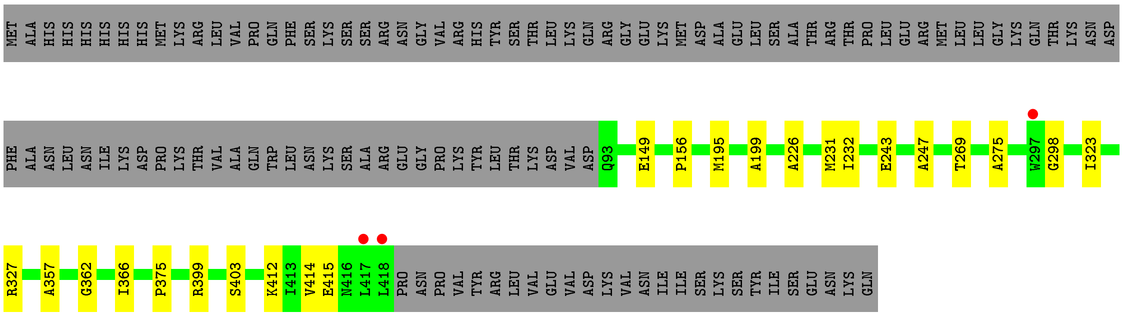

• Molecule 1: Malate dehydrogenase

Chain I:

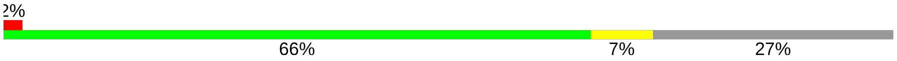

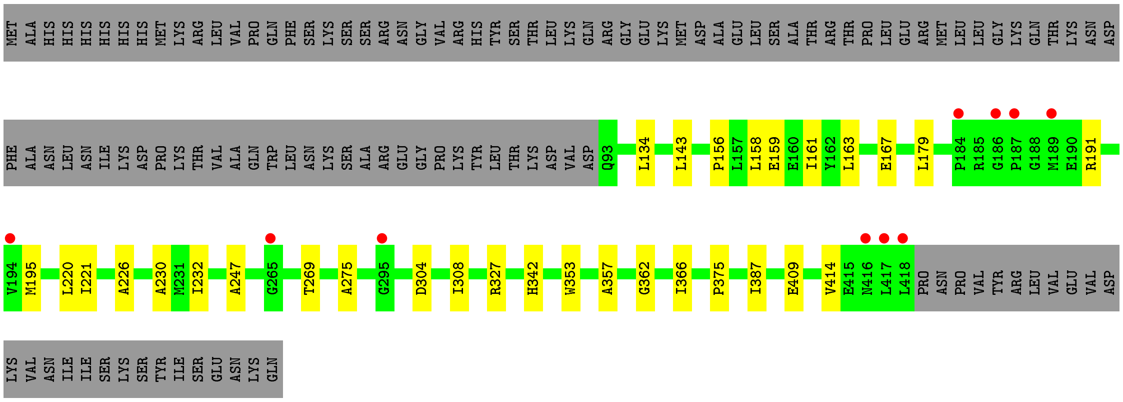

• Molecule 1: Malate dehydrogenase

Chain J:

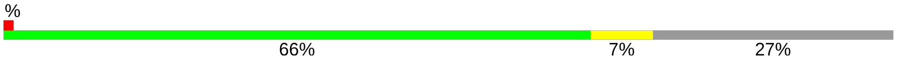

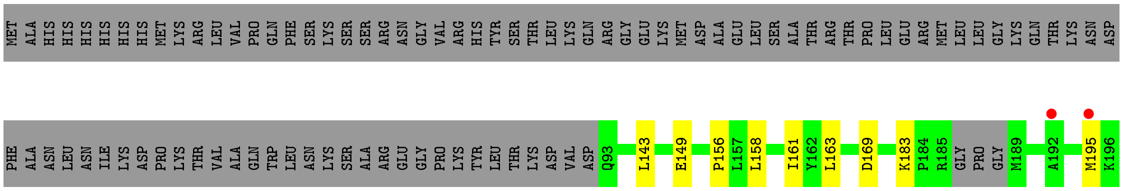

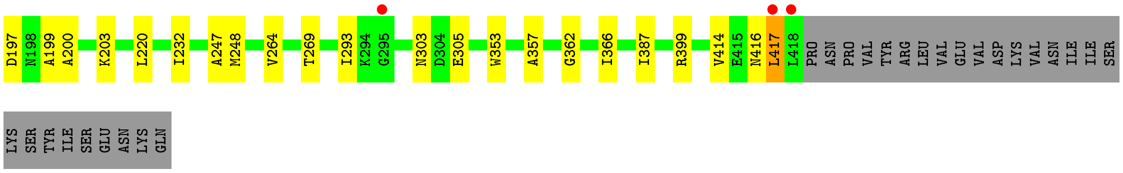

• Molecule 1: Malate dehydrogenase

Chain K:

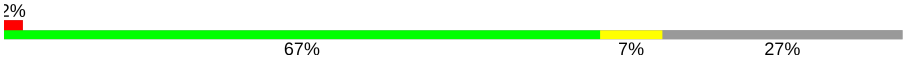

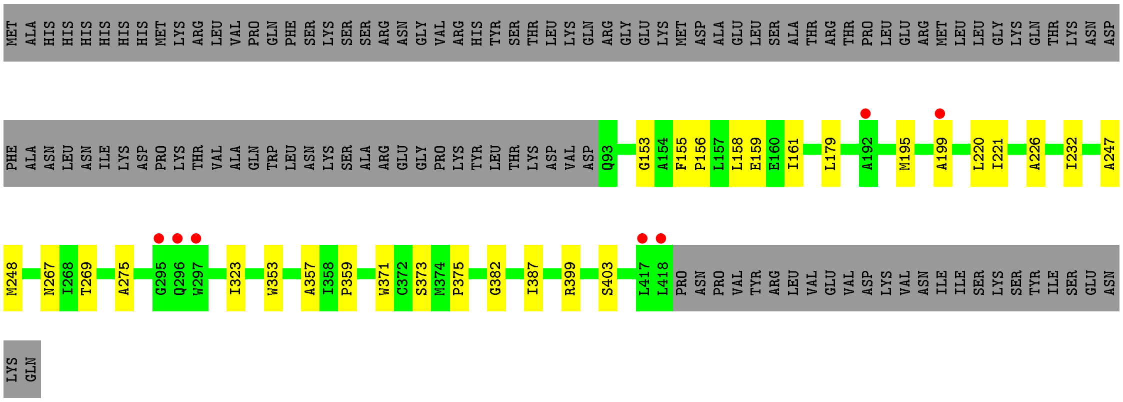

• Molecule 1: Malate dehydrogenase

Chain L:

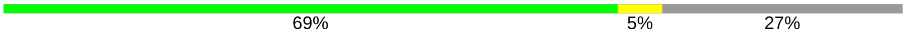

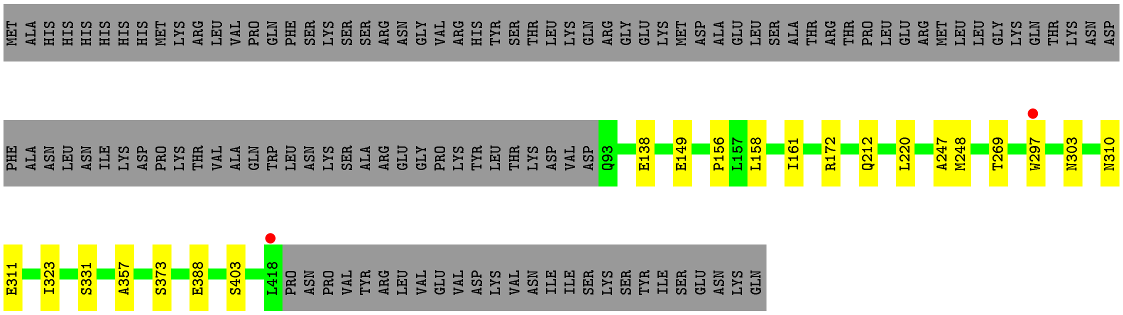

4 Data and refinement statistics (i)

Property	Value	Source
Space group	P 1	Depositor
Cell constants<br>a, b, c, α, β, γ	60.33Å 137.48Å 139.28Å<br>91.09° 90.00° 91.48°	Depositor
Resolution (Å)	44.23 – 2.05<br>48.44 – 2.05	Depositor<br>EDS
% Data completeness<br>(in resolution range)	96.5 (44.23-2.05)<br>96.7 (48.44-2.05)	Depositor<br>EDS
R_{merge}	0.09	Depositor
R_{sym}	(Not available)	Depositor
$< I/\sigma(I) >$ [1]	2.18 (at 2.05Å)	Xtriage
Refinement program	PHENIX 1.17_3644	Depositor
R, R_{free}	0.211 , 0.232<br>0.210 , 0.234	Depositor<br>DCC
R_{free} test set	1860 reflections (0.69%)	wwPDB-VP
Wilson B-factor (Å^2)	22.9	Xtriage
Anisotropy	1.133	Xtriage
Bulk solvent k_{sol}(e/Å^3), B_{sol}(Å^2)	0.35 , 57.1	EDS
L-test for twinning[2]	$< \|L\| >$ = 0.51, $< L^2 >$ = 0.34	Xtriage
Estimated twinning fraction	0.000 for h,l,-k<br>0.000 for h,-l,k<br>0.114 for h,-k,-l<br>0.000 for -h,k,-l<br>0.000 for -h,-k,l<br>0.000 for -h,l,k<br>0.000 for -h,-l,-k	Xtriage
F_o,F_c correlation	0.95	EDS
Total number of atoms	31359	wwPDB-VP
Average B, all atoms (Å^2)	33.0	wwPDB-VP

Xtriage's analysis on translational NCS is as follows: *The analyses of the Patterson function reveals a significant off-origin peak that is 48.05 % of the origin peak, indicating pseudo-translational symmetry. The chance of finding a peak of this or larger height randomly in a structure without pseudo-translational symmetry is equal to 9.0936e-05. The detected translational NCS is most likely also responsible for the elevated intensity ratio.*

[1] Intensities estimated from amplitudes.

[2] Theoretical values of $< |L| >$, $< L^2 >$ for acentric reflections are 0.5, 0.333 respectively for untwinned datasets, and 0.375, 0.2 for perfectly twinned datasets.

5 Model quality (i)

5.1 Standard geometry (i)

Bond lengths and bond angles in the following residue types are not validated in this section: EDO

The Z score for a bond length (or angle) is the number of standard deviations the observed value is removed from the expected value. A bond length (or angle) with $|Z| > 5$ is considered an outlier worth inspection. RMSZ is the root-mean-square of all Z scores of the bond lengths (or angles).

Mol	Chain	Bond lengths		Bond angles	
		RMSZ	#|Z| >5	RMSZ	#|Z| >5
1	A	0.38	0/2497	0.54	0/3396
1	B	0.37	0/2471	0.53	0/3365
1	C	0.38	0/2481	0.54	0/3373
1	D	0.36	0/2472	0.53	0/3365
1	E	0.38	0/2477	0.54	0/3371
1	F	0.36	0/2491	0.53	0/3389
1	G	0.39	0/2474	0.53	0/3370
1	H	0.38	0/2471	0.56	0/3367
1	I	0.37	0/2475	0.54	0/3371
1	J	0.40	0/2455	0.54	0/3338
1	K	0.37	0/2444	0.52	0/3332
1	L	0.38	0/2495	0.56	0/3393
All	All	0.38	0/29703	0.54	0/40430

There are no bond length outliers.

There are no bond angle outliers.

There are no chirality outliers.

There are no planarity outliers.

5.2 Too-close contacts (i)

In the following table, the Non-H and H(model) columns list the number of non-hydrogen atoms and hydrogen atoms in the chain respectively. The H(added) column lists the number of hydrogen atoms added and optimized by MolProbity. The Clashes column lists the number of clashes within the asymmetric unit, whereas Symm-Clashes lists symmetry related clashes.

Mol	Chain	Non-H	H(model)	H(added)	Clashes	Symm-Clashes
1	A	2442	0	2408	16	0

Continued on next page...

Continued from previous page...

Mol	Chain	Non-H	H(model)	H(added)	Clashes	Symm-Clashes
1	B	2419	0	2352	22	0
1	C	2431	0	2390	22	0
1	D	2420	0	2361	20	0
1	E	2428	0	2379	15	0
1	F	2436	0	2380	21	0
1	G	2422	0	2351	22	0
1	H	2419	0	2349	17	0
1	I	2423	0	2359	17	0
1	J	2410	0	2362	21	0
1	K	2398	0	2312	17	0
1	L	2443	0	2403	15	0
2	D	4	0	6	0	0
2	J	8	0	12	0	0
2	L	4	0	6	1	0
3	A	225	0	0	2	1
3	B	178	0	0	2	0
3	C	165	0	0	4	0
3	D	171	0	0	2	0
3	E	201	0	0	2	2
3	F	180	0	0	4	1
3	G	184	0	0	6	1
3	H	188	0	0	4	2
3	I	164	0	0	0	0
3	J	221	0	0	1	1
3	K	176	0	0	3	0
3	L	199	0	0	1	0
All	All	31359	0	28430	197	4

The all-atom clashscore is defined as the number of clashes found per 1000 atoms (including hydrogen atoms). The all-atom clashscore for this structure is 3.

All (197) close contacts within the same asymmetric unit are listed below, sorted by their clash magnitude.

Atom-1	Atom-2	Interatomic distance (Å)	Clash overlap (Å)
1:K:267:ASN:ND2	3:K:502:HOH:O	2.23	0.72
1:G:360:SER:O	3:G:501:HOH:O	2.08	0.71
1:D:399[B]:ARG:NH1	3:D:601:HOH:O	2.24	0.70
1:K:382:GLY:O	3:K:501:HOH:O	2.12	0.67
1:D:296:GLN:OE1	1:D:300:ASN:ND2	2.29	0.66
1:L:311:GLU:OE2	3:L:601:HOH:O	2.14	0.65
1:F:185:ARG:NH1	3:F:502:HOH:O	2.29	0.65

Continued on next page...

Continued from previous page...

Atom-1	Atom-2	Interatomic distance (Å)	Clash overlap (Å)
1:G:305:GLU:OE1	3:G:502:HOH:O	2.16	0.62
1:L:323:ILE:HG21	1:L:331:SER:HB3	1.80	0.62
1:G:369:GLY:N	3:G:501:HOH:O	2.13	0.61
1:H:298:GLY:N	3:H:502:HOH:O	2.24	0.60
1:A:179:LEU:HD12	1:A:221[A]:ILE:HD13	1.84	0.59
1:J:169:ASP:OD2	3:J:601:HOH:O	2.16	0.59
1:J:183:LYS:HD3	1:J:197:ASP:HB3	1.85	0.58
1:G:149:GLU:OE1	1:H:327:ARG:NH1	2.37	0.58
1:G:156:PRO:HB3	1:H:269:THR:HG23	1.87	0.57
1:B:247:ALA:HB3	1:B:357:ALA:HB3	1.87	0.57
1:C:123:LEU:O	3:C:501:HOH:O	2.17	0.57
1:B:125:GLU:O	3:B:501:HOH:O	2.18	0.56
1:F:264:VAL:HG11	1:F:293:ILE:HD13	1.87	0.56
1:J:203:LYS:HD3	1:J:417:LEU:HD23	1.88	0.55
1:I:269:THR:HG23	1:J:156:PRO:HB3	1.87	0.55
1:A:368:ARG:NH1	1:J:305:GLU:OE2	2.39	0.55
1:H:243:GLU:OE1	3:H:501:HOH:O	2.18	0.55
1:G:191:ARG:NH1	1:G:409[B]:GLU:OE2	2.39	0.55
1:E:382:GLY:O	3:E:501:HOH:O	2.18	0.54
1:F:232:ILE:HD11	1:F:414:VAL:HG21	1.89	0.54
1:C:232:ILE:HD11	1:C:414:VAL:HG21	1.89	0.54
1:H:247:ALA:HB3	1:H:357:ALA:HB3	1.89	0.54
1:C:368:ARG:NH2	3:C:510:HOH:O	2.41	0.53
1:E:220:LEU:HD11	1:E:248:MET:HB2	1.90	0.53
1:E:383:LYS:NZ	3:E:512:HOH:O	2.42	0.53
1:B:381:ALA:HB3	1:D:188:GLY:HA3	1.89	0.53
1:A:135:GLU:OE1	3:A:501:HOH:O	2.19	0.53
1:G:393:ASN:ND2	3:G:506:HOH:O	2.39	0.53
1:C:156:PRO:HB3	1:D:269:THR:HG23	1.90	0.52
1:G:323:ILE:HG21	1:G:331:SER:HB3	1.92	0.52
1:G:232:ILE:HD11	1:G:414:VAL:HG21	1.90	0.52
1:F:293:ILE:HD12	1:F:300:ASN:HD21	1.75	0.52
1:I:247:ALA:HB3	1:I:357:ALA:HB3	1.92	0.52
1:K:247:ALA:HB3	1:K:357:ALA:HB3	1.92	0.52
1:K:275:ALA:HB2	1:K:375:PRO:HB3	1.90	0.52
1:I:156:PRO:HB3	1:J:269:THR:HG23	1.92	0.51
1:K:269:THR:HG23	1:L:156:PRO:HB3	1.91	0.51
1:D:399[B]:ARG:HD3	3:D:660:HOH:O	2.10	0.51
1:G:220:LEU:HD11	1:G:248:MET:HB2	1.92	0.51
1:C:185:ARG:HH12	1:C:191:ARG:HB2	1.76	0.51
1:D:247:ALA:HB3	1:D:357:ALA:HB3	1.93	0.51

Continued on next page...

Continued from previous page...

Atom-1	Atom-2	Interatomic distance (Å)	Clash overlap (Å)
1:I:134:LEU:HD21	1:I:167[B]:GLU:HG3	1.92	0.51
1:B:362:GLY:HA2	1:B:366:ILE:O	2.11	0.51
1:C:259:GLN:NE2	3:C:503:HOH:O	2.32	0.51
1:D:220:LEU:HD11	1:D:248:MET:HB2	1.92	0.50
1:A:203:LYS:NZ	3:A:515:HOH:O	2.45	0.50
1:D:379:PHE:CD2	1:F:188:GLY:HA3	2.46	0.50
1:E:143:LEU:HG	1:E:163:LEU:HD22	1.93	0.50
1:A:199:ALA:HB3	1:A:417:LEU:HD13	1.94	0.50
1:G:353:TRP:CZ2	1:G:387:ILE:HG21	2.46	0.50
1:C:247:ALA:HB3	1:C:357:ALA:HB3	1.94	0.50
1:E:362:GLY:HA2	1:E:366:ILE:O	2.12	0.50
1:K:353:TRP:CZ2	1:K:387:ILE:HG21	2.46	0.50
1:K:220:LEU:HD11	1:K:248:MET:HB2	1.93	0.50
1:K:156:PRO:HB3	1:L:269:THR:HG23	1.94	0.50
1:I:179:LEU:HD12	1:I:221:ILE:CD1	2.42	0.49
1:H:275:ALA:HB2	1:H:375:PRO:HB3	1.93	0.49
1:L:220:LEU:HD11	1:L:248:MET:HB2	1.93	0.49
1:D:179:LEU:HD12	1:D:221:ILE:CD1	2.42	0.49
1:D:267:ASN:ND2	1:D:269:THR:H	2.10	0.49
1:J:199:ALA:HB3	1:J:417:LEU:HD13	1.95	0.49
1:D:276:ILE:HD13	1:D:286:PRO:HA	1.95	0.49
1:A:362:GLY:HA3	1:J:303:ASN:O	2.13	0.49
1:E:158:LEU:HD21	1:E:161:ILE:HD11	1.93	0.49
1:F:388:GLU:OE2	3:F:501:HOH:O	2.20	0.49
1:H:232:ILE:HD11	1:H:414:VAL:HG21	1.95	0.49
1:K:199:ALA:HA	1:K:232:ILE:HD13	1.95	0.48
1:L:158:LEU:HD21	1:L:161:ILE:HD11	1.94	0.48
1:G:275:ALA:HB2	1:G:375:PRO:HB3	1.96	0.48
1:L:310:ASN:N	1:L:310:ASN:HD22	2.11	0.48
1:A:247:ALA:HB3	1:A:357:ALA:HB3	1.96	0.48
1:J:158:LEU:HD21	1:J:161:ILE:HD11	1.95	0.48
1:F:399:ARG:NH1	3:F:517:HOH:O	2.46	0.48
1:H:412:LYS:O	1:H:415[B]:GLU:HG3	2.15	0.47
1:B:353:TRP:CZ2	1:B:387:ILE:HG21	2.50	0.47
1:K:179:LEU:HD12	1:K:221:ILE:CD1	2.45	0.47
1:B:249:THR:HG21	1:B:373:SER:HB2	1.95	0.47
1:G:247:ALA:HB3	1:G:357:ALA:HB3	1.96	0.47
1:C:269:THR:HG23	1:D:156:PRO:HB3	1.96	0.47
1:C:405:LYS:HB2	1:L:297:TRP:CH2	2.50	0.47
1:J:247:ALA:HB3	1:J:357:ALA:HB3	1.95	0.47
1:B:226:ALA:HB1	1:B:247:ALA:HB1	1.97	0.47

Continued on next page...

Continued from previous page...

Atom-1	Atom-2	Interatomic distance (Å)	Clash overlap (Å)
1:J:143:LEU:HG	1:J:163:LEU:HD22	1.97	0.47
1:E:247:ALA:HB3	1:E:357:ALA:HB3	1.96	0.47
1:F:220:LEU:HD11	1:F:248:MET:HB2	1.96	0.47
1:C:357:ALA:HA	1:C:373:SER:HA	1.97	0.46
1:E:269:THR:HG23	1:F:156:PRO:HB3	1.97	0.46
1:E:156:PRO:HB3	1:F:269:THR:HG23	1.97	0.46
1:F:353:TRP:CZ2	1:F:387:ILE:HG21	2.51	0.46
1:C:362:GLY:HA2	1:C:366:ILE:O	2.16	0.46
1:D:362:GLY:HA2	1:D:366:ILE:O	2.15	0.46
1:I:362:GLY:HA2	1:I:366:ILE:O	2.15	0.46
1:J:353:TRP:CZ2	1:J:387:ILE:HG21	2.51	0.46
1:I:158:LEU:HD21	1:I:161:ILE:HD11	1.96	0.46
1:B:158:LEU:HD21	1:B:161:ILE:HD11	1.96	0.46
1:A:323:ILE:HD13	1:B:149:GLU:HG2	1.97	0.46
1:I:304:ASP:O	1:I:308:ILE:HG13	2.15	0.46
1:I:191:ARG:NH1	1:I:409:GLU:OE2	2.46	0.46
1:G:191:ARG:O	1:G:195:MET:HG3	2.14	0.46
1:E:199:ALA:HB3	1:E:417:LEU:HD13	1.97	0.45
1:K:158:LEU:HD21	1:K:161:ILE:HD11	1.98	0.45
1:K:226:ALA:HB1	1:K:247:ALA:HB1	1.97	0.45
1:E:199:ALA:CB	1:E:417:LEU:HD13	2.47	0.45
1:B:143:LEU:HG	1:B:163:LEU:HD22	1.98	0.45
1:F:226:ALA:HB1	1:F:247:ALA:HB1	1.98	0.45
1:J:200:ALA:HA	1:J:417:LEU:HD22	1.97	0.45
1:I:221:ILE:HG13	1:I:230:ALA:HA	1.98	0.45
1:G:269:THR:HG23	1:H:156:PRO:HB3	1.99	0.45
1:J:199:ALA:HA	1:J:232:ILE:HD13	1.99	0.45
1:B:220:LEU:HD13	1:B:342:HIS:NE2	2.32	0.45
1:C:191:ARG:HH22	1:C:409:GLU:CD	2.20	0.45
1:D:275:ALA:HB2	1:D:375:PRO:HB3	1.99	0.45
1:B:346:TRP:CD1	1:B:382:GLY:HA2	2.52	0.44
1:G:357:ALA:HA	1:G:373:SER:HA	1.98	0.44
1:G:185:ARG:NH2	3:G:516:HOH:O	2.49	0.44
1:L:247:ALA:HB3	1:L:357:ALA:HB3	2.00	0.44
1:J:220:LEU:HD11	1:J:248:MET:HB2	1.99	0.44
1:F:179:LEU:HD12	1:F:221:ILE:CD1	2.46	0.44
1:F:191:ARG:O	1:F:195:MET:HG3	2.16	0.44
1:H:362:GLY:HA2	1:H:366:ILE:O	2.17	0.44
1:A:179:LEU:HD12	1:A:221[A]:ILE:CD1	2.47	0.44
1:B:220:LEU:HD11	1:B:248:MET:HB2	2.00	0.44
1:C:327:ARG:NH1	1:D:149:GLU:OE1	2.51	0.44

Continued on next page...

Continued from previous page...

Atom-1	Atom-2	Interatomic distance (Å)	Clash overlap (Å)
1:F:247:ALA:HB3	1:F:357:ALA:HB3	2.00	0.44
1:H:231:MET:HE2	1:H:232:ILE:HG13	1.99	0.44
1:A:357:ALA:HA	1:A:373:SER:HA	1.99	0.44
1:K:399:ARG:NH1	3:K:517:HOH:O	2.51	0.44
1:G:226:ALA:HB1	1:G:247:ALA:HB1	2.00	0.44
1:H:399[A]:ARG:HG2	3:H:570:HOH:O	2.18	0.44
1:J:264:VAL:HG11	1:J:293:ILE:HD13	1.99	0.44
1:C:323:ILE:HG21	1:C:331:SER:HB3	1.99	0.43
1:I:220:LEU:HD13	1:I:342:HIS:NE2	2.34	0.43
1:C:143:LEU:HG	1:C:163:LEU:HD22	2.00	0.43
1:G:399:ARG:NH1	3:G:517:HOH:O	2.51	0.43
1:A:156:PRO:HB3	1:B:269:THR:HG23	2.01	0.43
1:F:356:MET:O	1:F:373:SER:HA	2.19	0.43
1:G:149:GLU:HG2	1:H:323:ILE:HD13	1.99	0.43
1:H:226:ALA:HB1	1:H:247:ALA:HB1	1.99	0.43
1:I:226:ALA:HB1	1:I:247:ALA:HB1	2.00	0.43
1:C:362:GLY:HA3	1:L:303:ASN:O	2.19	0.43
1:A:368:ARG:HH22	1:J:305:GLU:HB2	1.84	0.42
1:C:304:ASP:O	1:C:308:ILE:HG13	2.19	0.42
1:A:327:ARG:NH1	1:B:149:GLU:OE1	2.52	0.42
1:B:221:ILE:HG13	1:B:230:ALA:HA	2.00	0.42
1:D:273:ARG:HA	1:D:353:TRP:CD2	2.54	0.42
1:C:252:ASP:OD2	1:C:280:HIS:ND1	2.46	0.42
1:C:411:LYS:NZ	3:C:522:HOH:O	2.53	0.42
1:E:200:ALA:HA	1:E:417:LEU:HD22	2.00	0.42
1:F:275:ALA:HB2	1:F:375:PRO:HB3	2.01	0.42
1:I:353:TRP:CZ2	1:I:387:ILE:HG21	2.55	0.42
1:E:323:ILE:HD13	1:F:149:GLU:HG2	2.01	0.42
1:L:357:ALA:HA	1:L:373:SER:HA	2.02	0.42
1:I:232:ILE:HD11	1:I:414:VAL:HG21	2.02	0.42
1:E:357:ALA:HA	1:E:373:SER:HA	2.02	0.41
1:K:357:ALA:HA	1:K:373:SER:HA	2.02	0.41
1:B:199:ALA:HA	1:B:232:ILE:HD13	2.01	0.41
1:C:356:MET:O	1:C:373:SER:HA	2.20	0.41
1:D:221:ILE:HG13	1:D:230:ALA:HA	2.03	0.41
1:G:264:VAL:HG11	1:G:293[A]:ILE:HD13	2.02	0.41
1:G:327:ARG:NH1	1:H:149:GLU:OE2	2.48	0.41
1:D:153:GLY:HA3	1:D:155:PHE:CE2	2.56	0.41
1:K:359:PRO:HD3	1:K:371:TRP:NE1	2.35	0.41
1:C:264:VAL:HG11	1:C:293:ILE:HD13	2.02	0.41
1:F:393:ASN:ND2	3:F:523:HOH:O	2.53	0.41

Continued on next page...

Continued from previous page...

Atom-1	Atom-2	Interatomic distance (Å)	Clash overlap (Å)
1:H:199:ALA:CB	1:H:232:ILE:HD13	2.50	0.41
1:K:153:GLY:HA3	1:K:155:PHE:CE2	2.56	0.41
1:K:323:ILE:HD13	1:L:149:GLU:HG2	2.03	0.41
1:B:275:ALA:HB2	1:B:375:PRO:HB3	2.02	0.41
1:D:179:LEU:HD12	1:D:221:ILE:HD13	2.02	0.41
1:L:388:GLU:HG2	2:L:501:EDO:O2	2.20	0.41
1:A:275:ALA:HB2	1:A:375:PRO:HB3	2.02	0.41
1:C:306:GLN:HG3	1:C:310:ASN:ND2	2.35	0.41
1:J:362:GLY:HA2	1:J:366:ILE:O	2.21	0.41
1:I:275:ALA:HB2	1:I:375:PRO:HB3	2.02	0.41
1:B:94:LEU:HD13	1:B:128:PRO:HG3	2.01	0.41
1:E:323:ILE:HG21	1:E:331:SER:HB3	2.02	0.41
1:F:131:LEU:HD23	1:F:131:LEU:HA	1.92	0.41
1:H:156:PRO:HD2	3:H:636:HOH:O	2.21	0.41
1:I:143:LEU:HG	1:I:163:LEU:HD22	2.03	0.41
1:J:232:ILE:HD11	1:J:414:VAL:HG21	2.03	0.41
1:L:138:GLU:H	1:L:138:GLU:CD	2.24	0.41
1:B:357:ALA:HA	1:B:373:SER:HA	2.02	0.40
1:A:149:GLU:OE1	1:B:327:ARG:NH2	2.54	0.40
1:I:327:ARG:NH1	1:J:149:GLU:OE2	2.49	0.40
1:L:172:ARG:HA	1:L:212:GLN:HB3	2.03	0.40
1:B:172[B]:ARG:HD3	3:B:634:HOH:O	2.22	0.40
1:A:308:ILE:HA	1:A:312:PHE:HB3	2.02	0.40
1:D:357:ALA:HA	1:D:373:SER:HA	2.03	0.40
1:F:357:ALA:HA	1:F:373:SER:HA	2.03	0.40
1:J:399[A]:ARG:HD3	1:J:399[A]:ARG:HA	1.89	0.40

All (4) symmetry-related close contacts are listed below. The label for Atom-2 includes the symmetry operator and encoded unit-cell translations to be applied.

Atom-1	Atom-2	Interatomic distance (Å)	Clash overlap (Å)
3:G:663:HOH:O	3:H:625:HOH:O[1_455]	2.15	0.05
3:E:682:HOH:O	3:H:684:HOH:O[1_545]	2.15	0.05
3:A:693:HOH:O	3:J:768:HOH:O[1_455]	2.15	0.05
3:E:600:HOH:O	3:F:653:HOH:O[1_655]	2.15	0.05

5.3 Torsion angles (i)

5.3.1 Protein backbone (i)

In the following table, the Percentiles column shows the percent Ramachandran outliers of the chain as a percentile score with respect to all X-ray entries followed by that with respect to entries of similar resolution.

The Analysed column shows the number of residues for which the backbone conformation was analysed, and the total number of residues.

Mol	Chain	Analysed	Favoured	Allowed	Outliers	Percentiles	
1	A	327/444 (74%)	325 (99%)	2 (1%)	0	100	100
1	B	326/444 (73%)	324 (99%)	2 (1%)	0	100	100
1	C	326/444 (73%)	323 (99%)	3 (1%)	0	100	100
1	D	325/444 (73%)	324 (100%)	1 (0%)	0	100	100
1	E	325/444 (73%)	323 (99%)	2 (1%)	0	100	100
1	F	327/444 (74%)	326 (100%)	1 (0%)	0	100	100
1	G	326/444 (73%)	323 (99%)	3 (1%)	0	100	100
1	H	326/444 (73%)	324 (99%)	2 (1%)	0	100	100
1	I	326/444 (73%)	323 (99%)	3 (1%)	0	100	100
1	J	320/444 (72%)	319 (100%)	1 (0%)	0	100	100
1	K	324/444 (73%)	322 (99%)	2 (1%)	0	100	100
1	L	326/444 (73%)	322 (99%)	4 (1%)	0	100	100
All	All	3904/5328 (73%)	3878 (99%)	26 (1%)	0	100	100

There are no Ramachandran outliers to report.

5.3.2 Protein sidechains (i)

In the following table, the Percentiles column shows the percent sidechain outliers of the chain as a percentile score with respect to all X-ray entries followed by that with respect to entries of similar resolution.

The Analysed column shows the number of residues for which the sidechain conformation was analysed, and the total number of residues.

Mol	Chain	Analysed	Rotameric	Outliers	Percentiles	
1	A	251/366 (69%)	247 (98%)	4 (2%)	62	59
1	B	245/366 (67%)	244 (100%)	1 (0%)	91	91

Continued on next page...

Continued from previous page...

Mol	Chain	Analysed	Rotameric	Outliers	Percentiles	
1	C	249/366 (68%)	248 (100%)	1 (0%)	91	91
1	D	246/366 (67%)	245 (100%)	1 (0%)	91	91
1	E	249/366 (68%)	246 (99%)	3 (1%)	71	70
1	F	249/366 (68%)	249 (100%)	0	100	100
1	G	246/366 (67%)	243 (99%)	3 (1%)	71	70
1	H	246/366 (67%)	244 (99%)	2 (1%)	81	82
1	I	246/366 (67%)	244 (99%)	2 (1%)	81	82
1	J	247/366 (68%)	244 (99%)	3 (1%)	71	70
1	K	241/366 (66%)	238 (99%)	3 (1%)	71	70
1	L	251/366 (69%)	250 (100%)	1 (0%)	91	91
All	All	2966/4392 (68%)	2942 (99%)	24 (1%)	81	82

All (24) residues with a non-rotameric sidechain are listed below:

Mol	Chain	Res	Type
1	A	159[A]	GLU
1	A	159[B]	GLU
1	A	195	MET
1	A	403	SER
1	B	195	MET
1	C	195	MET
1	D	403	SER
1	E	193	ASP
1	E	195	MET
1	E	403	SER
1	G	185	ARG
1	G	403	SER
1	G	415	GLU
1	H	195	MET
1	H	403	SER
1	I	159	GLU
1	I	195	MET
1	J	195	MET
1	J	416	ASN
1	J	417	LEU
1	K	159	GLU
1	K	195	MET
1	K	403	SER
1	L	403	SER

Some sidechains can be flipped to improve hydrogen bonding and reduce clashes. All (4) such sidechains are listed below:

Mol	Chain	Res	Type
1	A	108	GLN
1	D	267	ASN
1	G	244	ASN
1	L	310	ASN

5.3.3 RNA (i)

There are no RNA molecules in this entry.

5.4 Non-standard residues in protein, DNA, RNA chains (i)

There are no non-standard protein/DNA/RNA residues in this entry.

5.5 Carbohydrates (i)

There are no carbohydrates in this entry.

5.6 Ligand geometry (i)

4 ligands are modelled in this entry.

In the following table, the Counts columns list the number of bonds (or angles) for which Mogul statistics could be retrieved, the number of bonds (or angles) that are observed in the model and the number of bonds (or angles) that are defined in the Chemical Component Dictionary. The Link column lists molecule types, if any, to which the group is linked. The Z score for a bond length (or angle) is the number of standard deviations the observed value is removed from the expected value. A bond length (or angle) with $|Z| > 2$ is considered an outlier worth inspection. RMSZ is the root-mean-square of all Z scores of the bond lengths (or angles).

Mol	Type	Chain	Res	Link	Bond lengths			Bond angles		
					Counts	RMSZ	$\#\|Z\| > 2$	Counts	RMSZ	$\#\|Z\| > 2$
2	EDO	J	502	-	3,3,3	0.47	0	2,2,2	0.56	0
2	EDO	J	501	-	3,3,3	0.45	0	2,2,2	0.34	0
2	EDO	L	501	-	3,3,3	0.44	0	2,2,2	0.49	0
2	EDO	D	501	-	3,3,3	0.40	0	2,2,2	0.43	0

In the following table, the Chirals column lists the number of chiral outliers, the number of chiral centers analysed, the number of these observed in the model and the number defined in the Chemical Component Dictionary. Similar counts are reported in the Torsion and Rings columns.

'-' means no outliers of that kind were identified.

Mol	Type	Chain	Res	Link	Chirals	Torsions	Rings
2	EDO	J	502	-	-	1/1/1/1	-
2	EDO	J	501	-	-	0/1/1/1	-
2	EDO	L	501	-	-	0/1/1/1	-
2	EDO	D	501	-	-	0/1/1/1	-

There are no bond length outliers.

There are no bond angle outliers.

There are no chirality outliers.

All (1) torsion outliers are listed below:

Mol	Chain	Res	Type	Atoms
2	J	502	EDO	O1-C1-C2-O2

There are no ring outliers.

1 monomer is involved in 1 short contact:

Mol	Chain	Res	Type	Clashes	Symm-Clashes
2	L	501	EDO	1	0

5.7 Other polymers (i)

There are no such residues in this entry.

5.8 Polymer linkage issues (i)

There are no chain breaks in this entry.

6 Fit of model and data (i)

6.1 Protein, DNA and RNA chains (i)

In the following table, the column labelled '#RSRZ> 2' contains the number (and percentage) of RSRZ outliers, followed by percent RSRZ outliers for the chain as percentile scores relative to all X-ray entries and entries of similar resolution. The OWAB column contains the minimum, median, 95th percentile and maximum values of the occupancy-weighted average B-factor per residue. The column labelled 'Q< 0.9' lists the number of (and percentage) of residues with an average occupancy less than 0.9.

Mol	Chain	Analysed	<RSRZ>	#RSRZ>2			OWAB(Å^2)	Q<0.9
1	A	326/444 (73%)	-0.15	3 (0%)	84	86	17, 29, 47, 72	0
1	B	326/444 (73%)	-0.04	5 (1%)	73	76	21, 33, 54, 79	0
1	C	326/444 (73%)	-0.11	7 (2%)	63	67	18, 29, 54, 72	0
1	D	325/444 (73%)	-0.01	8 (2%)	57	61	22, 32, 56, 82	0
1	E	326/444 (73%)	-0.06	5 (1%)	73	76	19, 30, 50, 71	0
1	F	326/444 (73%)	-0.02	4 (1%)	79	81	23, 33, 53, 83	0
1	G	326/444 (73%)	-0.05	5 (1%)	73	76	23, 32, 52, 76	0
1	H	326/444 (73%)	-0.09	3 (0%)	84	86	19, 30, 53, 78	0
1	I	326/444 (73%)	0.01	10 (3%)	49	53	22, 32, 58, 79	0
1	J	323/444 (72%)	-0.09	5 (1%)	73	76	19, 30, 52, 69	0
1	K	326/444 (73%)	0.03	7 (2%)	63	67	20, 33, 57, 85	0
1	L	326/444 (73%)	-0.17	2 (0%)	89	91	18, 30, 48, 64	0
All	All	3908/5328 (73%)	-0.06	64 (1%)	72	74	17, 31, 53, 85	0

All (64) RSRZ outliers are listed below:

Mol	Chain	Res	Type	RSRZ
1	K	417	LEU	6.7
1	E	297	TRP	5.6
1	D	417	LEU	5.3
1	F	417	LEU	5.1
1	H	297	TRP	4.7
1	K	295	GLY	4.5
1	B	417	LEU	4.5
1	F	418	LEU	4.4
1	I	417	LEU	4.4
1	E	417	LEU	4.3
1	A	297	TRP	4.2

Continued on next page...

Continued from previous page...

Mol	Chain	Res	Type	RSRZ
1	D	418	LEU	4.2
1	A	418	LEU	4.1
1	E	418	LEU	4.0
1	I	194	VAL	3.9
1	E	295	GLY	3.7
1	I	418	LEU	3.5
1	C	297	TRP	3.5
1	B	199	ALA	3.4
1	J	418	LEU	3.3
1	C	192	ALA	3.2
1	G	417	LEU	3.2
1	F	186	GLY	3.2
1	I	184	PRO	3.1
1	B	265	GLY	3.1
1	B	295	GLY	3.1
1	D	188	GLY	3.1
1	F	188	GLY	3.0
1	G	186	GLY	2.9
1	C	295	GLY	2.8
1	C	418	LEU	2.7
1	D	184	PRO	2.7
1	J	192	ALA	2.7
1	B	418	LEU	2.6
1	I	186	GLY	2.6
1	I	189	MET	2.6
1	H	418	LEU	2.6
1	A	416	ASN	2.6
1	D	295	GLY	2.5
1	D	297	TRP	2.5
1	J	195	MET	2.5
1	K	296	GLN	2.5
1	K	199	ALA	2.4
1	C	191	ARG	2.4
1	E	416	ASN	2.4
1	G	297	TRP	2.4
1	I	416	ASN	2.3
1	C	417	LEU	2.3
1	K	297	TRP	2.3
1	G	188	GLY	2.3
1	L	418	LEU	2.3
1	I	295	GLY	2.3
1	K	192	ALA	2.2

Continued on next page...

Continued from previous page...

Mol	Chain	Res	Type	RSRZ
1	D	296	GLN	2.2
1	D	187	PRO	2.1
1	H	417	LEU	2.1
1	I	187	PRO	2.1
1	L	297	TRP	2.1
1	K	418	LEU	2.1
1	G	418	LEU	2.1
1	J	295	GLY	2.1
1	I	265	GLY	2.0
1	J	417	LEU	2.0
1	C	194	VAL	2.0

6.2 Non-standard residues in protein, DNA, RNA chains (i)

There are no non-standard protein/DNA/RNA residues in this entry.

6.3 Carbohydrates (i)

There are no carbohydrates in this entry.

6.4 Ligands (i)

In the following table, the Atoms column lists the number of modelled atoms in the group and the number defined in the chemical component dictionary. The B-factors column lists the minimum, median, 95th percentile and maximum values of B factors of atoms in the group. The column labelled 'Q< 0.9' lists the number of atoms with occupancy less than 0.9.

Mol	Type	Chain	Res	Atoms	RSCC	RSR	B-factors($Å^2$)	Q<0.9
2	EDO	J	501	4/4	0.80	0.11	52,53,56,57	0
2	EDO	J	502	4/4	0.85	0.11	47,47,52,52	0
2	EDO	L	501	4/4	0.88	0.17	53,55,57,60	0
2	EDO	D	501	4/4	0.94	0.12	61,61,62,65	0

6.5 Other polymers (i)

There are no such residues in this entry.
