# Supplementary material for: Naegleria fowleri: Protein structures to facilitate drug discovery for the deadly, pathogenic free-living amoeba
Source: PLoS One. 2021 Mar 24;16(3):e0241738. doi: 10.1371/journal.pone.0241738 (PMC7990177; doi:10.1371/journal.pone.0241738)
Supplement: S1 File — (ZIP) [file pone.0241738.s002.zip › 6uyh_full_validation.pdf]

Full wwPDB X-ray Structure Validation Report (i)

Aug 21, 2020 – 12:38 PM BST

PDB ID	:	6UYH
Title	:	Crystal structure of prolyl-tRNA synthetase from Naegleria fowleri in complex with halofuginone and AMPPNP
Authors	:	Seattle Structural Genomics Center for Infectious Disease (SSGCID)
Deposited on	:	2019-11-13
Resolution	:	1.75 Å(reported)

This is a Full wwPDB X-ray Structure Validation Report for a publicly released PDB entry.

We welcome your comments at *validation@mail.wwpdb.org*
A user guide is available at
https://www.wwpdb.org/validation/2017/XrayValidationReportHelp
with specific help available everywhere you see the (i) symbol.

The following versions of software and data (see references (i)) were used in the production of this report:

MolProbity	:	4.02b-467
Mogul	:	1.8.5 (274361), CSD as541be (2020)
Xtriage (Phenix)	:	1.13
EDS	:	2.13.1
buster-report	:	1.1.7 (2018)
Percentile statistics	:	20191225.v01 (using entries in the PDB archive December 25th 2019)
Refmac	:	5.8.0158
CCP4	:	7.0.044 (Gargrove)
Ideal geometry (proteins)	:	Engh & Huber (2001)
Ideal geometry (DNA, RNA)	:	Parkinson et al. (1996)
Validation Pipeline (wwPDB-VP)	:	2.13.1

1 Overall quality at a glance (i)

The following experimental techniques were used to determine the structure:
X-RAY DIFFRACTION

The reported resolution of this entry is 1.75 Å.

Percentile scores (ranging between 0-100) for global validation metrics of the entry are shown in the following graphic. The table shows the number of entries on which the scores are based.

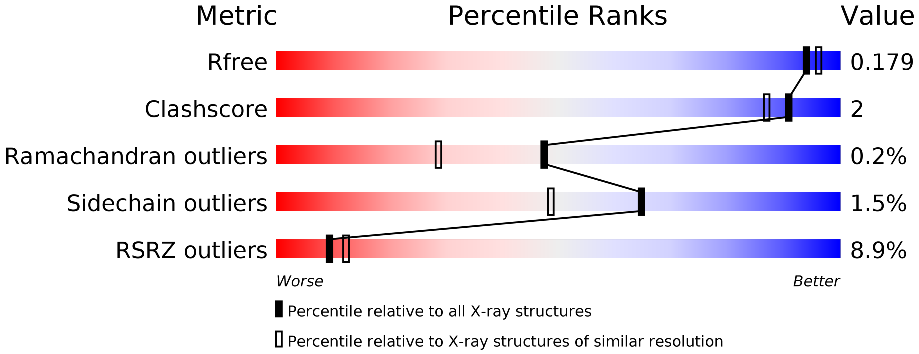

Metric	Whole archive (#Entries)	Similar resolution (#Entries, resolution range(Å))
R_{free}	130704	2340 (1.76-1.76)
Clashscore	141614	2466 (1.76-1.76)
Ramachandran outliers	138981	2437 (1.76-1.76)
Sidechain outliers	138945	2437 (1.76-1.76)
RSRZ outliers	127900	2298 (1.76-1.76)

The table below summarises the geometric issues observed across the polymeric chains and their fit to the electron density. The red, orange, yellow and green segments on the lower bar indicate the fraction of residues that contain outliers for >=3, 2, 1 and 0 types of geometric quality criteria respectively. A grey segment represents the fraction of residues that are not modelled. The numeric value for each fraction is indicated below the corresponding segment, with a dot representing fractions <=5% The upper red bar (where present) indicates the fraction of residues that have poor fit to the electron density. The numeric value is given above the bar.

Mol	Chain	Length	Quality of chain
1	A	513	12%<br>91% 5% •
1	B	513	5%<br>91% 5% •

2 Entry composition (i)

There are 7 unique types of molecules in this entry. The entry contains 9110 atoms, of which 0 are hydrogens and 0 are deuteriums.

In the tables below, the ZeroOcc column contains the number of atoms modelled with zero occupancy, the AltConf column contains the number of residues with at least one atom in alternate conformation and the Trace column contains the number of residues modelled with at most 2 atoms.

- Molecule 1 is a protein called Prolyl-tRNA synthetase.

Mol	Chain	Residues	Atoms					ZeroOcc	AltConf	Trace
1	A	493	Total<br>3979	C<br>2559	N<br>665	O<br>736	S<br>19	0	10	0
1	B	493	Total<br>4024	C<br>2594	N<br>664	O<br>747	S<br>19	0	14	0

- Molecule 2 is PHOSPHOAMINOPHOSPHONIC ACID-ADENYLATE ESTER (three-letter code: ANP) (formula: $C_{10}H_{17}N_6O_{12}P_3$).

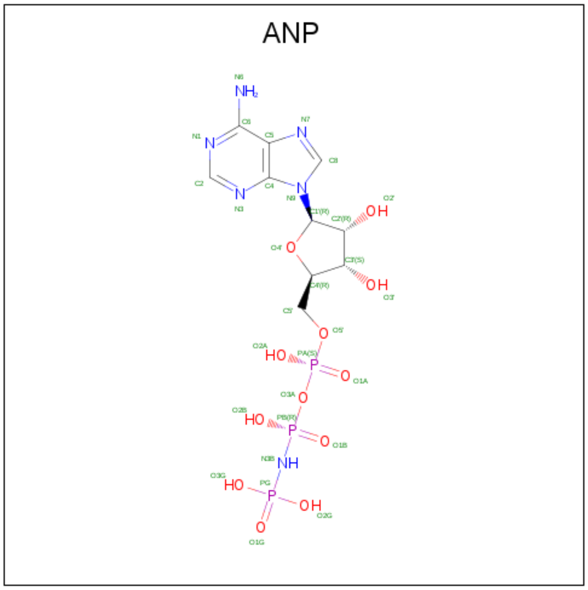

Mol	Chain	Residues	Atoms					ZeroOcc	AltConf
2	A	1	Total<br>31	C<br>10	N<br>6	O<br>12	P<br>3	0	0
2	B	1	Total<br>31	C<br>10	N<br>6	O<br>12	P<br>3	0	0

- Molecule 3 is 7-bromo-6-chloro-3-{3-[(2R,3S)-3-hydroxypiperidin-2-yl]-2-oxopropyl}quinazolin-4(3H)-one (three-letter code: HFG) (formula: $C_{16}H_{17}BrClN_3O_3$).

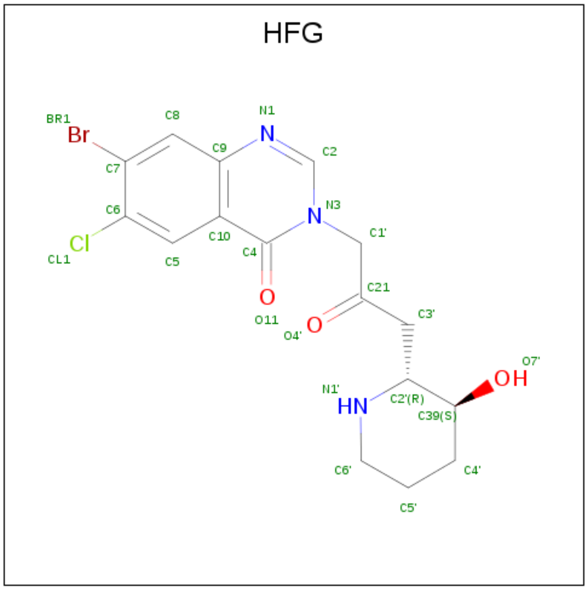

Mol	Chain	Residues	Atoms						ZeroOcc	AltConf
3	A	1	Total 24	Br 1	C 16	Cl 1	N 3	O 3	0	0
3	B	1	Total 24	Br 1	C 16	Cl 1	N 3	O 3	0	0

- Molecule 4 is MAGNESIUM ION (three-letter code: MG) (formula: Mg).

Mol	Chain	Residues	Atoms		ZeroOcc	AltConf
4	B	1	Total 1	Mg 1	0	0
4	A	1	Total 1	Mg 1	0	0

- Molecule 5 is 1,2-ETHANEDIOL (three-letter code: EDO) (formula: $C_2H_6O_2$).

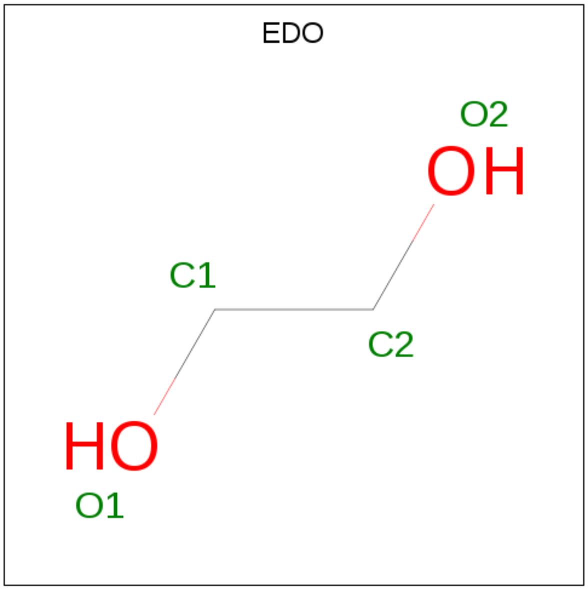

Mol	Chain	Residues	Atoms	ZeroOcc	AltConf
5	A	1	Total C O<br>4 2 2	0	0
5	A	1	Total C O<br>4 2 2	0	0
5	A	1	Total C O<br>4 2 2	0	0
5	A	1	Total C O<br>4 2 2	0	0
5	A	1	Total C O<br>4 2 2	0	0
5	A	1	Total C O<br>4 2 2	0	0
5	A	1	Total C O<br>4 2 2	0	0
5	A	1	Total C O<br>4 2 2	0	0
5	A	1	Total C O<br>4 2 2	0	0
5	A	1	Total C O<br>4 2 2	0	0
5	A	1	Total C O<br>4 2 2	0	0
5	A	1	Total C O<br>4 2 2	0	0
5	B	1	Total C O<br>4 2 2	0	0
5	B	1	Total C O<br>4 2 2	0	0

Continued on next page...

Continued from previous page...

Mol	Chain	Residues	Atoms	ZeroOcc	AltConf
5	B	1	Total C O<br>4 2 2	0	0
5	B	1	Total C O<br>4 2 2	0	0
5	B	1	Total C O<br>4 2 2	0	0
5	B	1	Total C O<br>4 2 2	0	0
5	B	1	Total C O<br>4 2 2	0	0
5	B	1	Total C O<br>4 2 2	0	0
5	B	1	Total C O<br>4 2 2	0	0

- Molecule 6 is SULFATE ION (three-letter code: SO4) (formula: O_4S).

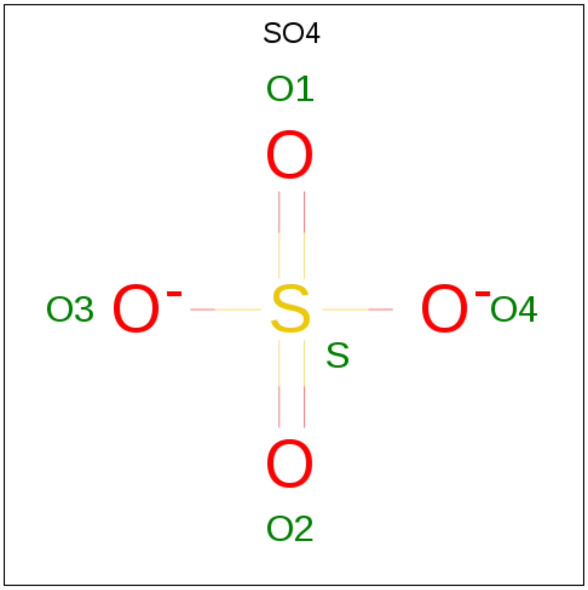

Mol	Chain	Residues	Atoms	ZeroOcc	AltConf
6	A	1	Total O S<br>5 4 1	0	0
6	B	1	Total O S<br>5 4 1	0	0

- Molecule 7 is water.

Mol	Chain	Residues	Atoms		ZeroOcc	AltConf
7	A	462	Total<br>468	O<br>468	0	6
7	B	426	Total<br>433	O<br>433	0	7

3 Residue-property plots (i)

These plots are drawn for all protein, RNA, DNA and oligosaccharide chains in the entry. The first graphic for a chain summarises the proportions of the various outlier classes displayed in the second graphic. The second graphic shows the sequence view annotated by issues in geometry and electron density. Residues are color-coded according to the number of geometric quality criteria for which they contain at least one outlier: green = 0, yellow = 1, orange = 2 and red = 3 or more. A red dot above a residue indicates a poor fit to the electron density (RSRZ > 2). Stretches of 2 or more consecutive residues without any outlier are shown as a green connector. Residues present in the sample, but not in the model, are shown in grey.

- Molecule 1: Prolyl-tRNA synthetase

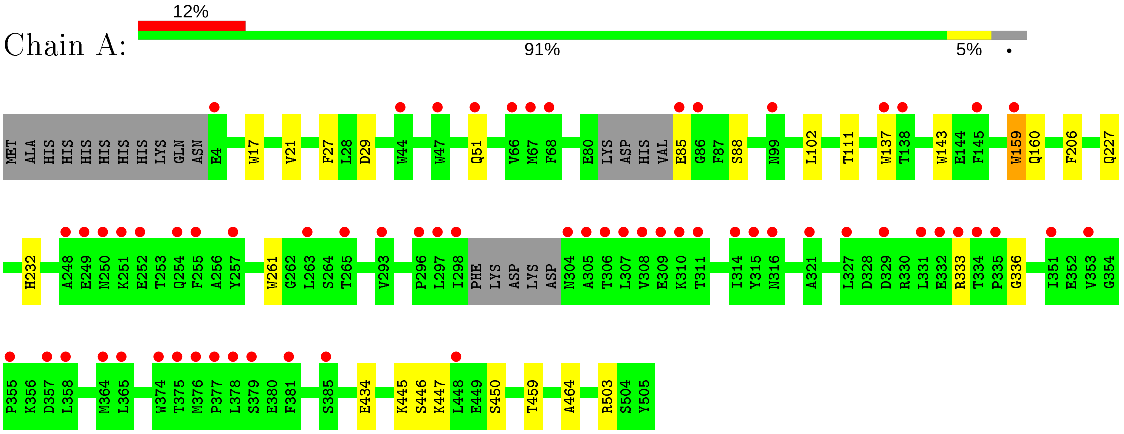

- Molecule 1: Prolyl-tRNA synthetase

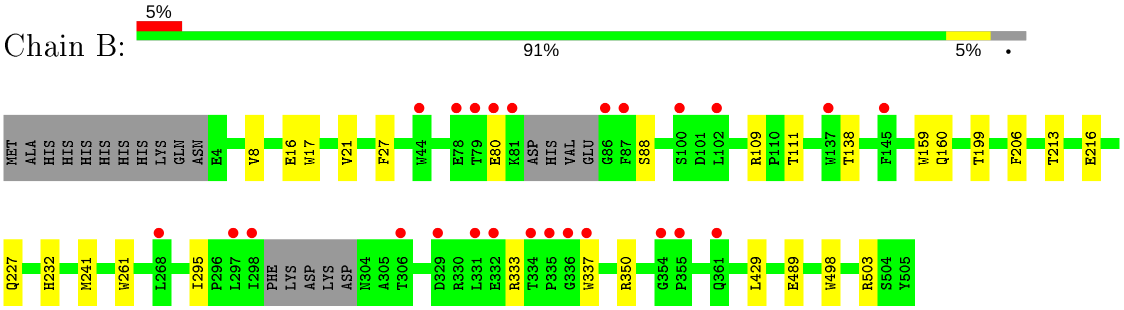

4 Data and refinement statistics (i)

Property	Value	Source
Space group	C 1 2 1	Depositor
Cell constants<br>a, b, c, α, β, γ	157.29Å 66.54Å 112.56Å<br>90.00° 101.74° 90.00°	Depositor
Resolution (Å)	42.66 – 1.75<br>42.66 – 1.75	Depositor<br>EDS
% Data completeness<br>(in resolution range)	99.9 (42.66-1.75)<br>99.9 (42.66-1.75)	Depositor<br>EDS
R_{merge}	0.05	Depositor
R_{sym}	(Not available)	Depositor
$< I/\sigma(I) >$ [1]	2.37 (at 1.75Å)	Xtriage
Refinement program	PHENIX 1.17RC1_3602	Depositor
R, R_{free}	0.154 , 0.180<br>0.154 , 0.179	Depositor<br>DCC
R_{free} test set	1918 reflections (1.67%)	wwPDB-VP
Wilson B-factor (Å^2)	22.3	Xtriage
Anisotropy	0.108	Xtriage
Bulk solvent k_{sol}(e/Å^3), B_{sol}(Å^2)	0.35 , 59.3	EDS
L-test for twinning[2]	$< \|L\| > = 0.50$, $< L^2 > = 0.33$	Xtriage
Estimated twinning fraction	No twinning to report.	Xtriage
F_o,F_c correlation	0.97	EDS
Total number of atoms	9110	wwPDB-VP
Average B, all atoms (Å^2)	30.0	wwPDB-VP

Xtriage's analysis on translational NCS is as follows: *The largest off-origin peak in the Patterson function is 4.94% of the height of the origin peak. No significant pseudotranslation is detected.*

[1]Intensities estimated from amplitudes.

[2]Theoretical values of $< |L| >$, $< L^2 >$ for acentric reflections are 0.5, 0.333 respectively for untwinned datasets, and 0.375, 0.2 for perfectly twinned datasets.

5 Model quality (i)

5.1 Standard geometry (i)

Bond lengths and bond angles in the following residue types are not validated in this section: MG, ANP, EDO, HFG, SO4

The Z score for a bond length (or angle) is the number of standard deviations the observed value is removed from the expected value. A bond length (or angle) with $|Z| > 5$ is considered an outlier worth inspection. RMSZ is the root-mean-square of all Z scores of the bond lengths (or angles).

Mol	Chain	Bond lengths		Bond angles	
		RMSZ	#|Z| >5	RMSZ	#|Z| >5
1	A	0.34	0/4107	0.52	0/5567
1	B	0.37	0/4173	0.54	0/5651
All	All	0.36	0/8280	0.53	0/11218

There are no bond length outliers.

There are no bond angle outliers.

There are no chirality outliers.

There are no planarity outliers.

5.2 Too-close contacts (i)

In the following table, the Non-H and H(model) columns list the number of non-hydrogen atoms and hydrogen atoms in the chain respectively. The H(added) column lists the number of hydrogen atoms added and optimized by MolProbity. The Clashes column lists the number of clashes within the asymmetric unit, whereas Symm-Clashes lists symmetry related clashes.

Mol	Chain	Non-H	H(model)	H(added)	Clashes	Symm-Clashes
1	A	3979	0	3866	15	0
1	B	4024	0	3955	13	0
2	A	31	0	13	0	0
2	B	31	0	13	0	0
3	A	24	0	17	1	0
3	B	24	0	17	0	0
4	A	1	0	0	0	0
4	B	1	0	0	0	0
5	A	48	0	72	1	0
5	B	36	0	54	1	0
6	A	5	0	0	0	0

Continued on next page...

Continued from previous page...

Mol	Chain	Non-H	H(model)	H(added)	Clashes	Symm-Clashes
6	B	5	0	0	1	0
7	A	468	0	0	2	0
7	B	433	0	0	2	0
All	All	9110	0	8007	28	0

The all-atom clashscore is defined as the number of clashes found per 1000 atoms (including hydrogen atoms). The all-atom clashscore for this structure is 2.

All (28) close contacts within the same asymmetric unit are listed below, sorted by their clash magnitude.

Atom-1	Atom-2	Interatomic distance (Å)	Clash overlap (Å)
1:A:85:GLU:N	1:A:88[A]:SER:HG	1.78	0.81
1:A:434:GLU:OE1	7:B:801:HOH:O	2.07	0.71
1:B:8[A]:VAL:HG11	1:B:16:GLU:HG3	1.71	0.70
1:B:8[B]:VAL:HG11	1:B:16:GLU:HG3	1.72	0.69
1:A:445:LYS:NZ	7:B:806:HOH:O	2.31	0.57
1:B:333:ARG:HD3	1:B:337:TRP:CZ3	2.40	0.57
1:A:51[B]:GLN:NE2	7:A:707:HOH:O	2.37	0.57
1:B:80:GLU:HA	1:B:241:MET:SD	2.49	0.52
1:A:206:PHE:HB2	1:A:232:HIS:CE1	2.46	0.51
1:B:109:ARG:NH2	1:B:138[B]:THR:OG1	2.45	0.49
1:B:489:GLU:N	1:B:489:GLU:OE2	2.45	0.49
1:A:446:SER:O	1:A:450:SER:HB3	2.14	0.46
1:A:447:LYS:HB2	1:A:464:ALA:HB2	1.96	0.46
1:A:227:GLN:HB3	7:A:869:HOH:O	2.17	0.45
1:B:199[B]:THR:HG22	1:B:213:THR:HG22	1.98	0.45
1:B:216:GLU:OE1	1:B:227:GLN:NE2	2.40	0.44
1:A:333:ARG:NH2	6:B:706:SO4:O3	2.31	0.44
1:A:336:GLY:HA3	5:B:712:EDO:H22	1.99	0.43
1:B:295:ILE:HD12	1:B:350:ARG:HD2	2.01	0.43
1:B:216:GLU:OE2	1:B:227:GLN:HG3	2.20	0.42
1:A:102:LEU:HD22	5:A:604:EDO:H22	2.03	0.41
1:B:206:PHE:HB2	1:B:232:HIS:CE1	2.55	0.41
1:B:429:LEU:HG	1:B:498:TRP:HB3	2.02	0.41
1:A:17:TRP:CE2	1:A:21:VAL:HG21	2.56	0.41
1:A:459:THR:HG22	1:A:459:THR:O	2.21	0.41
1:A:51[B]:GLN:HG3	1:A:137:TRP:CE2	2.56	0.40
1:A:159:TRP:CH2	3:A:602:HFG:H8	2.56	0.40
1:B:17:TRP:CE2	1:B:21:VAL:HG21	2.57	0.40

There are no symmetry-related clashes.

5.3 Torsion angles (i)

5.3.1 Protein backbone (i)

In the following table, the Percentiles column shows the percent Ramachandran outliers of the chain as a percentile score with respect to all X-ray entries followed by that with respect to entries of similar resolution.

The Analysed column shows the number of residues for which the backbone conformation was analysed, and the total number of residues.

Mol	Chain	Analysed	Favoured	Allowed	Outliers	Percentiles	
1	A	497/513 (97%)	489 (98%)	7 (1%)	1 (0%)	47	29
1	B	502/513 (98%)	490 (98%)	11 (2%)	1 (0%)	47	29
All	All	999/1026 (97%)	979 (98%)	18 (2%)	2 (0%)	47	29

All (2) Ramachandran outliers are listed below:

Mol	Chain	Res	Type
1	A	111	THR
1	B	111	THR

5.3.2 Protein sidechains (i)

In the following table, the Percentiles column shows the percent sidechain outliers of the chain as a percentile score with respect to all X-ray entries followed by that with respect to entries of similar resolution.

The Analysed column shows the number of residues for which the sidechain conformation was analysed, and the total number of residues.

Mol	Chain	Analysed	Rotameric	Outliers	Percentiles	
1	A	426/457 (93%)	419 (98%)	7 (2%)	62	45
1	B	438/457 (96%)	432 (99%)	6 (1%)	67	52
All	All	864/914 (94%)	851 (98%)	13 (2%)	65	49

All (13) residues with a non-rotameric sidechain are listed below:

Mol	Chain	Res	Type
1	A	27	PHE
1	A	29	ASP
1	A	143	TRP
1	A	159	TRP

Continued on next page...

Continued from previous page...

Mol	Chain	Res	Type
1	A	160	GLN
1	A	261	TRP
1	A	503	ARG
1	B	27	PHE
1	B	88	SER
1	B	159	TRP
1	B	160	GLN
1	B	261	TRP
1	B	503	ARG

Some sidechains can be flipped to improve hydrogen bonding and reduce clashes. All (2) such sidechains are listed below:

Mol	Chain	Res	Type
1	A	316	ASN
1	B	147	HIS

5.3.3 RNA (i)

There are no RNA molecules in this entry.

5.4 Non-standard residues in protein, DNA, RNA chains (i)

There are no non-standard protein/DNA/RNA residues in this entry.

5.5 Carbohydrates (i)

There are no monosaccharides in this entry.

5.6 Ligand geometry (i)

Of 29 ligands modelled in this entry, 2 are monoatomic - leaving 27 for Mogul analysis.

In the following table, the Counts columns list the number of bonds (or angles) for which Mogul statistics could be retrieved, the number of bonds (or angles) that are observed in the model and the number of bonds (or angles) that are defined in the Chemical Component Dictionary. The Link column lists molecule types, if any, to which the group is linked. The Z score for a bond length (or angle) is the number of standard deviations the observed value is removed from the expected value. A bond length (or angle) with $|Z| > 2$ is considered an outlier worth inspection. RMSZ is the root-mean-square of all Z scores of the bond lengths (or angles).

Mol	Type	Chain	Res	Link	Bond lengths			Bond angles		
					Counts	RMSZ	#|Z| > 2	Counts	RMSZ	#|Z| > 2
5	EDO	A	613	-	3,3,3	0.47	0	2,2,2	0.32	0
5	EDO	B	713	-	3,3,3	0.44	0	2,2,2	0.55	0
5	EDO	A	609	-	3,3,3	0.47	0	2,2,2	0.36	0
5	EDO	A	604	-	3,3,3	0.58	0	2,2,2	0.29	0
5	EDO	B	712	-	3,3,3	0.45	0	2,2,2	0.33	0
5	EDO	A	612	-	3,3,3	0.52	0	2,2,2	0.15	0
5	EDO	A	606	-	3,3,3	0.44	0	2,2,2	0.36	0
6	SO4	B	706	-	4,4,4	0.14	0	6,6,6	0.09	0
2	ANP	B	703	4	29,33,33	0.72	1 (3%)	31,52,52	1.10	4 (12%)
5	EDO	B	707	-	3,3,3	0.45	0	2,2,2	0.49	0
3	HFG	B	704	-	26,26,26	0.97	1 (3%)	22,37,37	0.99	2 (9%)
5	EDO	B	702	-	3,3,3	0.52	0	2,2,2	0.51	0
5	EDO	A	610	-	3,3,3	0.45	0	2,2,2	0.35	0
3	HFG	A	602	-	26,26,26	0.98	1 (3%)	22,37,37	0.84	1 (4%)
5	EDO	B	710	-	3,3,3	0.50	0	2,2,2	0.19	0
5	EDO	B	709	-	3,3,3	0.42	0	2,2,2	0.38	0
6	SO4	A	608	-	4,4,4	0.15	0	6,6,6	0.04	0
5	EDO	A	616	-	3,3,3	0.45	0	2,2,2	0.42	0
5	EDO	B	701	-	3,3,3	0.45	0	2,2,2	0.27	0
2	ANP	A	601	4	29,33,33	0.82	1 (3%)	31,52,52	1.04	3 (9%)
5	EDO	A	614	-	3,3,3	0.41	0	2,2,2	0.48	0
5	EDO	A	607	-	3,3,3	0.41	0	2,2,2	0.51	0
5	EDO	B	708	-	3,3,3	0.43	0	2,2,2	0.34	0
5	EDO	A	615	-	3,3,3	0.45	0	2,2,2	0.41	0
5	EDO	A	611	-	3,3,3	0.48	0	2,2,2	0.36	0
5	EDO	A	605	-	3,3,3	0.36	0	2,2,2	0.58	0
5	EDO	B	711	-	3,3,3	0.46	0	2,2,2	0.44	0

In the following table, the Chirals column lists the number of chiral outliers, the number of chiral centers analysed, the number of these observed in the model and the number defined in the Chemical Component Dictionary. Similar counts are reported in the Torsion and Rings columns. '-' means no outliers of that kind were identified.

Mol	Type	Chain	Res	Link	Chirals	Torsions	Rings
5	EDO	A	613	-	-	0/1/1/1	-
5	EDO	B	713	-	-	0/1/1/1	-
5	EDO	A	609	-	-	0/1/1/1	-
5	EDO	A	604	-	-	1/1/1/1	-
5	EDO	B	712	-	-	0/1/1/1	-
5	EDO	A	612	-	-	0/1/1/1	-
5	EDO	A	606	-	-	0/1/1/1	-
2	ANP	B	703	4	-	6/14/38/38	0/3/3/3

Continued on next page...

Continued from previous page...

Mol	Type	Chain	Res	Link	Chirals	Torsions	Rings
5	EDO	B	707	-	-	0/1/1/1	-
3	HFG	B	704	-	-	0/8/19/19	0/3/3/3
5	EDO	B	702	-	-	0/1/1/1	-
5	EDO	A	610	-	-	1/1/1/1	-
3	HFG	A	602	-	-	0/8/19/19	0/3/3/3
5	EDO	B	710	-	-	1/1/1/1	-
5	EDO	B	709	-	-	0/1/1/1	-
5	EDO	A	616	-	-	0/1/1/1	-
5	EDO	B	701	-	-	0/1/1/1	-
2	ANP	A	601	4	-	6/14/38/38	0/3/3/3
5	EDO	A	614	-	-	0/1/1/1	-
5	EDO	A	607	-	-	1/1/1/1	-
5	EDO	B	708	-	-	1/1/1/1	-
5	EDO	A	615	-	-	0/1/1/1	-
5	EDO	A	611	-	-	0/1/1/1	-
5	EDO	A	605	-	-	0/1/1/1	-
5	EDO	B	711	-	-	0/1/1/1	-

All (4) bond length outliers are listed below:

Mol	Chain	Res	Type	Atoms	Z	Observed(Å)	Ideal(Å)
3	B	704	HFG	C4-C10	3.21	1.47	1.41
3	A	602	HFG	C4-C10	3.18	1.47	1.41
2	A	601	ANP	PB-O3A	-2.77	1.55	1.59
2	B	703	ANP	PB-O3A	-2.19	1.56	1.59

All (10) bond angle outliers are listed below:

Mol	Chain	Res	Type	Atoms	Z	Observed(^{o})	Ideal(^{o})
3	B	704	HFG	C10-C4-N3	-3.48	113.77	116.15
3	A	602	HFG	C10-C4-N3	-3.48	113.77	116.15
2	B	703	ANP	O3G-PG-O1G	-2.84	106.30	113.45
2	A	601	ANP	O3G-PG-O1G	-2.79	106.43	113.45
2	A	601	ANP	C5-C6-N6	2.44	124.06	120.35
2	B	703	ANP	O2G-PG-O1G	-2.27	107.75	113.45
2	B	703	ANP	C5-C6-N6	2.26	123.79	120.35
3	B	704	HFG	C6'-N1'-C2'	2.23	113.01	111.62
2	B	703	ANP	O5'-PA-O1A	2.18	117.57	109.07
2	A	601	ANP	O2G-PG-O1G	-2.09	108.19	113.45

There are no chirality outliers.

All (17) torsion outliers are listed below:

Mol	Chain	Res	Type	Atoms
2	B	703	ANP	PB-N3B-PG-O1G
2	B	703	ANP	PG-N3B-PB-O1B
2	B	703	ANP	C5'-O5'-PA-O1A
2	A	601	ANP	PB-N3B-PG-O1G
2	A	601	ANP	PG-N3B-PB-O1B
2	A	601	ANP	C5'-O5'-PA-O1A
2	B	703	ANP	C5'-O5'-PA-O2A
2	A	601	ANP	C5'-O5'-PA-O2A
5	A	610	EDO	O1-C1-C2-O2
5	A	604	EDO	O1-C1-C2-O2
2	B	703	ANP	C4'-C5'-O5'-PA
5	B	710	EDO	O1-C1-C2-O2
2	A	601	ANP	C4'-C5'-O5'-PA
5	A	607	EDO	O1-C1-C2-O2
2	B	703	ANP	C5'-O5'-PA-O3A
2	A	601	ANP	C5'-O5'-PA-O3A
5	B	708	EDO	O1-C1-C2-O2

There are no ring outliers.

4 monomers are involved in 4 short contacts:

Mol	Chain	Res	Type	Clashes	Symm-Clashes
5	A	604	EDO	1	0
5	B	712	EDO	1	0
6	B	706	SO4	1	0
3	A	602	HFG	1	0

The following is a two-dimensional graphical depiction of Mogul quality analysis of bond lengths, bond angles, torsion angles, and ring geometry for all instances of the Ligand of Interest. In addition, ligands with molecular weight > 250 and outliers as shown on the validation Tables will also be included. For torsion angles, if less then 5% of the Mogul distribution of torsion angles is within 10 degrees of the torsion angle in question, then that torsion angle is considered an outlier. Any bond that is central to one or more torsion angles identified as an outlier by Mogul will be highlighted in the graph. For rings, the root-mean-square deviation (RMSD) between the ring in question and similar rings identified by Mogul is calculated over all ring torsion angles. If the average RMSD is greater than 60 degrees and the minimal RMSD between the ring in question and any Mogul-identified rings is also greater than 60 degrees, then that ring is considered an outlier. The outliers are highlighted in purple. The color gray indicates Mogul did not find sufficient equivalents in the CSD to analyse the geometry.

Ligand ANP B 703

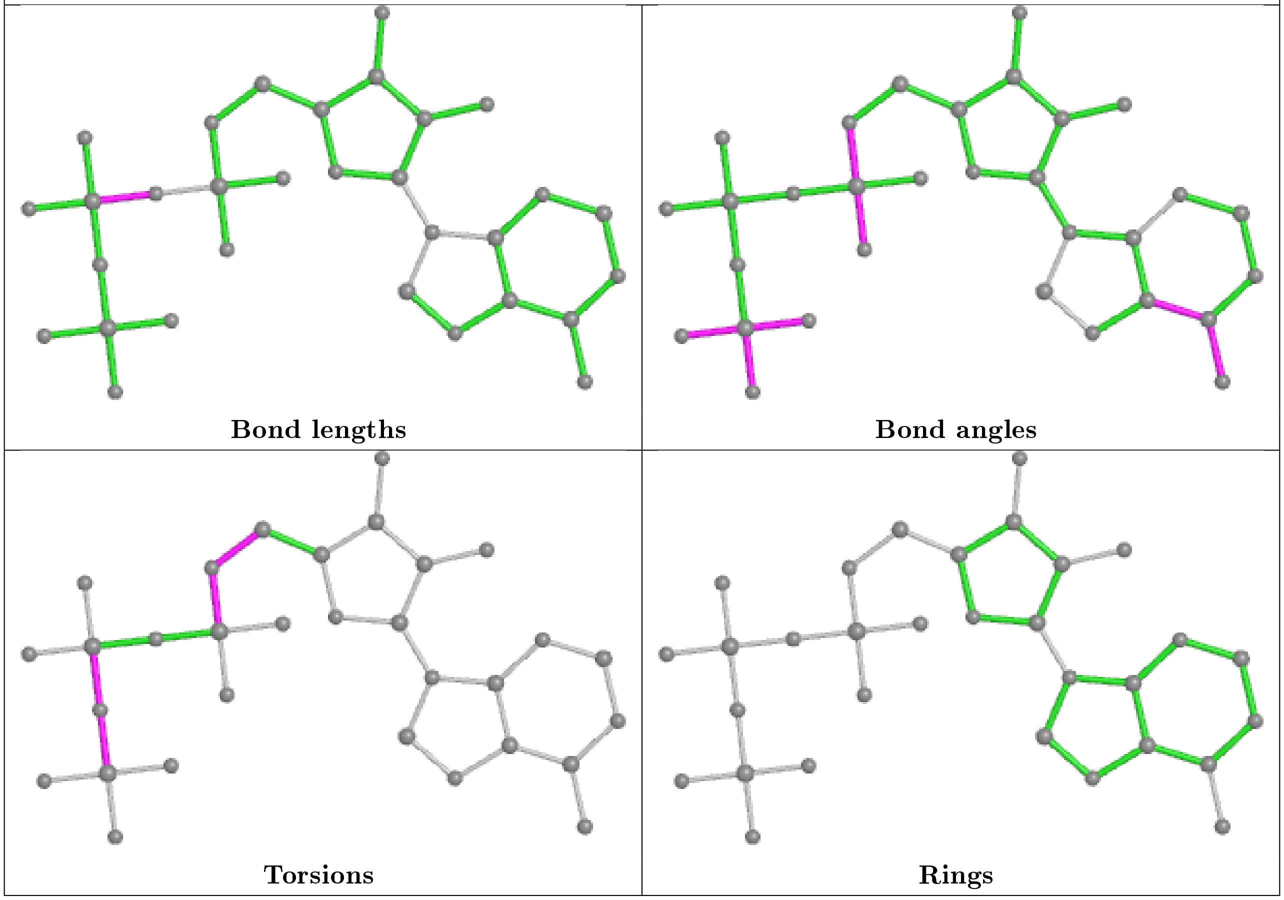

Ligand HFG B 704

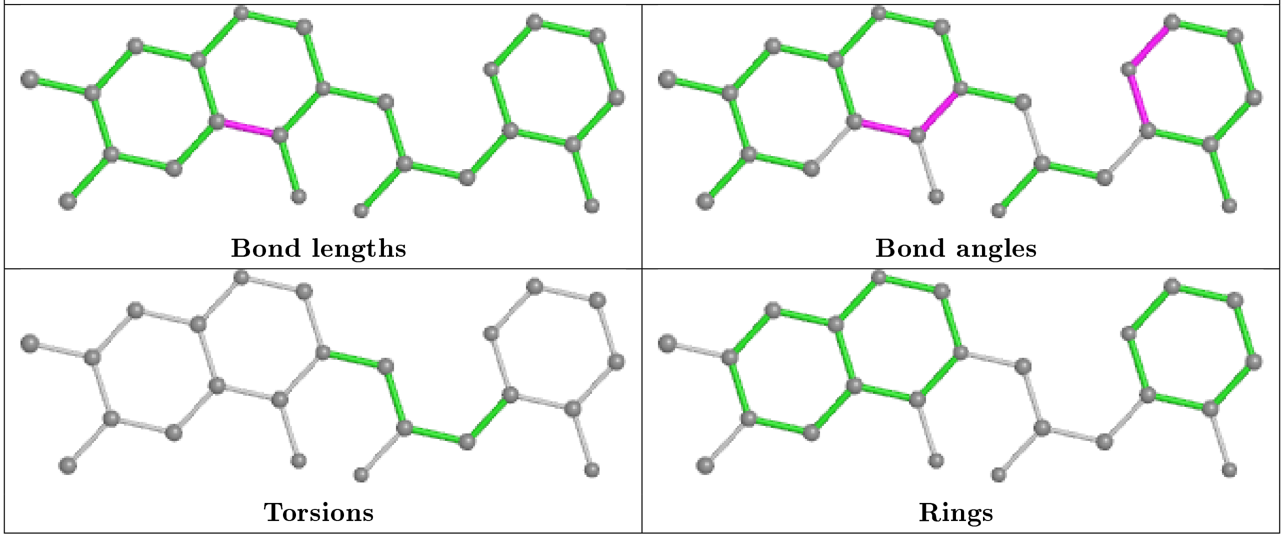

Ligand HFG A 602

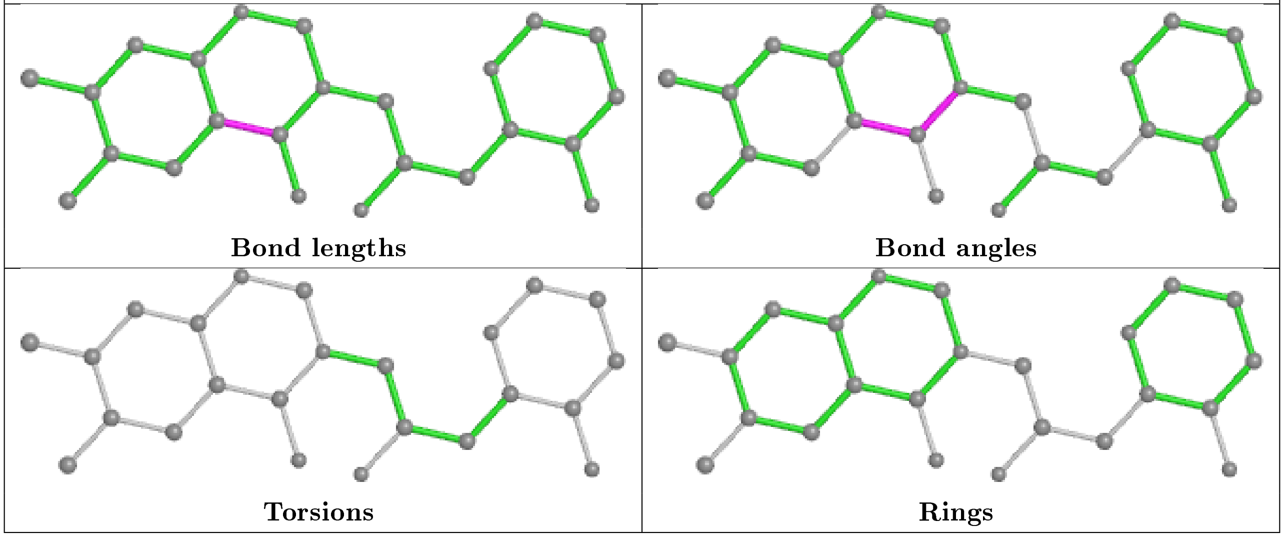

Ligand ANP A 601

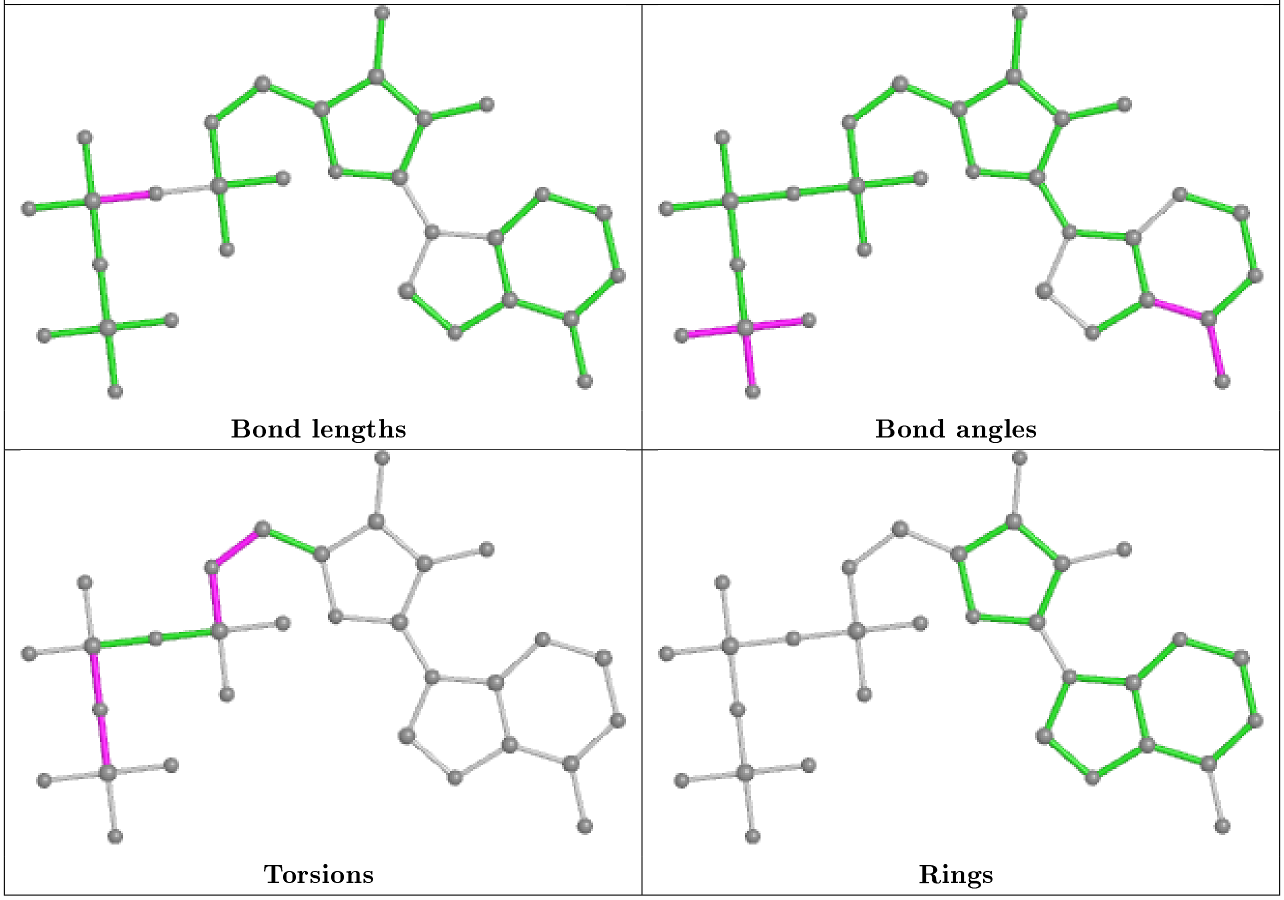

5.7 Other polymers ⓘ

There are no such residues in this entry.

5.8 Polymer linkage issues (i)

There are no chain breaks in this entry.

6 Fit of model and data (i)

6.1 Protein, DNA and RNA chains (i)

In the following table, the column labelled '#RSRZ> 2' contains the number (and percentage) of RSRZ outliers, followed by percent RSRZ outliers for the chain as percentile scores relative to all X-ray entries and entries of similar resolution. The OWAB column contains the minimum, median, 95th percentile and maximum values of the occupancy-weighted average B-factor per residue. The column labelled 'Q< 0.9' lists the number of (and percentage) of residues with an average occupancy less than 0.9.

Mol	Chain	Analysed	<RSRZ>	#RSRZ>2			OWAB(Å^2)	Q<0.9
1	A	493/513 (96%)	0.48	63 (12%)	3	5	16, 28, 63, 91	1 (0%)
1	B	493/513 (96%)	0.20	25 (5%)	28	34	15, 23, 49, 81	0
All	All	986/1026 (96%)	0.34	88 (8%)	9	12	15, 26, 56, 91	1 (0%)

All (88) RSRZ outliers are listed below:

Mol	Chain	Res	Type	RSRZ
1	B	331	LEU	9.8
1	B	335	PRO	6.6
1	B	298	ILE	6.3
1	A	298	ILE	6.1
1	A	248	ALA	5.3
1	A	306	THR	5.1
1	A	250	ASN	5.1
1	B	87	PHE	4.8
1	B	332	GLU	4.7
1	A	316	ASN	4.6
1	A	309	GLU	4.6
1	A	353	VAL	4.5
1	A	378	LEU	4.4
1	B	86	GLY	4.4
1	A	307	LEU	4.3
1	A	297	LEU	4.3
1	B	81	LYS	4.3
1	A	314	ILE	4.2
1	A	381	PHE	4.2
1	B	297	LEU	4.1
1	A	315	TYR	4.1
1	A	308	VAL	3.9
1	A	355	PRO	3.8
1	A	364	MET	3.8

Continued on next page...

Continued from previous page...

Mol	Chain	Res	Type	RSRZ
1	A	327	LEU	3.8
1	B	306	THR	3.7
1	A	305	ALA	3.5
1	B	100	SER	3.5
1	B	334	THR	3.4
1	A	304	ASN	3.2
1	B	79	THR	3.1
1	A	335	PRO	3.0
1	B	355	PRO	3.0
1	A	249	GLU	2.9
1	A	159	TRP	2.9
1	B	80	GLU	2.9
1	B	354	GLY	2.8
1	B	102	LEU	2.8
1	A	333	ARG	2.7
1	A	66	VAL	2.7
1	A	310	LYS	2.7
1	A	251	LYS	2.7
1	A	365	LEU	2.7
1	A	85	GLU	2.7
1	B	44	TRP	2.7
1	A	311	THR	2.6
1	A	145	PHE	2.5
1	B	361	GLN	2.5
1	A	351	ILE	2.5
1	A	358	LEU	2.5
1	A	374	TRP	2.5
1	A	296	PRO	2.5
1	A	4	GLU	2.5
1	A	138	THR	2.4
1	A	254	GLN	2.4
1	A	255	PHE	2.4
1	A	376	MET	2.4
1	A	86	GLY	2.4
1	B	145	PHE	2.3
1	A	332	GLU	2.3
1	A	51[A]	GLN	2.3
1	B	137	TRP	2.3
1	B	336	GLY	2.3
1	B	78	GLU	2.3
1	A	293	VAL	2.2
1	A	377	PRO	2.2

Continued on next page...

Continued from previous page...

Mol	Chain	Res	Type	RSRZ
1	A	331	LEU	2.2
1	A	334	THR	2.2
1	A	379	SER	2.2
1	A	448	LEU	2.2
1	A	329	ASP	2.2
1	A	252	GLU	2.2
1	A	68	PHE	2.1
1	A	263	LEU	2.1
1	A	375	THR	2.1
1	A	67	MET	2.1
1	A	257	TYR	2.1
1	A	47	TRP	2.1
1	A	99	ASN	2.1
1	B	268	LEU	2.1
1	A	357	ASP	2.1
1	B	329	ASP	2.1
1	A	44	TRP	2.1
1	A	137	TRP	2.1
1	A	321	ALA	2.0
1	B	337	TRP	2.0
1	A	385	SER	2.0
1	A	265	THR	2.0

6.2 Non-standard residues in protein, DNA, RNA chains (i)

There are no non-standard protein/DNA/RNA residues in this entry.

6.3 Carbohydrates (i)

There are no monosaccharides in this entry.

6.4 Ligands (i)

In the following table, the Atoms column lists the number of modelled atoms in the group and the number defined in the chemical component dictionary. The B-factors column lists the minimum, median, 95th percentile and maximum values of B factors of atoms in the group. The column labelled 'Q< 0.9' lists the number of atoms with occupancy less than 0.9.

Mol	Type	Chain	Res	Atoms	RSCC	RSR	B-factors(Å^2)	Q<0.9
5	EDO	A	609	4/4	0.75	0.22	77,79,83,84	0

Continued on next page...

Continued from previous page...

Mol	Type	Chain	Res	Atoms	RSCC	RSR	B-factors(Å^2)	Q<0.9
5	EDO	A	613	4/4	0.79	0.11	52,54,55,56	0
5	EDO	A	604	4/4	0.83	0.15	36,38,51,56	0
5	EDO	B	712	4/4	0.83	0.25	57,62,65,68	0
5	EDO	B	711	4/4	0.83	0.22	42,50,59,61	0
5	EDO	A	607	4/4	0.85	0.12	48,54,56,64	0
5	EDO	B	713	4/4	0.88	0.16	37,39,42,45	0
5	EDO	A	605	4/4	0.88	0.16	35,40,41,43	0
5	EDO	A	614	4/4	0.88	0.12	60,61,63,67	0
5	EDO	B	708	4/4	0.89	0.13	46,47,48,49	0
5	EDO	A	611	4/4	0.90	0.23	30,36,46,54	0
5	EDO	B	707	4/4	0.90	0.13	31,38,46,47	0
5	EDO	A	606	4/4	0.90	0.14	53,55,55,59	0
5	EDO	B	702	4/4	0.91	0.14	29,30,34,35	0
6	SO4	B	706	5/5	0.92	0.28	94,96,98,98	0
5	EDO	B	709	4/4	0.92	0.10	47,53,53,56	0
5	EDO	A	616	4/4	0.92	0.19	40,46,53,59	0
5	EDO	A	615	4/4	0.93	0.15	39,40,43,43	0
4	MG	A	603	1/1	0.94	0.04	31,31,31,31	0
2	ANP	B	703	31/31	0.95	0.09	16,27,47,48	0
2	ANP	A	601	31/31	0.95	0.10	19,26,47,52	0
5	EDO	B	710	4/4	0.95	0.14	18,24,44,47	0
5	EDO	A	610	4/4	0.96	0.12	25,36,41,46	0
3	HFG	A	602	24/24	0.96	0.09	16,20,24,38	0
3	HFG	B	704	24/24	0.96	0.08	15,18,25,40	0
5	EDO	A	612	4/4	0.96	0.10	28,36,37,41	0
6	SO4	A	608	5/5	0.96	0.12	66,70,74,75	0
5	EDO	B	701	4/4	0.97	0.15	34,38,39,43	0
4	MG	B	705	1/1	0.99	0.07	25,25,25,25	0

The following is a graphical depiction of the model fit to experimental electron density of all instances of the Ligand of Interest. In addition, ligands with molecular weight > 250 and outliers as shown on the geometry validation Tables will also be included. Each fit is shown from different orientation to approximate a three-dimensional view.

Electron density around ANP B 703:

$2mF_o$-DF_c (at 0.7 rmsd) in gray
mF_o-DF_c (at 3 rmsd) in purple (negative)
and green (positive)

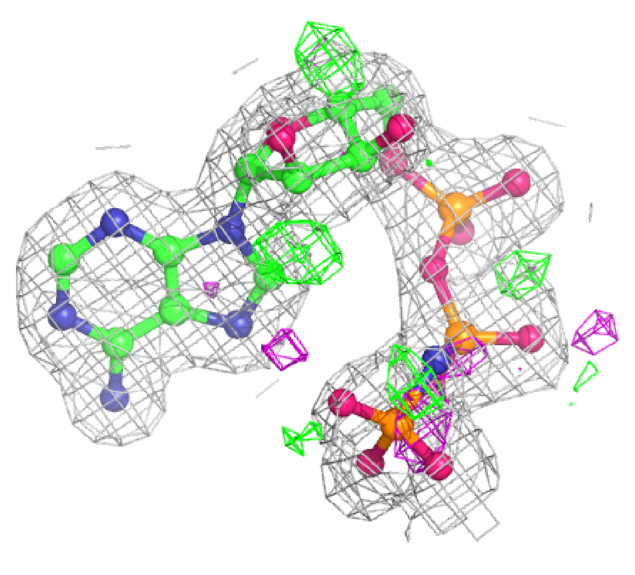

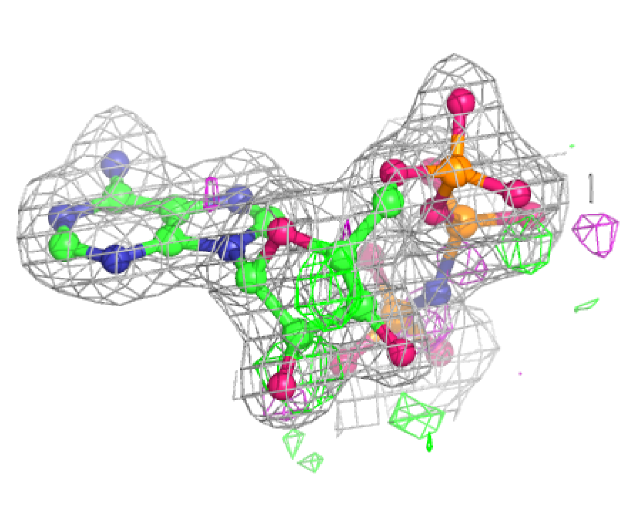

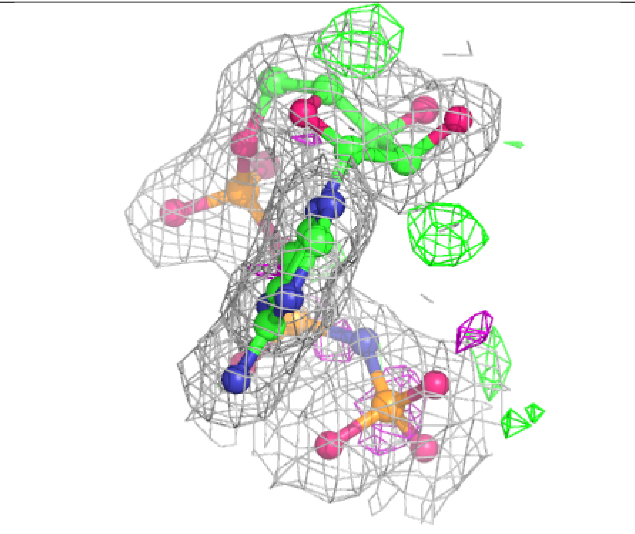

Electron density around ANP A 601:

2mF$_o$-DF$_c$ (at 0.7 rmsd) in gray
mF$_o$-DF$_c$ (at 3 rmsd) in purple (negative)
and green (positive)

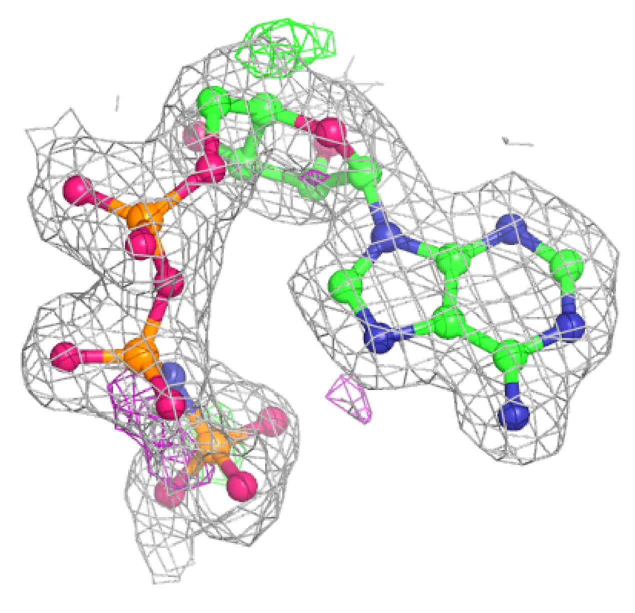

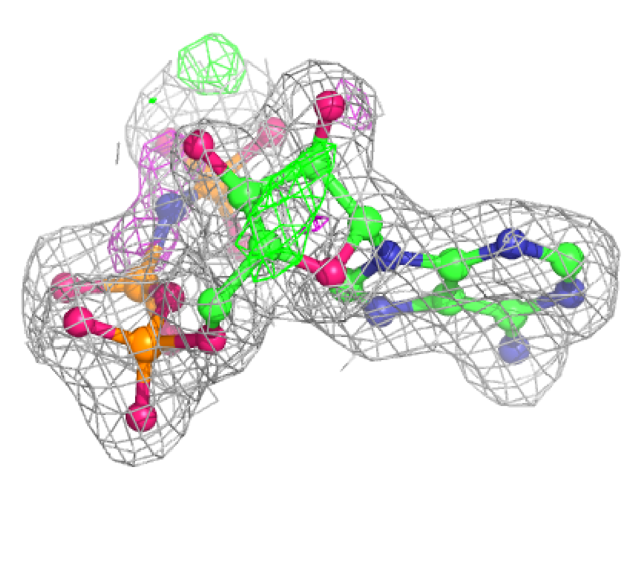

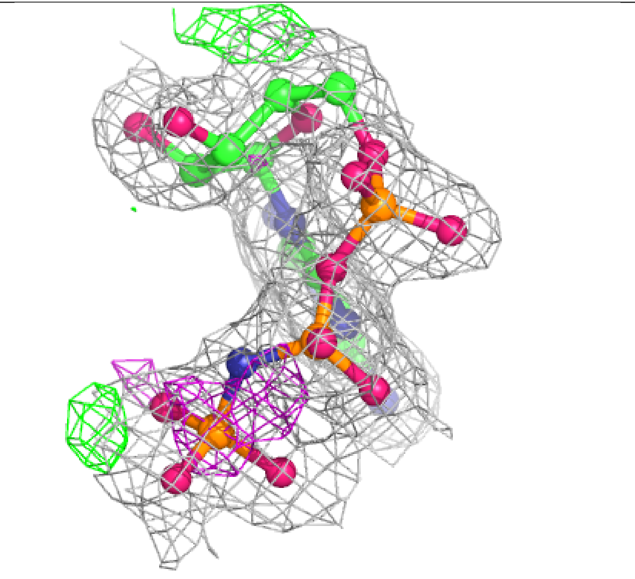

Electron density around HFG A 602:

$2mF_o$-DF_c (at 0.7 rmsd) in gray
mF_o-DF_c (at 3 rmsd) in purple (negative)
and green (positive)

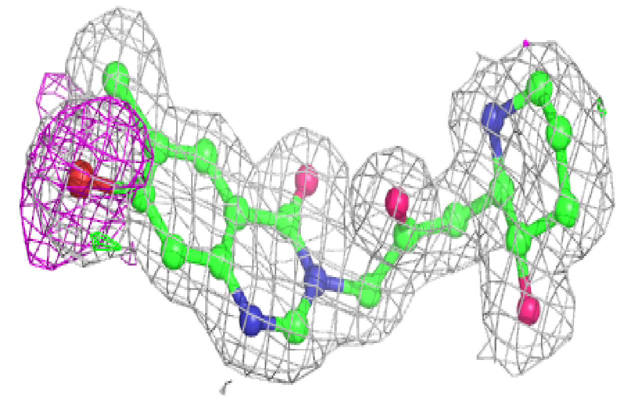

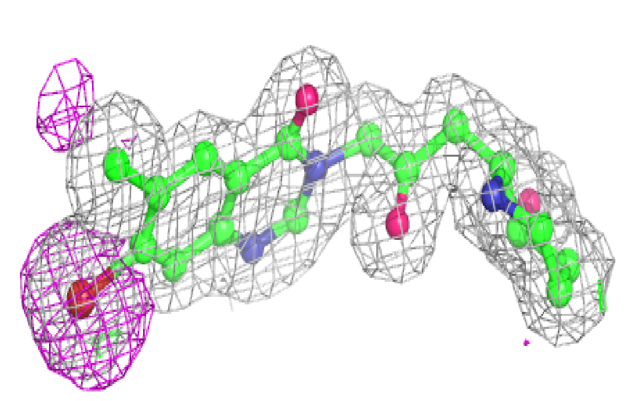

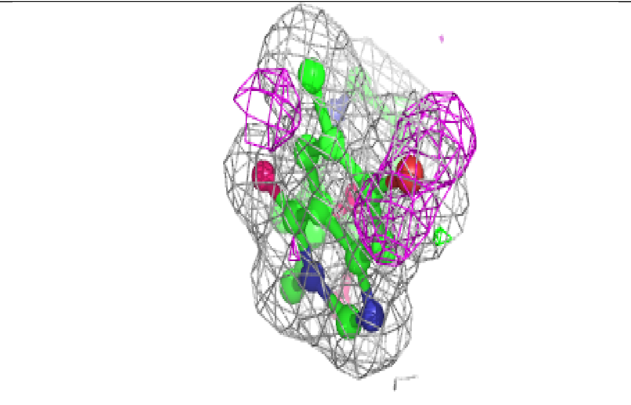

Electron density around HFG B 704:

$2mF_o$-DF_c (at 0.7 rmsd) in gray
mF_o-DF_c (at 3 rmsd) in purple (negative)
and green (positive)

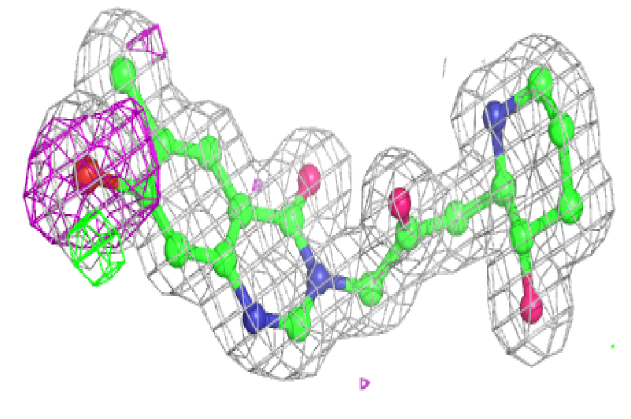

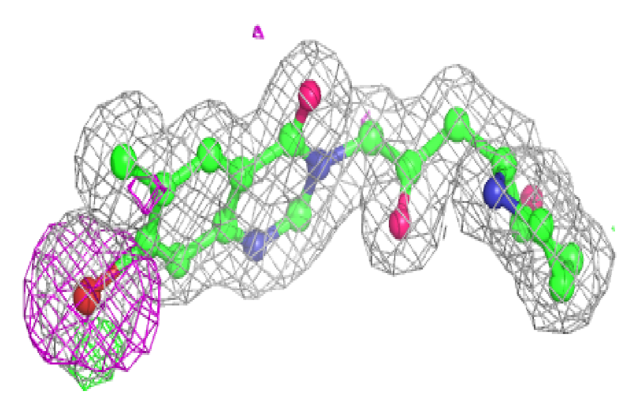

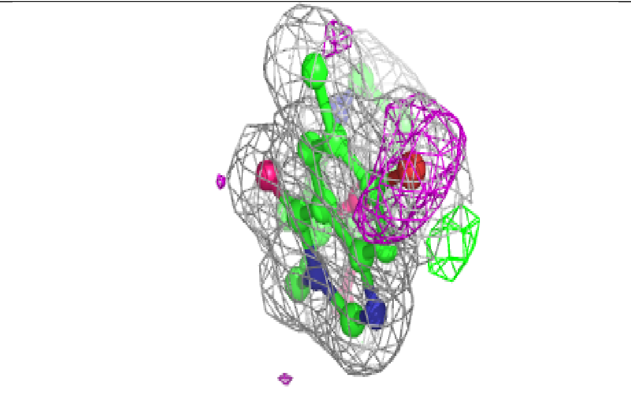

6.5 Other polymers (i)

There are no such residues in this entry.
